# Supplementary material for: Peer-Developed Modules on Basic Biostatistics and Evidence-Based Medicine Principles for Undergraduate Medical Education
Source: MedEdPORTAL. 2020 Nov 24;16:11026. doi: 10.15766/mep_2374-8265.11026 (PMC7703476; doi:10.15766/mep_2374-8265.11026)
Supplement: Supplementary file 1 — Module 1 Study Design and Bias.pptxModule 1 Problem Set.docxModule 1 Problem Set Answer Key.docxModule 1 Formative Quiz.docxModule 1 Formative Quiz Answer Key.docxModule 2 Interpreting Data from Clinical Trials.pptxModule 2 Problem Set.docxModule 2 Problem Set Answer Key.docxModule 2 Formative Quiz.docxModule 2 Formative Quiz Answer Key.docxModule 3 Diagnostic and Therapy Trial Results.pptxModule 3 Problem Set.docxModule 3 Problem Set Answer Key.docxModule 3 Formative Quiz.docxModule 3 Formative Quiz Answer Key.docxImplementation Guide.docxPostsession Evaluation Survey.docx [file mep_2374-8265.11026-s001.zip › A. Module 1 Study Design and Bias.pptx]

## Slide 1
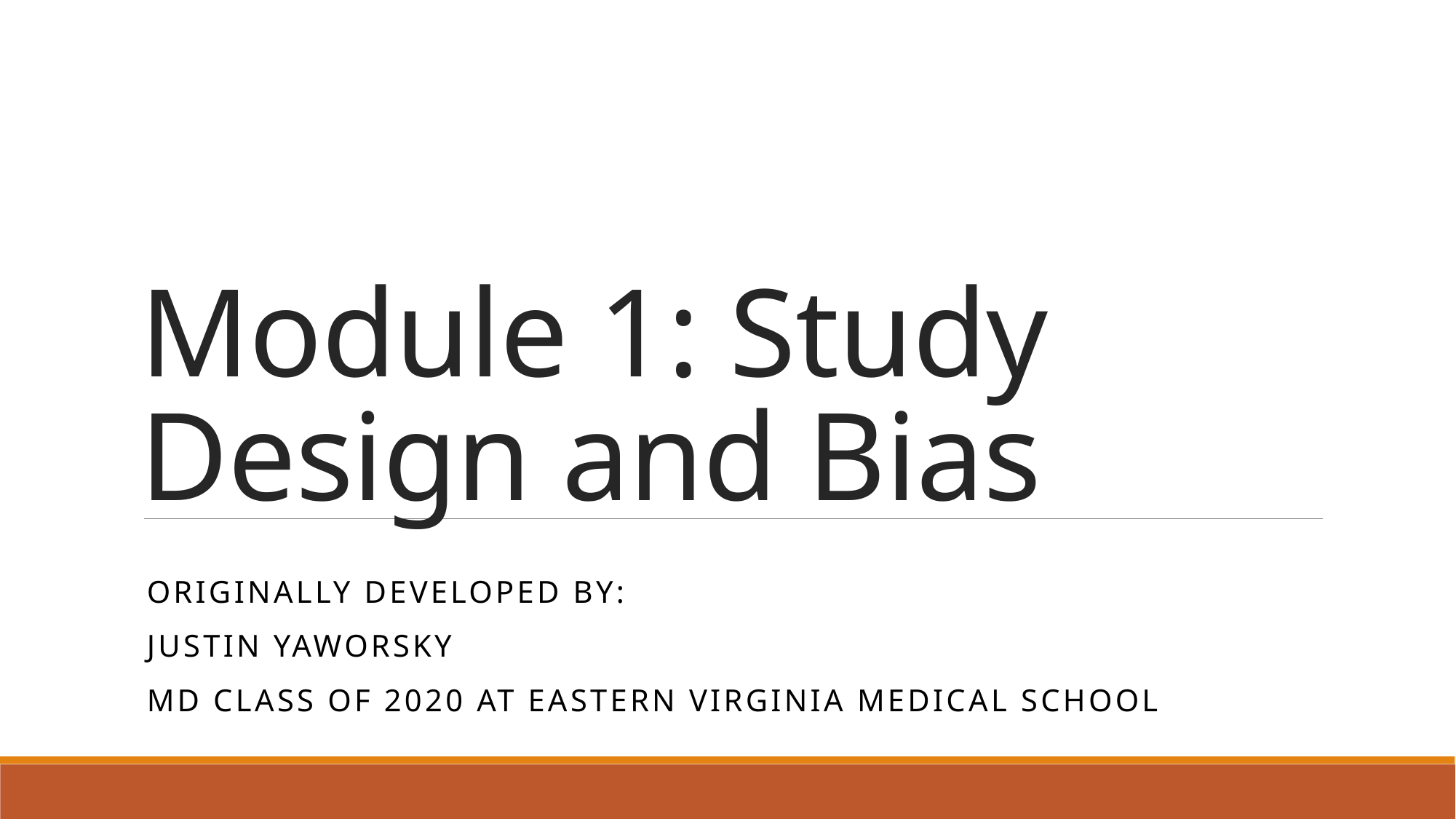

# Module 1: Study Design and Bias
Originally Developed By:
Justin Yaworsky
MD Class of 2020 AT Eastern Virginia Medical School

## Slide 2
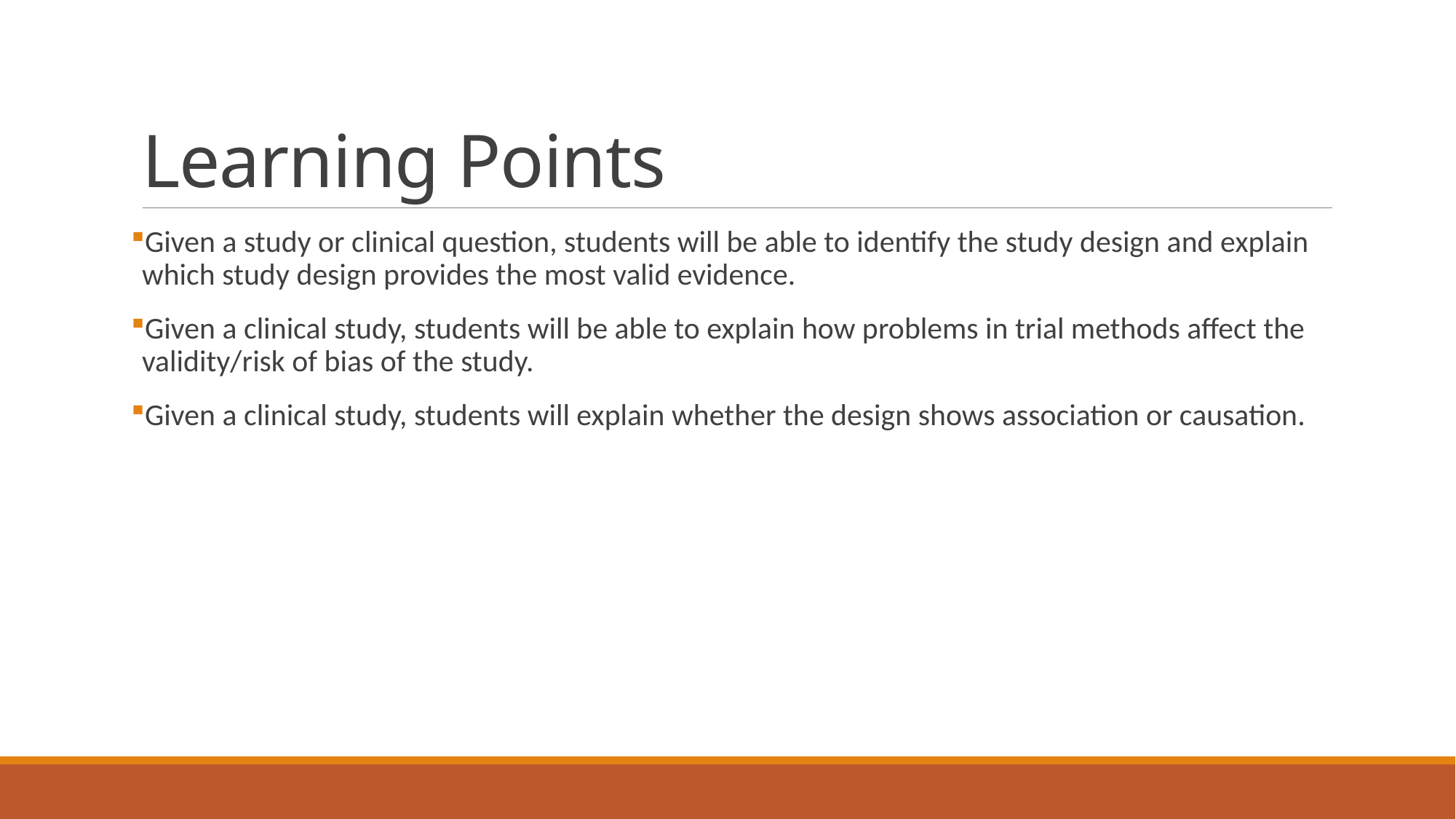

# Learning Points
Given a study or clinical question, students will be able to identify the study design and explain which study design provides the most valid evidence.
Given a clinical study, students will be able to explain how problems in trial methods affect the validity/risk of bias of the study.
Given a clinical study, students will explain whether the design shows association or causation.

## Slide 3
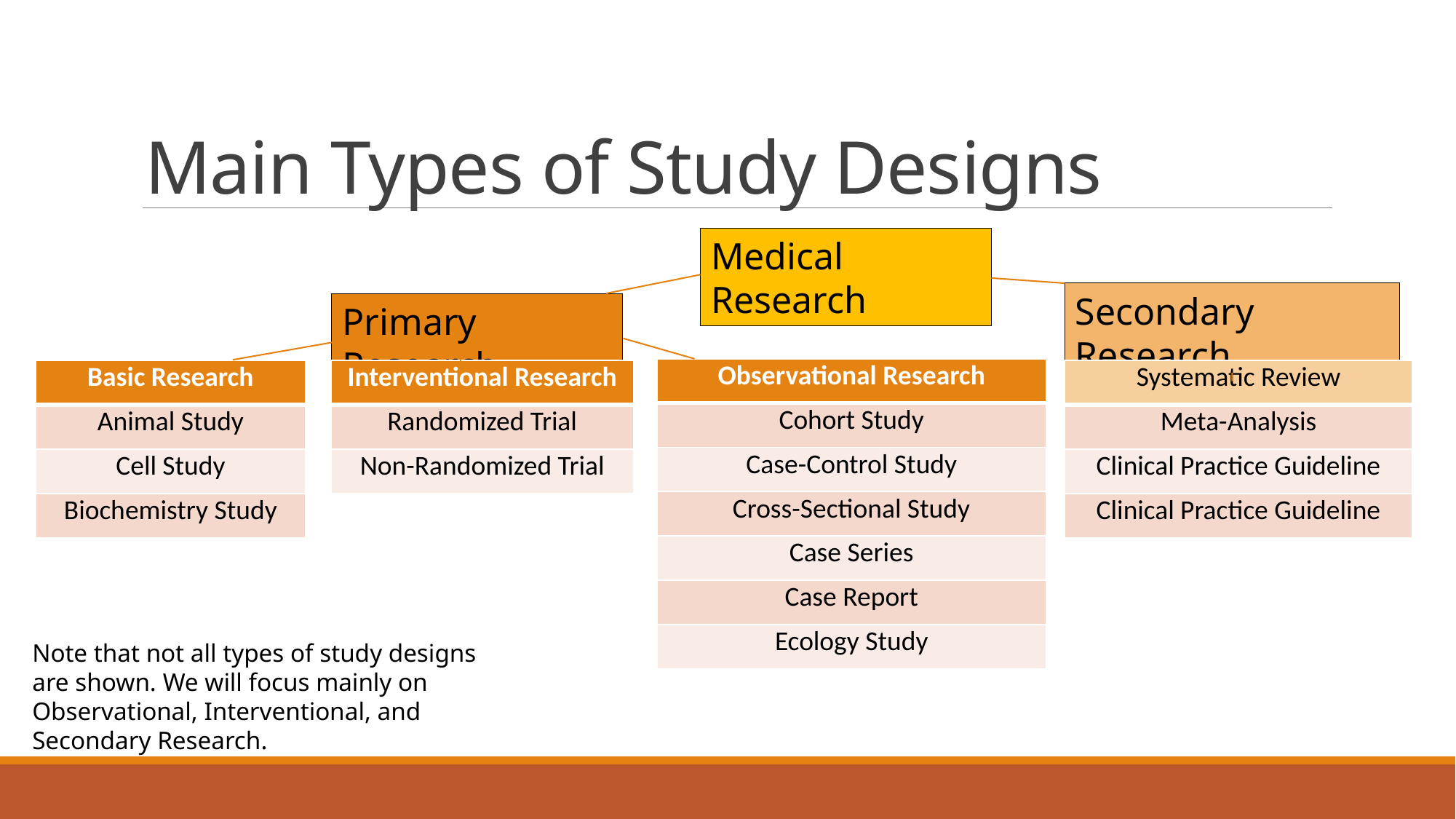

# Main Types of Study Designs
Medical Research
Secondary Research
Primary Research
| Observational Research |
| --- |
| Cohort Study |
| Case-Control Study |
| Cross-Sectional Study |
| Case Series |
| Case Report |
| Ecology Study |
| Basic Research |
| --- |
| Animal Study |
| Cell Study |
| Biochemistry Study |
| Interventional Research |
| --- |
| Randomized Trial |
| Non-Randomized Trial |
| Systematic Review |
| --- |
| Meta-Analysis |
| Clinical Practice Guideline |
| Clinical Practice Guideline |
Note that not all types of study designs are shown. We will focus mainly on Observational, Interventional, and Secondary Research.

## Slide 4
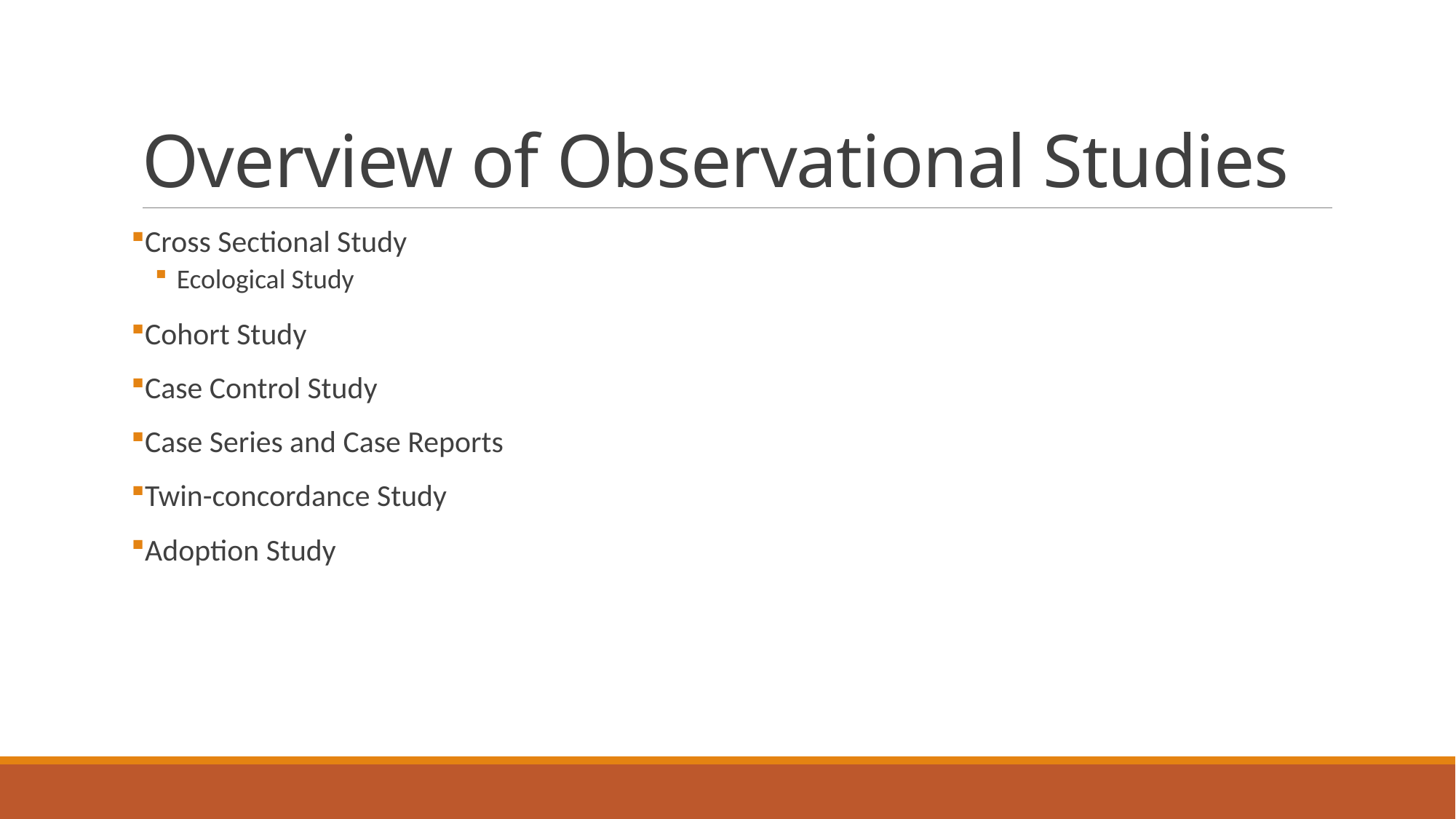

# Overview of Observational Studies
Cross Sectional Study
Ecological Study
Cohort Study
Case Control Study
Case Series and Case Reports
Twin-concordance Study
Adoption Study

## Slide 5
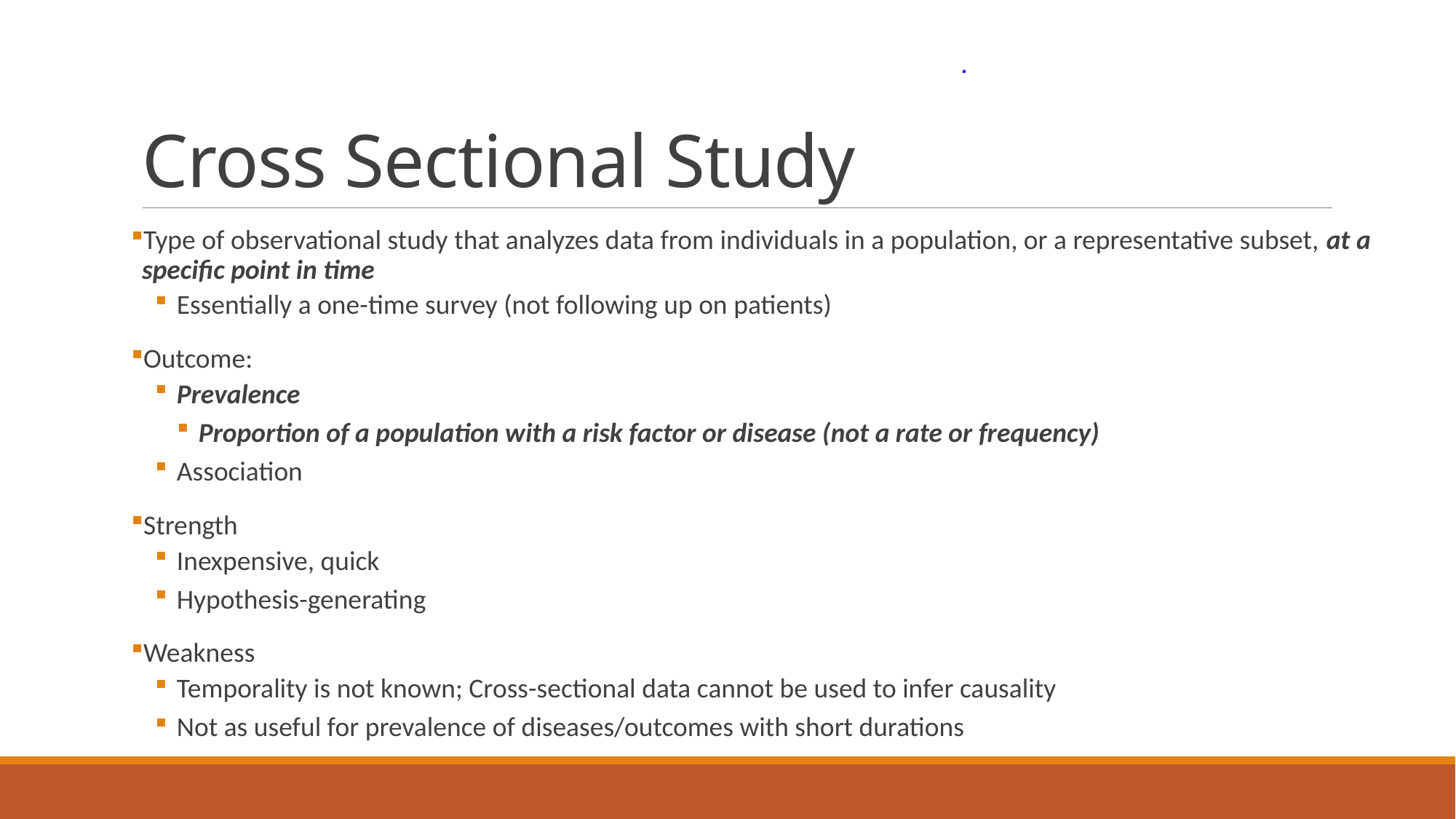

# Cross Sectional Study
Type of observational study that analyzes data from individuals in a population, or a representative subset, at a specific point in time
Essentially a one-time survey (not following up on patients)
Outcome:
Prevalence
Proportion of a population with a risk factor or disease (not a rate or frequency)
Association
Strength
Inexpensive, quick
Hypothesis-generating
Weakness
Temporality is not known; Cross-sectional data cannot be used to infer causality
Not as useful for prevalence of diseases/outcomes with short durations

## Slide 6
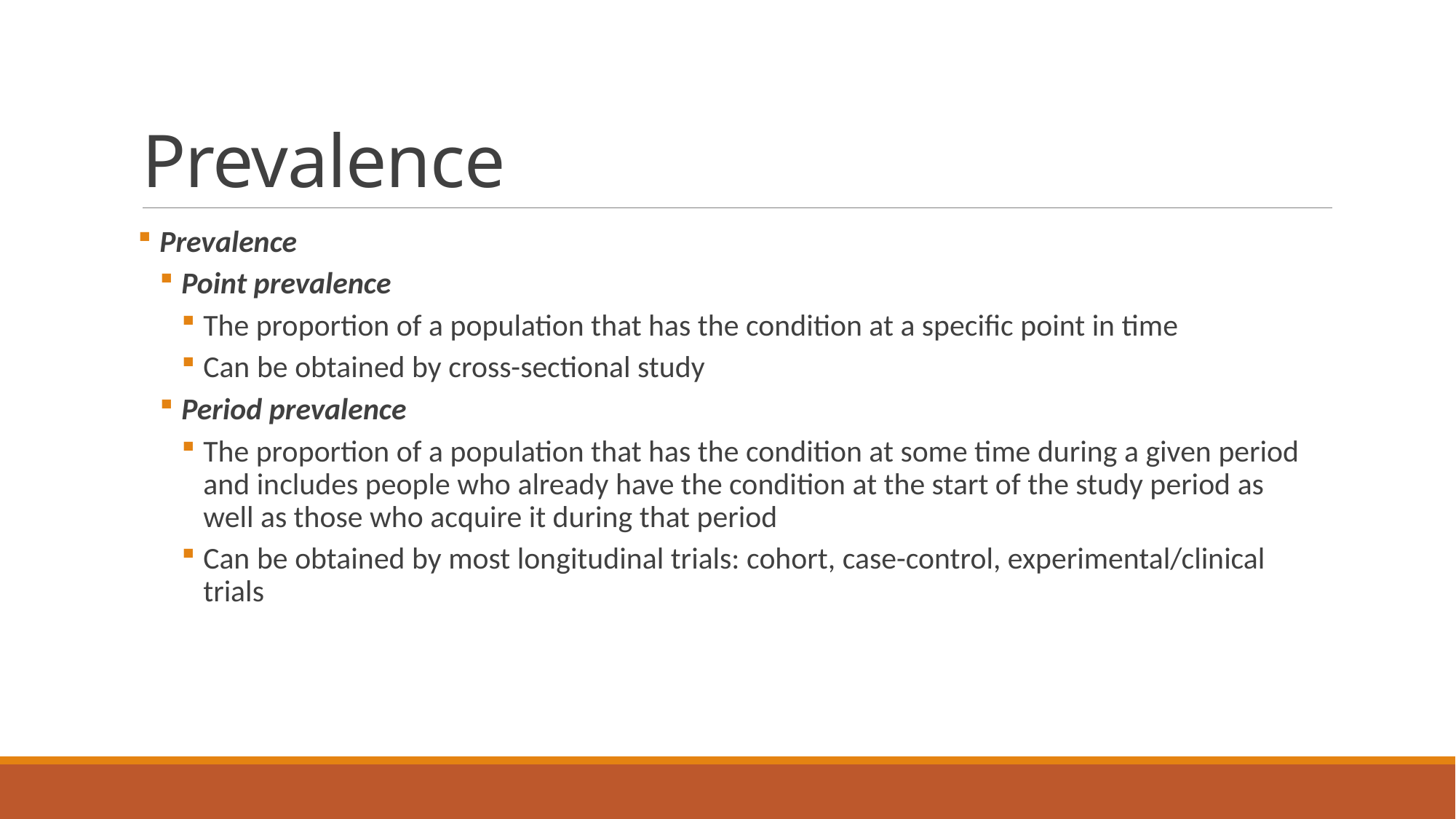

# Prevalence
Prevalence
Point prevalence
The proportion of a population that has the condition at a specific point in time
Can be obtained by cross-sectional study
Period prevalence
The proportion of a population that has the condition at some time during a given period and includes people who already have the condition at the start of the study period as well as those who acquire it during that period
Can be obtained by most longitudinal trials: cohort, case-control, experimental/clinical trials

## Slide 7
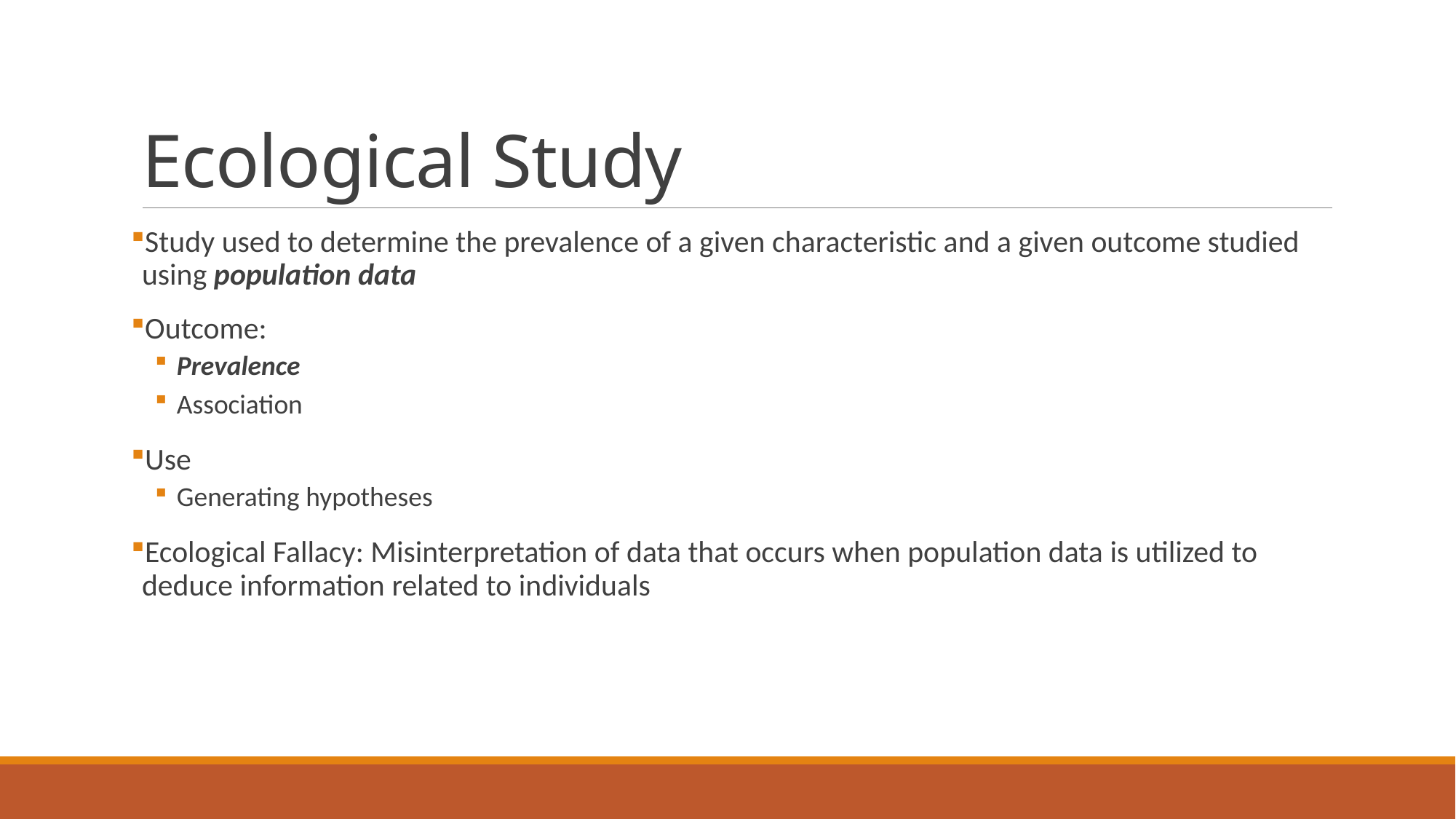

# Ecological Study
Study used to determine the prevalence of a given characteristic and a given outcome studied using population data
Outcome:
Prevalence
Association
Use
Generating hypotheses
Ecological Fallacy: Misinterpretation of data that occurs when population data is utilized to deduce information related to individuals

## Slide 8
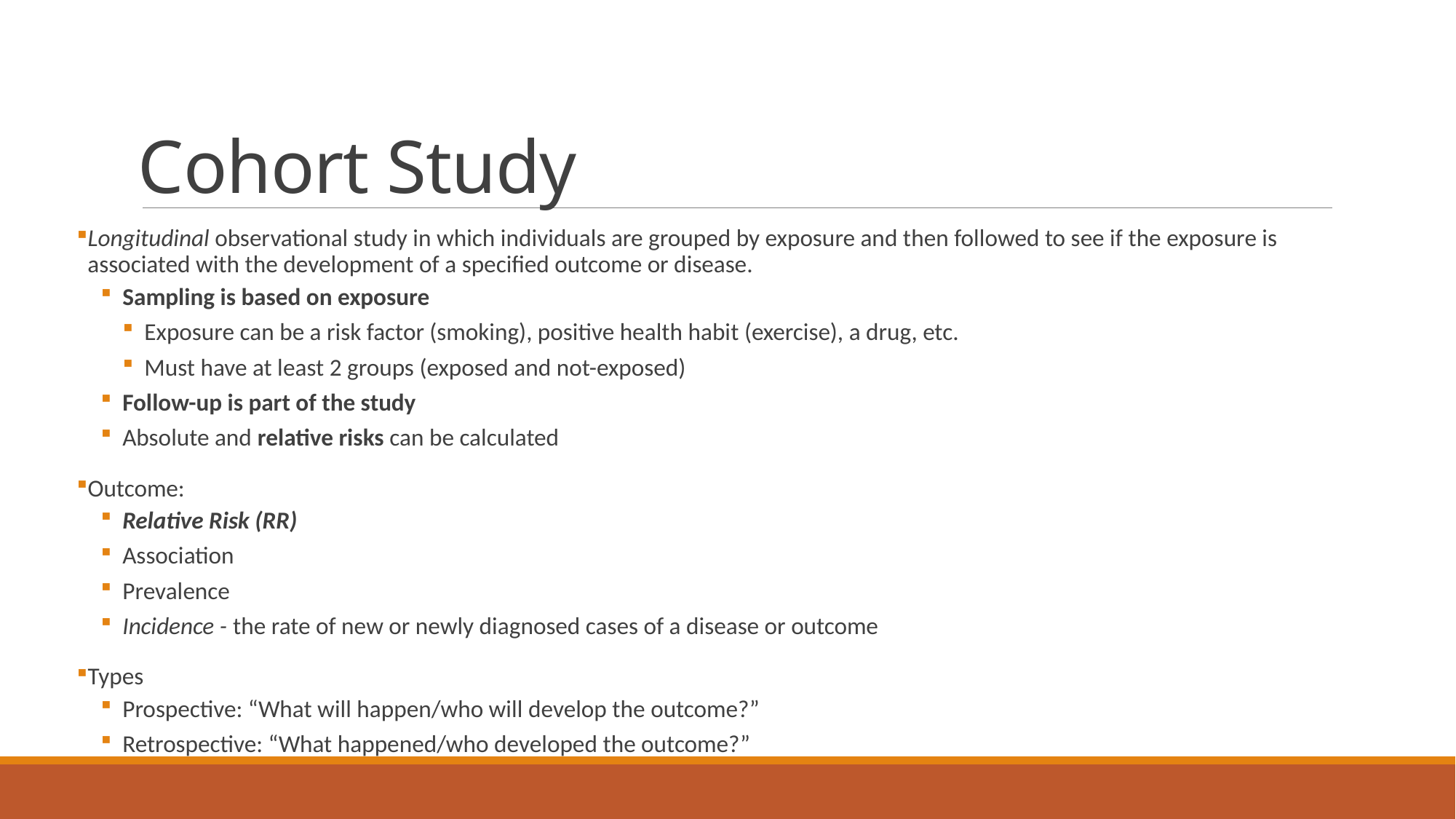

# Cohort Study
Longitudinal observational study in which individuals are grouped by exposure and then followed to see if the exposure is associated with the development of a specified outcome or disease.
Sampling is based on exposure
Exposure can be a risk factor (smoking), positive health habit (exercise), a drug, etc.
Must have at least 2 groups (exposed and not-exposed)
Follow-up is part of the study
Absolute and relative risks can be calculated
Outcome:
Relative Risk (RR)
Association
Prevalence
Incidence - the rate of new or newly diagnosed cases of a disease or outcome
Types
Prospective: “What will happen/who will develop the outcome?”
Retrospective: “What happened/who developed the outcome?”

## Slide 9
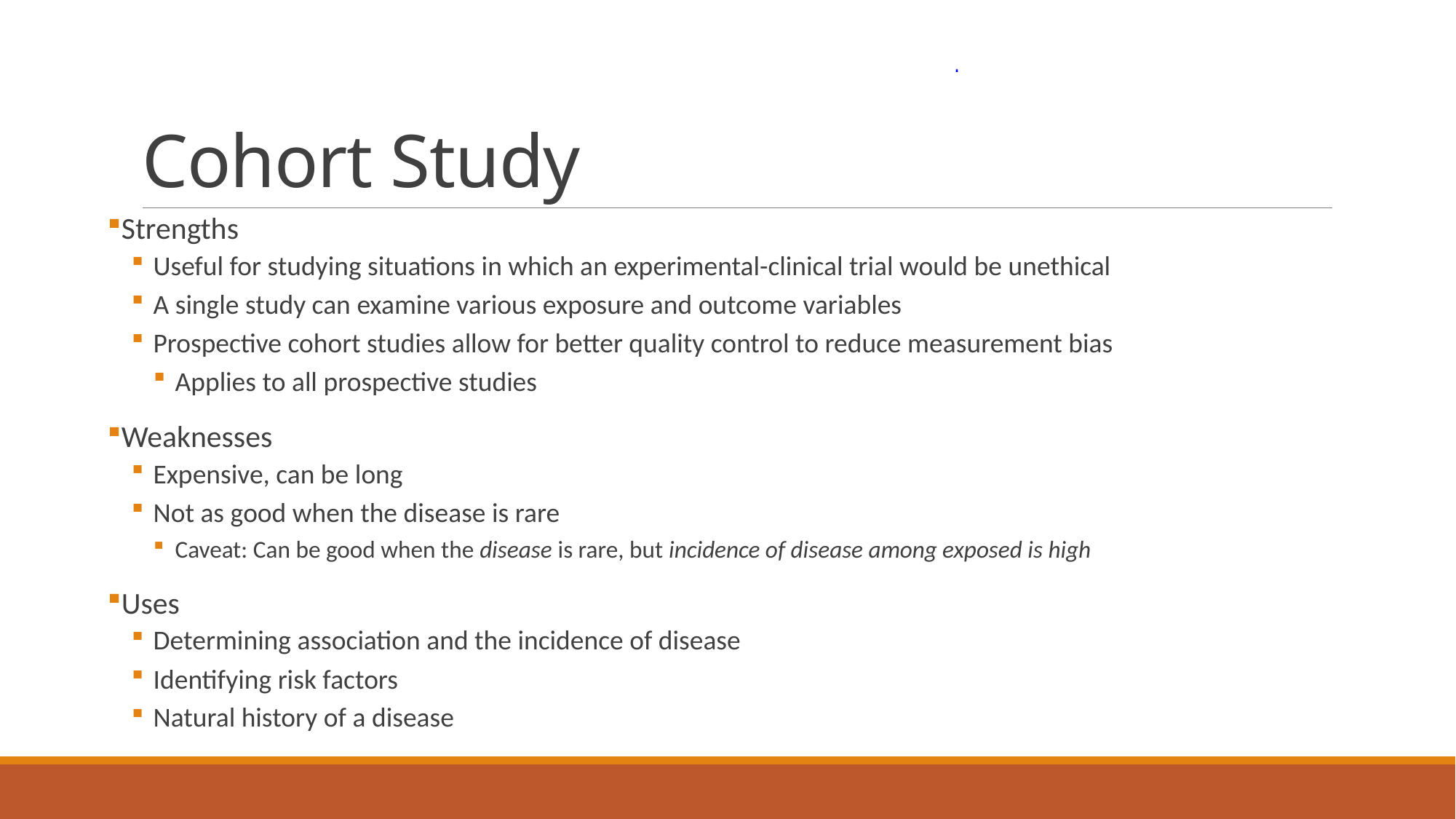

# Cohort Study
Strengths
Useful for studying situations in which an experimental-clinical trial would be unethical
A single study can examine various exposure and outcome variables
Prospective cohort studies allow for better quality control to reduce measurement bias
Applies to all prospective studies
Weaknesses
Expensive, can be long
Not as good when the disease is rare
Caveat: Can be good when the disease is rare, but incidence of disease among exposed is high
Uses
Determining association and the incidence of disease
Identifying risk factors
Natural history of a disease

## Slide 10
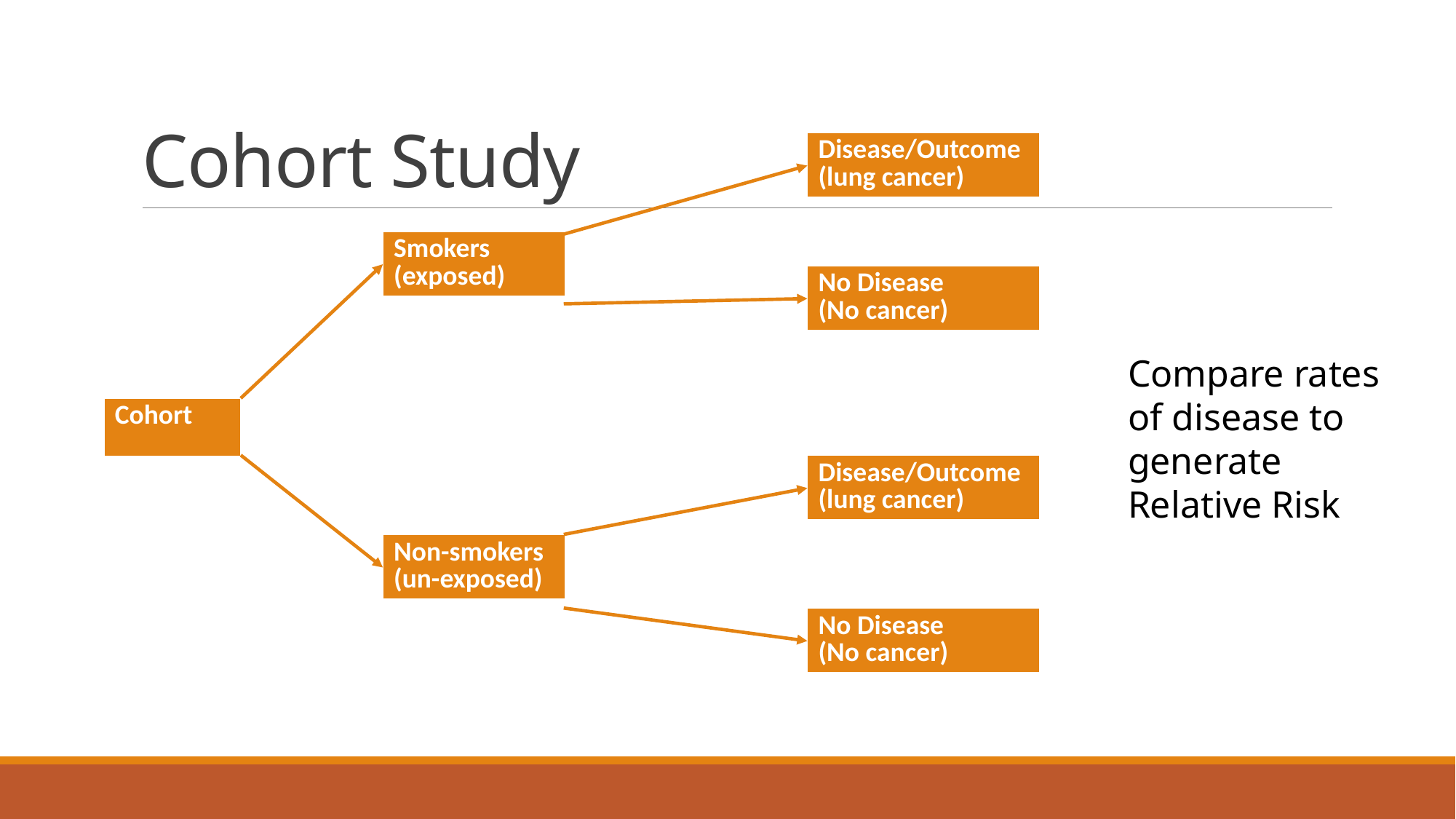

# Cohort Study
| Disease/Outcome (lung cancer) |
| --- |
| Smokers (exposed) |
| --- |
| No Disease (No cancer) |
| --- |
Compare rates of disease to generate Relative Risk
| Cohort |
| --- |
| Disease/Outcome (lung cancer) |
| --- |
| Non-smokers (un-exposed) |
| --- |
| No Disease (No cancer) |
| --- |

## Slide 11
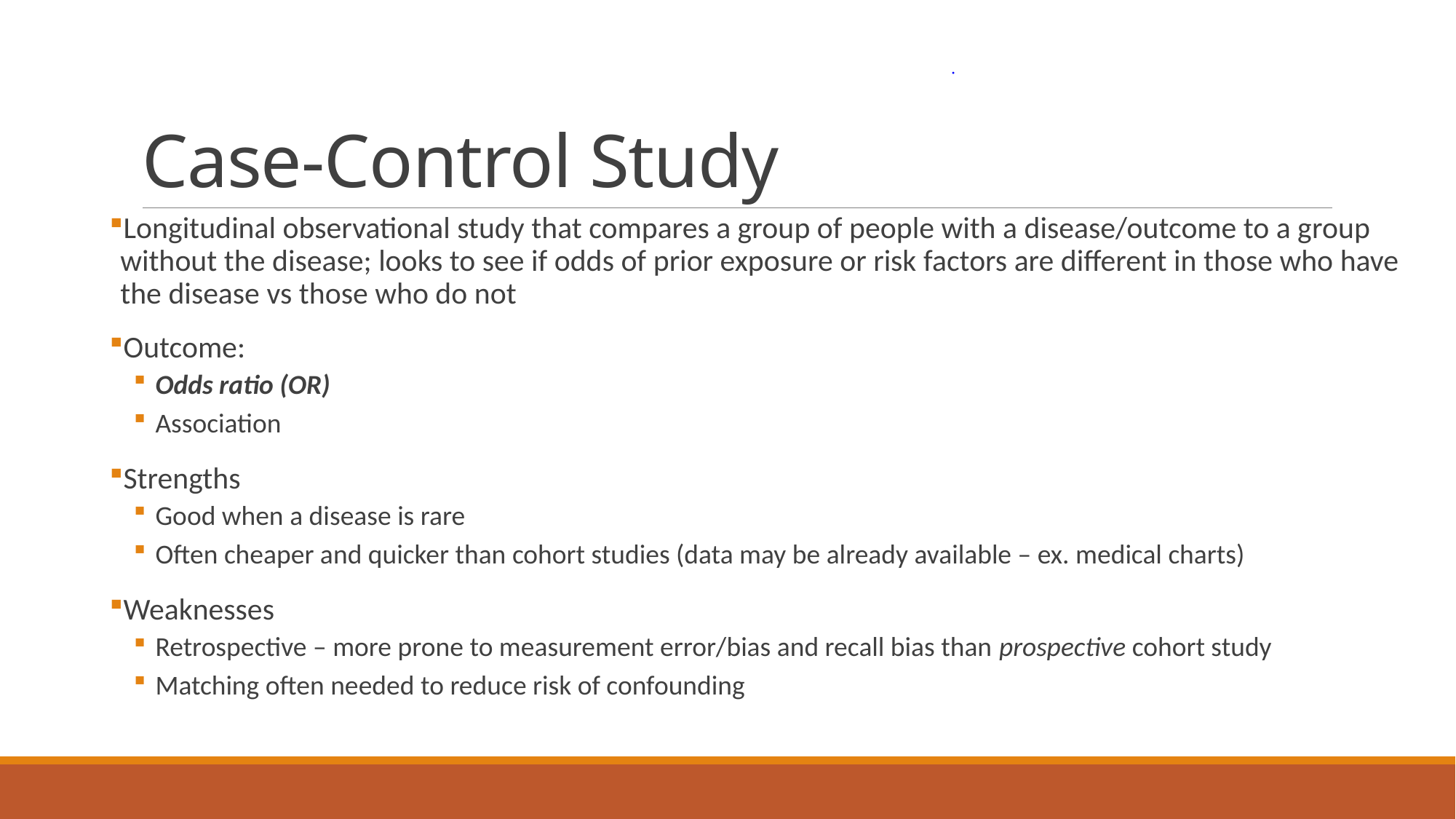

# Case-Control Study
Longitudinal observational study that compares a group of people with a disease/outcome to a group without the disease; looks to see if odds of prior exposure or risk factors are different in those who have the disease vs those who do not
Outcome:
Odds ratio (OR)
Association
Strengths
Good when a disease is rare
Often cheaper and quicker than cohort studies (data may be already available – ex. medical charts)
Weaknesses
Retrospective – more prone to measurement error/bias and recall bias than prospective cohort study
Matching often needed to reduce risk of confounding

## Slide 12
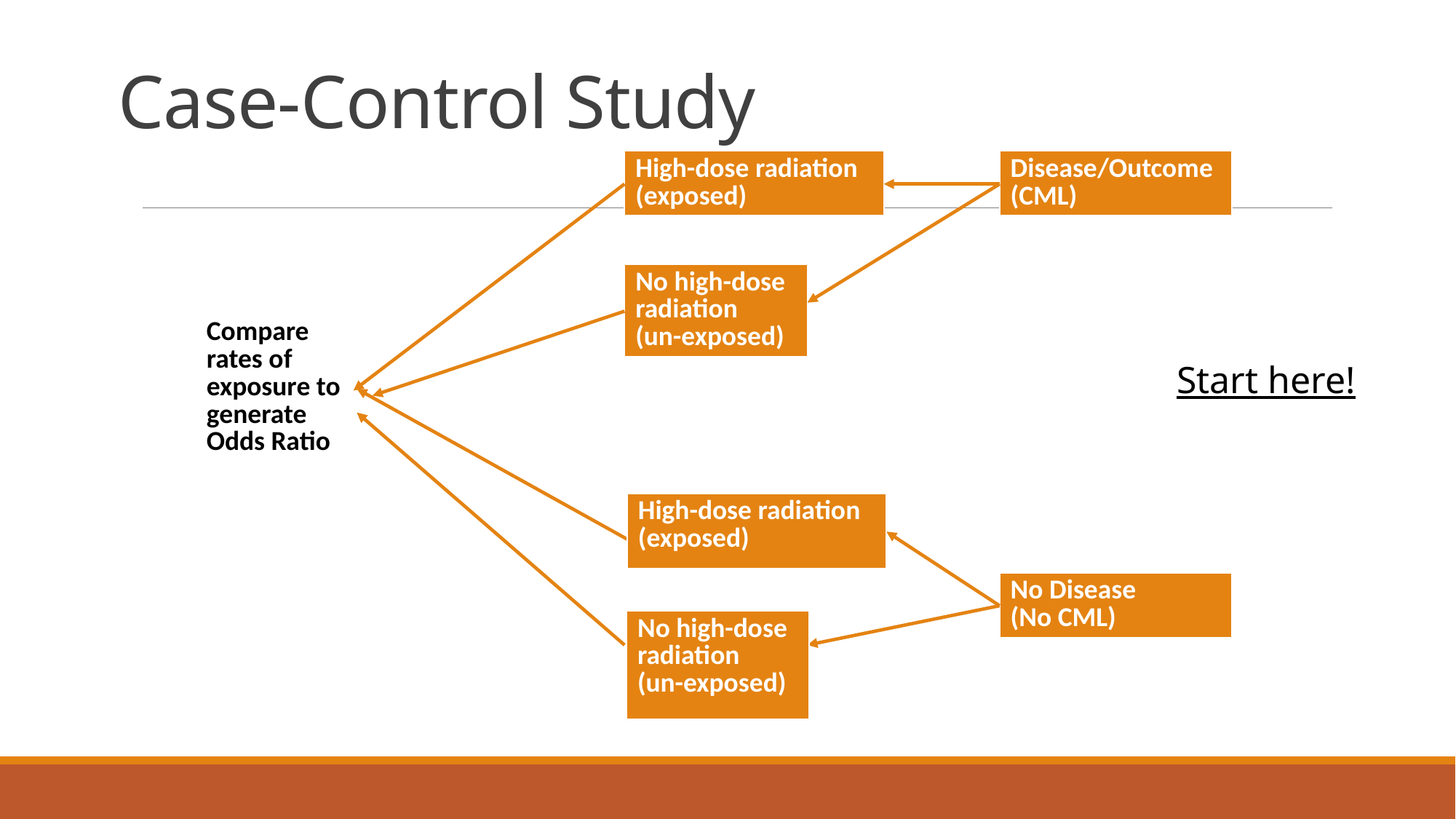

# Case-Control Study
| High-dose radiation (exposed) |
| --- |
| Disease/Outcome (CML) |
| --- |
| No high-dose radiation (un-exposed) |
| --- |
| Compare rates of exposure to generate Odds Ratio |
| --- |
Start here!
| High-dose radiation (exposed) |
| --- |
| No Disease (No CML) |
| --- |
| No high-dose radiation (un-exposed) |
| --- |

## Slide 13
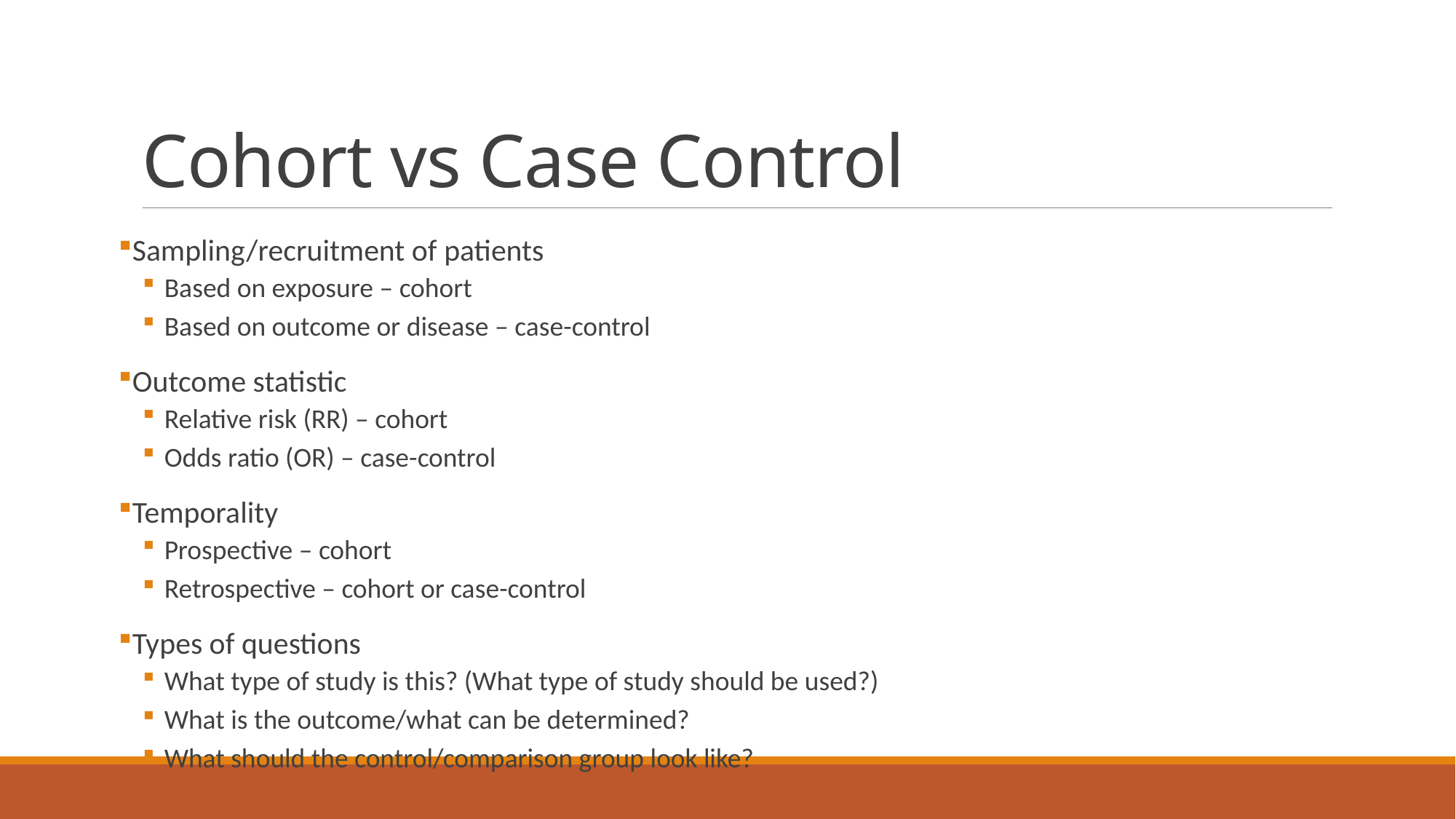

# Cohort vs Case Control
Sampling/recruitment of patients
Based on exposure – cohort
Based on outcome or disease – case-control
Outcome statistic
Relative risk (RR) – cohort
Odds ratio (OR) – case-control
Temporality
Prospective – cohort
Retrospective – cohort or case-control
Types of questions
What type of study is this? (What type of study should be used?)
What is the outcome/what can be determined?
What should the control/comparison group look like?

## Slide 14
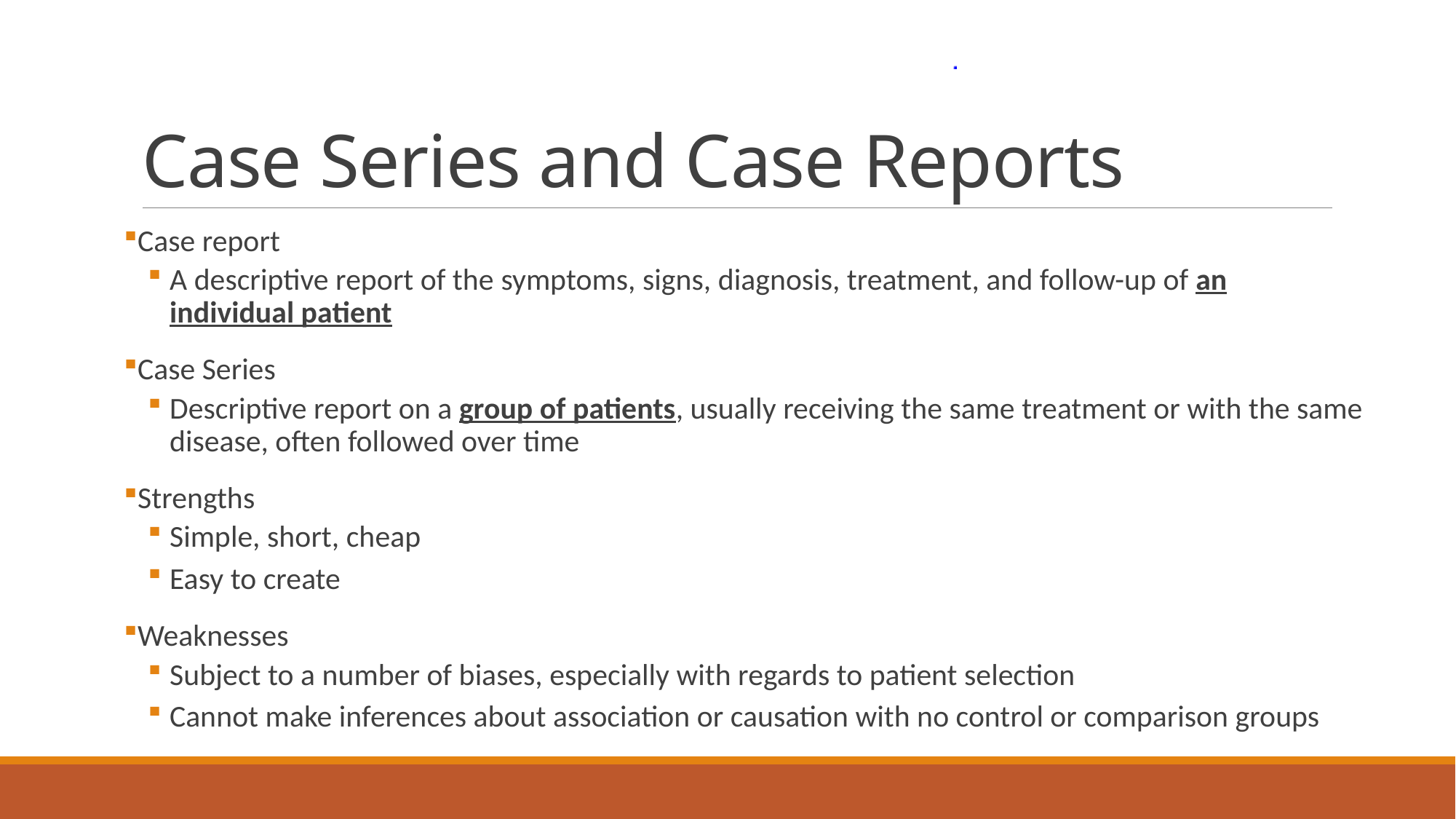

# Case Series and Case Reports
Case report
A descriptive report of the symptoms, signs, diagnosis, treatment, and follow-up of an individual patient
Case Series
Descriptive report on a group of patients, usually receiving the same treatment or with the same disease, often followed over time
Strengths
Simple, short, cheap
Easy to create
Weaknesses
Subject to a number of biases, especially with regards to patient selection
Cannot make inferences about association or causation with no control or comparison groups

## Slide 15
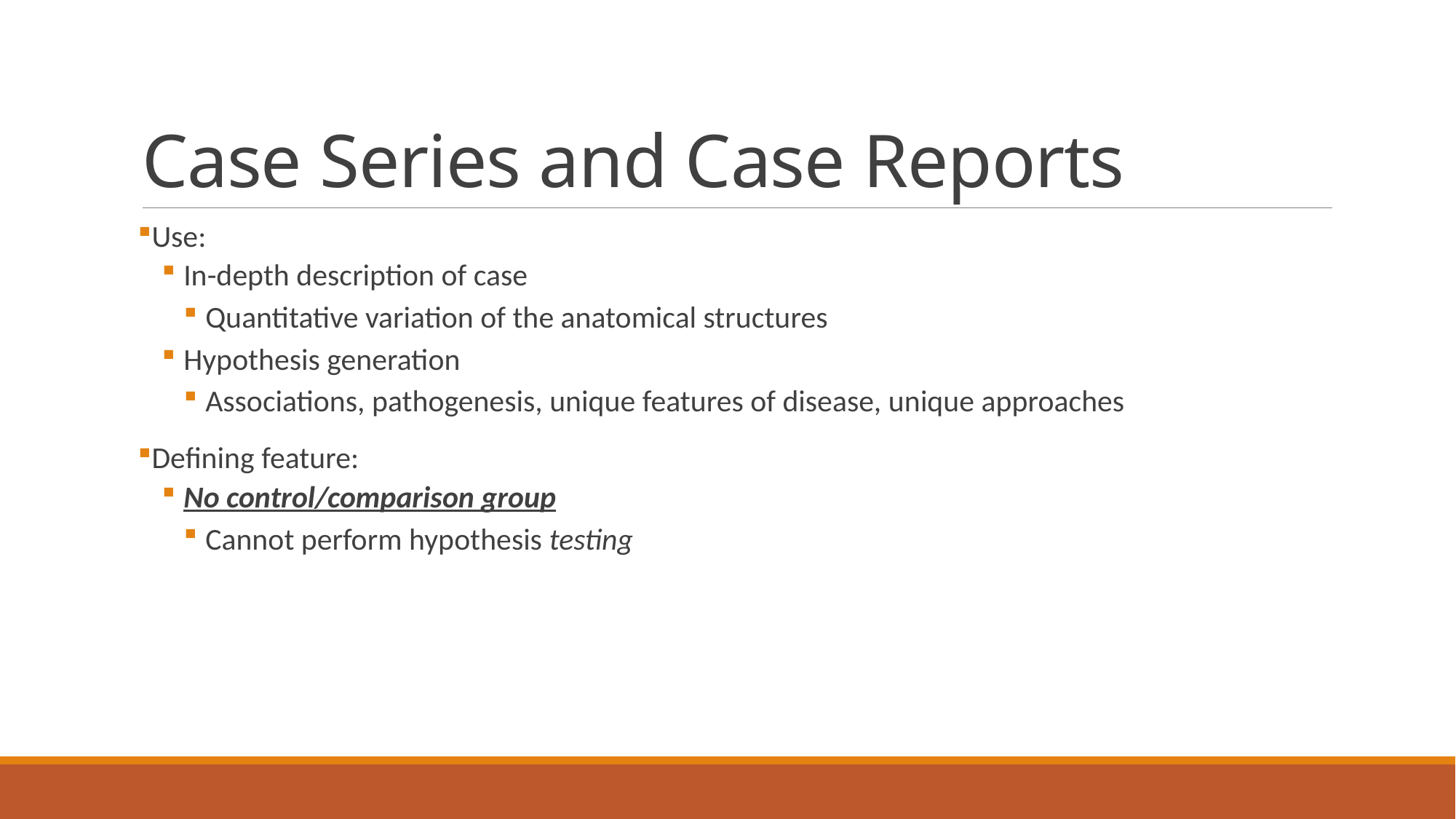

# Case Series and Case Reports
Use:
In-depth description of case
Quantitative variation of the anatomical structures
Hypothesis generation
Associations, pathogenesis, unique features of disease, unique approaches
Defining feature:
No control/comparison group
Cannot perform hypothesis testing

## Slide 16
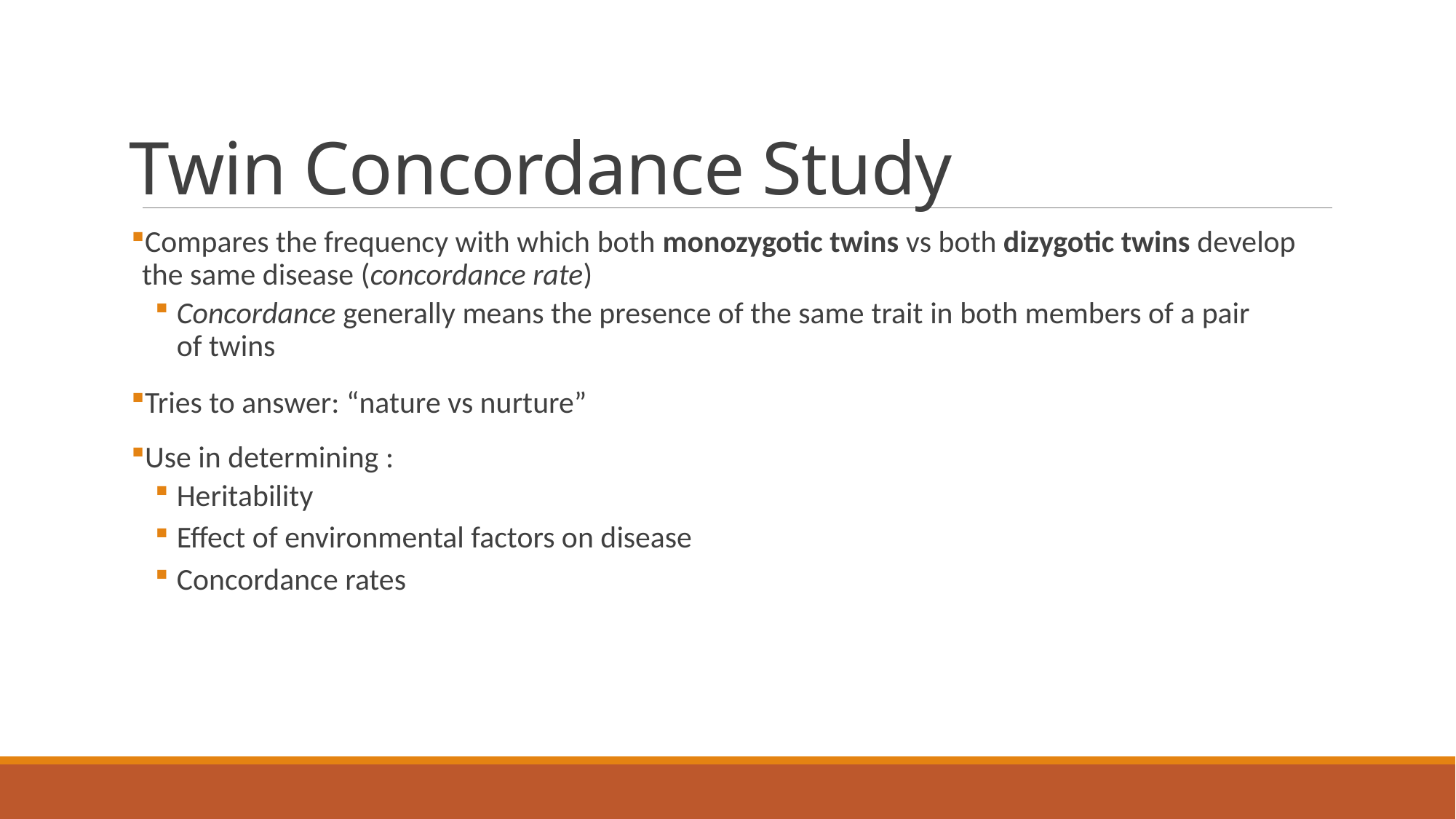

# Twin Concordance Study
Compares the frequency with which both monozygotic twins vs both dizygotic twins develop the same disease (concordance rate)
Concordance generally means the presence of the same trait in both members of a pair of twins
Tries to answer: “nature vs nurture”
Use in determining :
Heritability
Effect of environmental factors on disease
Concordance rates

## Slide 17
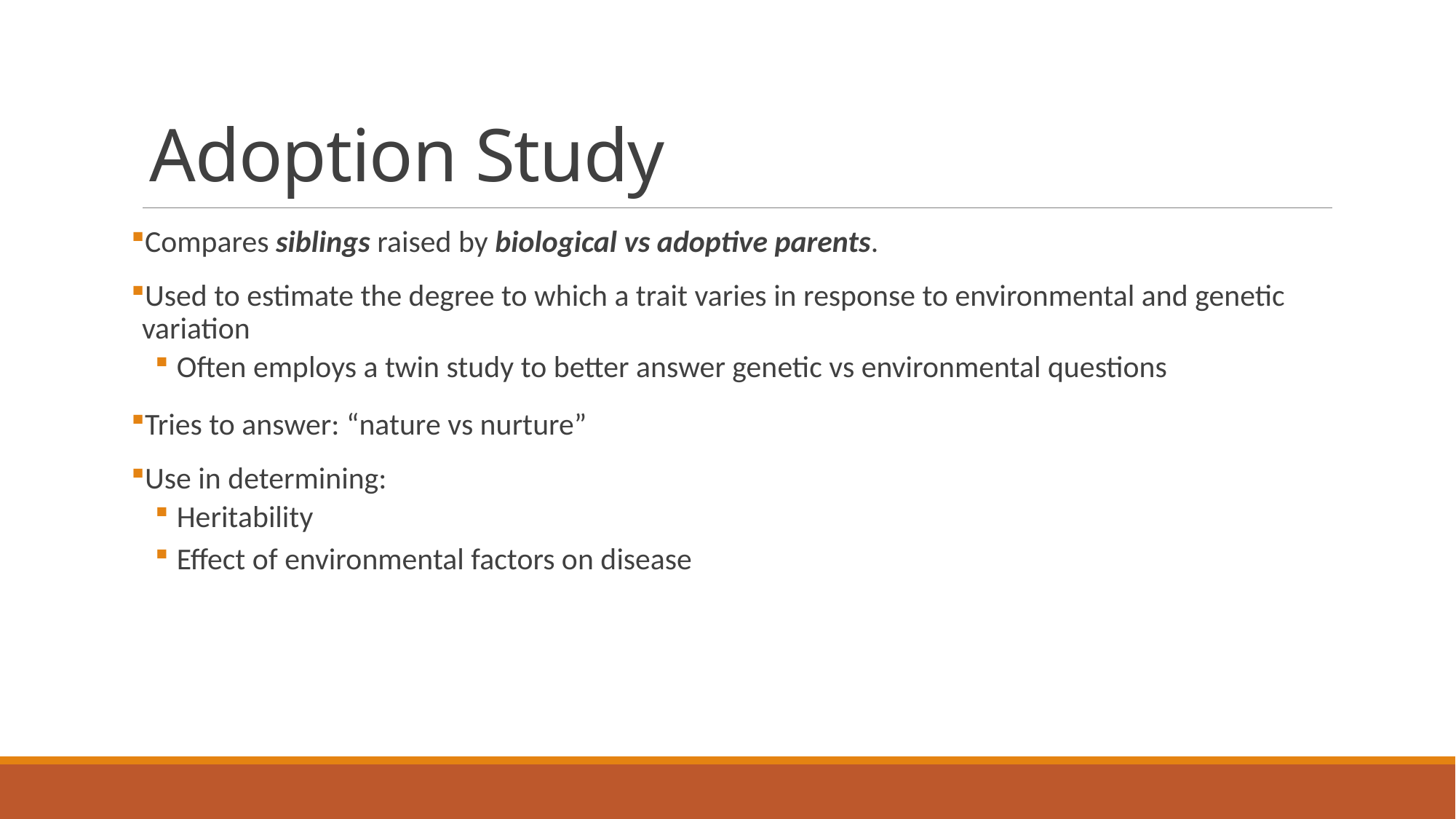

# Adoption Study
Compares siblings raised by biological vs adoptive parents.
Used to estimate the degree to which a trait varies in response to environmental and genetic variation
Often employs a twin study to better answer genetic vs environmental questions
Tries to answer: “nature vs nurture”
Use in determining:
Heritability
Effect of environmental factors on disease

## Slide 18
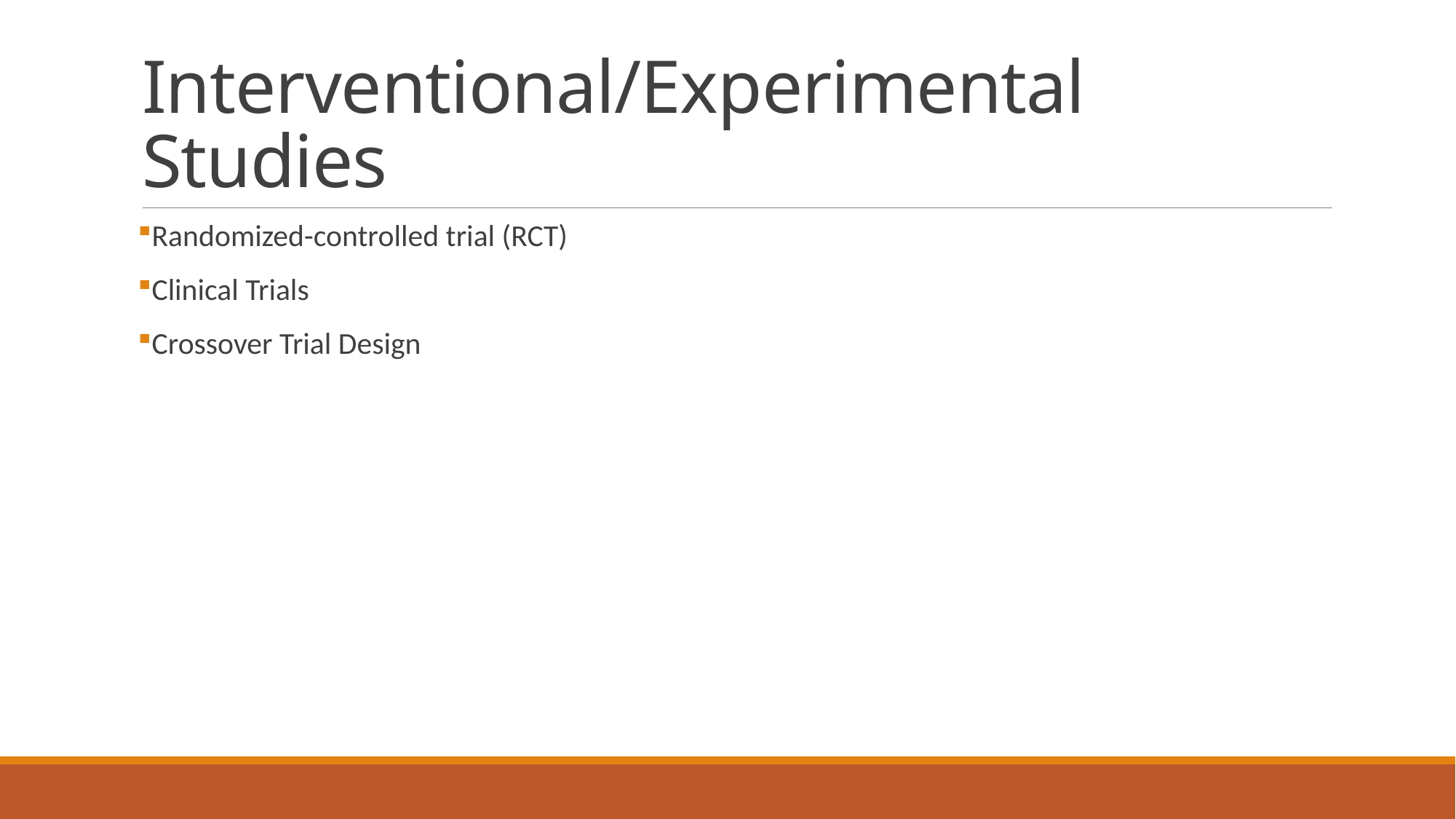

# Interventional/Experimental Studies
Randomized-controlled trial (RCT)
Clinical Trials
Crossover Trial Design

## Slide 19
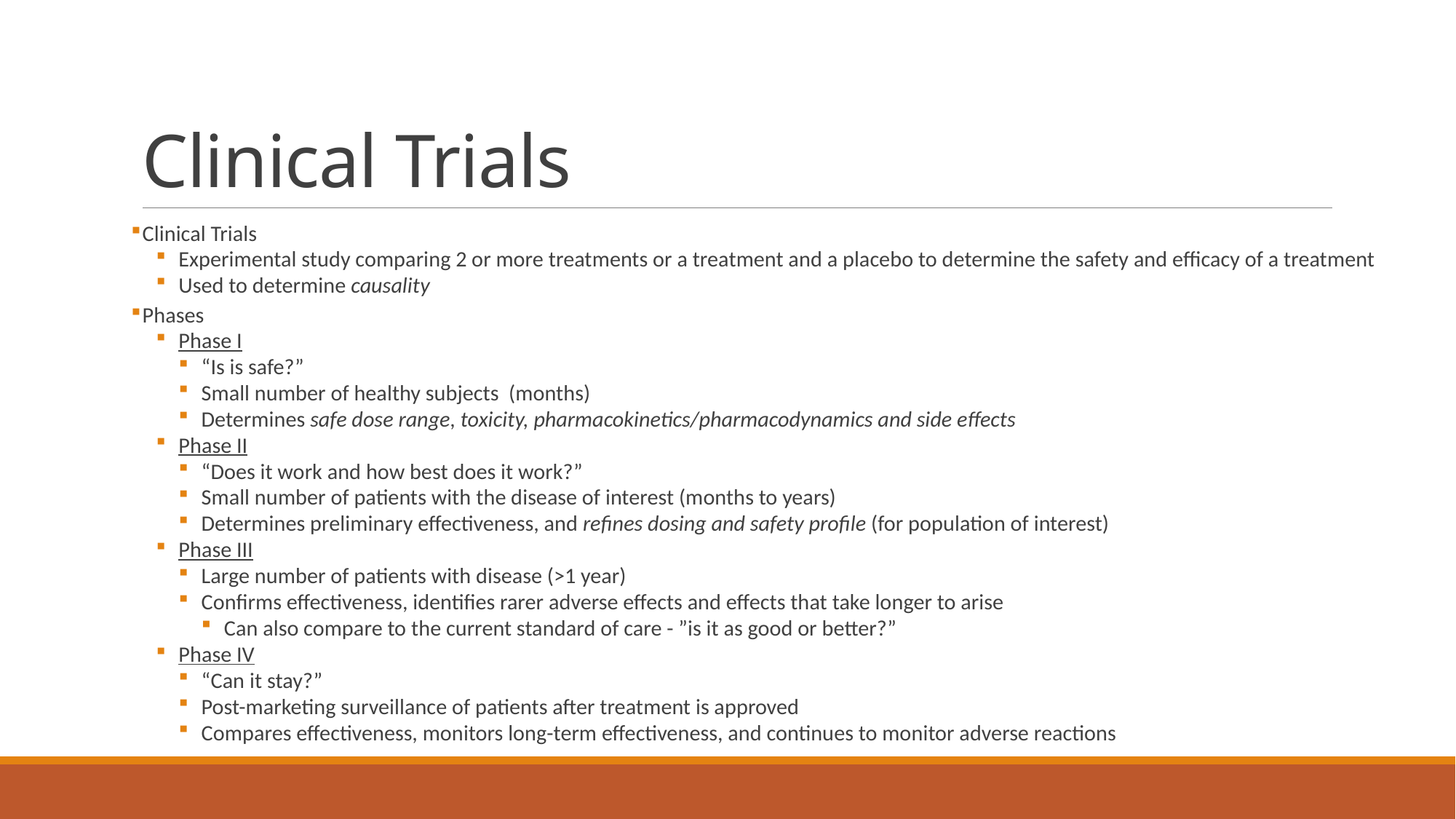

# Clinical Trials
Clinical Trials
Experimental study comparing 2 or more treatments or a treatment and a placebo to determine the safety and efficacy of a treatment
Used to determine causality
Phases
Phase I
“Is is safe?”
Small number of healthy subjects (months)
Determines safe dose range, toxicity, pharmacokinetics/pharmacodynamics and side effects
Phase II
“Does it work and how best does it work?”
Small number of patients with the disease of interest (months to years)
Determines preliminary effectiveness, and refines dosing and safety profile (for population of interest)
Phase III
Large number of patients with disease (>1 year)
Confirms effectiveness, identifies rarer adverse effects and effects that take longer to arise
Can also compare to the current standard of care - ”is it as good or better?”
Phase IV
“Can it stay?”
Post-marketing surveillance of patients after treatment is approved
Compares effectiveness, monitors long-term effectiveness, and continues to monitor adverse reactions

## Slide 20
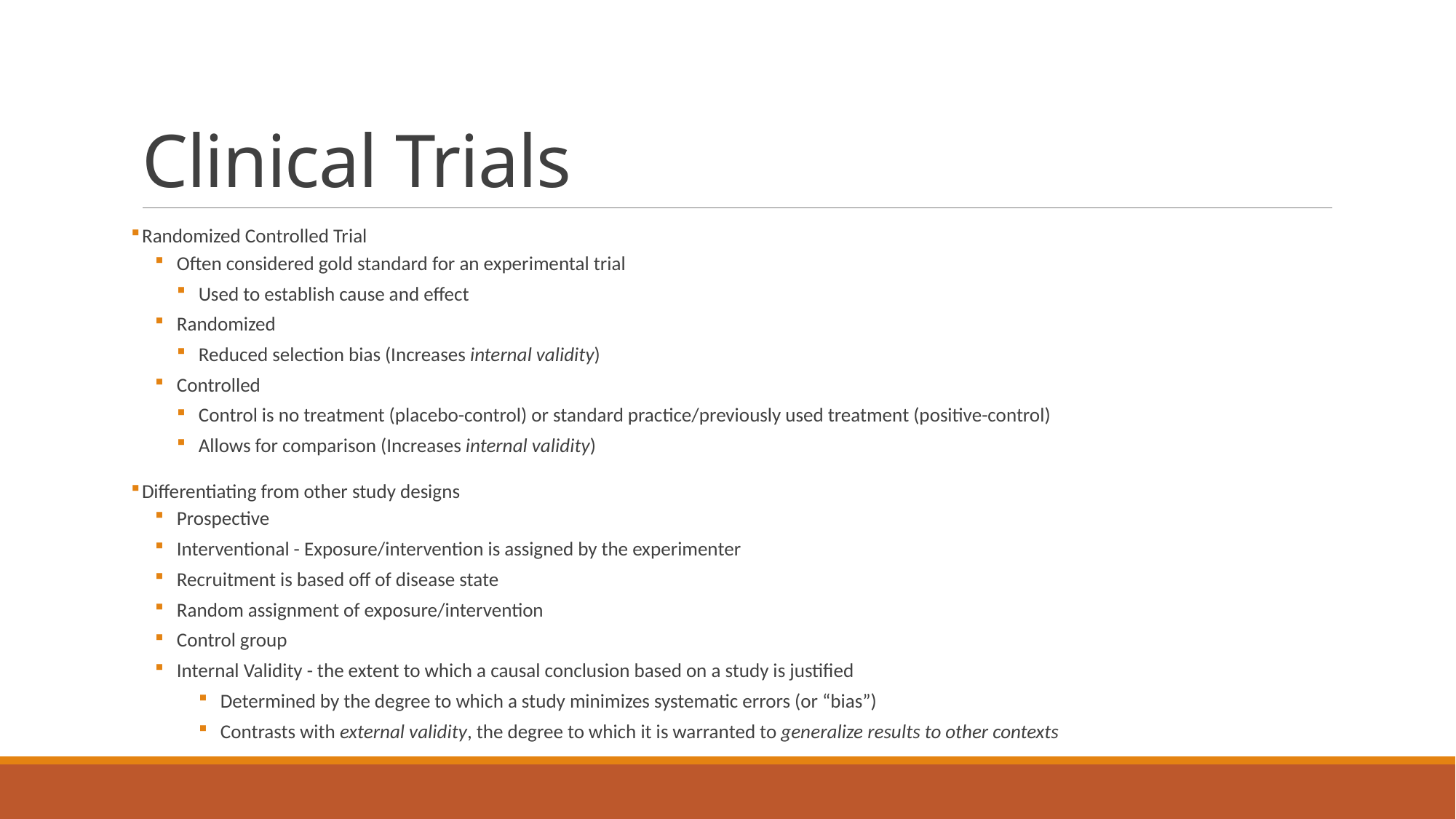

# Clinical Trials
Randomized Controlled Trial
Often considered gold standard for an experimental trial
Used to establish cause and effect
Randomized
Reduced selection bias (Increases internal validity)
Controlled
Control is no treatment (placebo-control) or standard practice/previously used treatment (positive-control)
Allows for comparison (Increases internal validity)
Differentiating from other study designs
Prospective
Interventional - Exposure/intervention is assigned by the experimenter
Recruitment is based off of disease state
Random assignment of exposure/intervention
Control group
Internal Validity - the extent to which a causal conclusion based on a study is justified
Determined by the degree to which a study minimizes systematic errors (or “bias”)
Contrasts with external validity, the degree to which it is warranted to generalize results to other contexts

## Slide 21
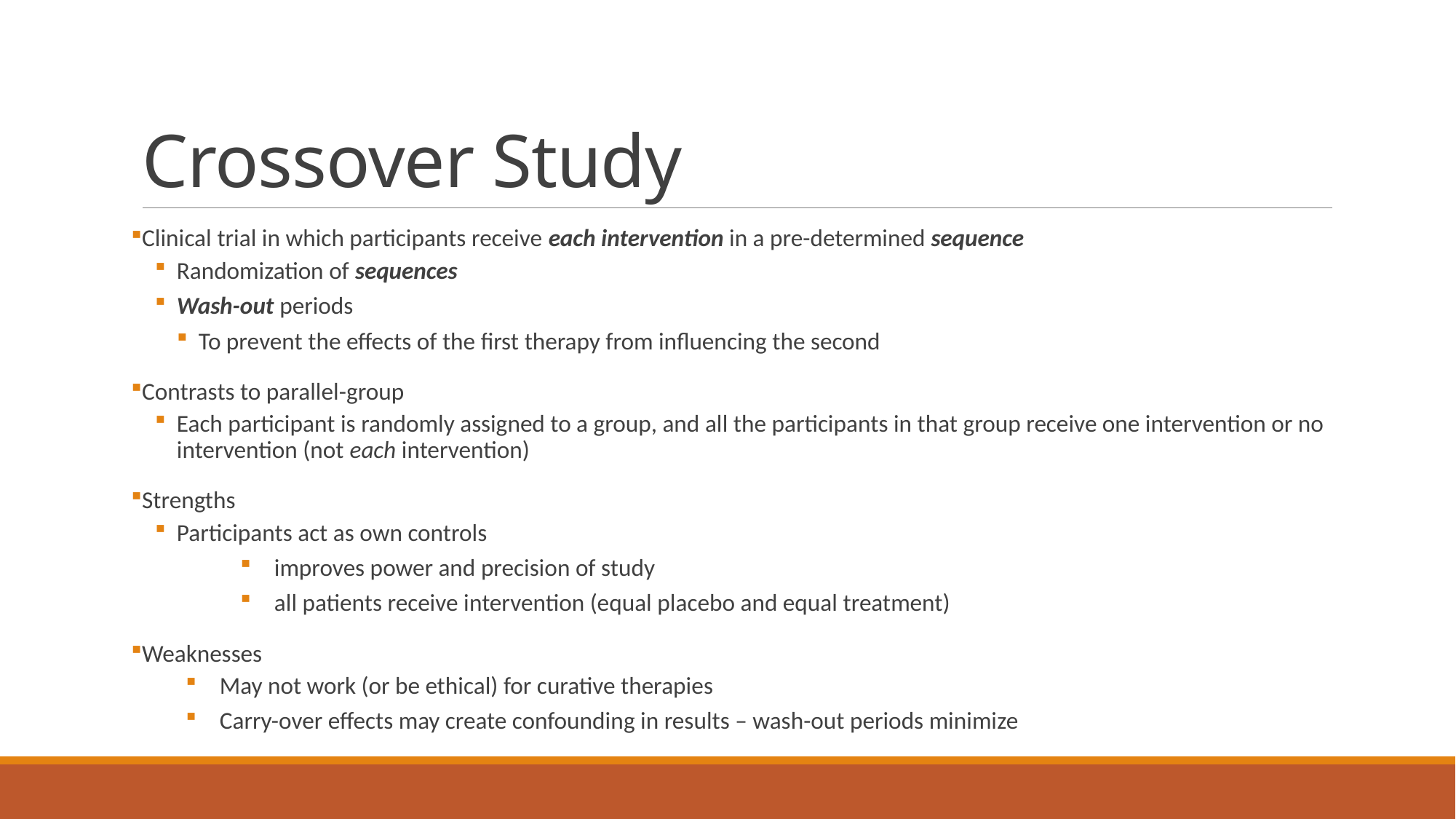

# Crossover Study
Clinical trial in which participants receive each intervention in a pre-determined sequence
Randomization of sequences
Wash-out periods
To prevent the effects of the first therapy from influencing the second
Contrasts to parallel-group
Each participant is randomly assigned to a group, and all the participants in that group receive one intervention or no intervention (not each intervention)
Strengths
Participants act as own controls
improves power and precision of study
all patients receive intervention (equal placebo and equal treatment)
Weaknesses
May not work (or be ethical) for curative therapies
Carry-over effects may create confounding in results – wash-out periods minimize

## Slide 22
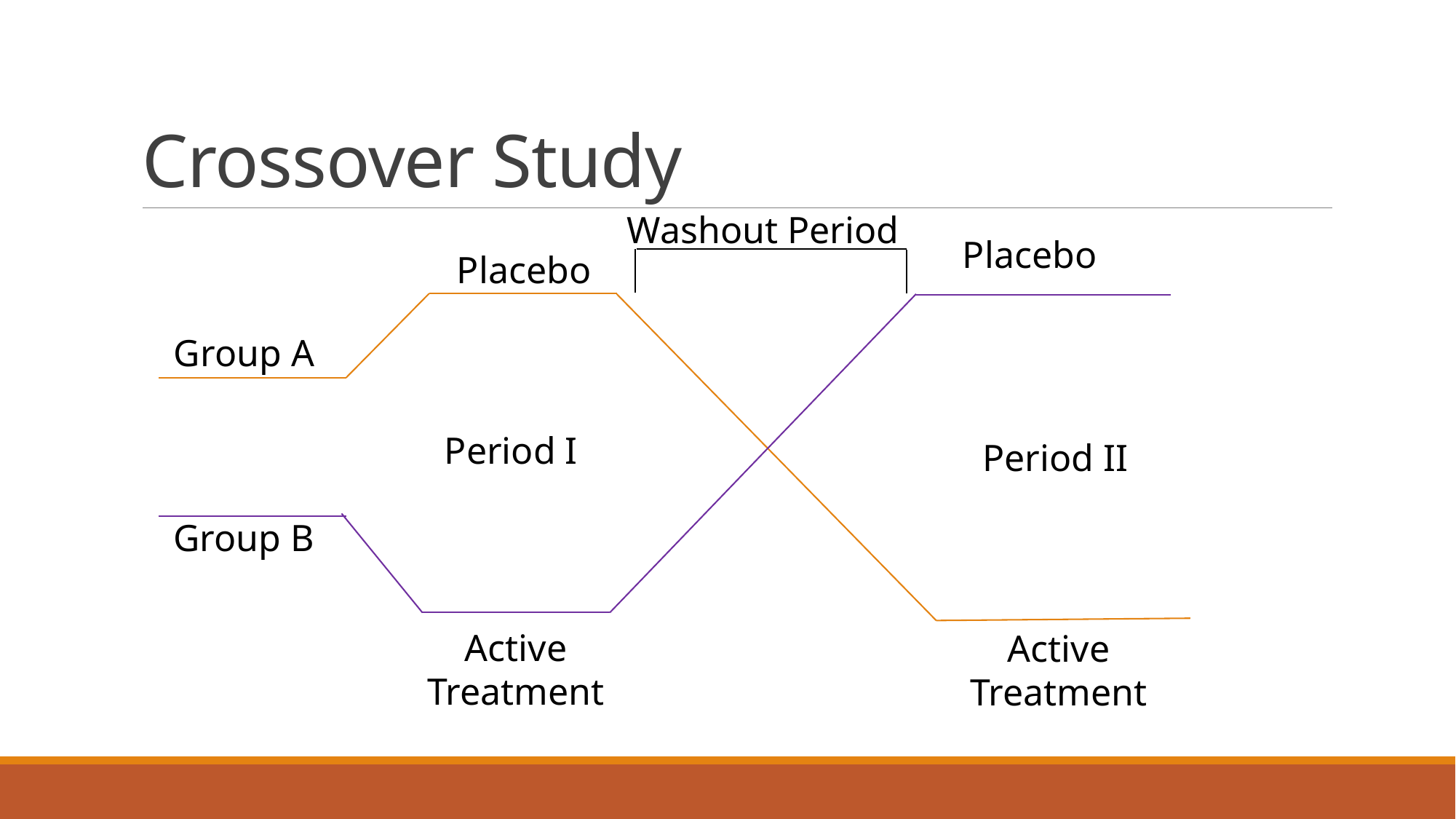

# Crossover Study
Washout Period
Placebo
Placebo
Group A
Period I
Period II
Group B
Active Treatment
Active Treatment

## Slide 23
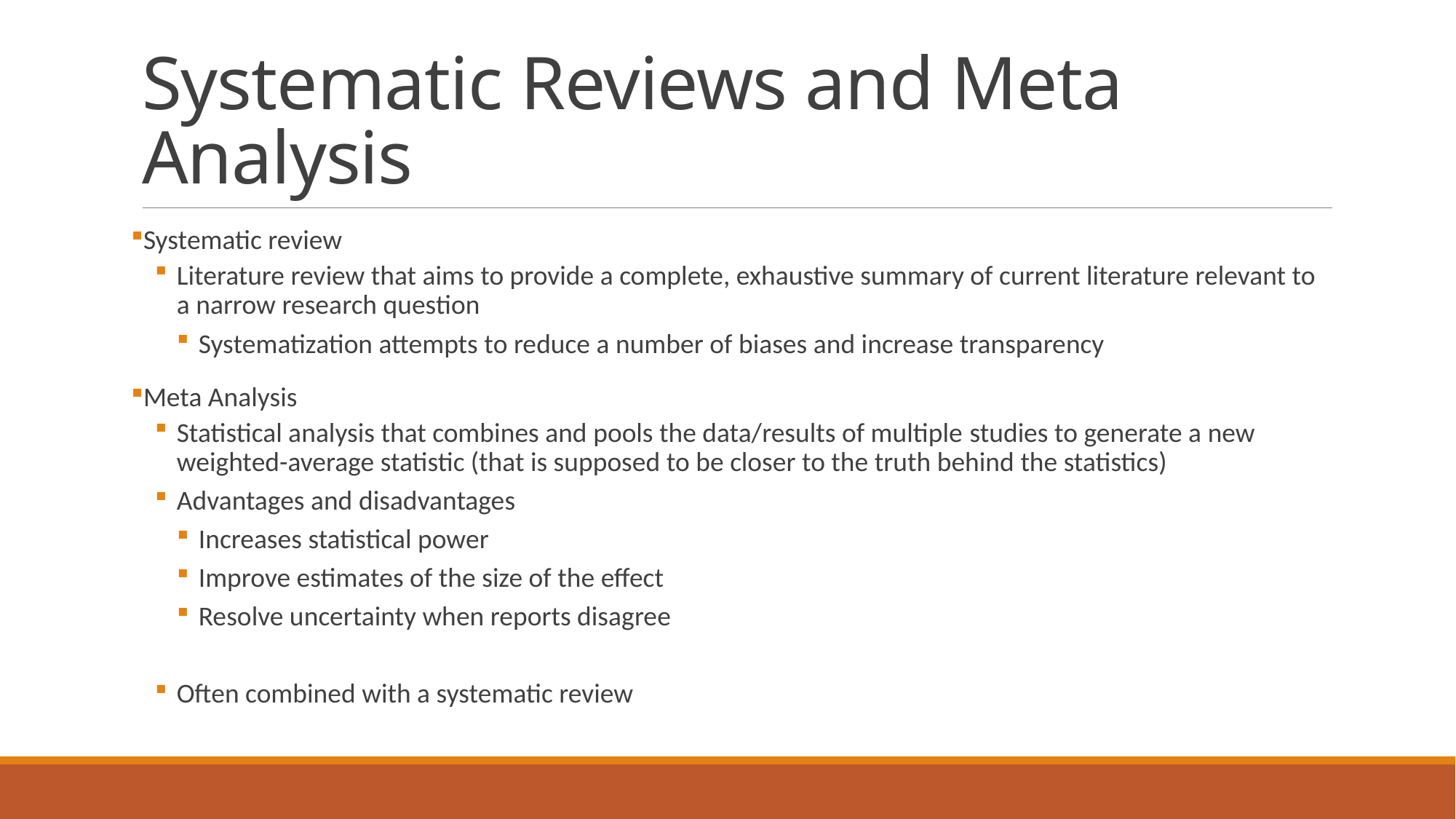

# Systematic Reviews and Meta Analysis
Systematic review
Literature review that aims to provide a complete, exhaustive summary of current literature relevant to a narrow research question
Systematization attempts to reduce a number of biases and increase transparency
Meta Analysis
Statistical analysis that combines and pools the data/results of multiple studies to generate a new weighted-average statistic (that is supposed to be closer to the truth behind the statistics)
Advantages and disadvantages
Increases statistical power
Improve estimates of the size of the effect
Resolve uncertainty when reports disagree
Often combined with a systematic review

## Slide 24
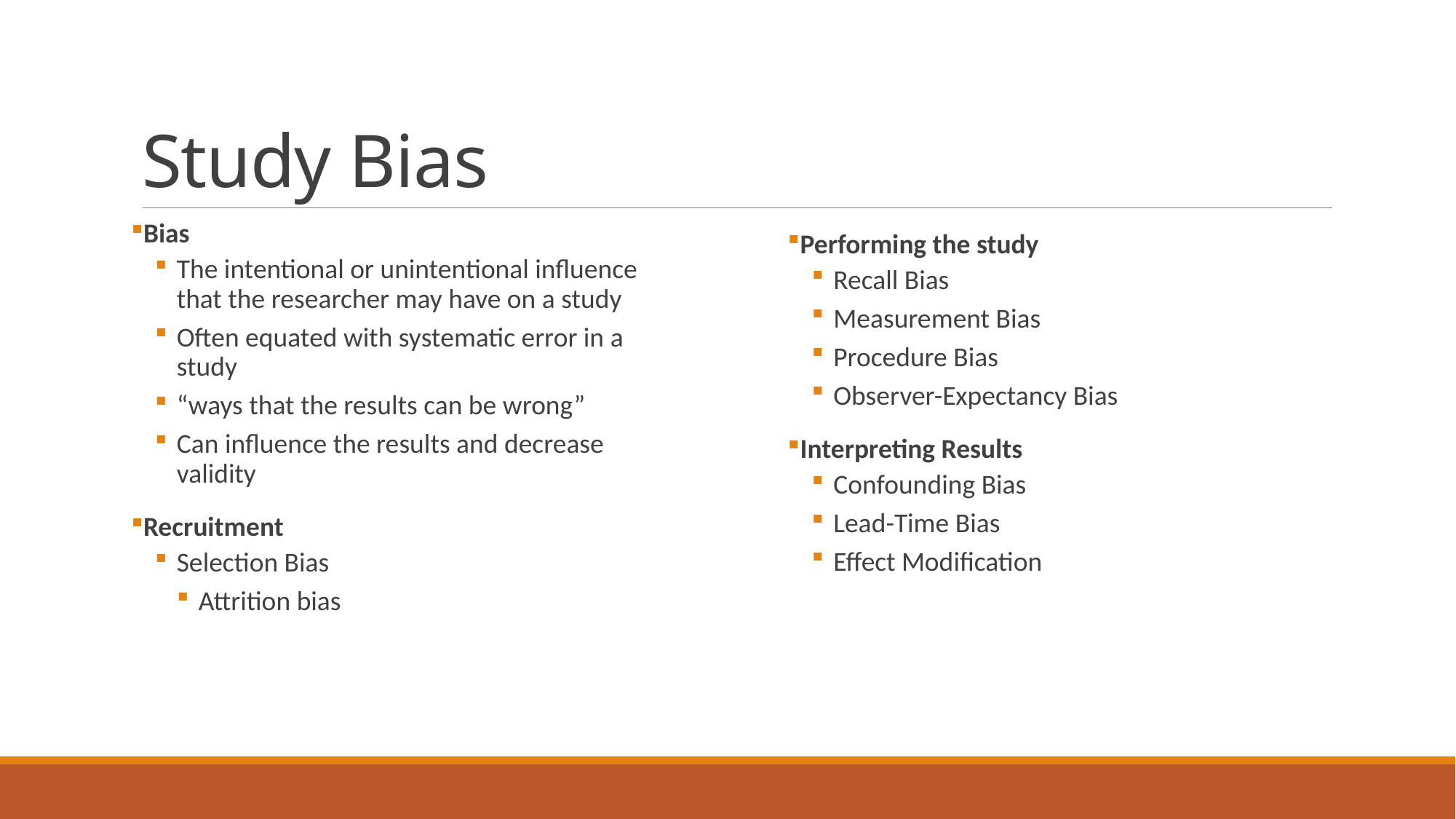

# Study Bias
Bias
The intentional or unintentional influence that the researcher may have on a study
Often equated with systematic error in a study
“ways that the results can be wrong”
Can influence the results and decrease validity
Recruitment
Selection Bias
Attrition bias
Performing the study
Recall Bias
Measurement Bias
Procedure Bias
Observer-Expectancy Bias
Interpreting Results
Confounding Bias
Lead-Time Bias
Effect Modification

## Slide 25
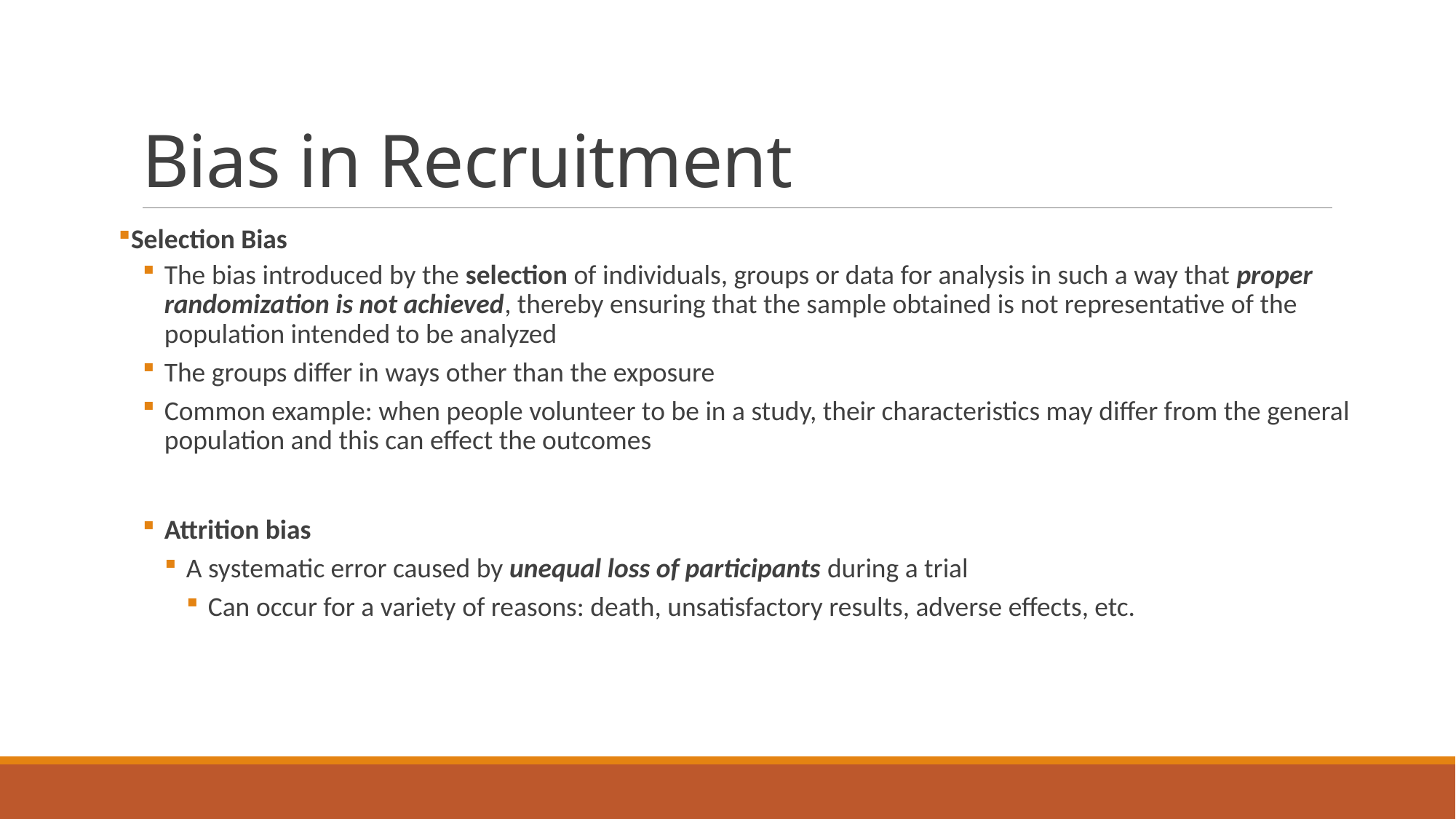

# Bias in Recruitment
Selection Bias
The bias introduced by the selection of individuals, groups or data for analysis in such a way that proper randomization is not achieved, thereby ensuring that the sample obtained is not representative of the population intended to be analyzed
The groups differ in ways other than the exposure
Common example: when people volunteer to be in a study, their characteristics may differ from the general population and this can effect the outcomes
Attrition bias
A systematic error caused by unequal loss of participants during a trial
Can occur for a variety of reasons: death, unsatisfactory results, adverse effects, etc.

## Slide 26
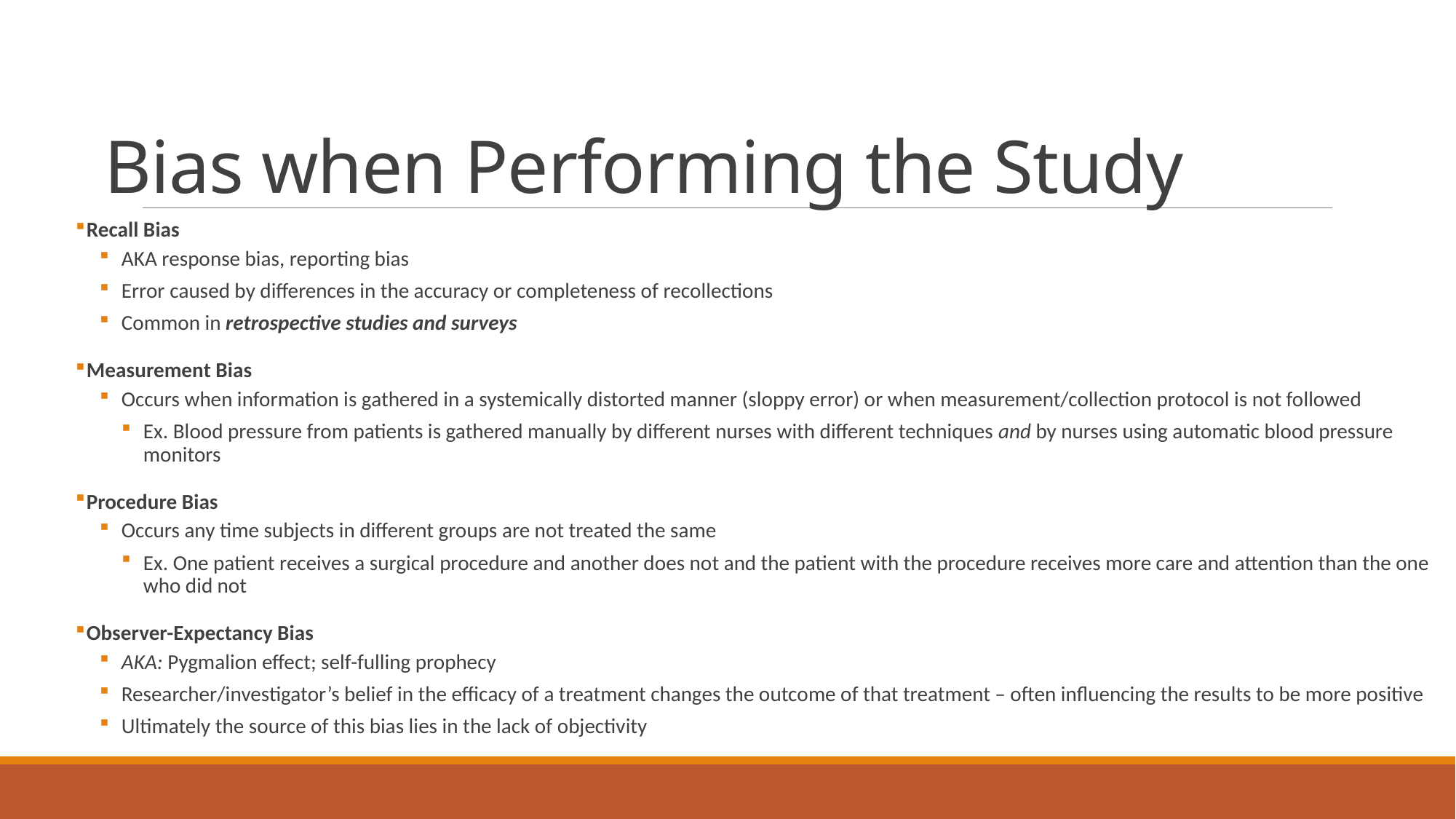

# Bias when Performing the Study
Recall Bias
AKA response bias, reporting bias
Error caused by differences in the accuracy or completeness of recollections
Common in retrospective studies and surveys
Measurement Bias
Occurs when information is gathered in a systemically distorted manner (sloppy error) or when measurement/collection protocol is not followed
Ex. Blood pressure from patients is gathered manually by different nurses with different techniques and by nurses using automatic blood pressure monitors
Procedure Bias
Occurs any time subjects in different groups are not treated the same
Ex. One patient receives a surgical procedure and another does not and the patient with the procedure receives more care and attention than the one who did not
Observer-Expectancy Bias
AKA: Pygmalion effect; self-fulling prophecy
Researcher/investigator’s belief in the efficacy of a treatment changes the outcome of that treatment – often influencing the results to be more positive
Ultimately the source of this bias lies in the lack of objectivity

## Slide 27
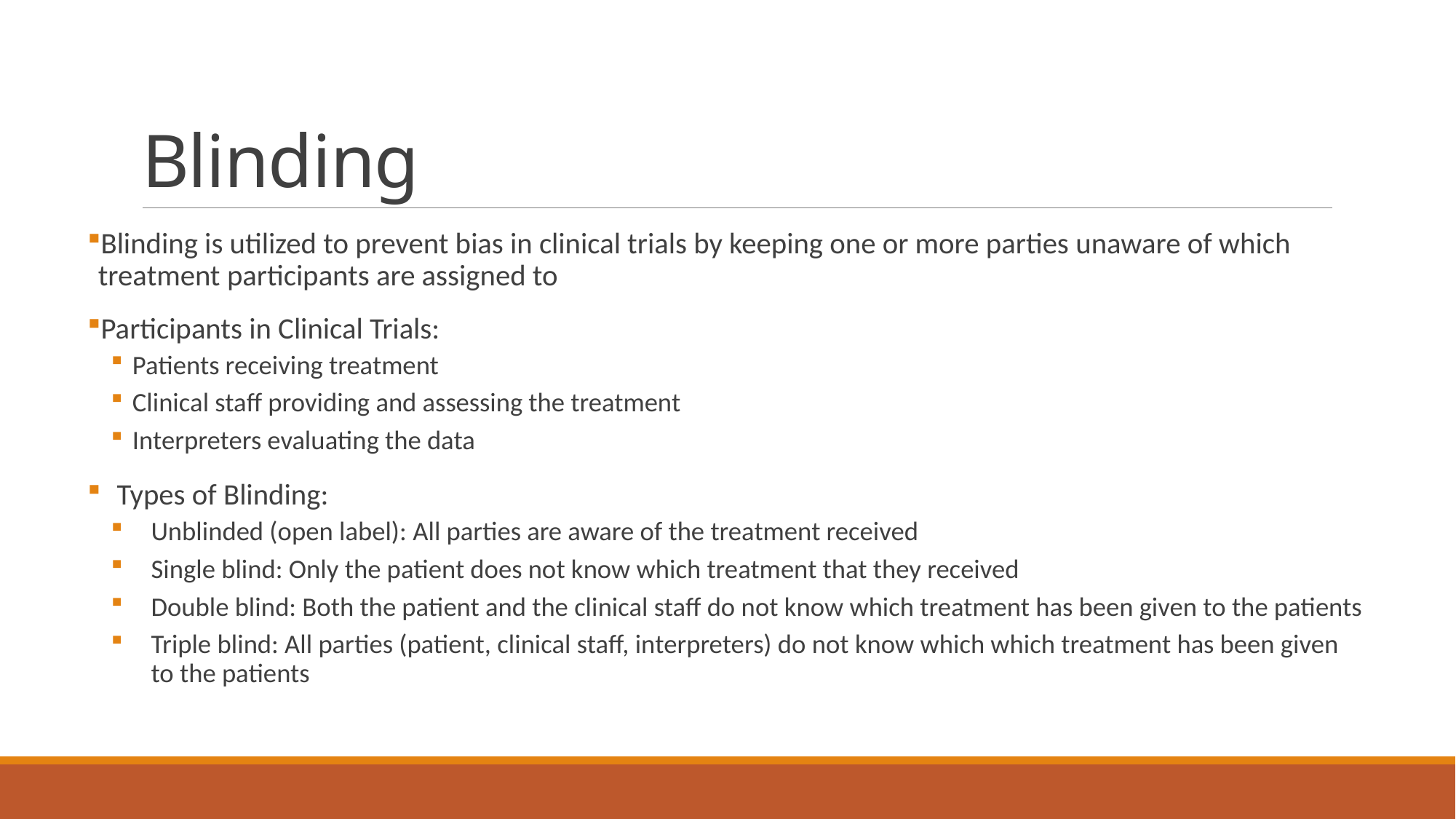

# Blinding
Blinding is utilized to prevent bias in clinical trials by keeping one or more parties unaware of which treatment participants are assigned to
Participants in Clinical Trials:
Patients receiving treatment
Clinical staff providing and assessing the treatment
Interpreters evaluating the data
Types of Blinding:
Unblinded (open label): All parties are aware of the treatment received
Single blind: Only the patient does not know which treatment that they received
Double blind: Both the patient and the clinical staff do not know which treatment has been given to the patients
Triple blind: All parties (patient, clinical staff, interpreters) do not know which which treatment has been given to the patients

## Slide 28
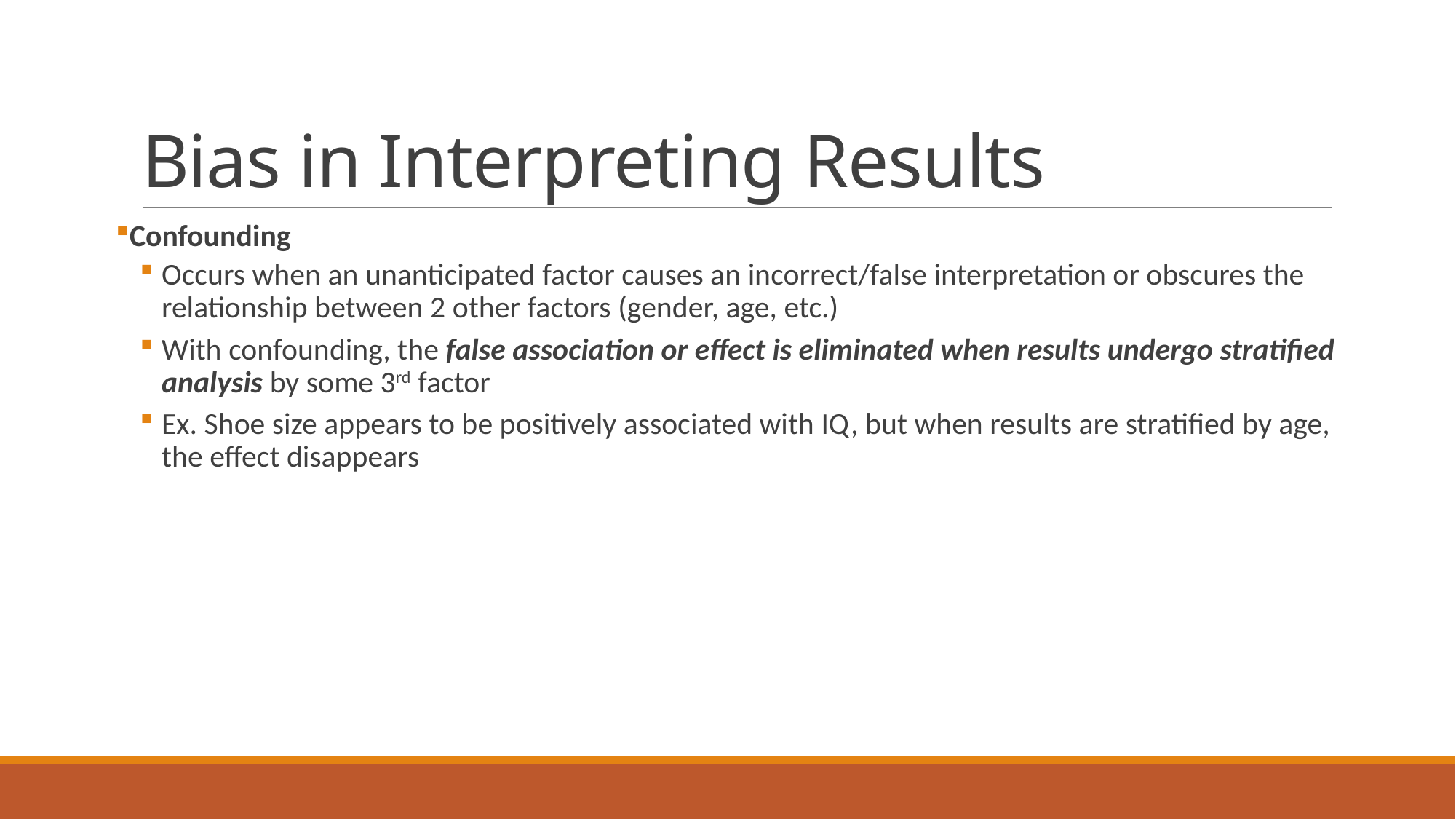

# Bias in Interpreting Results
Confounding
Occurs when an unanticipated factor causes an incorrect/false interpretation or obscures the relationship between 2 other factors (gender, age, etc.)
With confounding, the false association or effect is eliminated when results undergo stratified analysis by some 3rd factor
Ex. Shoe size appears to be positively associated with IQ, but when results are stratified by age, the effect disappears

## Slide 29
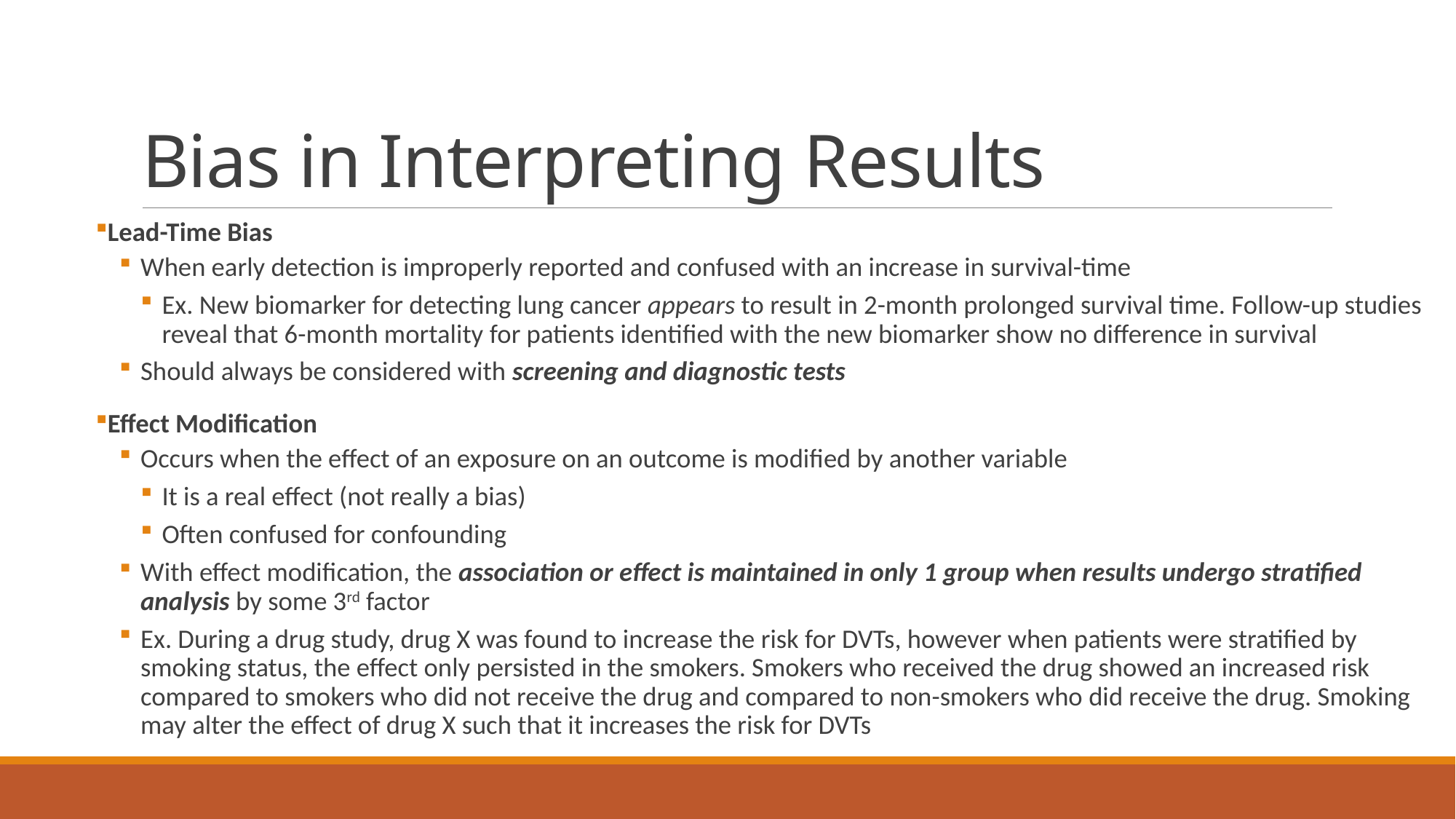

# Bias in Interpreting Results
Lead-Time Bias
When early detection is improperly reported and confused with an increase in survival-time
Ex. New biomarker for detecting lung cancer appears to result in 2-month prolonged survival time. Follow-up studies reveal that 6-month mortality for patients identified with the new biomarker show no difference in survival
Should always be considered with screening and diagnostic tests
Effect Modification
Occurs when the effect of an exposure on an outcome is modified by another variable
It is a real effect (not really a bias)
Often confused for confounding
With effect modification, the association or effect is maintained in only 1 group when results undergo stratified analysis by some 3rd factor
Ex. During a drug study, drug X was found to increase the risk for DVTs, however when patients were stratified by smoking status, the effect only persisted in the smokers. Smokers who received the drug showed an increased risk compared to smokers who did not receive the drug and compared to non-smokers who did receive the drug. Smoking may alter the effect of drug X such that it increases the risk for DVTs
