# Supplementary material for: Peer-Developed Modules on Basic Biostatistics and Evidence-Based Medicine Principles for Undergraduate Medical Education
Source: MedEdPORTAL. 2020 Nov 24;16:11026. doi: 10.15766/mep_2374-8265.11026 (PMC7703476; doi:10.15766/mep_2374-8265.11026)
Supplement: Supplementary file 1 — Module 1 Study Design and Bias.pptxModule 1 Problem Set.docxModule 1 Problem Set Answer Key.docxModule 1 Formative Quiz.docxModule 1 Formative Quiz Answer Key.docxModule 2 Interpreting Data from Clinical Trials.pptxModule 2 Problem Set.docxModule 2 Problem Set Answer Key.docxModule 2 Formative Quiz.docxModule 2 Formative Quiz Answer Key.docxModule 3 Diagnostic and Therapy Trial Results.pptxModule 3 Problem Set.docxModule 3 Problem Set Answer Key.docxModule 3 Formative Quiz.docxModule 3 Formative Quiz Answer Key.docxImplementation Guide.docxPostsession Evaluation Survey.docx [file mep_2374-8265.11026-s001.zip › B. Module 1 Problem Set.docx]

**Module 1 Problem Set**

Instructions: After reviewing Module 1, please work through the following problem set. Refer to the module if necessary. Discussion with peers is encouraged to exchange thought processes while explaining concepts fully in detail.

1a. A researcher conducts a study to evaluate obesity in a community. 3,500 patients are asked to complete a survey regarding their current health and habits. Using the data, it is found that 40% of respondents who responded "yes" to smoking status have COPD while 20% of respondents who responded "no" to smoking status have COPD. What study design does this demonstrate?

1. Case-Control
2. Case Study
3. Cross-Sectional
4. Twin Concordance

1b. What conclusion might you draw from the study detailed above?

1. Smoking causes COPD
2. Smoking may be a risk factor associated with COPD
3. Smoking is protective against COPD
4. You cannot say anything about the association between smoking and COPD

1c. If the same survey were given to citizens from Australia, United States and Japan and the results were compared, what study design would this be?

1. Ecological Study
2. Cohort Study
3. Case-Control
4. Clinical Trial

2a. A new oral anticoagulant (Drug X) is being evaluated in clinical trials. Over a course of 5 years, Drug X and Warfarin are administered to 2 different groups of patients who require long-term anticoagulation. At the end, it is found that Drug X carries a 10% risk reduction in drug-induced skin necrosis compared to Warfarin. Which phase of clinical trials is Drug X currently in?

1. Phase I
2. Phase II
3. Phase III
4. Phase IV

2b. In processing the results, it is found that a disproportionate number of patients in the drug X trial did not follow up at the 5-year mark. What bias might contribute to this?

1. Lead-time bias
2. Observer bias
3. Random misclassification bias
4. Recall bias
5. Selection bias

3. Which of the following types of evidence is most reliable to base clinical decisions on?

1. Meta-Analysis
2. Case Series
3. Ecological Study
4. Cohort Study

4. Which study design allows participants to act as their own controls?

1. Twin Concordance Study
2. Adoption Study
3. Crossover Study
4. Cross-Sectional Study

5. Drug A is being evaluated in clinical trials. During administration to a group of healthy subjects, it is determined that it has substantial first-pass metabolism, and poor oral bioavailability. Which phase of clinical trials did this data come from?

1. Phase I
2. Phase II
3. Phase III
4. Phase IV

6a. You are a researcher who wants to evaluate the effect of coffee consumption on hypertension in your population. You separate a group of 5,000 people into two groups, one that regularly consumes coffee and one that does not and over the next 10 years record the incidence of hypertension in each group. After 10 years of follow up, the investigators find a relative risk of 1.81 (CI 1.53-2.07) with a p-value of 0.03. What study design is this?

1. Case-Control
2. Cohort Study
3. Ecological Study
4. Cross-Sectional

6b. In analyzing the results, the investigators were worried that smoking may have an effect on the outcome of the study. They are stratifying the subjects into smokers and nonsmokers to see if the association between coffee and hypertension still exists.

RR p-value

Smokers 1.04 .092

Non-smokers 0.98 .086

What explains the difference in results when stratifying?

1. Confounding
2. Effect Modification
3. Measurement Bias
4. Meta-analysis

6c. Based on the results of stratifying for smoking, the investigators want to test for other potential misinterpretations. They postulate that gene X may affect the association between coffee and hypertension. As a result, they stratify the results based on subjects who were identified to be a carrier of gene X and subjects who were identified to not have gene X.

RR p-value

Gene X 1.78 .04

No Gene X 1.02 .073

What explains the difference in results when stratifying?

1. Confounding
2. Effect Modification
3. Measurement Bias
4. Meta-analysis

7a. You identify 3,678 new mothers with postpartum depression (Group A) and 2,212 new mothers without (Group B). In the pediatrician’s office you conduct a survey with new mothers to assess the relationship between Reagent X and postpartum depression. You discover that 40% of Group A was exposed to newly discovered Reagent X during their pregnancy while only 20% of Group B was exposed to it. What is the study design?

1. Case-Control
2. Cohort
3. Ecological
4. Case-Series

7b. What kind of bias may be affecting this study?

1. Allocation bias
2. Detection bias
3. Recall bias
4. Referral bias
5. Selection bias
