# Supplementary material for: Peer-Developed Modules on Basic Biostatistics and Evidence-Based Medicine Principles for Undergraduate Medical Education
Source: MedEdPORTAL. 2020 Nov 24;16:11026. doi: 10.15766/mep_2374-8265.11026 (PMC7703476; doi:10.15766/mep_2374-8265.11026)
Supplement: Supplementary file 1 — Module 1 Study Design and Bias.pptxModule 1 Problem Set.docxModule 1 Problem Set Answer Key.docxModule 1 Formative Quiz.docxModule 1 Formative Quiz Answer Key.docxModule 2 Interpreting Data from Clinical Trials.pptxModule 2 Problem Set.docxModule 2 Problem Set Answer Key.docxModule 2 Formative Quiz.docxModule 2 Formative Quiz Answer Key.docxModule 3 Diagnostic and Therapy Trial Results.pptxModule 3 Problem Set.docxModule 3 Problem Set Answer Key.docxModule 3 Formative Quiz.docxModule 3 Formative Quiz Answer Key.docxImplementation Guide.docxPostsession Evaluation Survey.docx [file mep_2374-8265.11026-s001.zip › C. Module 1 Problem Set Answer Key.docx]

**Module 1 Problem Set Answer Key**

Instructions: Please review the following answers. For incorrect answers, please refer back to module and/or discuss with peers on why each answer was correct or incorrect.

1a. A researcher conducts a study to evaluate obesity in a community. 3,500 patients are asked to complete a survey regarding their current health and habits. Using the data it is found that 40% of respondents who responded "yes" to smoking status have COPD while 20% of respondents who responded "no" to smoking status have COPD. What study design does this demonstrate?

1. Case-Control
2. Case Study
3. **Cross-Sectional**
4. Twin Concordance

*Cross-Sectional studies are a snapshot in time, usually done with a survey just to see what relationships might exist among individuals. Case Studies are done on one person (here there are 3500), twin concordance studies uses twins to evaluate environmental vs. genetic factors and case-control studies are when you first identify a population based on disease (people with and without COPD) and then looking in the past to see if the patients with COPD have had greater smoking exposure than the people who don’t.*

1b. What conclusion might you draw from the study detailed above?

1. Smoking causes COPD
2. **Smoking may be a risk factor associated with COPD**
3. Smoking is protective against COPD
4. You cannot say anything about the association between smoking and COPD

*Cross-sectional studies can say nothing about causes of disease but they can show that something is associated with disease; in this case a cross-sectional study involving smoking and COPD can only show that the two have an association but you cannot really say that smoking caused it since this is not a relationship that you observed for yourself.*

1c. If the same survey were given to citizens from Australia, United States and Japan and the results were compared, what study design would this be?

1. **Ecological Study**
2. Cohort Study
3. Case-Control
4. Clinical Trial

*Ecological studies are utilized to collect data related to different populations or countries to evaluate the differences in population prevalence. Clinical trials evaluate a new drug or intervention; case-control studies involve finding people with a disease and looking to see if they have had exposure while cohort studies find people based on exposure and see if they develop the disease.*

2a. A new oral anticoagulant (Drug X) is being evaluated in clinical trials. Over a course of 5 years, Drug X and Warfarin are administered to 2 different groups of patients who require long-term anticoagulation. At the end, it is found that Drug X carries a 10% risk reduction in drug-induced skin necrosis compared to Warfarin. Which phase of clinical trials is Drug X currently in?

1. Phase I
2. Phase II
3. **Phase III**
4. Phase IV

*Phase III is when you evaluate a new drug or intervention and compare it to existing therapy (in this case, Warfarin). Phase I is when you give the drug to a small group of people without the disease (unless it’s a cancer drug, which are known to be particularly toxic) to evaluate a drug’s safety, pharmacokinetics, pharmacodynamics. Phase II is when you give the drug to a small group of people with the disease and detect the its efficacy, optimal dosing, adverse effects. Phase IV is after the drug has been released to the market and is being monitored for long-term major adverse effects.*

2b. In processing the results, it is found that a disproportionate number of patients in the drug X trial did not follow up at the 5-year mark. What bias might contribute to this?

1. Lead-time bias
2. Observer bias
3. Random misclassification bias
4. Recall bias
5. **Selection bias**

*This question specifically* ***discusses attrition bias. Attrition bias is a form of selection bias that can occur when the subjects lost differ in their risk of developing the outcome compared to the remaining subjects.*** *In this case, because a disproportionate number of people from the drug X trial did not follow up compared to the Warfarin group there may be a effect on the results of the trial. Specifically, if the patients taking Drug X that had an adverse reaction chose not to follow up, this could result in finding a significant difference in skin necrosis where one doesn’t exist. If the loss were random proportional between the groups, attrition bias would be less likely.*

3. Which of the following types of evidence is most reliable to base clinical decisions on?

1. **Meta-Analysis**
2. Case Series
3. Ecological Study
4. Cohort Study

*Meta-Analyses are the best to base clinical decisions on because they involve the pooling of multiple different studies and generate more power for the study. Cohort studies are when you follow a group with exposure over time to see if they develop disease (vs. a group without exposure); ecological studies use populations and a case series reports on a series of patients having the outcome of interest. It is a descriptive observational study and does not usually have a control group.*

4. Which study design allows participants to act as their own controls?

1. Twin Concordance Study
2. Adoption Study
3. **Crossover Study**
4. Cross-Sectional Study

*Crossover Studies allow an individual to be their own control for two different interventions (drug a and b) since you have a washout period to clear the first drug from their system before administering the second one. If you are comparing two drugs, Drug A and Drug B and give it to Group A and Group B respectively, and then have a period of time where you “washout” the drugs, allowing them to wear off and get out of their systems, and then switch it so that Drug A is given to Group B and Drug B is given to Group A, after the study you could then see how one particular person reacted to Drug A and B and compare the drugs without worrying too much about individual variation*

5. Drug A is being evaluated in clinical trials. During administration to a group of healthy subjects, it is determined that it has substantial first-pass metabolism, and poor oral bioavailability. Which phase of clinical trials did this data come from?

1. **Phase I**
2. Phase II
3. Phase III
4. Phase IV

*Phase I is when you evaluate pharmacokinetics and pharmacodynamics.*

6a. You are a researcher who wants to evaluate the effect of coffee consumption on hypertension in your population. You separate a group of 5,000 people into two groups, one that regularly consumes coffee and one that does not and over the next 10 years record the incidence of hypertension in each group. After 10 years of follow up, the investigators find a relative risk of 1.81 (CI 1.53-2.07) with a p-value of 0.03. What study design is this?

1. Case-Control
2. **Cohort Study**
3. Ecological Study
4. Cross-Sectional

*Cohort is when you identify participants based on exposure/risk (coffee). Case-Control is when you identify them based on disease. Ecological evaluates populations. Cross-Sectional is a snapshot in time; no following over time involved.*

6b. In analyzing the results, the investigators were worried that smoking may have an effect on the outcome of the study. They are stratifying the subjects into smokers and nonsmokers to see if the association between coffee and hypertension still exists.

RR p-value

Smokers 1.04 .092

Non-smokers 0.98 .086

What explains the difference in results when stratifying?

1. **Confounding**
2. Effect Modification
3. Measurement Bias
4. Meta-analysis

*Because* ***the relative risk decreases in both groups when stratifying the results****, smoking can be shown to have* ***confounded*** *the results of the study. Confounding occurs when there is an extraneous factor (in this case smoking) that effects both the exposure (coffee) and disease (hypertension). If when stratifying for smoking, the relationship between coffee and hypertension disappears or is equally effected the smoking can be shown to be a confounder.*

6c. Based on the results of stratifying for smoking, the investigators want to test for other potential misinterpretations. They postulate that gene X may affect the association between coffee and hypertension. As a result, they stratify the results based on subjects who were identified to be a carrier of gene X and subjects who were identified to not have gene X.

RR p-value

Gene X 1.78 .04

No Gene X 1.02 .073

What explains the difference in results when stratifying?

1. Confounding
2. **Effect Modification**
3. Measurement Bias
4. Meta-analysis

*In this example, Gene X is an* ***effect modification*** *as when the results are stratified* ***the increased risk between coffee and hypertension only exists in one group (those with Gene X)****. In this case, Gene X does not change the results of the study (there is still a significant association between coffee and hypertension in patients with Gene X). However, it further identifies that relationship by showing that coffee is not associated with hypertension among all subjects but only those who possess Gene X.* ***Effect modification is not a type of bias.***

*If Gene X were a confounder, it would remove or affect the significant association between coffee and hypertension in both groups.*

7a. You identify 3,678 new mothers with postpartum depression (Group A) and 2,212 new mothers without (Group B). In the pediatrician’s office you conduct a survey with new mothers to assess the relationship between Reagent X and postpartum depression. You discover that 40% of Group A was exposed to newly discovered Reagent X during their pregnancy while only 20% of Group B was exposed to it. What is the study design?

1. **Case-Control**
2. Cohort
3. Ecological
4. Case-Series

*Case-Control is when you identify based on disease; cohort is when you identify based on exposure; ecological evaluates populations; case-series evaluates multiple case studies.*

7b. What kind of bias may be affecting this study?

1. Allocation bias
2. Detection bias
3. **Recall bias**
4. Referral bias
5. Selection bias

***Recall bias*** *results from an inaccurate recall of past exposures.* ***It occurs most often in retrospective studies (such as case-control studies) where people who have had an adverse event are more likely to recall previous potential risk factors that people who have not experienced an adverse event****. In this case, mothers who experienced postpartum depression may be more likely to indicate being exposed to Reagent X then mothers who didn’t experience postpartum depression.*
