# Supplementary material for: Peer-Developed Modules on Basic Biostatistics and Evidence-Based Medicine Principles for Undergraduate Medical Education
Source: MedEdPORTAL. 2020 Nov 24;16:11026. doi: 10.15766/mep_2374-8265.11026 (PMC7703476; doi:10.15766/mep_2374-8265.11026)
Supplement: Supplementary file 1 — Module 1 Study Design and Bias.pptxModule 1 Problem Set.docxModule 1 Problem Set Answer Key.docxModule 1 Formative Quiz.docxModule 1 Formative Quiz Answer Key.docxModule 2 Interpreting Data from Clinical Trials.pptxModule 2 Problem Set.docxModule 2 Problem Set Answer Key.docxModule 2 Formative Quiz.docxModule 2 Formative Quiz Answer Key.docxModule 3 Diagnostic and Therapy Trial Results.pptxModule 3 Problem Set.docxModule 3 Problem Set Answer Key.docxModule 3 Formative Quiz.docxModule 3 Formative Quiz Answer Key.docxImplementation Guide.docxPostsession Evaluation Survey.docx [file mep_2374-8265.11026-s001.zip › E. Module 1 Formative Quiz Answer Key.docx]

**Module 1 Formative Quiz Answer Key**

Instructions: Please review the following answers and explanations. For each incorrect answer, please refer back to the module and/or discuss with peers to exchange thoughts.

1. A study is conducted to describe a novel infectious syndrome seen in 30 people on a Cruise Ship. Investigators record the signs, symptoms, timing on onset, duration of symptoms, current treatment, and discuss the possible causal organisms.  One conclusion from the study is that the researchers believe the novel infection is associated with the consumption of pork. Which of the following best describes this study design?

a) Case-control Study

b) Cohort Study

c) Crossover Study

**d) Case-Series Study**

e) Cross-Sectional Study

*This study is most appropriately labeled as a case-series study, which is a descriptive report on a group of patients (“30 people on a Cruise Ship”) receiving the same treatment or with the same disease (“novel infectious syndrome”). The report aims to follow the same group of patients over time to detail symptoms, signs, diagnosis, timing, treatment, possible etiologies, and other factors related to the novel treatment or disease.*

2. A group of researchers wants to determine if individuals who eat a high antioxidant diet are less likely to develop colon cancer. To achieve this, they recruit 1,000 individuals who currently eat a high antioxidant diet, as identified by certain study criteria, and they recruit 1,000 people who eat a more ordinary diet that is not high in antioxidants. The researchers follow both groups of people for 10 years, tracking whether or not they develop colon cancer. At the conclusion of this study, what is the outcome statistic the researchers will most likely use to analyze the results?

a) Odds Ratio

b) Pearson Correlation Coefficient

**c) Relative Risk**

d) Paired T-test

e) ANOVA

*Relative risk is the most appropriate risk quantifier because the study in question is a cohort study. Cohort studies are longitudinal observational studies in which individuals are grouped by exposure (“high antioxidant diet”) and followed over a period of time (“10 years”) to determine whether the exposure is related to the development of a specified outcome or disease (“colon cancer”).*

3. Investigators are studying a potential new blood pressure medication. 40 healthy participants are assigned to receive various doses of the drug. Adverse effects, the pharmacokinetics, and pharmacodynamics are assessed. Which step in drug development does this process represent?

a) Preclinical Trial

**b) Phase I**

c) Phase II

d) Phase III

e) Phase IV

*Phase I trials question whether the treatment is safe by utilizing a small number of healthy subjects as the sample group. During the treatment phase, researchers determine the safe dose range, toxicity, pharmacokinetics/pharmacodynamics, and side effects.*

4. A randomized controlled trial is designed to compare the use of beta blockers versus placebo-pill in heart failure patients. The primary outcome of interest was mortality, and the secondary outcome was exercise tolerance. A total of 2,000 patients with heart failure are enrolled in the study through random selection of clinic charts from a large health system. Beta blocker adherence, mortality rate, and 10-minute walk test are assessed throughout the study for a total of 2 years. In the end, 580 patients were lost to follow-up or were removed from the study due to non-adherence, with a majority of these patients coming from the beta blocker group. The relative risk mortality in patients on beta blockers was found to be 0.9 (95% CI = 0.7 – 1.3). This was concluded to be a non-significant reduction in mortality. Which of the following biases was most likely to have affected the results of this study?

a) Lead-time bias

b) Observer bias

c) Recall bias

**d) Selection bias**

e) Random misclassification bias

*Selection bias, defined as bias introduced by the selection of individuals, groups or data for analysis in such a way that proper randomization is not achieved, thereby ensuring that the sample obtained is not representative of the population intended to be analyzed, is present. In this example, the loss of 580 patients from the study, many of whom were a part of the beta blocker therapy group, leads to a misrepresentation of the population intended to undergo treatment, thereby affecting the results as a consequence of selection bias.*

5. A clinical trial conducted to evaluate the efficacy of a new drug to increase HDL cholesterol. 100 patients are enrolled and randomized to receive either the new drug or a placebo. Background characteristics (e.g., age, sex, educational level, income) are measured at baseline, and they are found to be comparable. Participants take the medication for 8 weeks and asked to return for follow-up. After running the analysis on the results, the researchers found that there was no significant difference in HDL levels between the two groups. This surprised the researchers and so they stratified the results for further answers. When stratified by sex, the researchers found a significant increase in HDL levels in male patients and no significant change in HDL levels in female patients. Which of the following best describes this phenomenon?

a) Confounding

**b) Effect modification**

c) Latent period

d) Observer bias

e) Selection bias

*Effect modification occurs when the effect of an exposure on an outcome is modified by another variable. With effect modification, the association or effect is maintained in only one group when results undergo stratified analysis by a third factor. In this example, the original study evaluates the efficacy of a drug on HDL cholesterol levels. Upon stratifying by sex, the researchers found an increase in the outcome (“HDL cholesterol levels”) in one group only (“male patients”), but no change in the other group (“female patients”).*

6. A study is conducted to determine the association between drinking 3 or more cups of coffee daily and the chance of developing skin cancer. Three groups of volunteer subjects are selected from a large healthcare system and they are followed for 5 years. The first group of 500 participants consists of day shift nurses who do not drink 3 cups of coffee daily. The second group of 500 participants consists of night shift nurses who drink > 3 cups of coffee daily. When the results of the study are finished, it is determined that coffee decreases the relative risk of skin cancer by 18% (P-value 0.03). Which is most likely the design of this study?

a) Case-control Study

b) Randomized Controlled Trial

**c) Cohort Study**

d) Ecological Study

e) Case Series

*A cohort study is a longitudinal observational study in which individuals are grouped by exposure (“3 or more cups of coffee”) and followed over a period of time (“5 years”) to determine whether the exposure is related to the development of a specified outcome or disease (“skin cancer”).*
