# Supplementary material for: Peer-Developed Modules on Basic Biostatistics and Evidence-Based Medicine Principles for Undergraduate Medical Education
Source: MedEdPORTAL. 2020 Nov 24;16:11026. doi: 10.15766/mep_2374-8265.11026 (PMC7703476; doi:10.15766/mep_2374-8265.11026)
Supplement: Supplementary file 1 — Module 1 Study Design and Bias.pptxModule 1 Problem Set.docxModule 1 Problem Set Answer Key.docxModule 1 Formative Quiz.docxModule 1 Formative Quiz Answer Key.docxModule 2 Interpreting Data from Clinical Trials.pptxModule 2 Problem Set.docxModule 2 Problem Set Answer Key.docxModule 2 Formative Quiz.docxModule 2 Formative Quiz Answer Key.docxModule 3 Diagnostic and Therapy Trial Results.pptxModule 3 Problem Set.docxModule 3 Problem Set Answer Key.docxModule 3 Formative Quiz.docxModule 3 Formative Quiz Answer Key.docxImplementation Guide.docxPostsession Evaluation Survey.docx [file mep_2374-8265.11026-s001.zip › F. Module 2 Interpreting Data from Clinical Trials.pptx]

## Slide 1
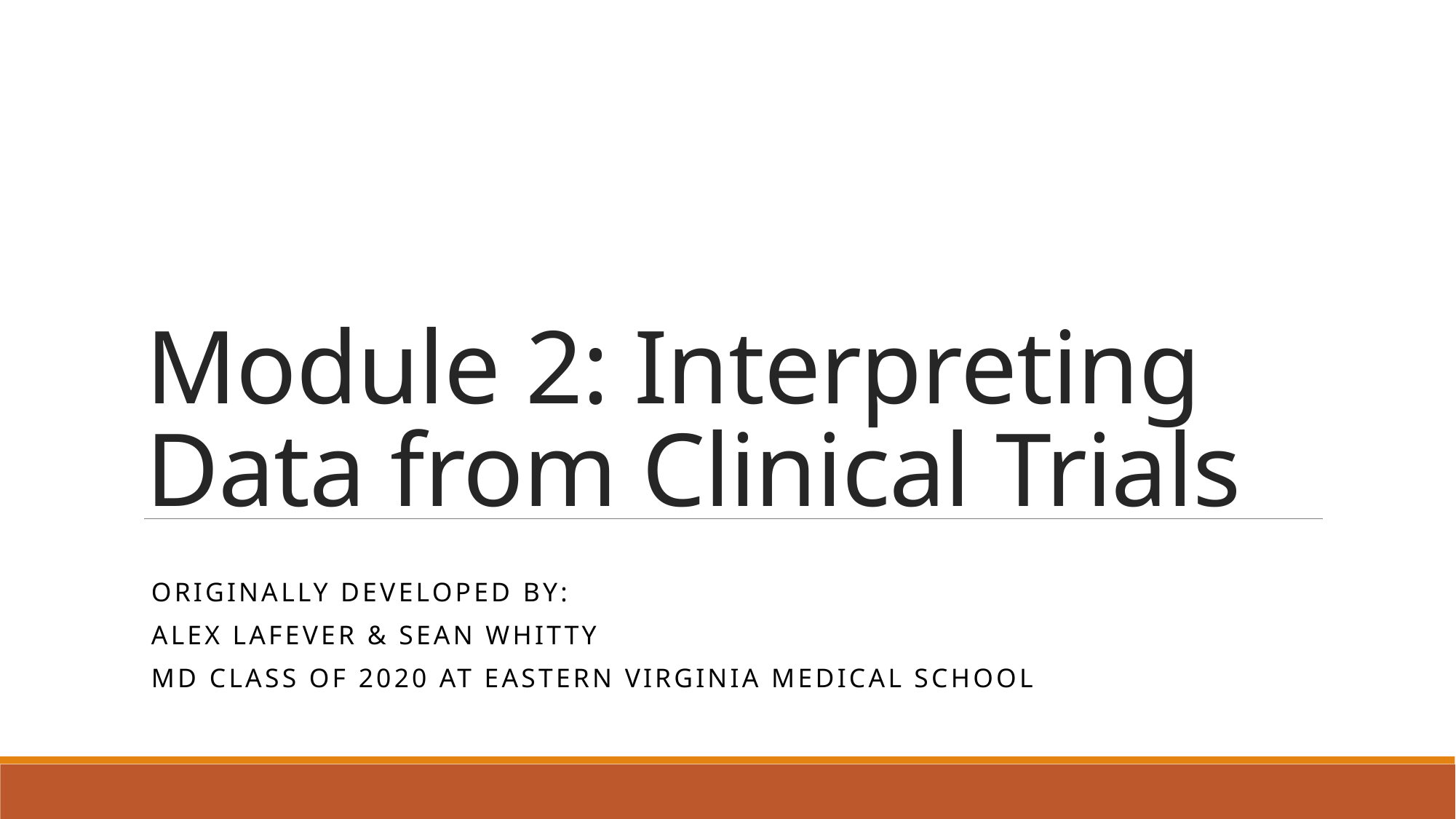

# Module 2: Interpreting Data from Clinical Trials
Originally Developed by:
Alex LaFever & Sean Whitty
MD Class of 2020 at Eastern Virginia Medical School

## Slide 2
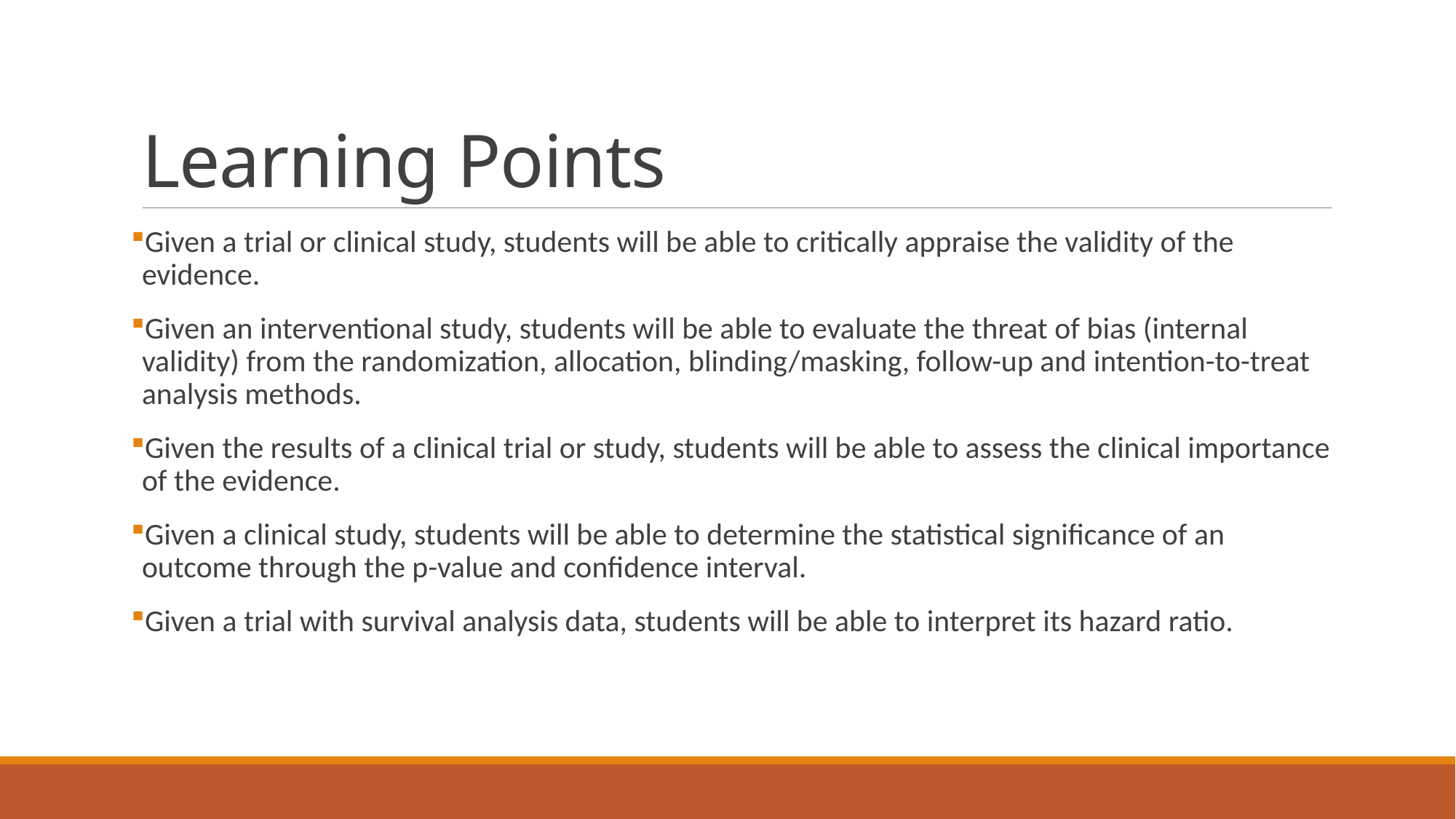

# Learning Points
Given a trial or clinical study, students will be able to critically appraise the validity of the evidence.
Given an interventional study, students will be able to evaluate the threat of bias (internal validity) from the randomization, allocation, blinding/masking, follow-up and intention-to-treat analysis methods.
Given the results of a clinical trial or study, students will be able to assess the clinical importance of the evidence.
Given a clinical study, students will be able to determine the statistical significance of an outcome through the p-value and confidence interval.
Given a trial with survival analysis data, students will be able to interpret its hazard ratio.

## Slide 3
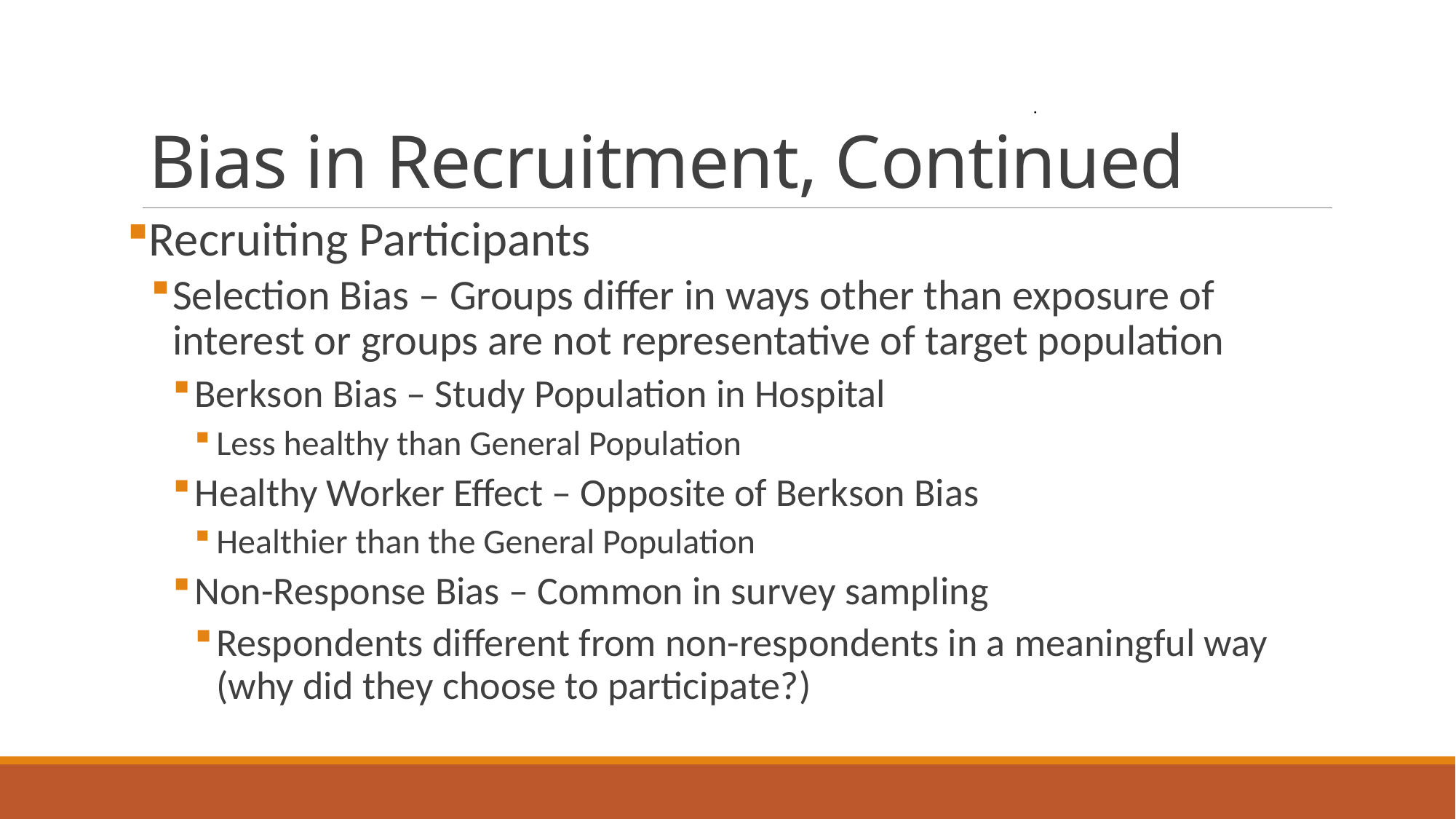

# Bias in Recruitment, Continued
Recruiting Participants
Selection Bias – Groups differ in ways other than exposure of interest or groups are not representative of target population
Berkson Bias – Study Population in Hospital
Less healthy than General Population
Healthy Worker Effect – Opposite of Berkson Bias
Healthier than the General Population
Non-Response Bias – Common in survey sampling
Respondents different from non-respondents in a meaningful way (why did they choose to participate?)

## Slide 4
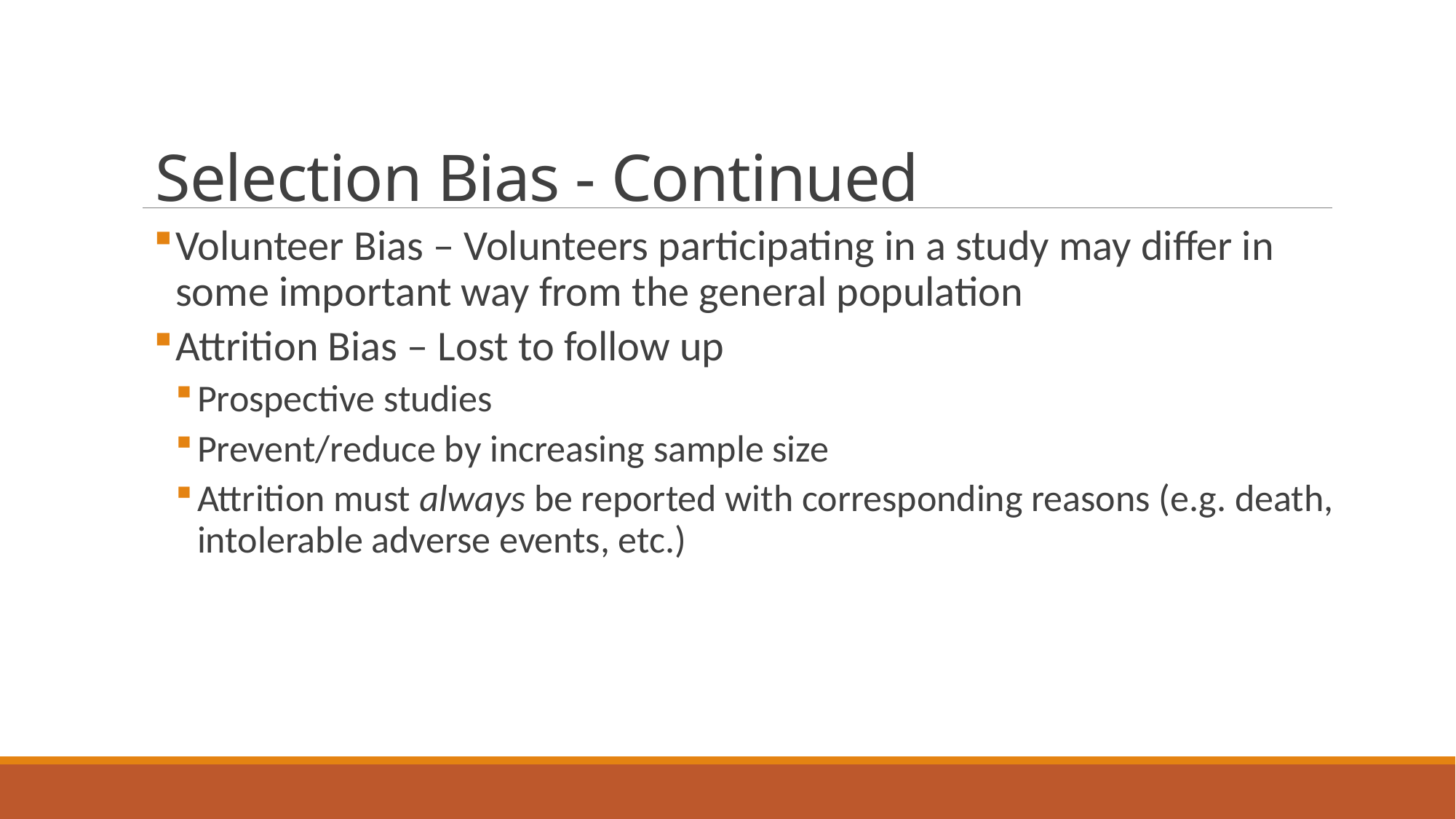

# Selection Bias - Continued
Volunteer Bias – Volunteers participating in a study may differ in some important way from the general population
Attrition Bias – Lost to follow up
Prospective studies
Prevent/reduce by increasing sample size
Attrition must always be reported with corresponding reasons (e.g. death, intolerable adverse events, etc.)

## Slide 5
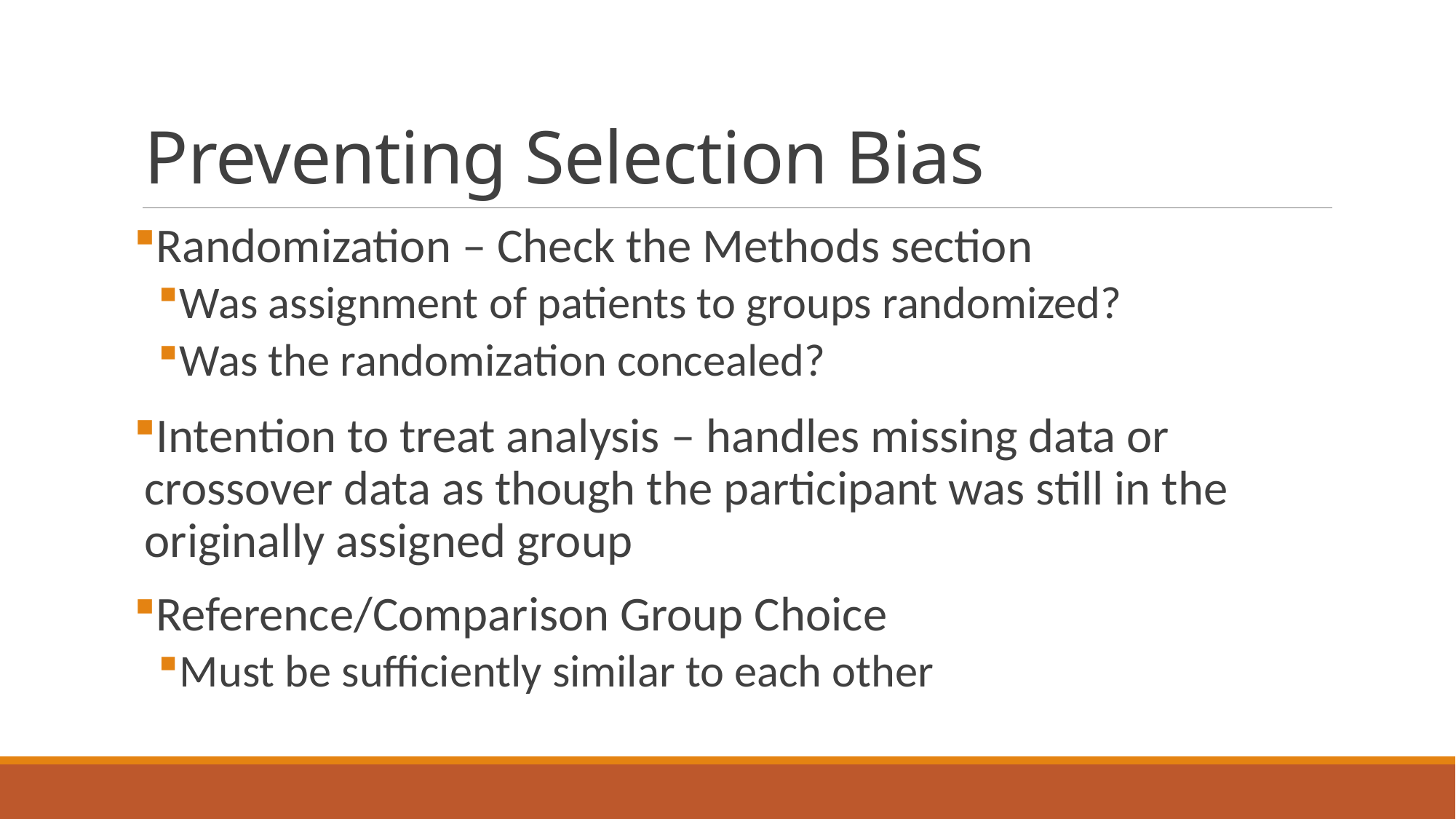

# Preventing Selection Bias
Randomization – Check the Methods section
Was assignment of patients to groups randomized?
Was the randomization concealed?
Intention to treat analysis – handles missing data or crossover data as though the participant was still in the originally assigned group
Reference/Comparison Group Choice
Must be sufficiently similar to each other

## Slide 6
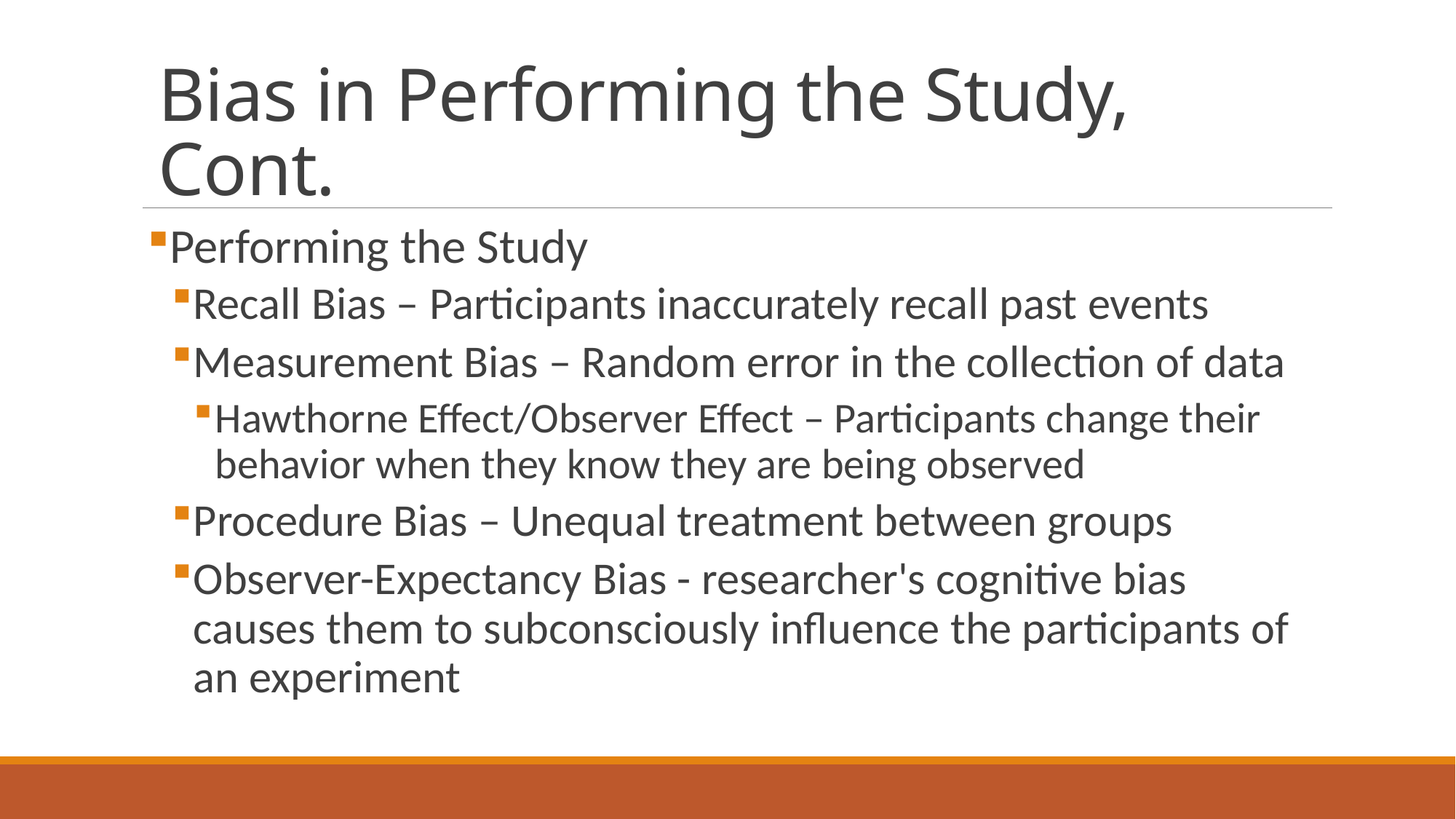

# Bias in Performing the Study, Cont.
Performing the Study
Recall Bias – Participants inaccurately recall past events
Measurement Bias – Random error in the collection of data
Hawthorne Effect/Observer Effect – Participants change their behavior when they know they are being observed
Procedure Bias – Unequal treatment between groups
Observer-Expectancy Bias - researcher's cognitive bias causes them to subconsciously influence the participants of an experiment

## Slide 7
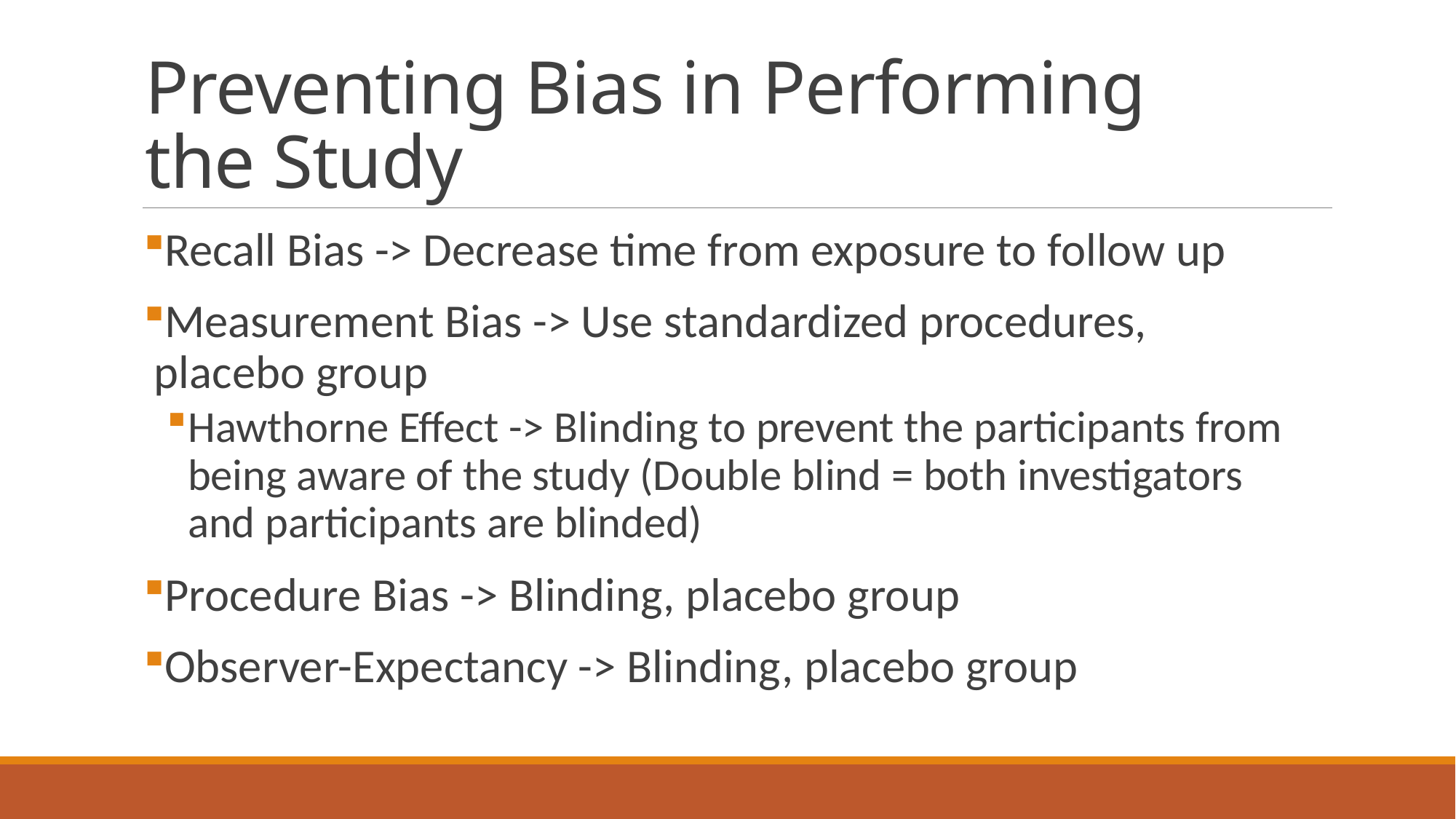

# Preventing Bias in Performing the Study
Recall Bias -> Decrease time from exposure to follow up
Measurement Bias -> Use standardized procedures, placebo group
Hawthorne Effect -> Blinding to prevent the participants from being aware of the study (Double blind = both investigators and participants are blinded)
Procedure Bias -> Blinding, placebo group
Observer-Expectancy -> Blinding, placebo group

## Slide 8
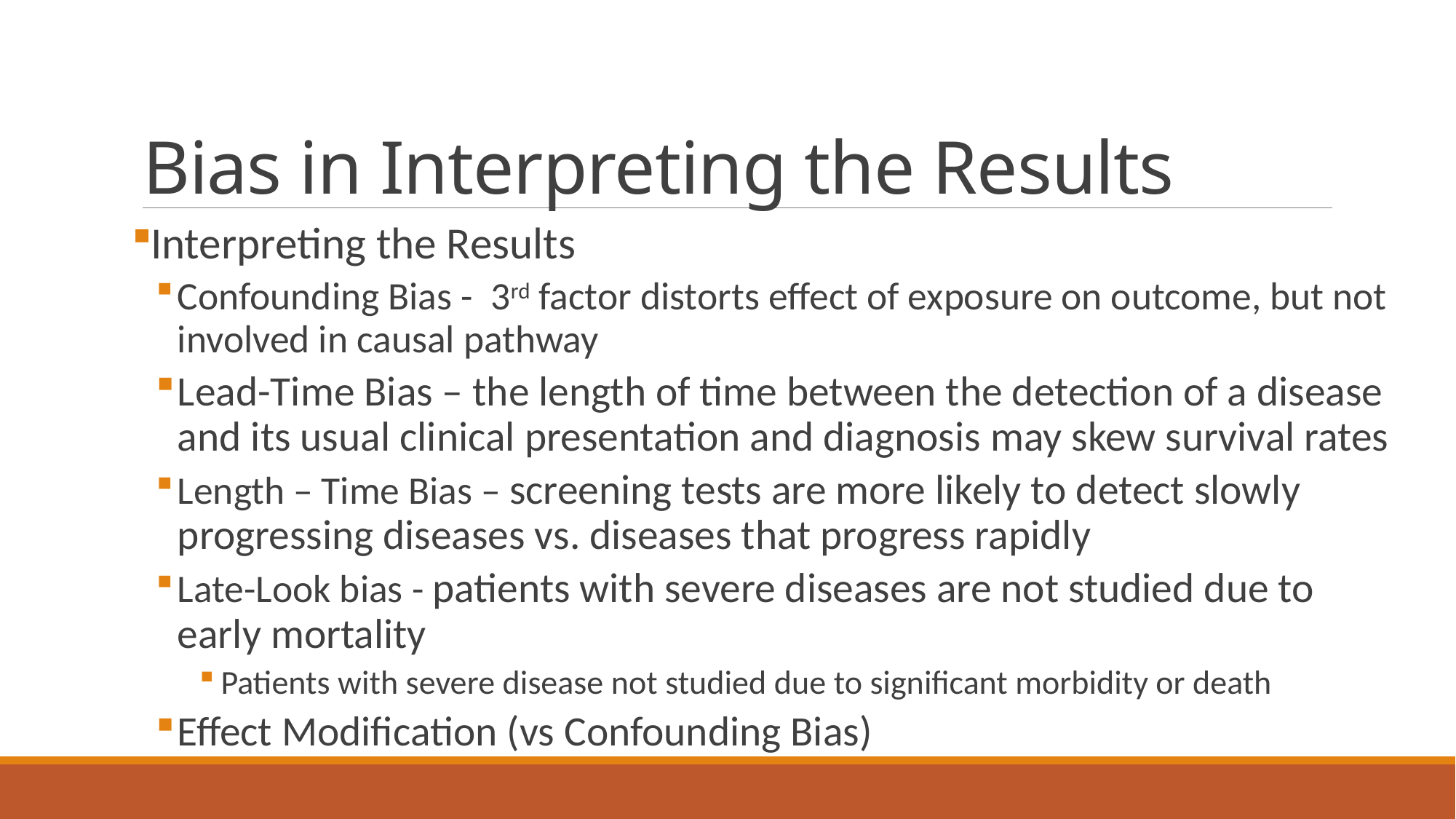

# Bias in Interpreting the Results
Interpreting the Results
Confounding Bias - 3rd factor distorts effect of exposure on outcome, but not involved in causal pathway
Lead-Time Bias – the length of time between the detection of a disease and its usual clinical presentation and diagnosis may skew survival rates
Length – Time Bias – screening tests are more likely to detect slowly progressing diseases vs. diseases that progress rapidly
Late-Look bias - patients with severe diseases are not studied due to early mortality
Patients with severe disease not studied due to significant morbidity or death
Effect Modification (vs Confounding Bias)

## Slide 9
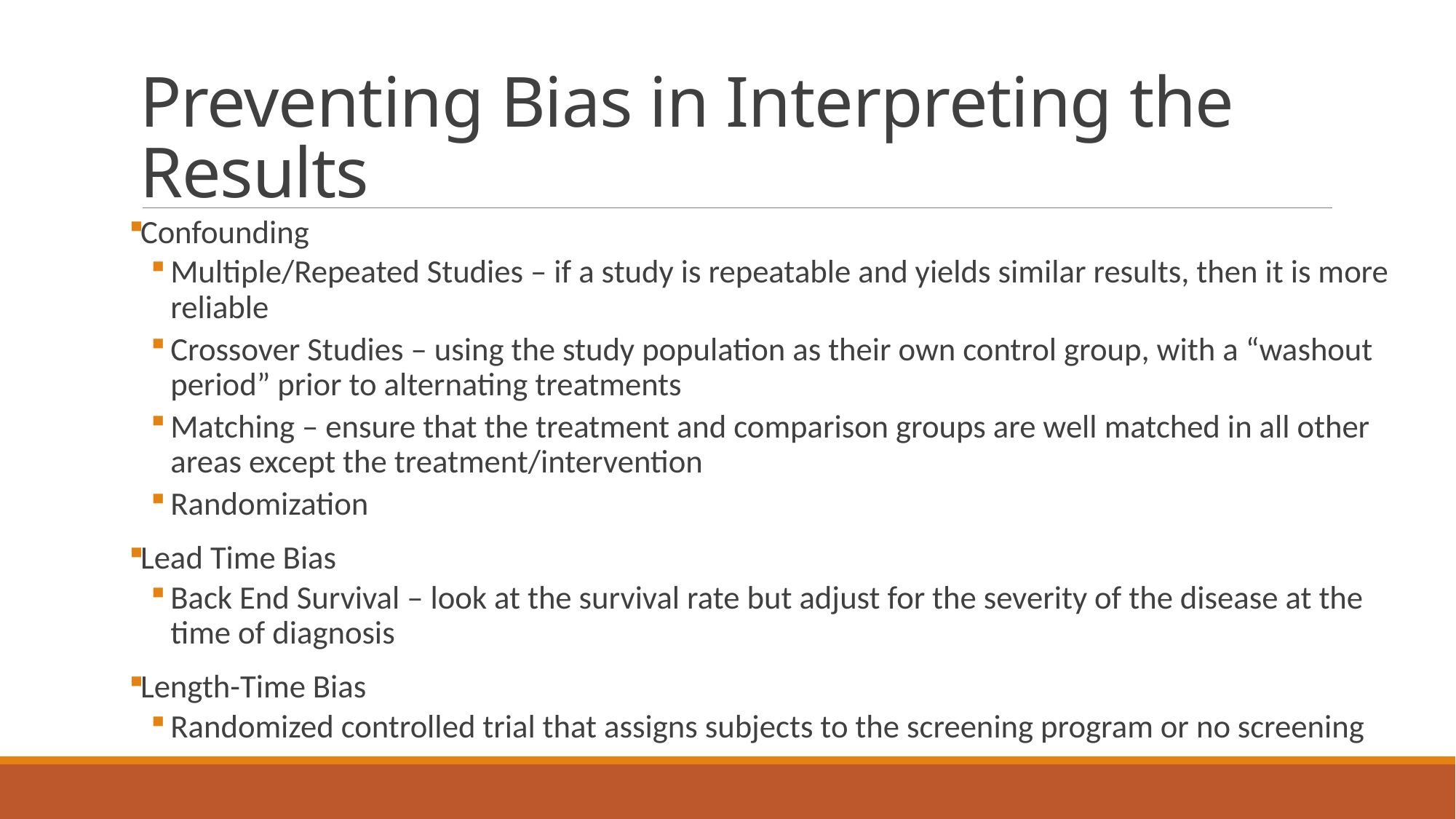

# Preventing Bias in Interpreting the Results
Confounding
Multiple/Repeated Studies – if a study is repeatable and yields similar results, then it is more reliable
Crossover Studies – using the study population as their own control group, with a “washout period” prior to alternating treatments
Matching – ensure that the treatment and comparison groups are well matched in all other areas except the treatment/intervention
Randomization
Lead Time Bias
Back End Survival – look at the survival rate but adjust for the severity of the disease at the time of diagnosis
Length-Time Bias
Randomized controlled trial that assigns subjects to the screening program or no screening

## Slide 10
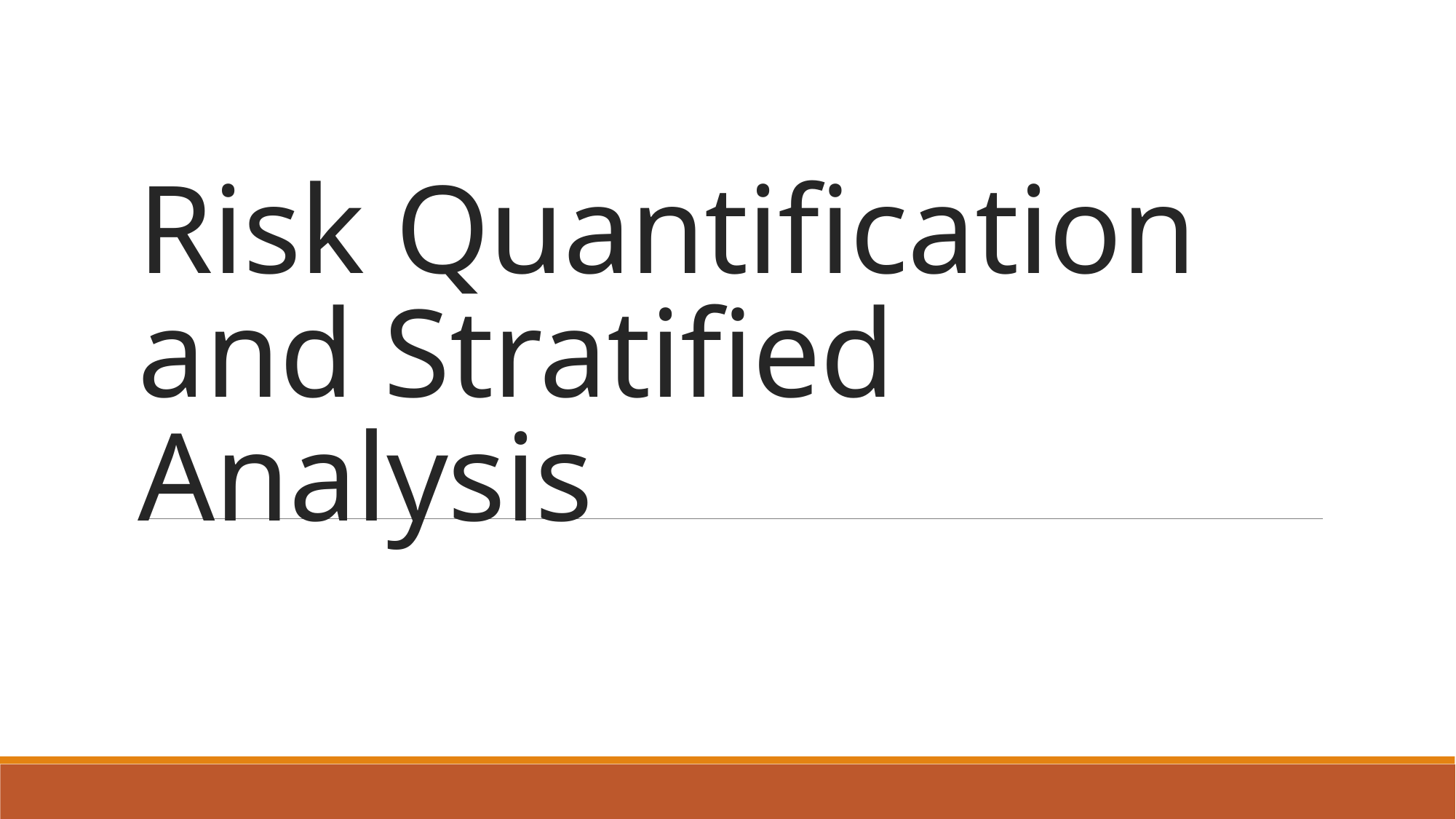

# Risk Quantification and Stratified Analysis

## Slide 11
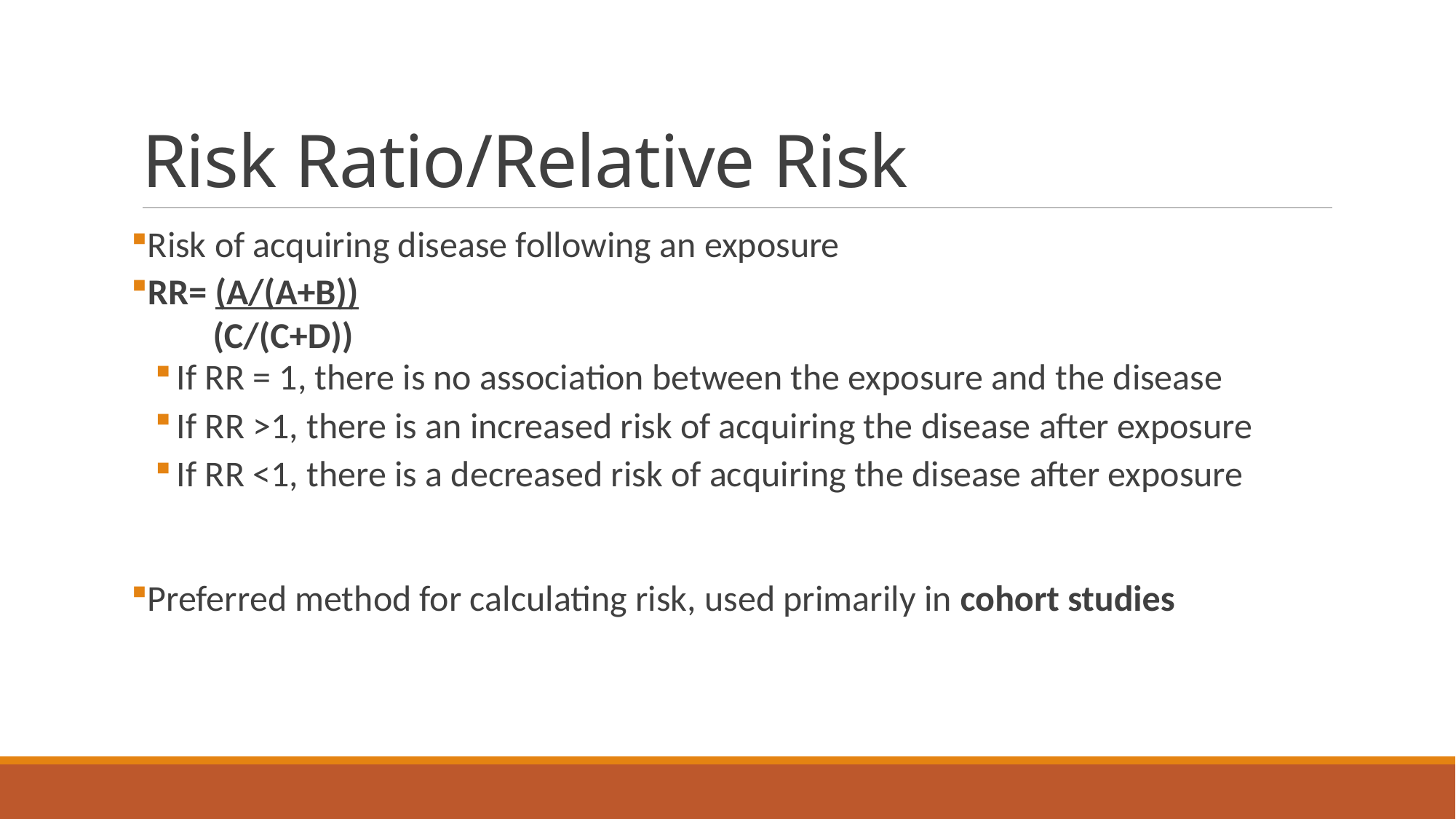

# Risk Ratio/Relative Risk
Risk of acquiring disease following an exposure
RR= (A/(A+B))
 (C/(C+D))
If RR = 1, there is no association between the exposure and the disease
If RR >1, there is an increased risk of acquiring the disease after exposure
If RR <1, there is a decreased risk of acquiring the disease after exposure
Preferred method for calculating risk, used primarily in cohort studies

## Slide 12
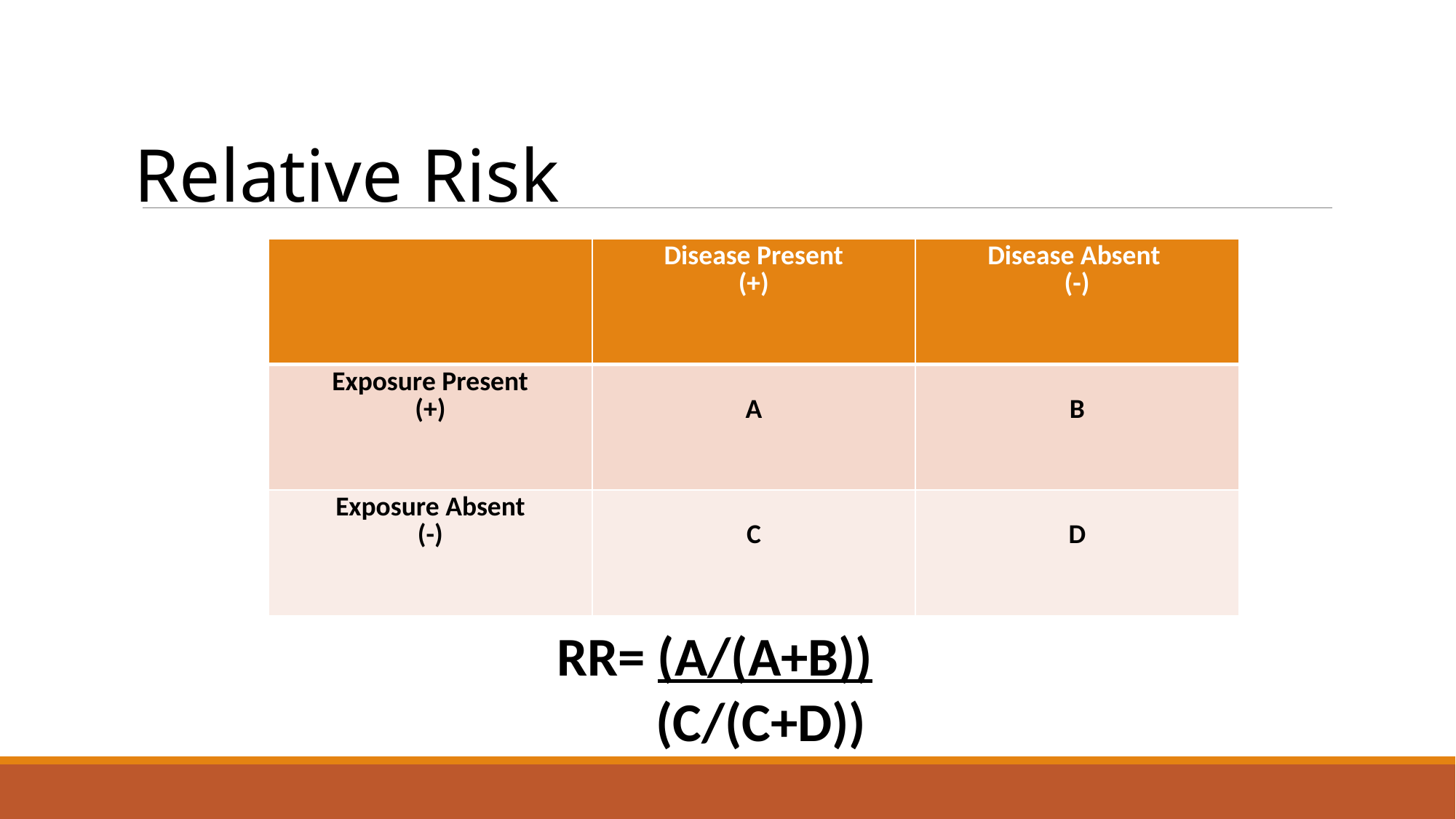

Relative Risk
| | Disease Present (+) | Disease Absent (-) |
| --- | --- | --- |
| Exposure Present (+) | A | B |
| Exposure Absent (-) | C | D |
RR= (A/(A+B))
 (C/(C+D))

## Slide 13
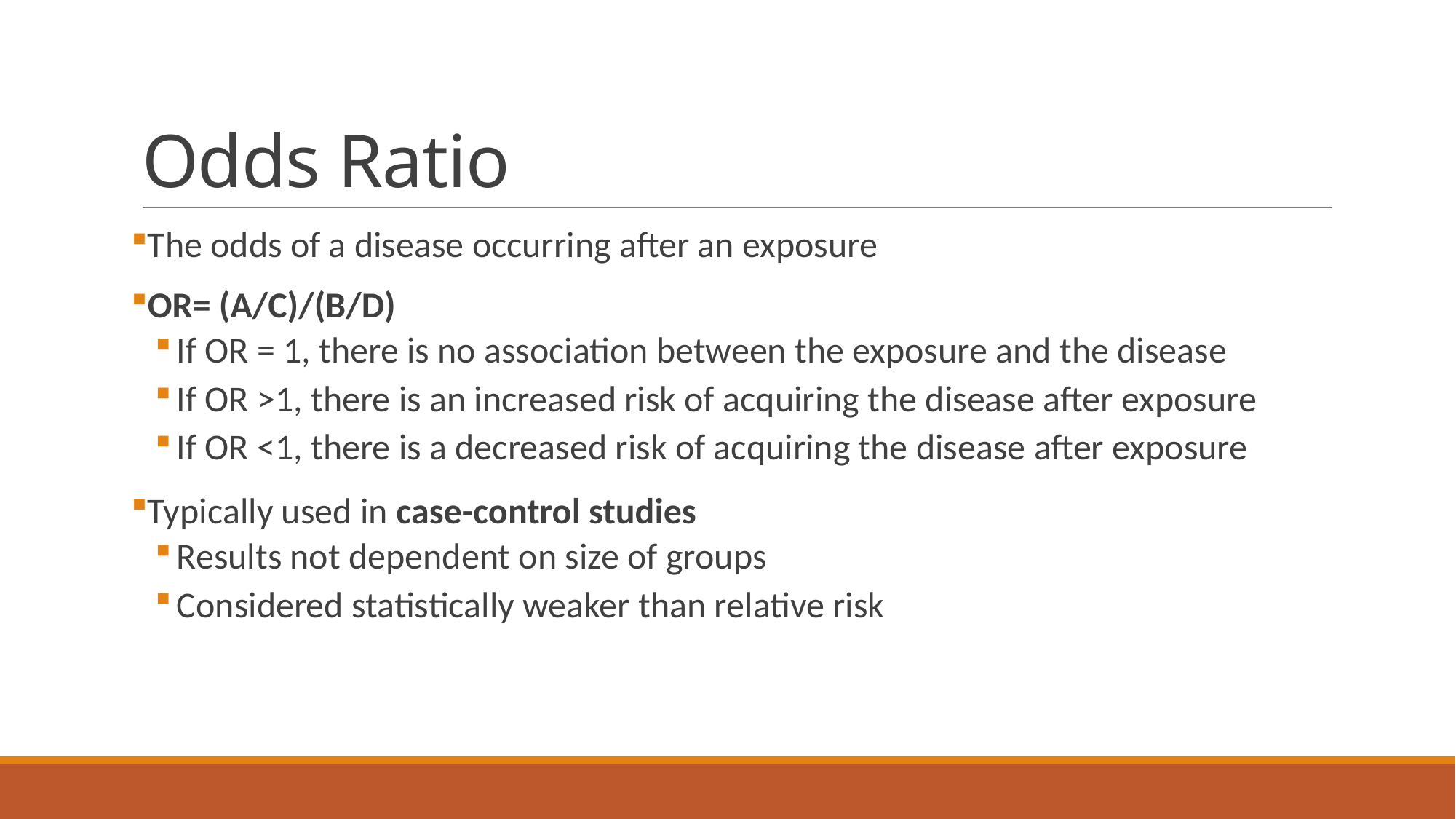

# Odds Ratio
The odds of a disease occurring after an exposure
OR= (A/C)/(B/D)
If OR = 1, there is no association between the exposure and the disease
If OR >1, there is an increased risk of acquiring the disease after exposure
If OR <1, there is a decreased risk of acquiring the disease after exposure
Typically used in case-control studies
Results not dependent on size of groups
Considered statistically weaker than relative risk

## Slide 14
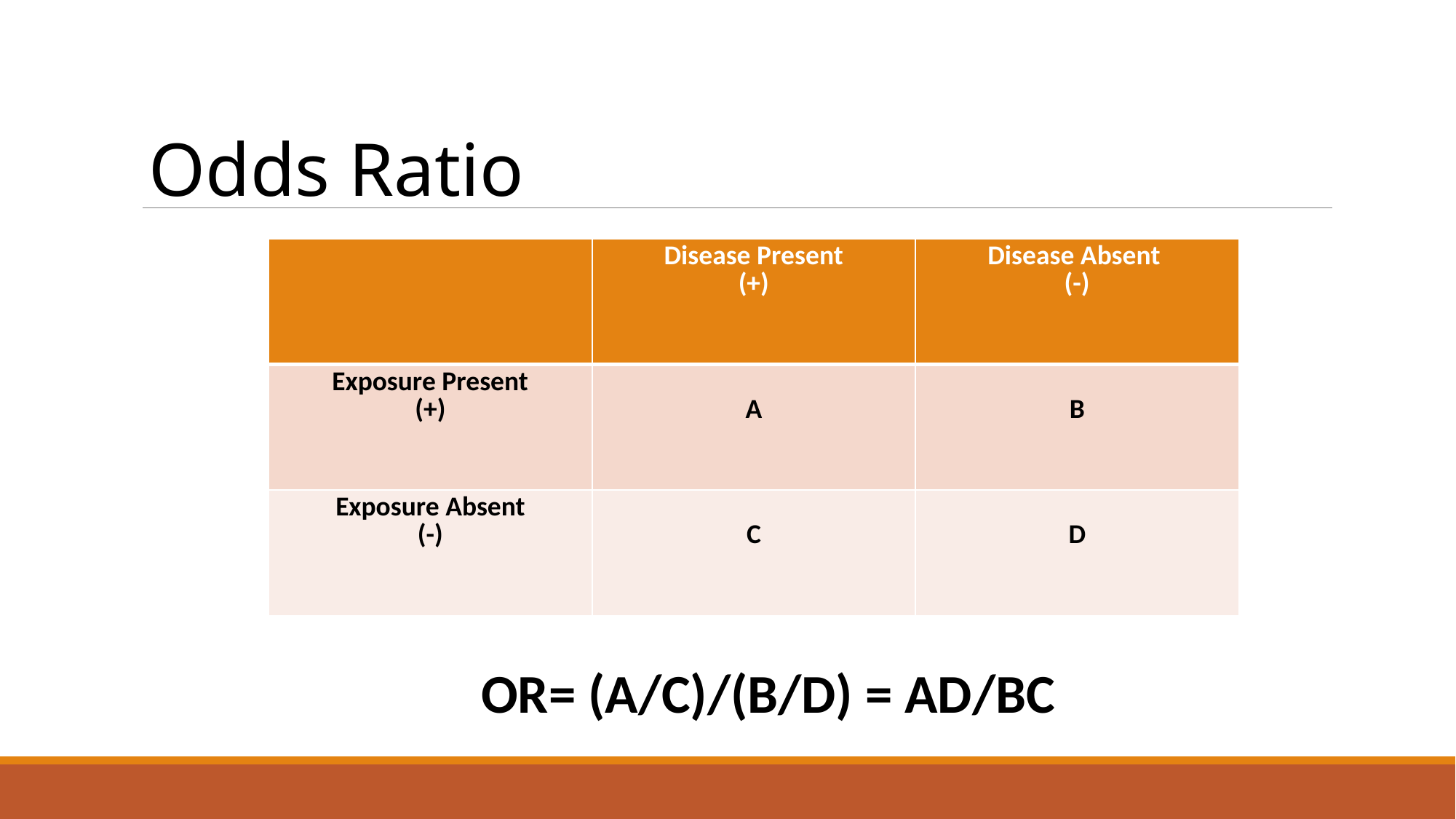

Odds Ratio
| | Disease Present (+) | Disease Absent (-) |
| --- | --- | --- |
| Exposure Present (+) | A | B |
| Exposure Absent (-) | C | D |
OR= (A/C)/(B/D) = AD/BC

## Slide 15
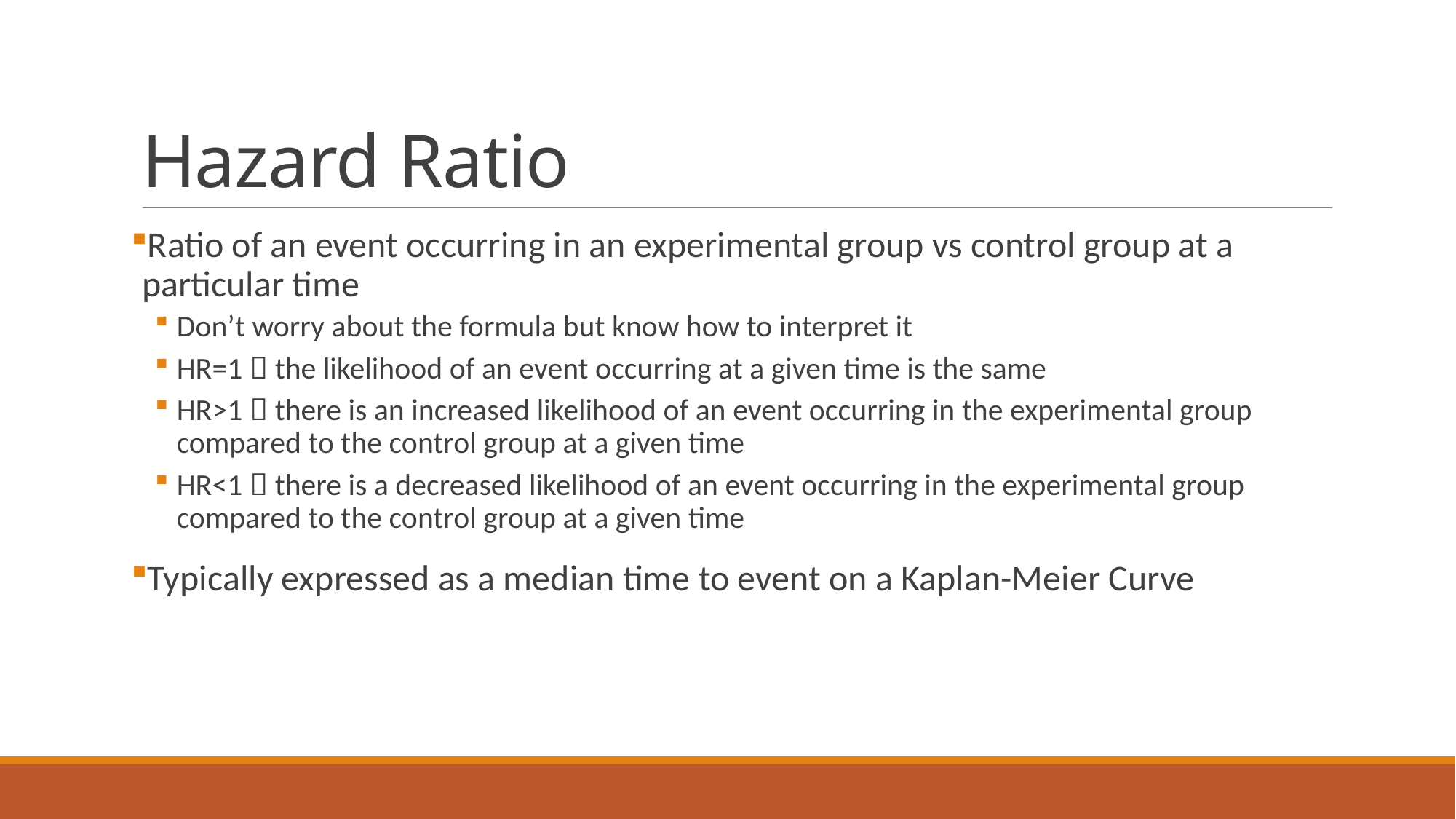

# Hazard Ratio
Ratio of an event occurring in an experimental group vs control group at a particular time
Don’t worry about the formula but know how to interpret it
HR=1  the likelihood of an event occurring at a given time is the same
HR>1  there is an increased likelihood of an event occurring in the experimental group compared to the control group at a given time
HR<1  there is a decreased likelihood of an event occurring in the experimental group compared to the control group at a given time
Typically expressed as a median time to event on a Kaplan-Meier Curve

## Slide 16
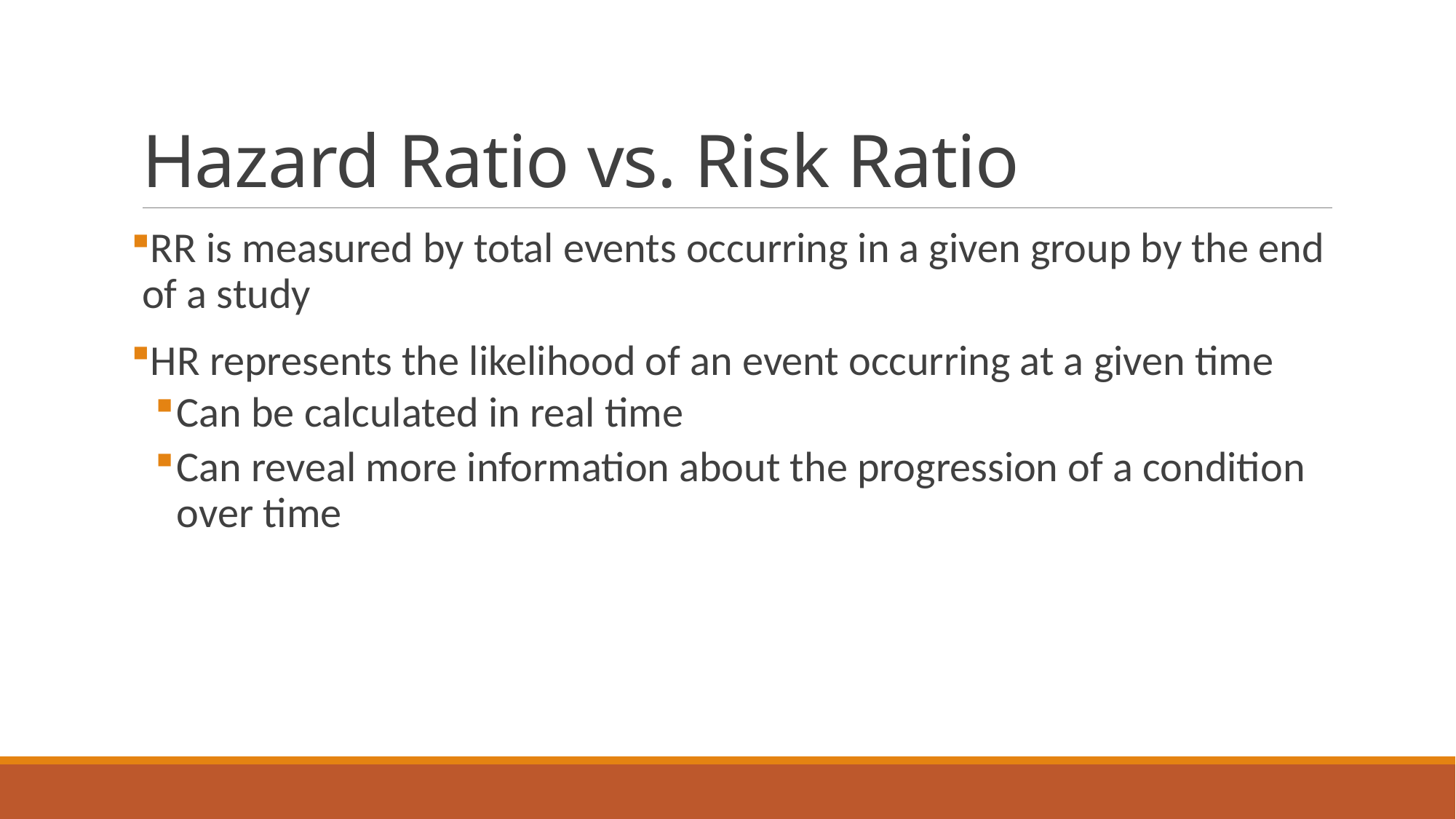

# Hazard Ratio vs. Risk Ratio
RR is measured by total events occurring in a given group by the end of a study
HR represents the likelihood of an event occurring at a given time
Can be calculated in real time
Can reveal more information about the progression of a condition over time

## Slide 17
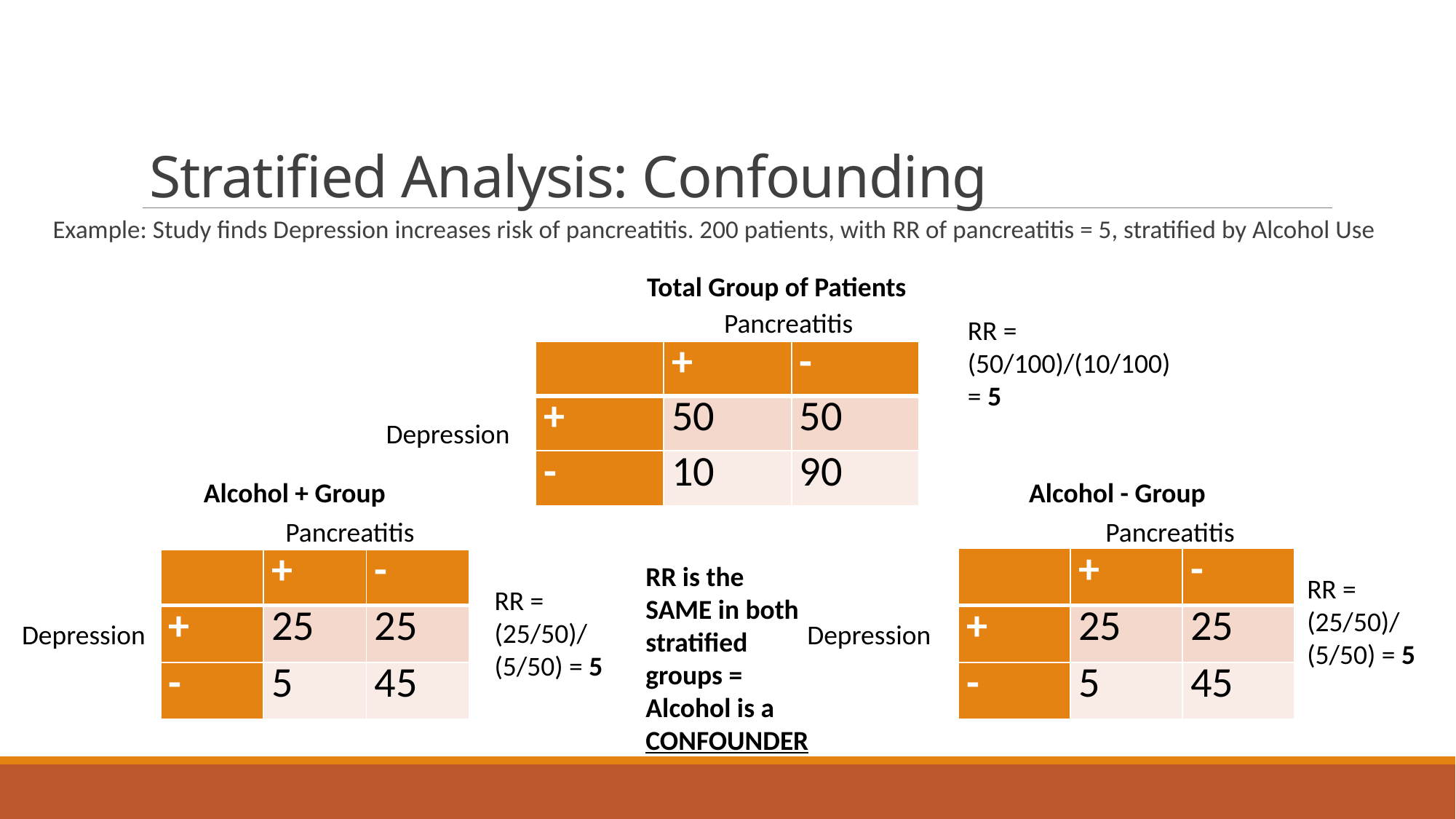

# Stratified Analysis: Confounding
Example: Study finds Depression increases risk of pancreatitis. 200 patients, with RR of pancreatitis = 5, stratified by Alcohol Use
Total Group of Patients
Pancreatitis
RR = (50/100)/(10/100) = 5
| | + | - |
| --- | --- | --- |
| + | 50 | 50 |
| - | 10 | 90 |
Depression
Alcohol + Group
Alcohol - Group
Pancreatitis
Pancreatitis
| | + | - |
| --- | --- | --- |
| + | 25 | 25 |
| - | 5 | 45 |
| | + | - |
| --- | --- | --- |
| + | 25 | 25 |
| - | 5 | 45 |
RR is the SAME in both stratified groups = Alcohol is a CONFOUNDER
RR = (25/50)/
(5/50) = 5
RR = (25/50)/
(5/50) = 5
Depression
Depression

## Slide 18
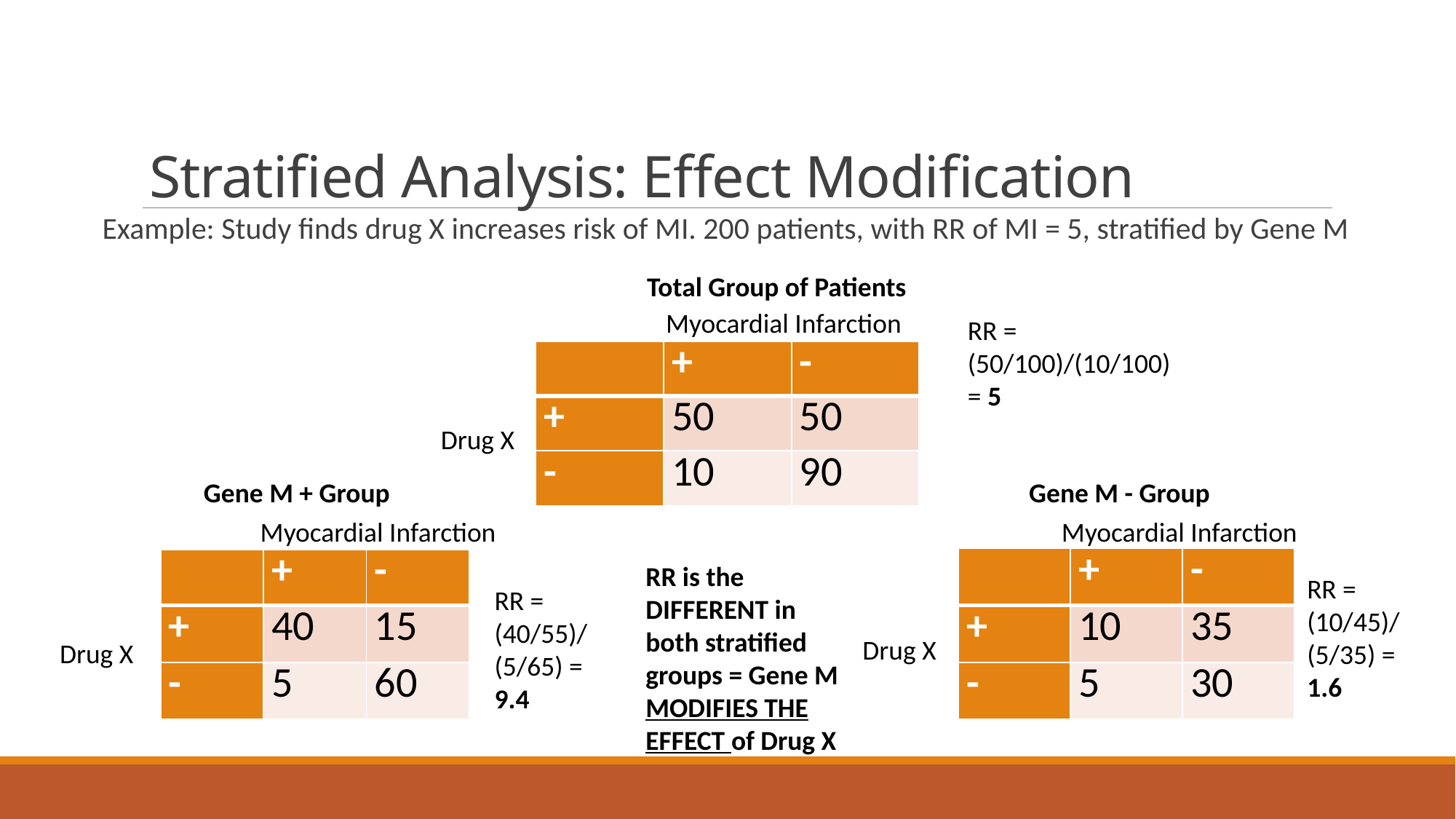

# Stratified Analysis: Effect Modification
Example: Study finds drug X increases risk of MI. 200 patients, with RR of MI = 5, stratified by Gene M
Total Group of Patients
Myocardial Infarction
RR = (50/100)/(10/100) = 5
| | + | - |
| --- | --- | --- |
| + | 50 | 50 |
| - | 10 | 90 |
Drug X
Gene M + Group
Gene M - Group
Myocardial Infarction
Myocardial Infarction
| | + | - |
| --- | --- | --- |
| + | 10 | 35 |
| - | 5 | 30 |
| | + | - |
| --- | --- | --- |
| + | 40 | 15 |
| - | 5 | 60 |
RR is the DIFFERENT in both stratified groups = Gene M MODIFIES THE EFFECT of Drug X
RR = (10/45)/
(5/35) = 1.6
RR = (40/55)/
(5/65) = 9.4
Drug X
Drug X

## Slide 19
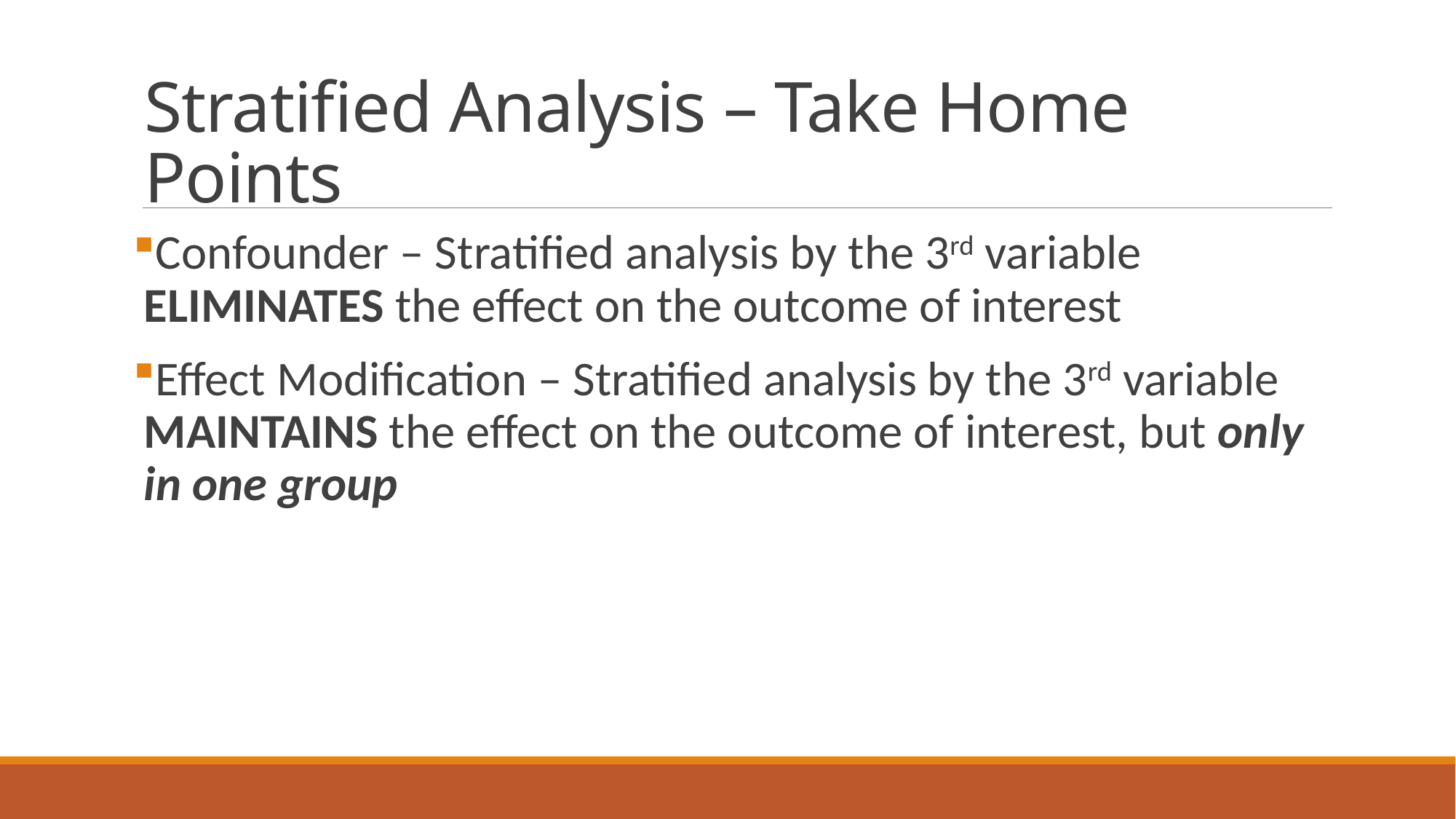

# Stratified Analysis – Take Home Points
Confounder – Stratified analysis by the 3rd variable ELIMINATES the effect on the outcome of interest
Effect Modification – Stratified analysis by the 3rd variable MAINTAINS the effect on the outcome of interest, but only in one group

## Slide 20
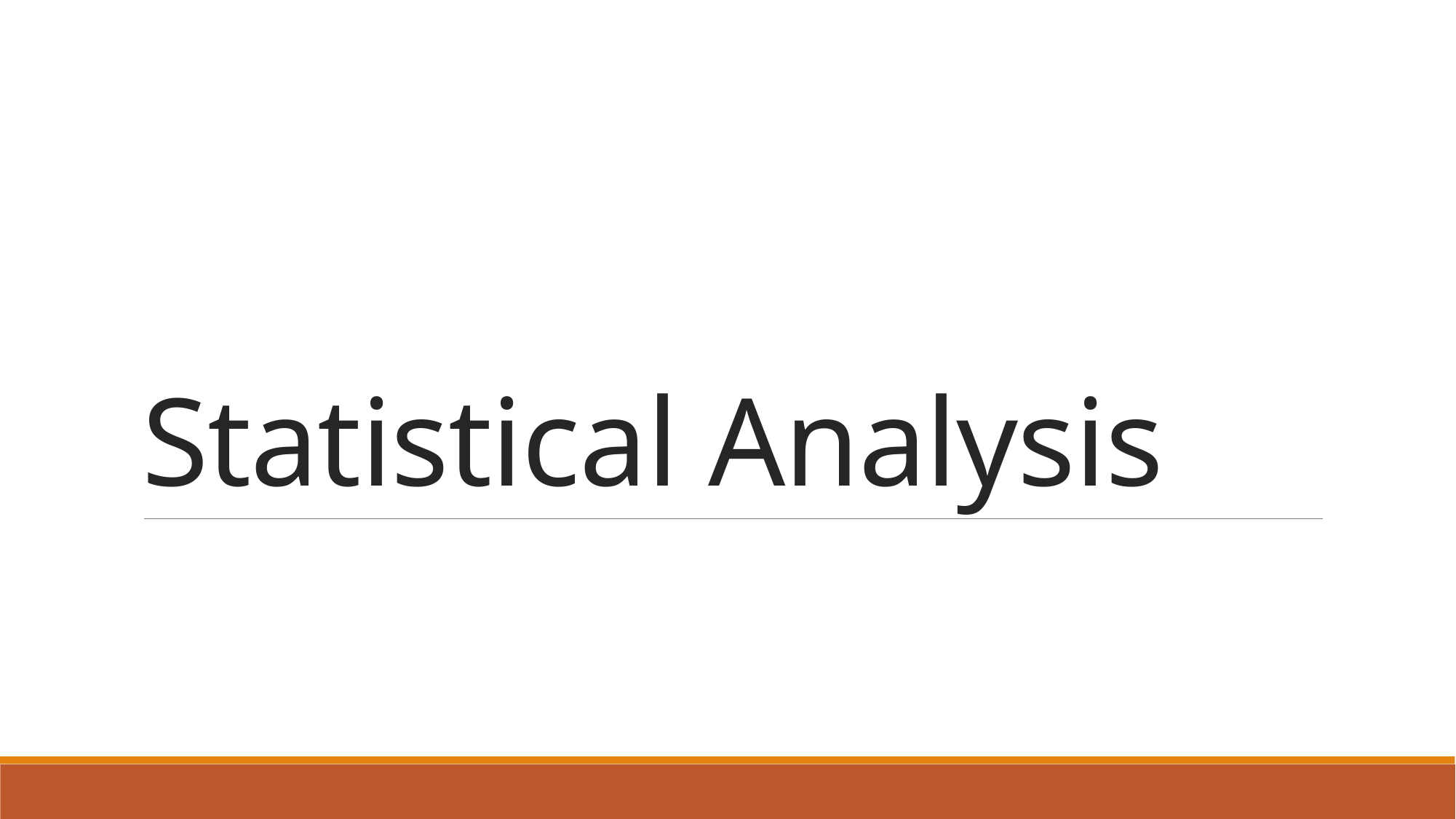

# Statistical Analysis

## Slide 21
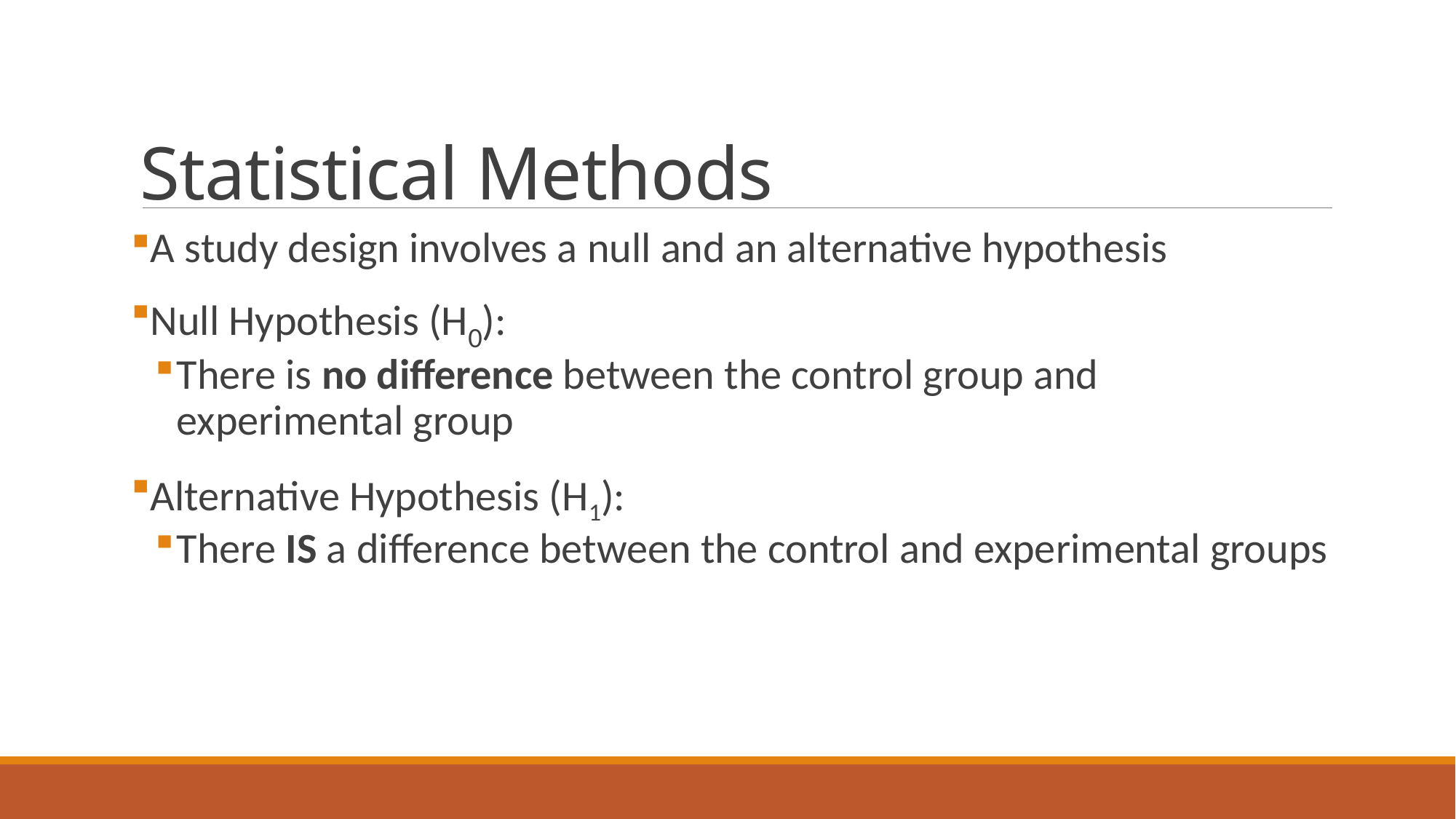

# Statistical Methods
A study design involves a null and an alternative hypothesis
Null Hypothesis (H0):
There is no difference between the control group and experimental group
Alternative Hypothesis (H1):
There IS a difference between the control and experimental groups

## Slide 22
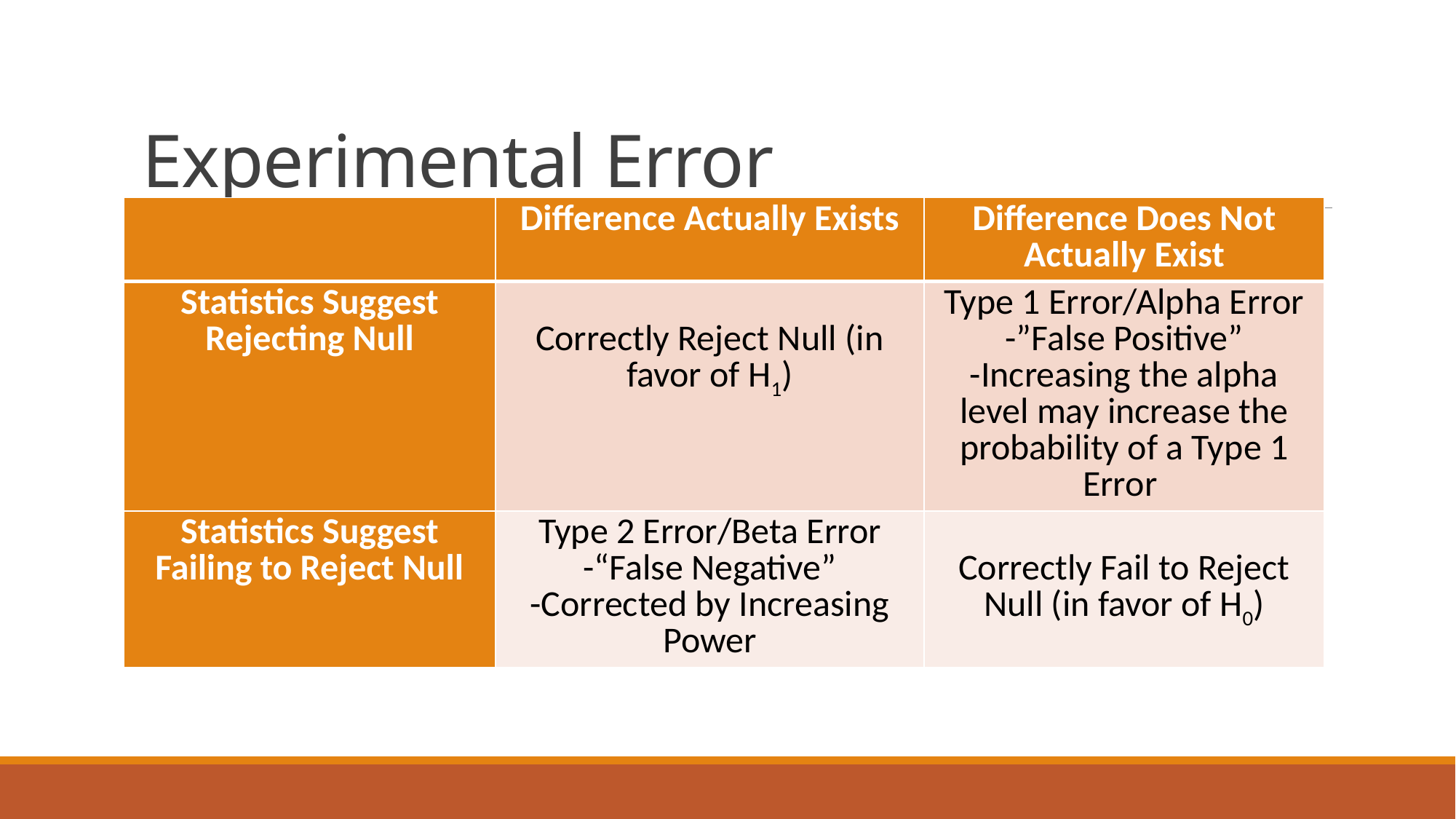

# Experimental Error
| | Difference Actually Exists | Difference Does Not Actually Exist |
| --- | --- | --- |
| Statistics Suggest Rejecting Null | Correctly Reject Null (in favor of H1) | Type 1 Error/Alpha Error -”False Positive” -Increasing the alpha level may increase the probability of a Type 1 Error |
| Statistics Suggest Failing to Reject Null | Type 2 Error/Beta Error -“False Negative” -Corrected by Increasing Power | Correctly Fail to Reject Null (in favor of H0) |

## Slide 23
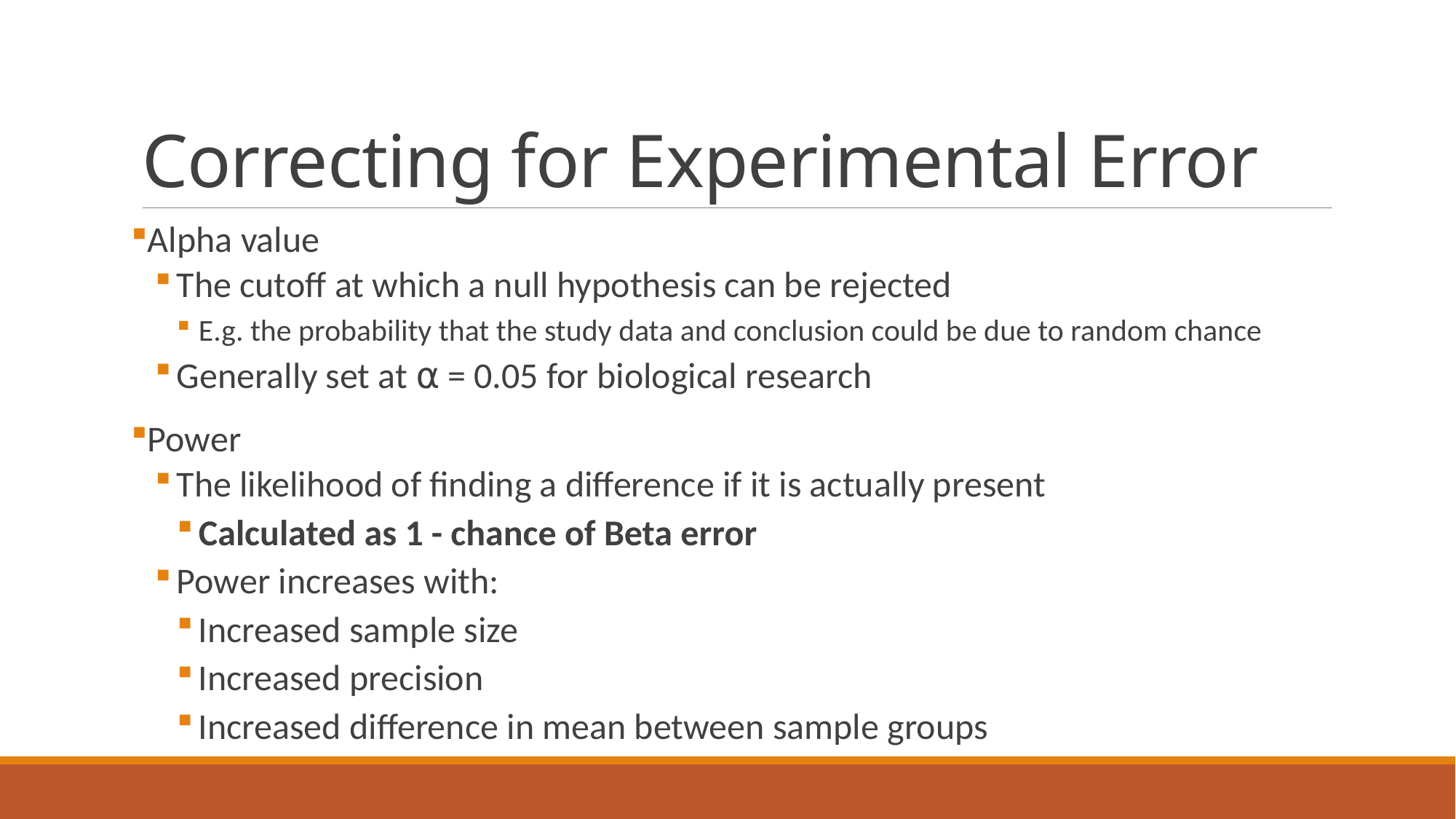

# Correcting for Experimental Error
Alpha value
The cutoff at which a null hypothesis can be rejected
E.g. the probability that the study data and conclusion could be due to random chance
Generally set at ⍺ = 0.05 for biological research
Power
The likelihood of finding a difference if it is actually present
Calculated as 1 - chance of Beta error
Power increases with:
Increased sample size
Increased precision
Increased difference in mean between sample groups

## Slide 24
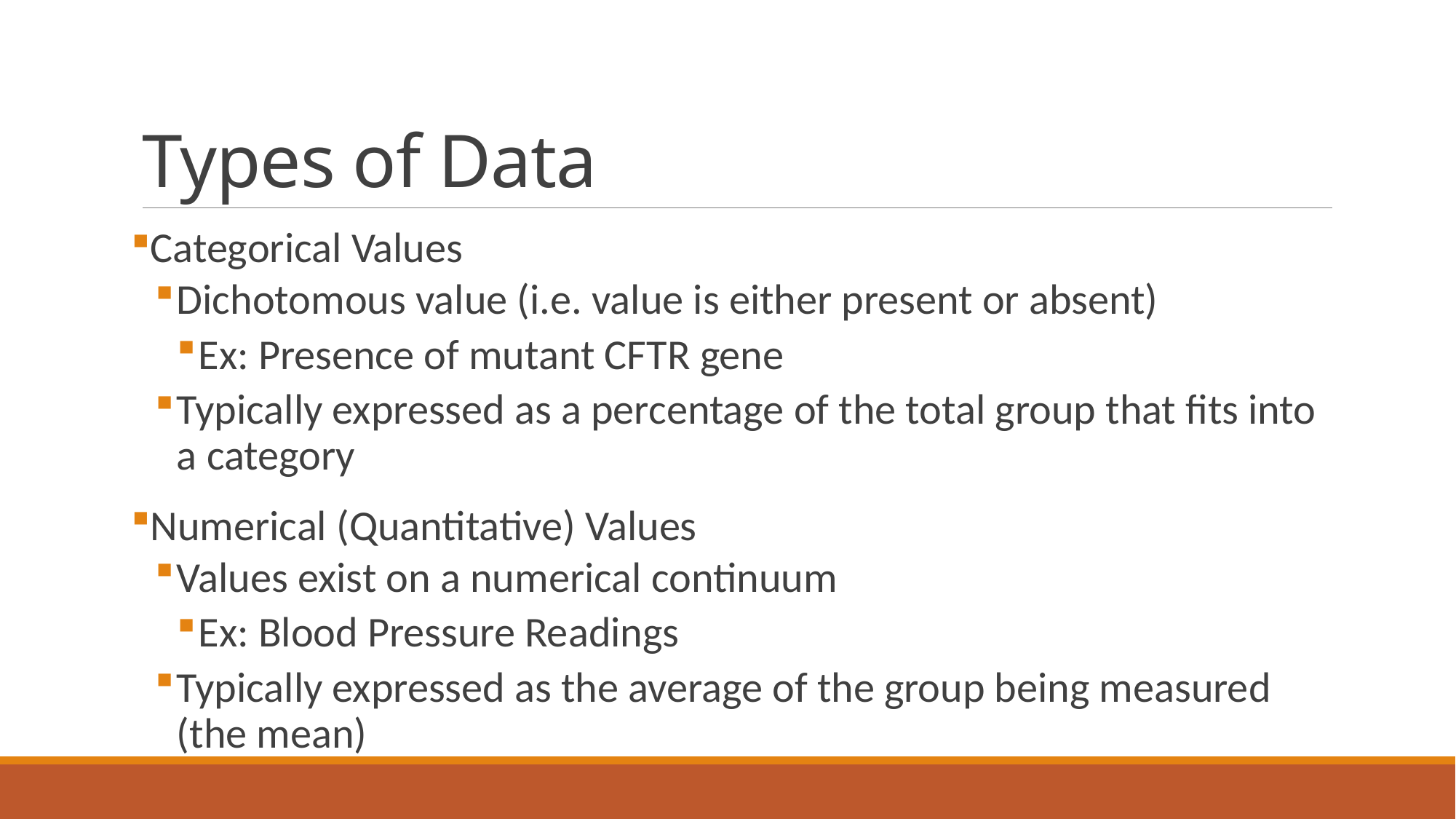

# Types of Data
Categorical Values
Dichotomous value (i.e. value is either present or absent)
Ex: Presence of mutant CFTR gene
Typically expressed as a percentage of the total group that fits into a category
Numerical (Quantitative) Values
Values exist on a numerical continuum
Ex: Blood Pressure Readings
Typically expressed as the average of the group being measured (the mean)

## Slide 25
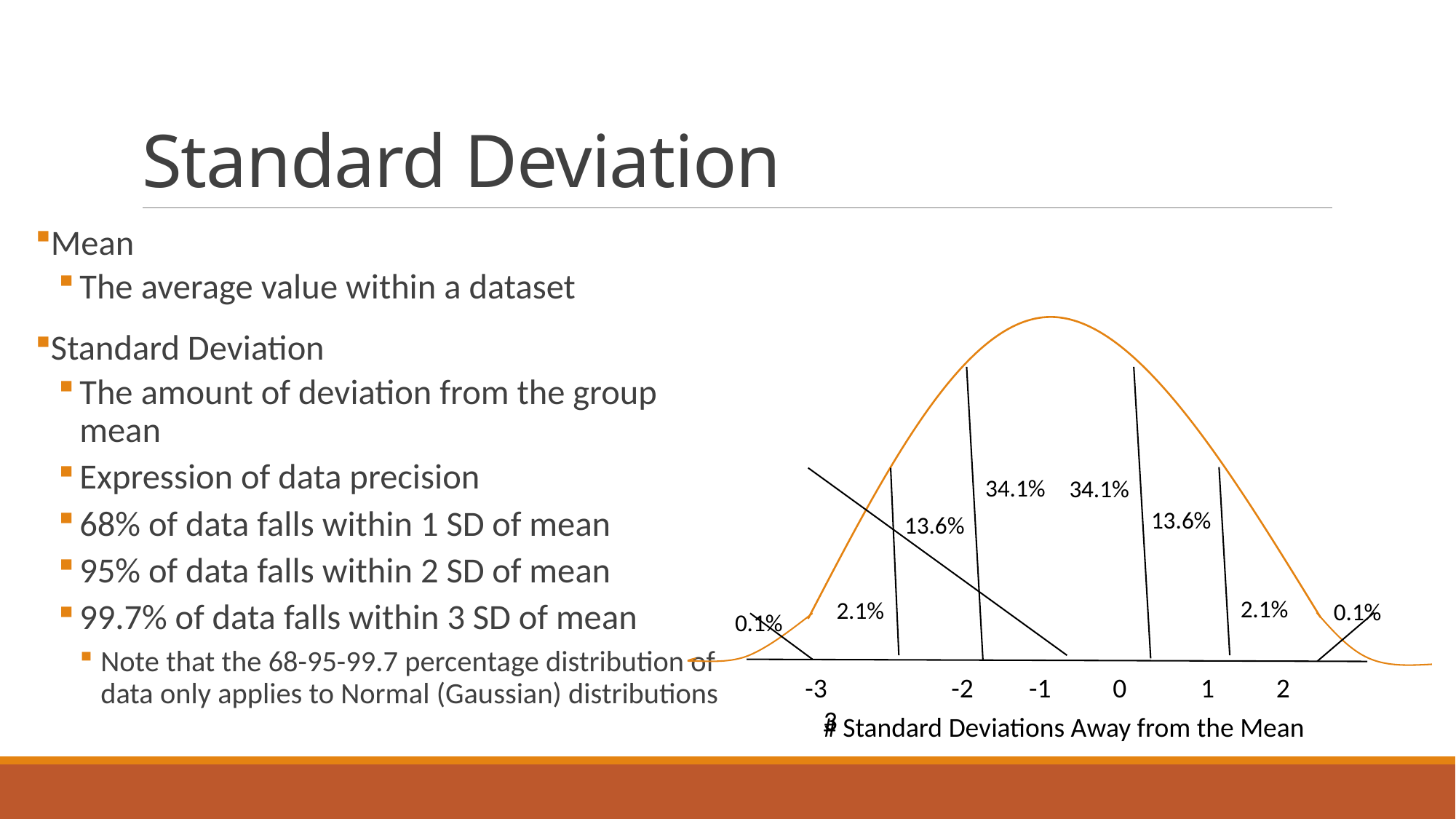

# Standard Deviation
Mean
The average value within a dataset
Standard Deviation
The amount of deviation from the group mean
Expression of data precision
68% of data falls within 1 SD of mean
95% of data falls within 2 SD of mean
99.7% of data falls within 3 SD of mean
Note that the 68-95-99.7 percentage distribution of data only applies to Normal (Gaussian) distributions
34.1%
34.1%
13.6%
13.6%
2.1%
2.1%
0.1%
0.1%
-3	 -2 -1 0 1 2 3
# Standard Deviations Away from the Mean

## Slide 26
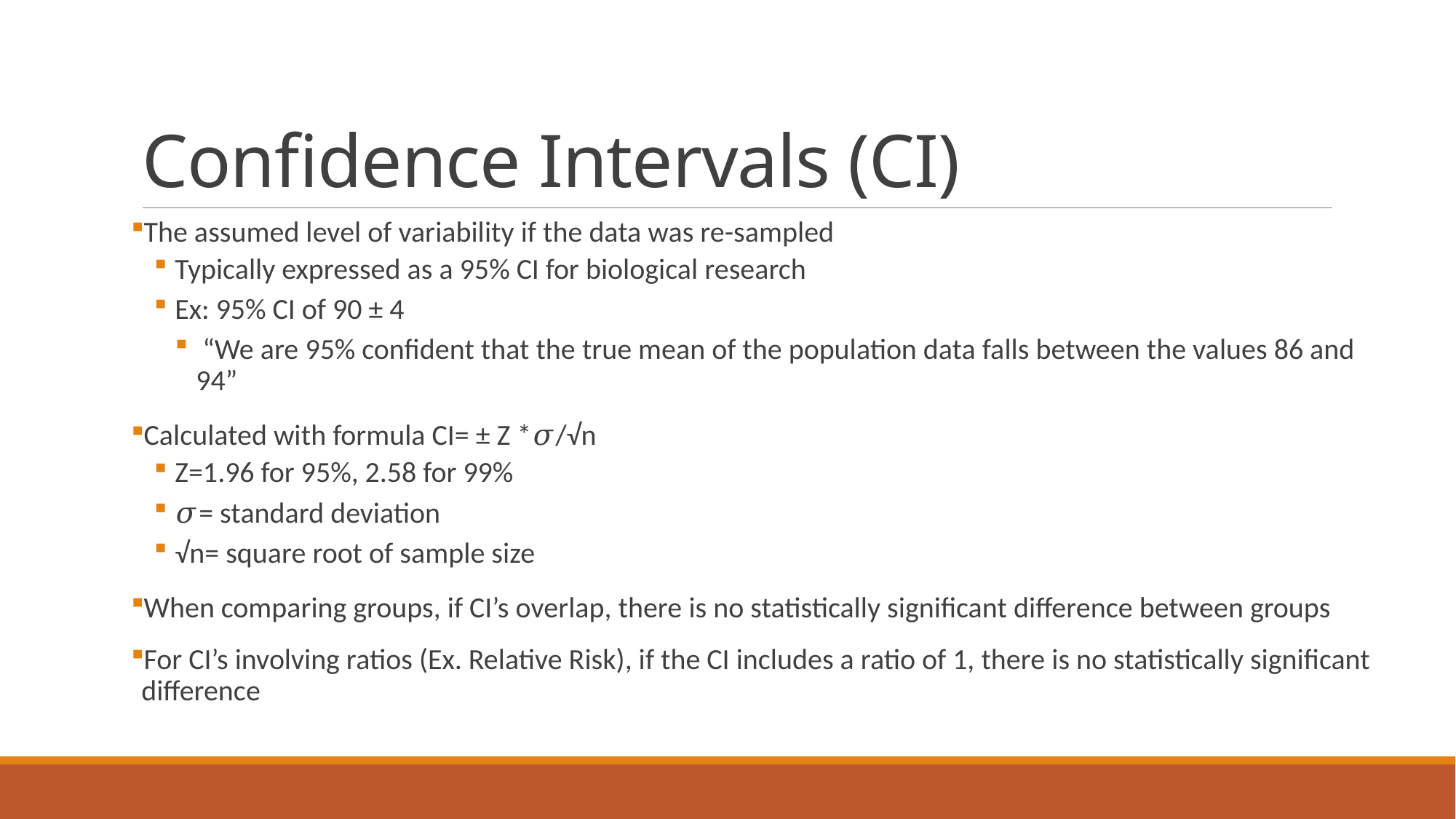

# Confidence Intervals (CI)
The assumed level of variability if the data was re-sampled
Typically expressed as a 95% CI for biological research
Ex: 95% CI of 90 ± 4
 “We are 95% confident that the true mean of the population data falls between the values 86 and 94”
Calculated with formula CI= ± Z *𝜎/√n
Z=1.96 for 95%, 2.58 for 99%
𝜎= standard deviation
√n= square root of sample size
When comparing groups, if CI’s overlap, there is no statistically significant difference between groups
For CI’s involving ratios (Ex. Relative Risk), if the CI includes a ratio of 1, there is no statistically significant difference

## Slide 27
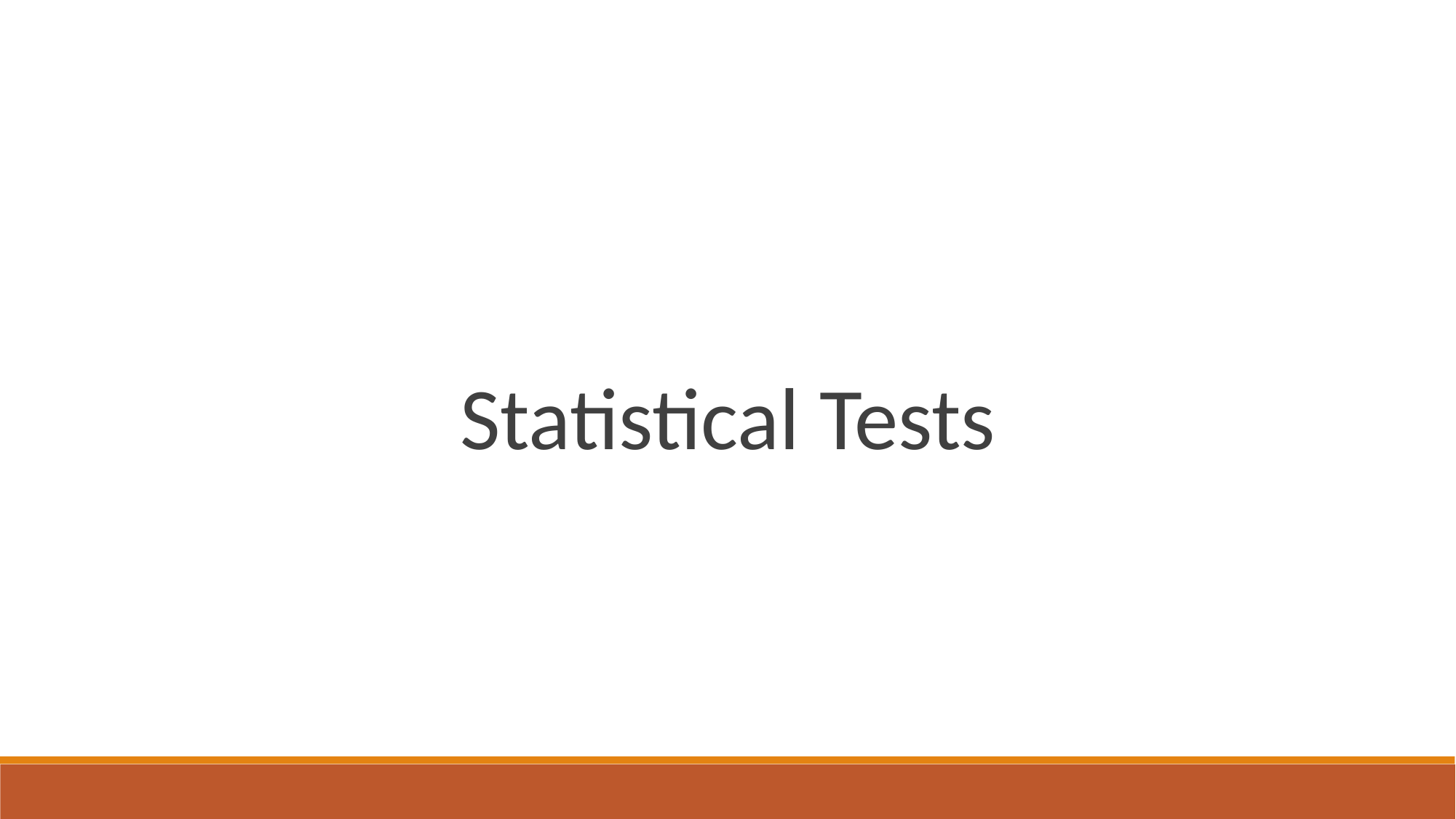

Statistical Tests

## Slide 28
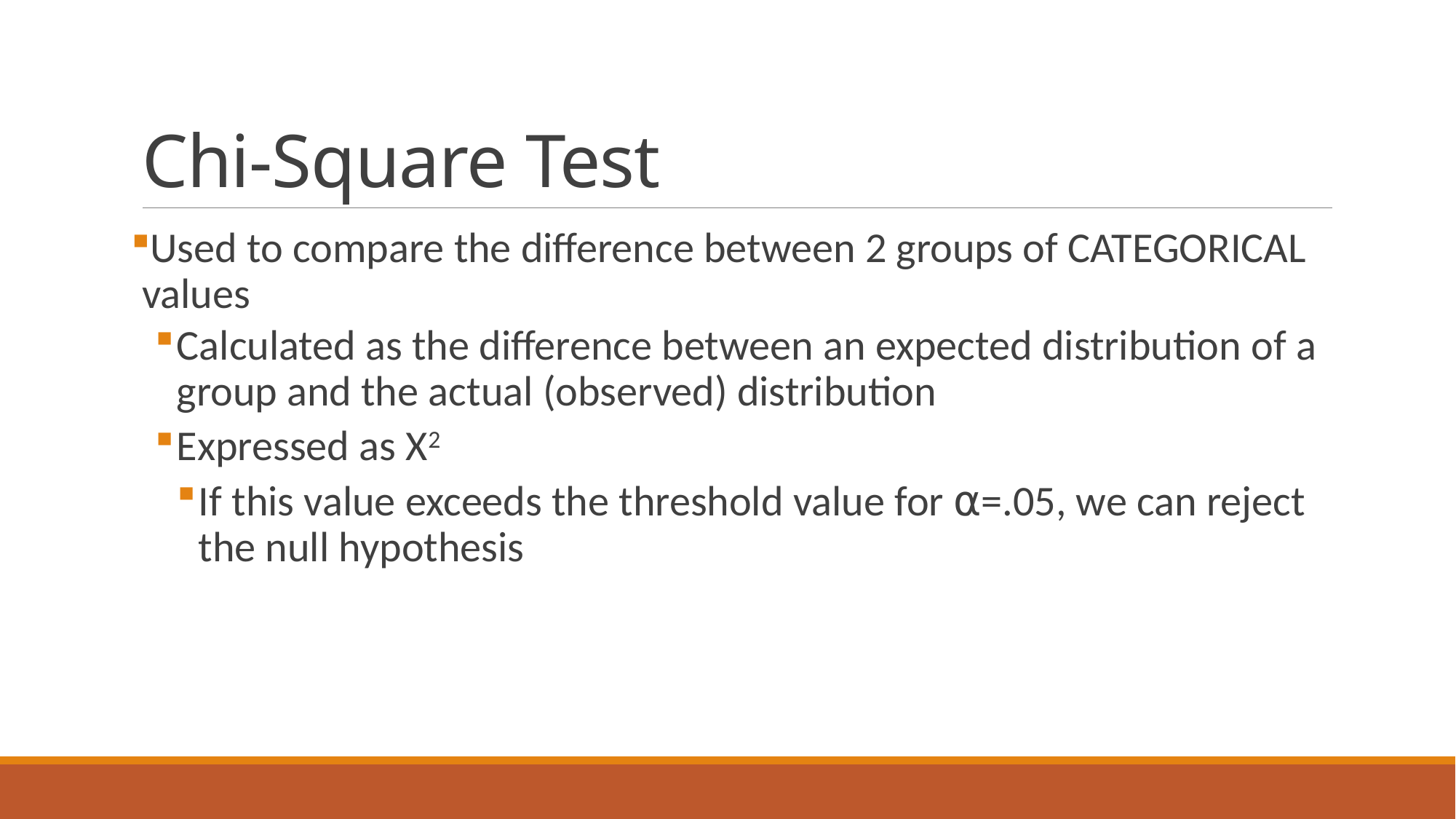

# Chi-Square Test
Used to compare the difference between 2 groups of CATEGORICAL values
Calculated as the difference between an expected distribution of a group and the actual (observed) distribution
Expressed as X2
If this value exceeds the threshold value for ⍺=.05, we can reject the null hypothesis

## Slide 29
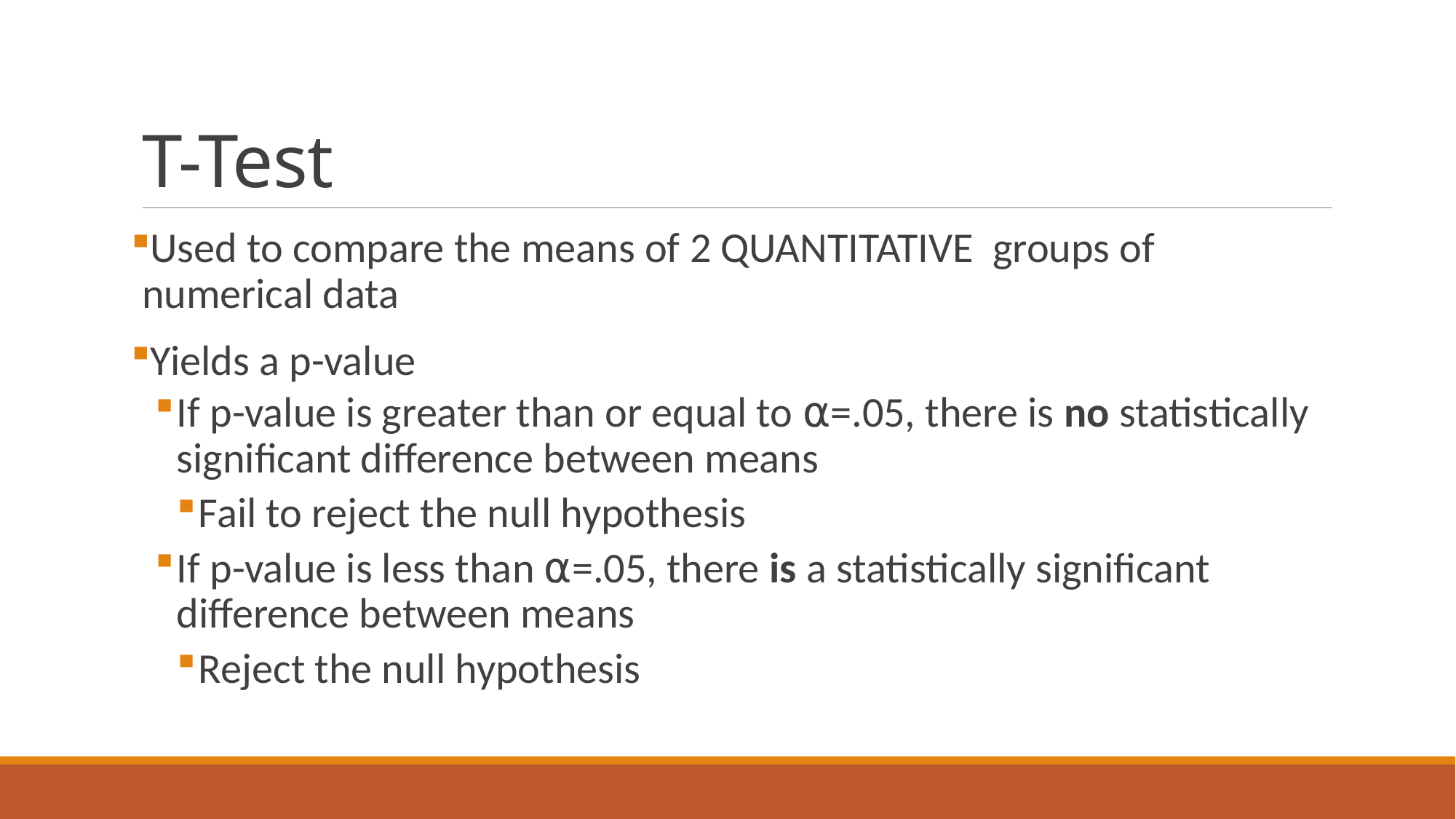

# T-Test
Used to compare the means of 2 QUANTITATIVE groups of numerical data
Yields a p-value
If p-value is greater than or equal to ⍺=.05, there is no statistically significant difference between means
Fail to reject the null hypothesis
If p-value is less than ⍺=.05, there is a statistically significant difference between means
Reject the null hypothesis

## Slide 30
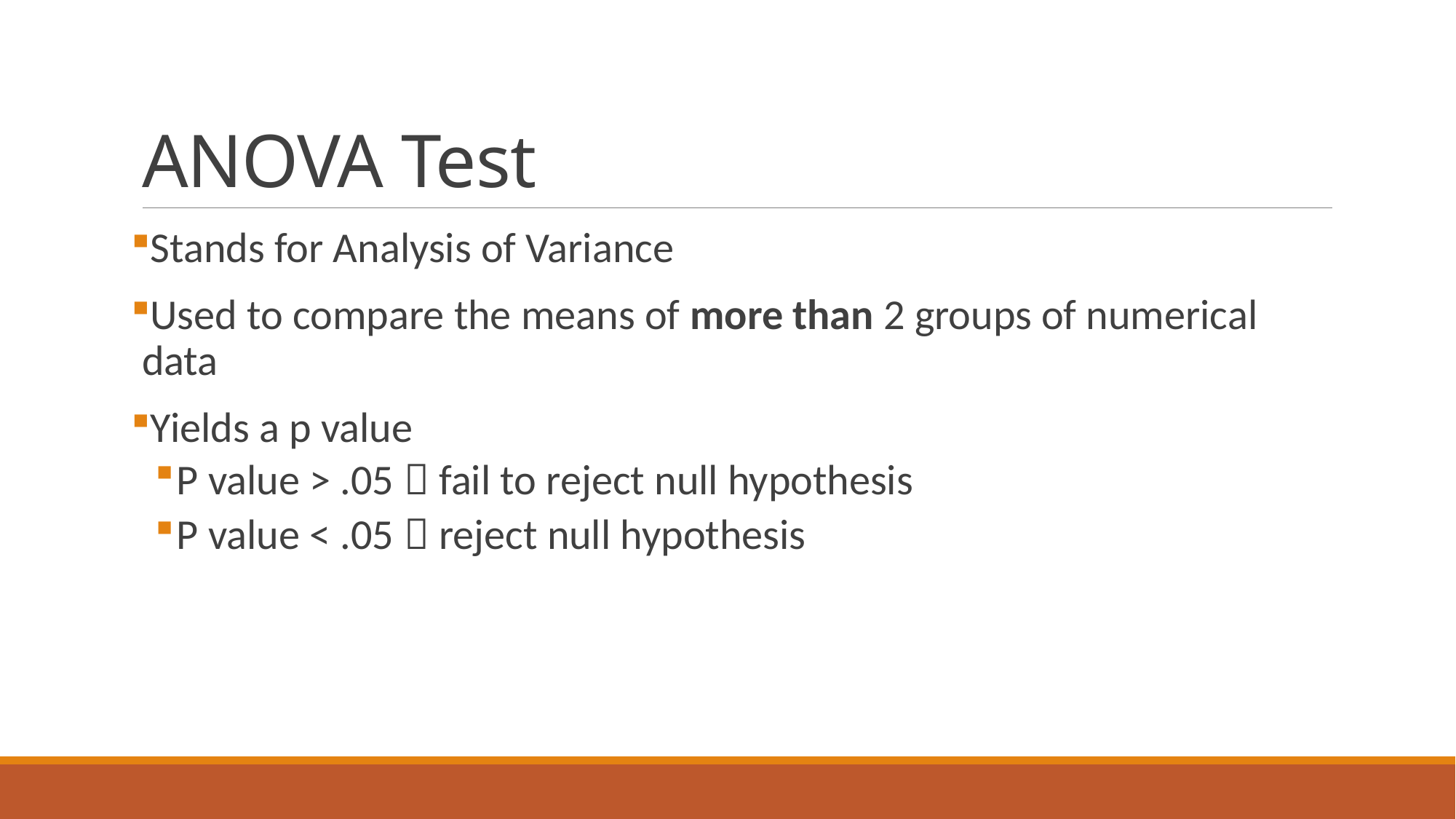

# ANOVA Test
Stands for Analysis of Variance
Used to compare the means of more than 2 groups of numerical data
Yields a p value
P value > .05  fail to reject null hypothesis
P value < .05  reject null hypothesis

## Slide 31
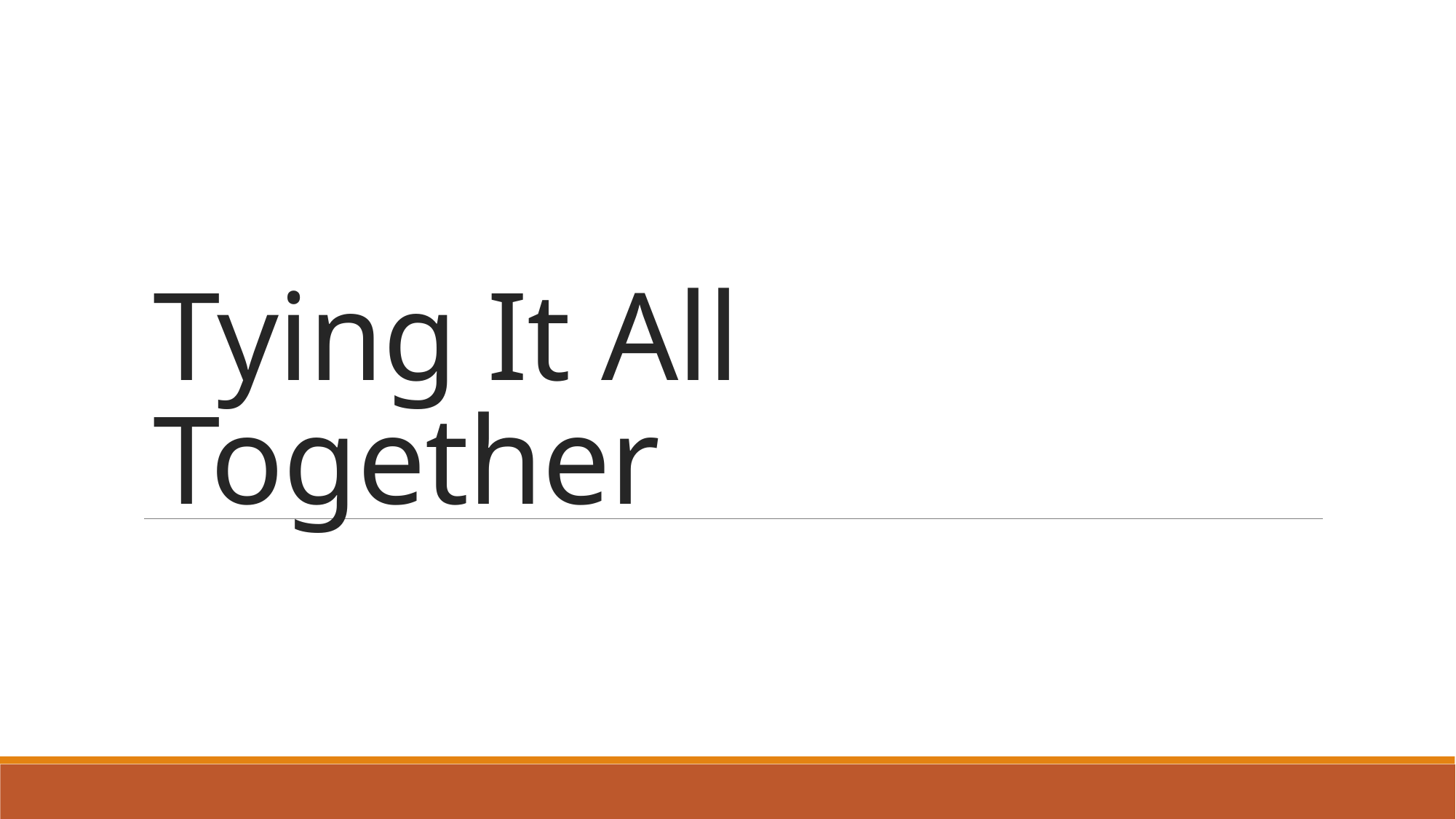

# Tying It All Together

## Slide 32
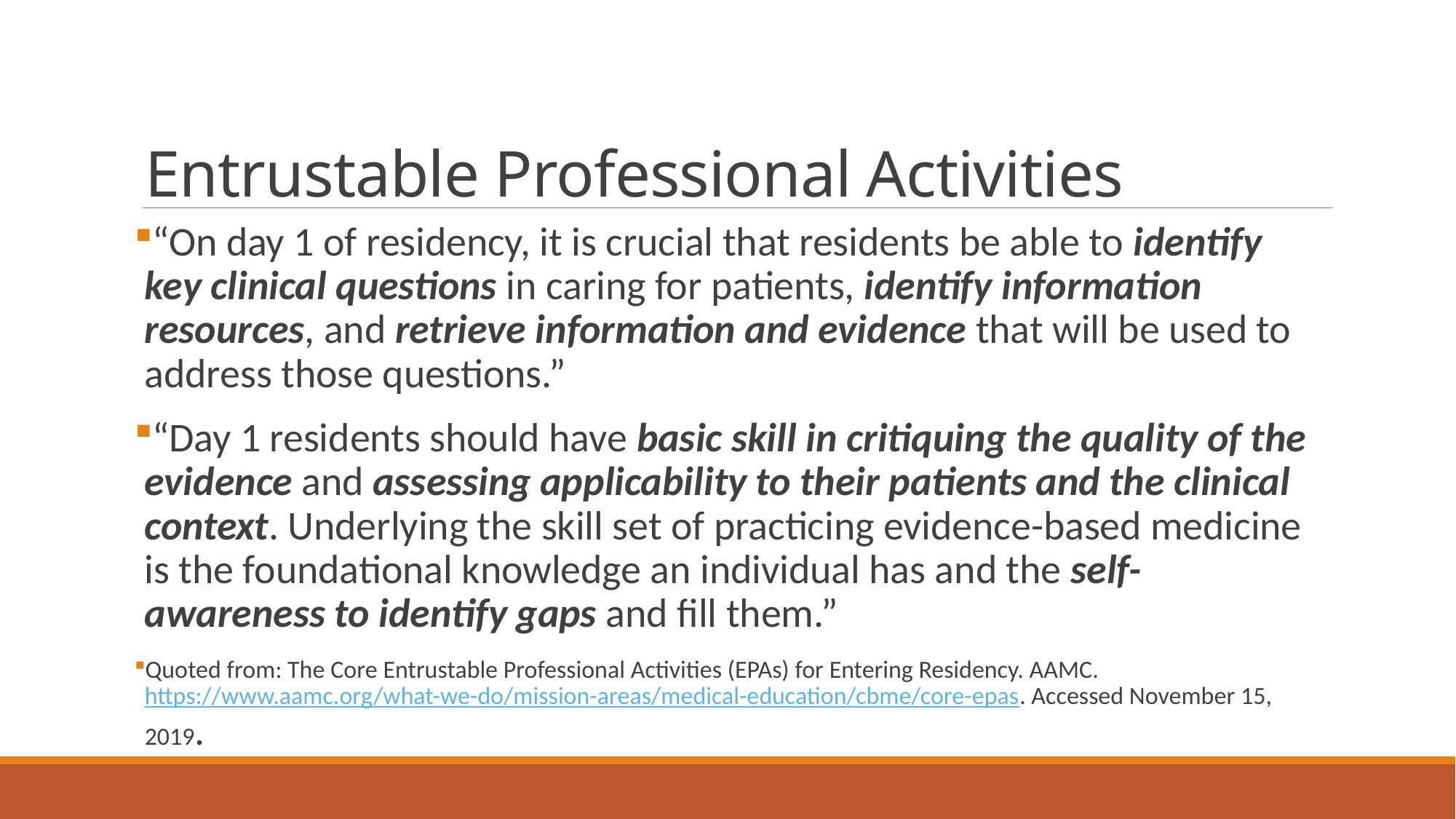

# Entrustable Professional Activities
“On day 1 of residency, it is crucial that residents be able to identify key clinical questions in caring for patients, identify information resources, and retrieve information and evidence that will be used to address those questions.”
“Day 1 residents should have basic skill in critiquing the quality of the evidence and assessing applicability to their patients and the clinical context. Underlying the skill set of practicing evidence-based medicine is the foundational knowledge an individual has and the self-awareness to identify gaps and fill them.”
Quoted from: The Core Entrustable Professional Activities (EPAs) for Entering Residency. AAMC. https://www.aamc.org/what-we-do/mission-areas/medical-education/cbme/core-epas. Accessed November 15, 2019.

## Slide 33
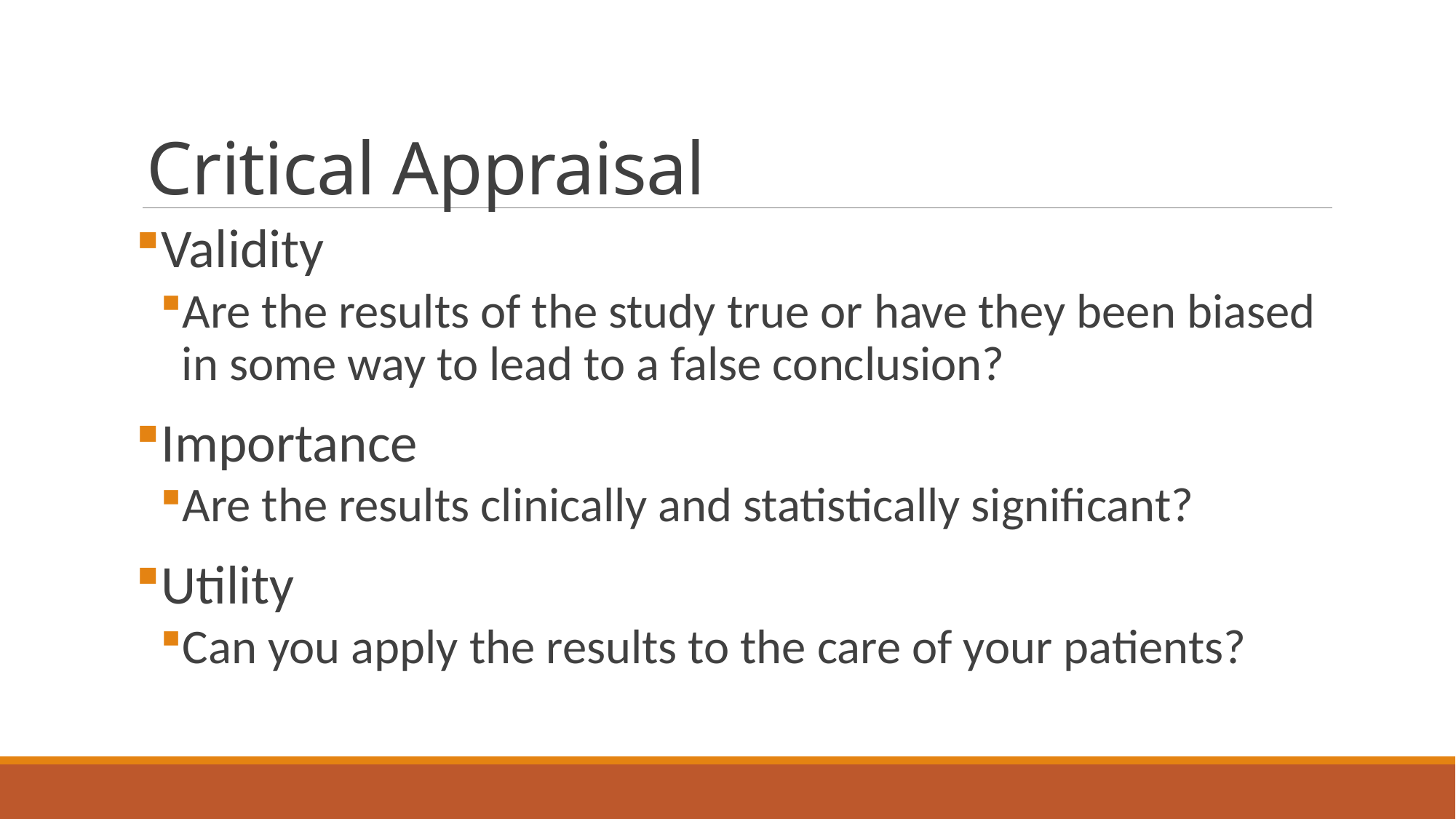

# Critical Appraisal
Validity
Are the results of the study true or have they been biased in some way to lead to a false conclusion?
Importance
Are the results clinically and statistically significant?
Utility
Can you apply the results to the care of your patients?

## Slide 34
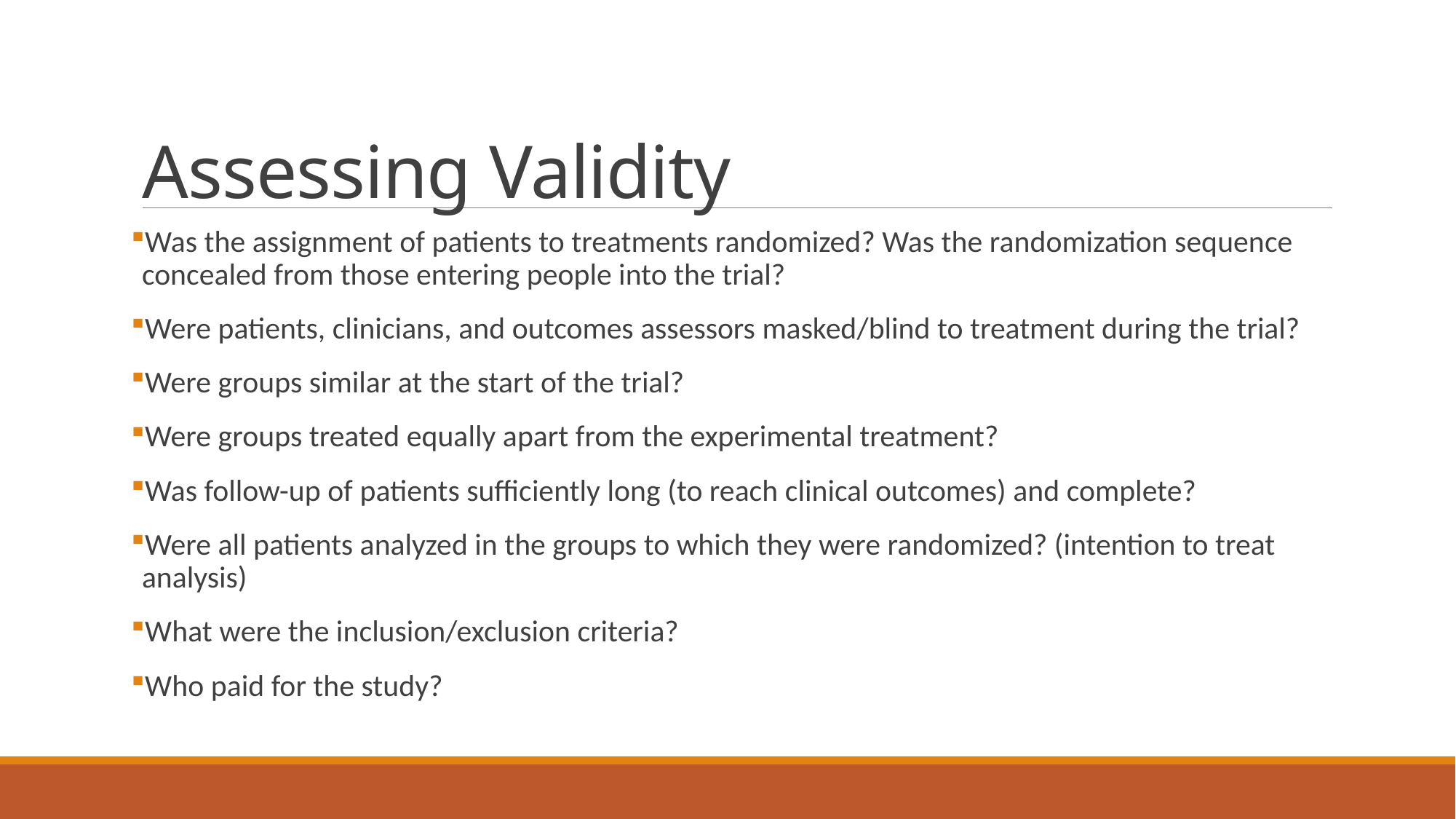

# Assessing Validity
Was the assignment of patients to treatments randomized? Was the randomization sequence concealed from those entering people into the trial?
Were patients, clinicians, and outcomes assessors masked/blind to treatment during the trial?
Were groups similar at the start of the trial?
Were groups treated equally apart from the experimental treatment?
Was follow-up of patients sufficiently long (to reach clinical outcomes) and complete?
Were all patients analyzed in the groups to which they were randomized? (intention to treat analysis)
What were the inclusion/exclusion criteria?
Who paid for the study?

## Slide 35
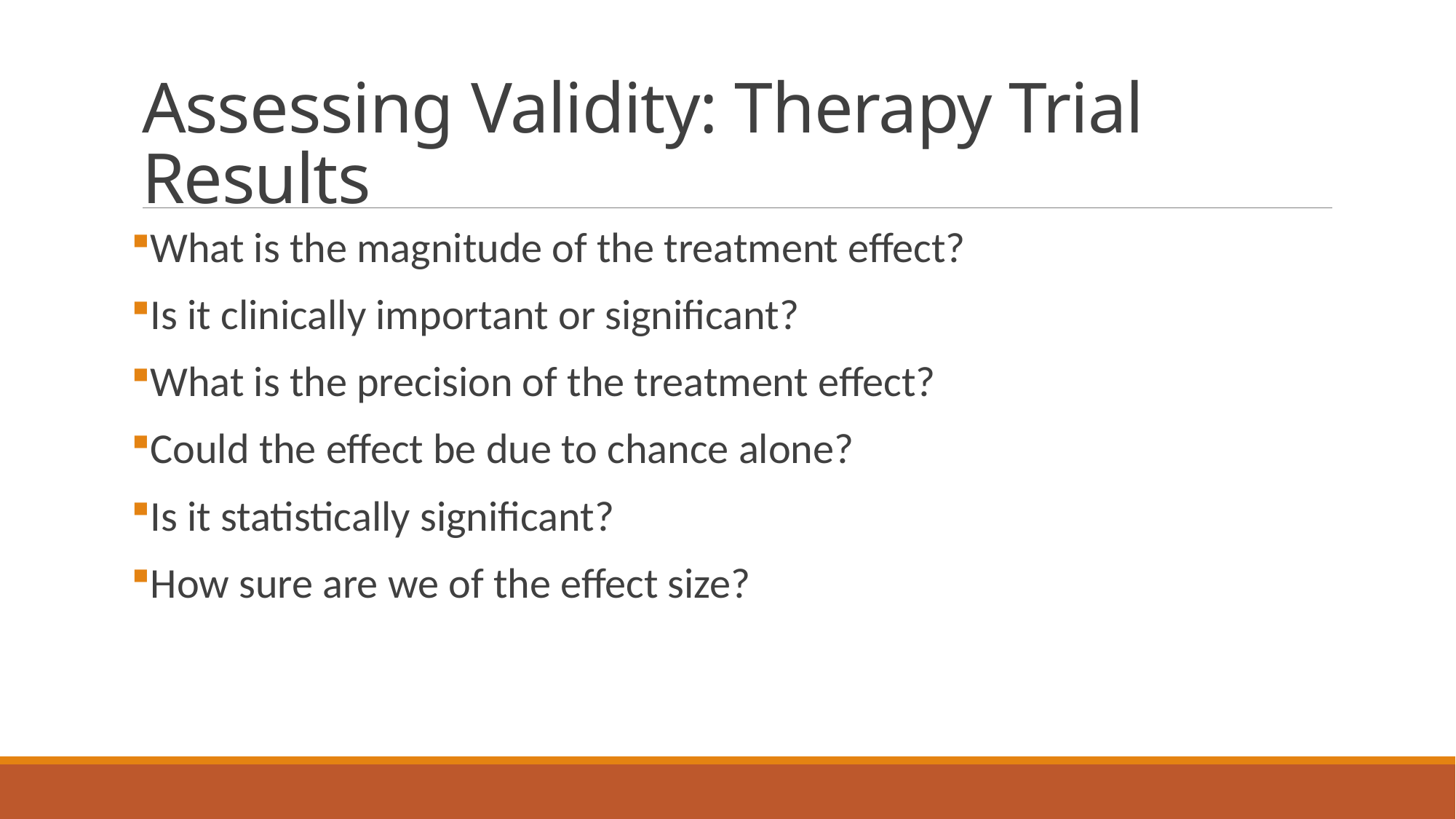

# Assessing Validity: Therapy Trial Results
What is the magnitude of the treatment effect?
Is it clinically important or significant?
What is the precision of the treatment effect?
Could the effect be due to chance alone?
Is it statistically significant?
How sure are we of the effect size?

## Slide 36
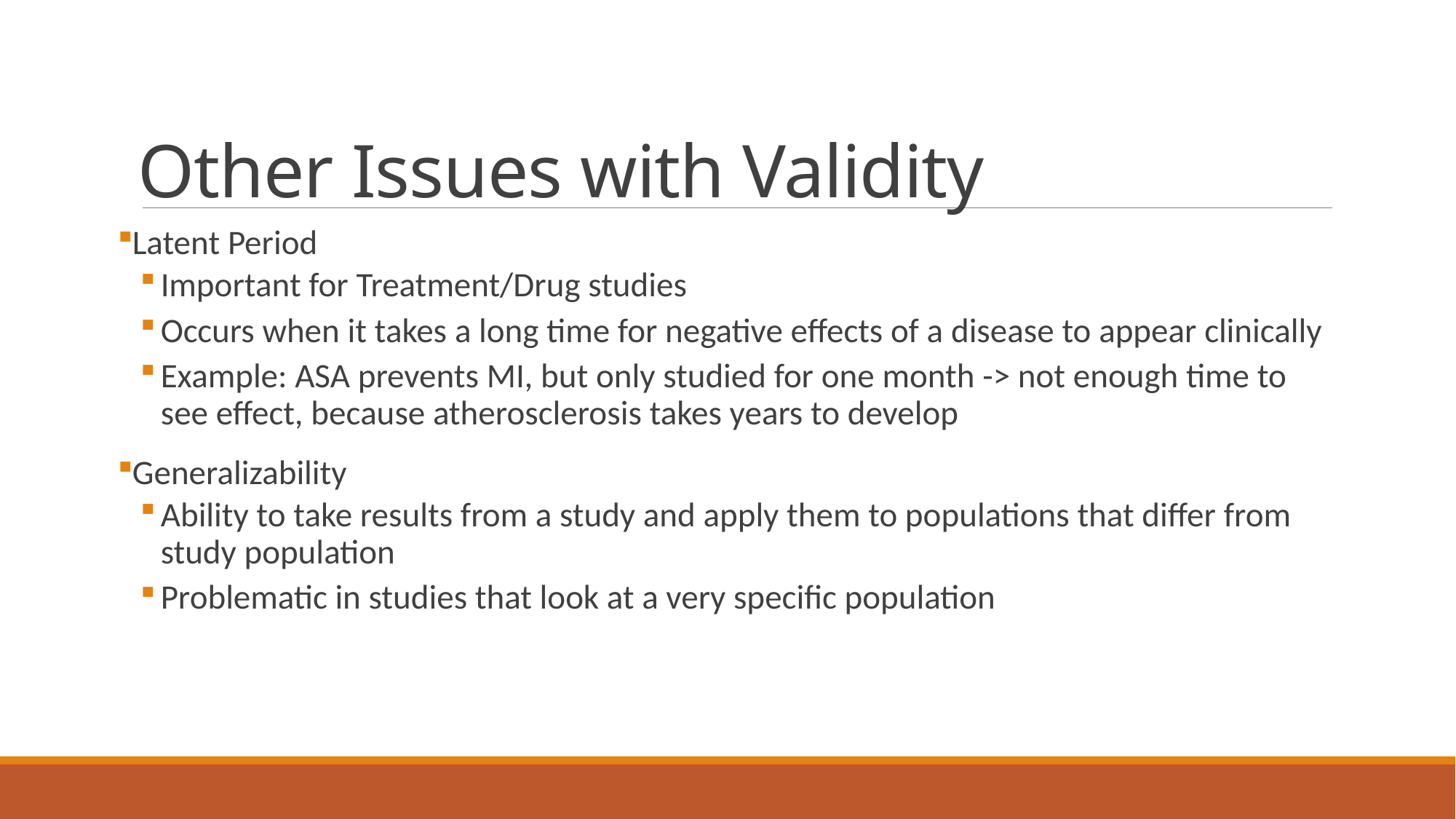

# Other Issues with Validity
Latent Period
Important for Treatment/Drug studies
Occurs when it takes a long time for negative effects of a disease to appear clinically
Example: ASA prevents MI, but only studied for one month -> not enough time to see effect, because atherosclerosis takes years to develop
Generalizability
Ability to take results from a study and apply them to populations that differ from study population
Problematic in studies that look at a very specific population
