# Supplementary material for: Peer-Developed Modules on Basic Biostatistics and Evidence-Based Medicine Principles for Undergraduate Medical Education
Source: MedEdPORTAL. 2020 Nov 24;16:11026. doi: 10.15766/mep_2374-8265.11026 (PMC7703476; doi:10.15766/mep_2374-8265.11026)
Supplement: Supplementary file 1 — Module 1 Study Design and Bias.pptxModule 1 Problem Set.docxModule 1 Problem Set Answer Key.docxModule 1 Formative Quiz.docxModule 1 Formative Quiz Answer Key.docxModule 2 Interpreting Data from Clinical Trials.pptxModule 2 Problem Set.docxModule 2 Problem Set Answer Key.docxModule 2 Formative Quiz.docxModule 2 Formative Quiz Answer Key.docxModule 3 Diagnostic and Therapy Trial Results.pptxModule 3 Problem Set.docxModule 3 Problem Set Answer Key.docxModule 3 Formative Quiz.docxModule 3 Formative Quiz Answer Key.docxImplementation Guide.docxPostsession Evaluation Survey.docx [file mep_2374-8265.11026-s001.zip › H. Module 2 Problem Set Answer Key.docx]

**Module 2 Problem Set Answer Key**

Instructions: Please review the following answers. For incorrect answers, please refer back to module and/or discuss with peers on why each answer was correct or incorrect.

**Anti-CD19 Chimeric Antigen Receptor (CAR) T-cell Therapy**

The following questions have been adapted from: Maude SL, Laetsch TW, Buechner J, et al. Tisagenlecleucel in Children and Young Adults with B-Cell Lymphoblastic Leukemia. *The New England Journal of Medicine.* 2018;378(5):439–48. <https://doi.org/10.1056/NEJMoa1709866>

**Case 1**

Between 2015 and 2017, researchers studied the use of tisagenlecleucel (“Kymriah”), an anti-CD19 chimeric antigen receptor (CAR) T-cell therapy, in patients with relapsed or refractory B-cell acute lymphoblastic leukemia (ALL). The primary endpoint was the overall remission rate, defined either as the rate of complete remission or complete remission with incomplete hematologic recovery, within 3 months. To participate in the study, patients had to be at least 3 years of age at screening and no older than 21 years of age at diagnosis and to have at least 5% lymphoblasts in bone marrow at screening. Patients who had previously received anti-CD19 therapy were excluded. 92 patients were enrolled, of which 75 received an infusion of tisagenlecleucel, whereas the remaining 17 were excluded due to tisagenlecleucel product-related issues, death, or adverse effects.

1. If the CAR-T was designed as a case-control study with a control group of 92 patients, which form of bias would most likely be present in the intervention group?

a) Non-response Bias

**b) Attrition Bias**

c) Procedure Bias

d) Berkson Bias

*Attrition Bias. With 17 patients that were excluded, attrition bias may become an issue. Attrition bias can be defined as an unequal loss of participants in one group compared to another. Participants may withdraw due to unsatisfactory treatment efficacy, intolerable adverse events, or even death. Dropouts can influence the statistical power of the study.*

2. Suppose that while interpreting the results, a biostatistician found that patients at higher education levels exhibited higher rates of remission in the intervention group compared to those within the theoretical control group. Which of the following may be responsible for the results above?

a) Confounding Bias

b) Effect Modification

c) Measurement Bias

**d) Too early to tell – Stratification by Education Level is needed**

*Too early to tell – Stratification by Education Level is Needed. To determine whether the third variable (education level) affects overall remission rate for patients undergoing CAR-T therapy, stratification is first necessary. Stratifying the groups by education level will determine whether it is a confounder or an effect modifier. Confounders merely distort the effect and upon stratification, the difference between groups disappears. By contrast, effect modification does indeed exhibit an effect on the outcome, meaning that upon stratification, the difference between groups remains. As a general tip, be careful with bringing any unconscious assumptions; education level may seem like an obvious confounder, but stratification is first necessary to conclude so.*

3. In addition, the biostatistician above identified geographic location as a confounding variable. To prevent or reduce confounding, what could the researchers have done to improve the study design?

*To prevent or reduce confounding, it is recommended to match patients with similar characteristics in both treatment and control groups. Furthermore, performing multiple/repeated studies or crossover studies, in which patients serve as their own controls, will prevent or reduce confounding bias.*

**Case 2**

The primary endpoint, or the overall remission rate, was assessed by an independent review committee on the basis of the results of laboratory testing of blood, bone marrow, and CSF, as well as physical examination. Patients would be classified as “in complete remission” if there was no sign of cancer. To determine whether the CAR T-cell therapy was effective, the researchers defined what would constitute as a “difference” versus “no difference” in their primary endpoint. “No difference” would be defined as an overall remission rate of only 20% or less, whereas a “difference” would demonstrate an overall remission rate of 45% or higher. In an analysis involving 75 patients who received a tisagenlecleucel (“Kymriah”) infusion and had at least 3 months of follow-up, the overall remission rate was 81% (95% CI, .71 to .89), which was determined to be statistically significant.

1. With the above information, define the null (H_0_) and alternative hypotheses (H_1_) in the context of this study.

*Recall that the null hypothesis would conclude no difference, whereas the alternative hypothesis would conclude that there is a difference. Therefore, in the context of this study:*

*H_0_: Patients with refractory or relapsed B-ALL treated with tisagenlecleucel demonstrate an overall remission rate of 20% or less.*

*H_1_: Patients with refractory or relapsed B-ALL treated with tisagenlecleucel demonstrate an overall remission rate of 45% or higher.*

2. Is the primary endpoint a categorical or quantitative variable?

**a) Categorical**

b) Quantitative

c) Neither

*Categorical. Patients would be categorized as “in complete remission” (versus “not in complete remission”) if they did not exhibit any signs of cancer, as determined by laboratory testing of blood, bone marrow, and CSF, as well as physical examination.*

3. If the 75 patients with relapsed or refractory B-ALL above were compared to patients in two separate and independent studies, which test would be most appropriate to determine whether there is a statistically significant difference between the 3 groups?

a) T-test

b) Analysis of Variance

c) Regression Analysis

**d) Chi-Square**

*Chi-Square. Recall that the primary endpoint is overall remission rate, which is a categorical variable. For categorical variables, a chi-square test can be used to compare 2 or more groups and determine whether a statistically significant difference exists between them.*

4. Suppose that a research group wanted to mimic the CAR-T study with a group of 32 patients, recruited under similar criteria. However, based on their confidence interval, they were unable to find a statistically significant difference within their study. If a difference truly exists, how could they improve their study to find a statistically significant difference?

*Increase power (and thus minimize the risk of a type II error) to improve the likelihood of finding a statistically significant difference if one truly exists. To increase power (and decrease the risk of a type II error), the researchers should increase their sample size and increase their precision of measurement. Increasing expected effect size (i.e. the remission rate of patients) would increase the power, but would be nearly impossible to control.*

5. From the question above, how would such improvements affect the size of their original confidence interval?

*Decrease the size of the confidence interval. Increasing the power of the study would involve increasing the sample size. According to the formula for confidence intervals,*

*Confidence Interval = Mean ± Z * 𝜎*

*√n*

*n would indicate the sample size. Therefore, increasing n would lead to a smaller confidence interval.*

**Case 3**

During the CAR-T therapy study, researchers followed 75 patients over approximately 2 years and monitored their response to tisagenlecleucel (“Kymriah”). No control group was established. In addition to finding higher overall remission rates, researchers also found that cytokine release syndrome, characterized by difficulty breathing, dizziness, hypotension, and fever, occurred in 88% of patients and was effectively managed with supportive measures and anticytokine therapy. Furthermore, it was found that neurologic adverse events appeared to be more frequent in patients with cytokine release syndrome, especially in those with higher-grade symptoms. In spite of these side effects and given the efficacy of the CAR-T therapy, tisagenlecleucel was approved by the FDA in July 2017 for treatment of patients with refractory or relapsed B-ALL.

1. Which type of study would the CAR-T therapy be characterized as?

a) Prospective cohort study

b) Case-control study

c) Cross-sectional observation study

**d) Uncontrolled clinical trial**

*Uncontrolled clinical trial. In the CAR-T therapy trial, the researchers are administering an intervention (tisagenlecleucel) to all 75 patients and monitoring their response over ~2 years. Note that no control group was utilized.*

*Note that the CAR-T therapy paper does mention the use of a single-cohort; however, in the context of the study, a cohort is defined as “a group of people treated together.” Compare this with a cohort study, in which we are generally following people based on presence or absence of an unassigned exposure – not an intervention.*

2. Suppose that in a separate cohort study, researchers evaluated neurologic adverse events as a consequence of patients who developed cytokine release syndrome (CRS) from CAR-T therapy. Among 256 patients with B-ALL, 126 patients with CRS had developed seizures, 34 patients without CRS had developed seizures, 59 patients with CRS did not develop seizures, and 37 patients without CRS did not develop seizures. Which of the following is true?

a) The relative risk of developing seizures in those with CRS is 2.32.

b) The odds ratio of developing seizures in those with CRS is 2.32.

**c) The relative risk of developing seizures in those with CRS is 1.42.**

d) The odds ratio of developing seizures in those with CRS is 1.42.

*The relative risk of developing seizures in those with CRS is 1.42.*

*2 x 2 table:*

|  | *Seizures* | *No Seizures* |
| --- | --- | --- |
| *CRS* | *126* | *59* |
| *No CRS* | *34* | *37* |

*In a cohort study, relative risk would be most appropriate to assess risk.*

*Relative risk = (A / (A + B)) = 126/185 = 1.42.*

*(C / (C + D)) 34/71*

3. In the study above, it was determined that the researchers had committed egregious errors. Of the following, which may compromise the validity of the study? Choose all that apply.

**a) Inconsistent follow up to determine whether an adverse neurologic event has occurred**

**b) Failure to report patients who had dropped out of the study**

c) Stratifying patients by demographics to identify and eliminate confounding bias

d) Failure to inform neurologists which patients developed cytokine release syndrome

**e) Sponsorship by a pharmaceutical group that develops antiseizure medication**

*Inconsistent follow up to determine whether an adverse neurologic event has occurred – it is best to follow up consistently with patients within a given timeframe to determine whether an outcome of interest (e.g. adverse neurologic event) has occurred. Poor follow up would lead to inconsistent and unreliable data.*

*Failure to report patients who had dropped out from the study – all dropouts must be accounted for and reported with accompanying reasons for dropout. For example, dropouts due to an adverse drug-related event must be reported for safety analysis. Failure to report dropouts may raise questions whether the researchers are hiding information deliberately in their favor.*

*Sponsorship by a pharmaceutical group that develops antiseizure medication – sponsorship MAY compromise the validity, especially if the study has been funded by a group with conflicts of interest (e.g. financial gain). All studies should disclose any possible conflicts of interest. However, it is also possible that underlying motives may not be involved. Regardless, it is important to be wary and critical.*

*Note that failure to inform researchers which patients developed cytokine release syndrome does NOT compromise validity. This could otherwise be interpreted as blinding, which would benefit the study by preventing any potential bias (e.g. procedure bias, observer-expectancy bias, etc.).*
