# Supplementary material for: Peer-Developed Modules on Basic Biostatistics and Evidence-Based Medicine Principles for Undergraduate Medical Education
Source: MedEdPORTAL. 2020 Nov 24;16:11026. doi: 10.15766/mep_2374-8265.11026 (PMC7703476; doi:10.15766/mep_2374-8265.11026)
Supplement: Supplementary file 1 — Module 1 Study Design and Bias.pptxModule 1 Problem Set.docxModule 1 Problem Set Answer Key.docxModule 1 Formative Quiz.docxModule 1 Formative Quiz Answer Key.docxModule 2 Interpreting Data from Clinical Trials.pptxModule 2 Problem Set.docxModule 2 Problem Set Answer Key.docxModule 2 Formative Quiz.docxModule 2 Formative Quiz Answer Key.docxModule 3 Diagnostic and Therapy Trial Results.pptxModule 3 Problem Set.docxModule 3 Problem Set Answer Key.docxModule 3 Formative Quiz.docxModule 3 Formative Quiz Answer Key.docxImplementation Guide.docxPostsession Evaluation Survey.docx [file mep_2374-8265.11026-s001.zip › I. Module 2 Formative Quiz.docx]

**Module 2 Formative Quiz**

Instructions: Please complete the following quiz individually, closed-book, and within 10 minutes to assess your knowledge and understanding of the Module 2 material.

1. An investigator is studying physician behavior at 3 different primary care offices. She conducts a study to see how often physicians sit down when conducting the patient interview. Throughout the duration of the study, the investigator sends a member of her team to each location who explains the study to each patient and asks them to pay attention to whether the physician sits down or stands during the interview. After their visit, the patients fill out a survey. At the end of the study, the results show that there was a dramatic increase in the frequency of the physicians sitting down during the interview about 3 days after the study began.

What is most likely to explain the reason for the increase in physician sitting down during the interview?

1. Volunteer Bias
2. Recall Bias
3. Procedure Bias
4. Hawthorne Effect
5. Observer-Expectancy Bias

2. A study investigates the association between sun exposure and melanoma. The study enrolls 20 participants who have melanoma and surveys them via telephone regarding their previous sun exposure. 20 participants who do not have a history of melanoma are also surveyed and are asked about their previous sun exposure. The study finds that there is no significant association between sun exposure and incidence of melanoma (RR = 0.99, p = 0.05).

Which of the following is most likely causing the findings?

1. Hawthorne Effect
2. Observer-Expectancy Bias
3. Pygmalion Effect
4. Late-Look Bias
5. Sample Size

3. Researchers conduct a study examining the rate of transient aplastic crisis in patients with sickle cell disease after exposure to parvovirus B19. A group of 200 sickle cell patients that meet criteria for aplastic anemia (Hb <10, reticulocyte count <2.5%) and 200 sickle cell patients that do not meet criteria for aplastic anemia are subsequently tested for infection with parvovirus B19 using PCR. The results of the test are shown below.

|  | Aplastic anemia | No aplastic anemia | Total |
| --- | --- | --- | --- |
| Positive B19 PCR | 135 | 75 | 210 |
| Negative B19  PCR | 65 | 125 | 190 |
| Total | 200 | 200 |  |

What is the relative risk of acquiring aplastic anemia after exposure to parvovirus B19?

1. 3.46 times as likely
2. 1.87 times as likely
3. .53 times as likely
4. 1.10 times as likely

4. A study investigates the effects the effects of hypertension on renal function. 298 patients without hypertension (BP<130/80), 300 patients with stage 1 hypertension (BP>130/80) and 300 patients with stage 2 hypertension (BP>140/90) are examined and have blood samples taken for baseline creatinine measurements. These patients are then followed for 5 years to evaluate for impaired renal function (creatinine level >2). What is the best way to assess whether is a statistically significant difference between the groups in this study?

1. T-test
2. ANOVA test
3. Relative Risk
4. Chi-Square Test

5. An experimenter investigates the effects of a new aromatase inhibitor on recurrence rates of ER+/PR+ breast cancer. At the end of the study, it is determined that there is a statistically significant (p=.049) difference between these group receiving the drug therapy vs standard therapy despite numerous prior studies failing to detect a statistically significant difference in the past. Upon careful analysis of the article, you find that this study has been entirely funded by the pharmaceutical company responsible for development of this drug. What is the most appropriate interpretation of this article?

1. Disregard the results entirely
2. Decide whether to use this drug with your patients only after there has been sufficient evidence from non-partisan sources that this drug has a tolerable risk-benefit profile.
3. Write an angry letter to the journal publishing this article admonishing the author for accepting big pharma money
4. Adopt the treatment for use in your clinical practice
