# Supplementary material for: Peer-Developed Modules on Basic Biostatistics and Evidence-Based Medicine Principles for Undergraduate Medical Education
Source: MedEdPORTAL. 2020 Nov 24;16:11026. doi: 10.15766/mep_2374-8265.11026 (PMC7703476; doi:10.15766/mep_2374-8265.11026)
Supplement: Supplementary file 1 — Module 1 Study Design and Bias.pptxModule 1 Problem Set.docxModule 1 Problem Set Answer Key.docxModule 1 Formative Quiz.docxModule 1 Formative Quiz Answer Key.docxModule 2 Interpreting Data from Clinical Trials.pptxModule 2 Problem Set.docxModule 2 Problem Set Answer Key.docxModule 2 Formative Quiz.docxModule 2 Formative Quiz Answer Key.docxModule 3 Diagnostic and Therapy Trial Results.pptxModule 3 Problem Set.docxModule 3 Problem Set Answer Key.docxModule 3 Formative Quiz.docxModule 3 Formative Quiz Answer Key.docxImplementation Guide.docxPostsession Evaluation Survey.docx [file mep_2374-8265.11026-s001.zip › K. Module 3 Diagnostic and Therapy Trial Results.pptx]

## Slide 1
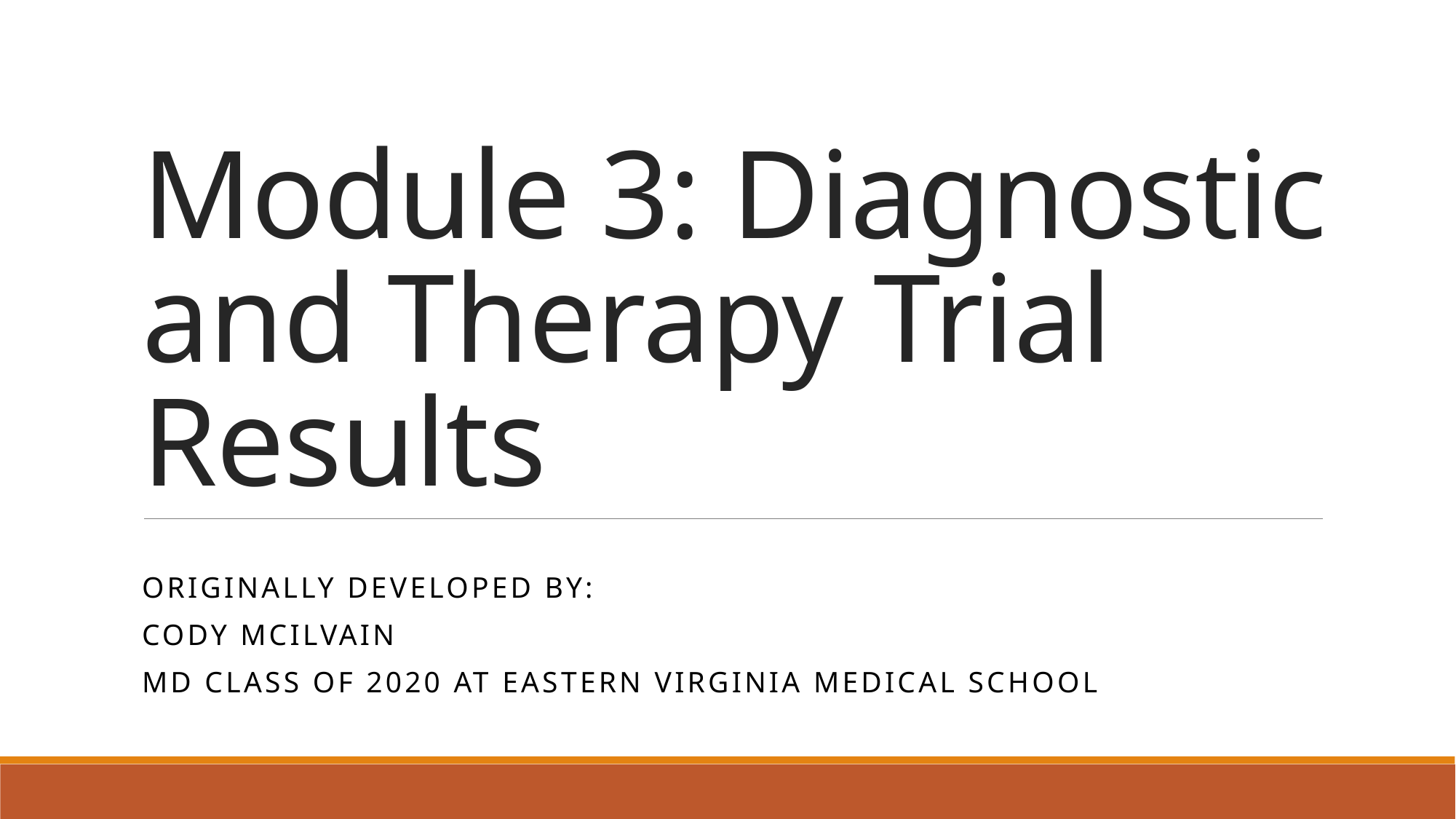

# Module 3: Diagnostic and Therapy Trial Results
Originally developed by:
cODY mCilvain
Md cLASS OF 2020 aT eASTERN vIRGINIA mEDICAL sCHOOL

## Slide 2
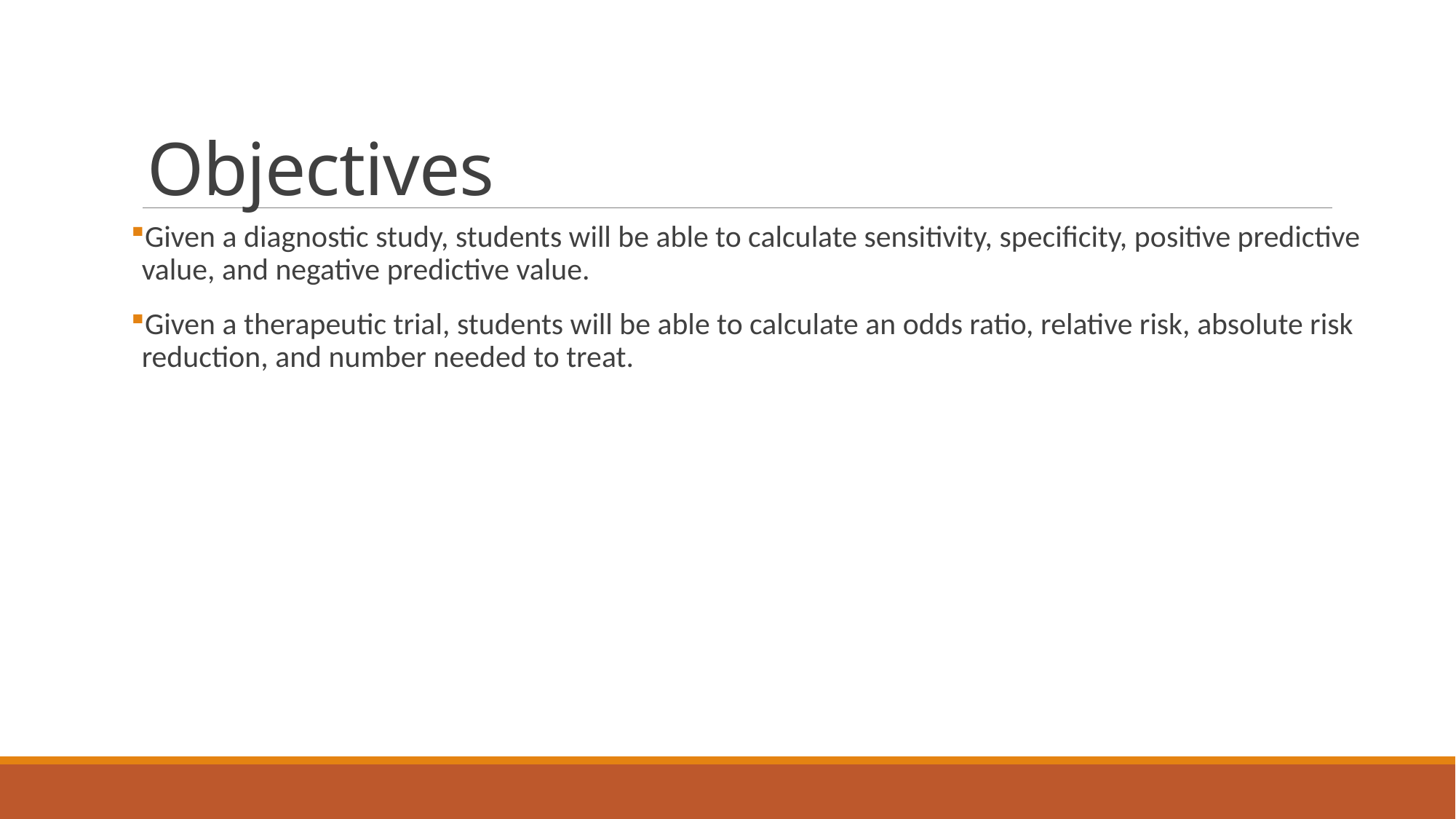

# Objectives
Given a diagnostic study, students will be able to calculate sensitivity, specificity, positive predictive value, and negative predictive value.
Given a therapeutic trial, students will be able to calculate an odds ratio, relative risk, absolute risk reduction, and number needed to treat.

## Slide 3
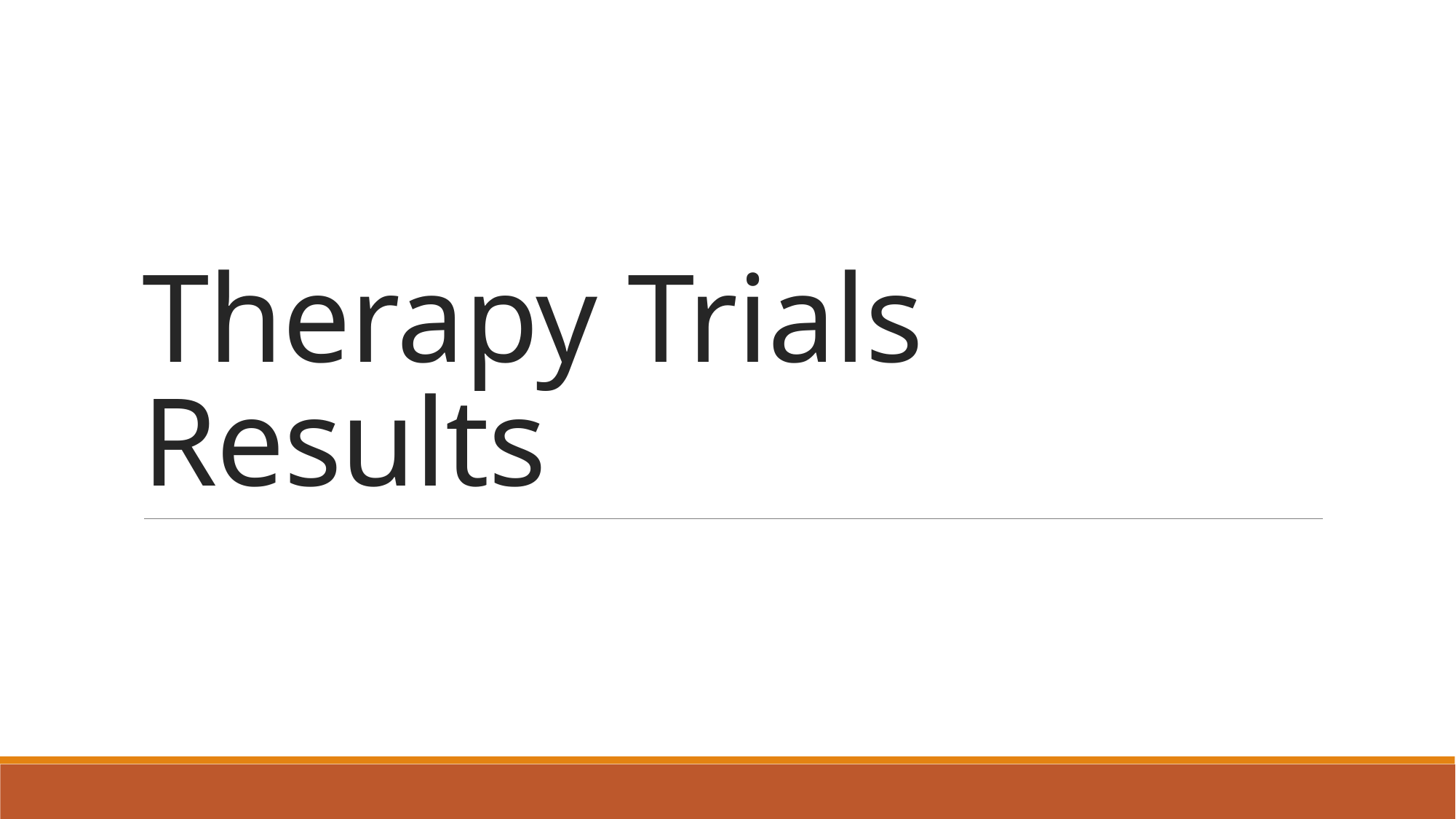

# Therapy Trials Results

## Slide 4
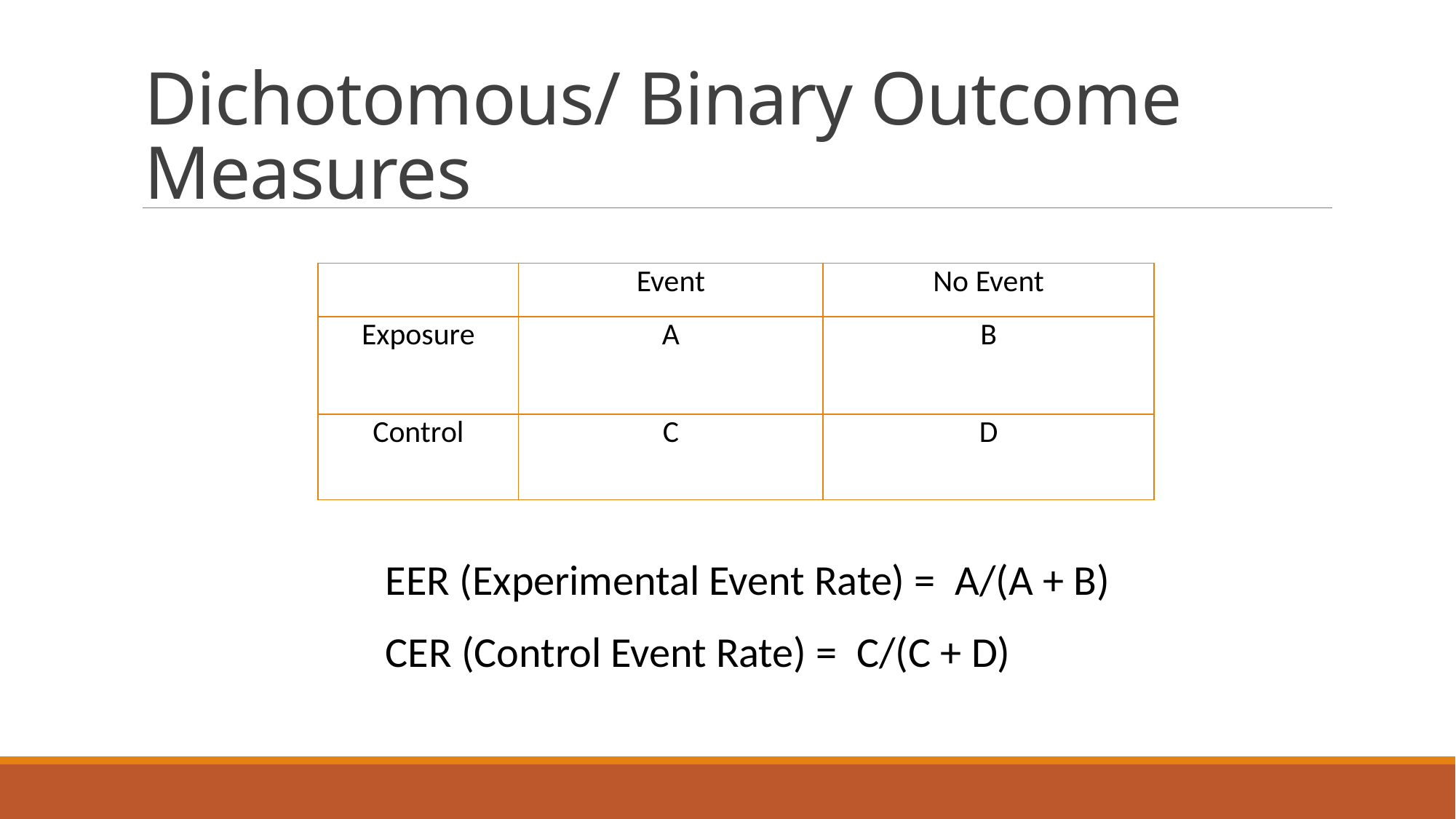

# Dichotomous/ Binary Outcome Measures
| | Event | No Event |
| --- | --- | --- |
| Exposure | A | B |
| Control | C | D |
EER (Experimental Event Rate) = A/(A + B)
CER (Control Event Rate) = C/(C + D)

## Slide 5
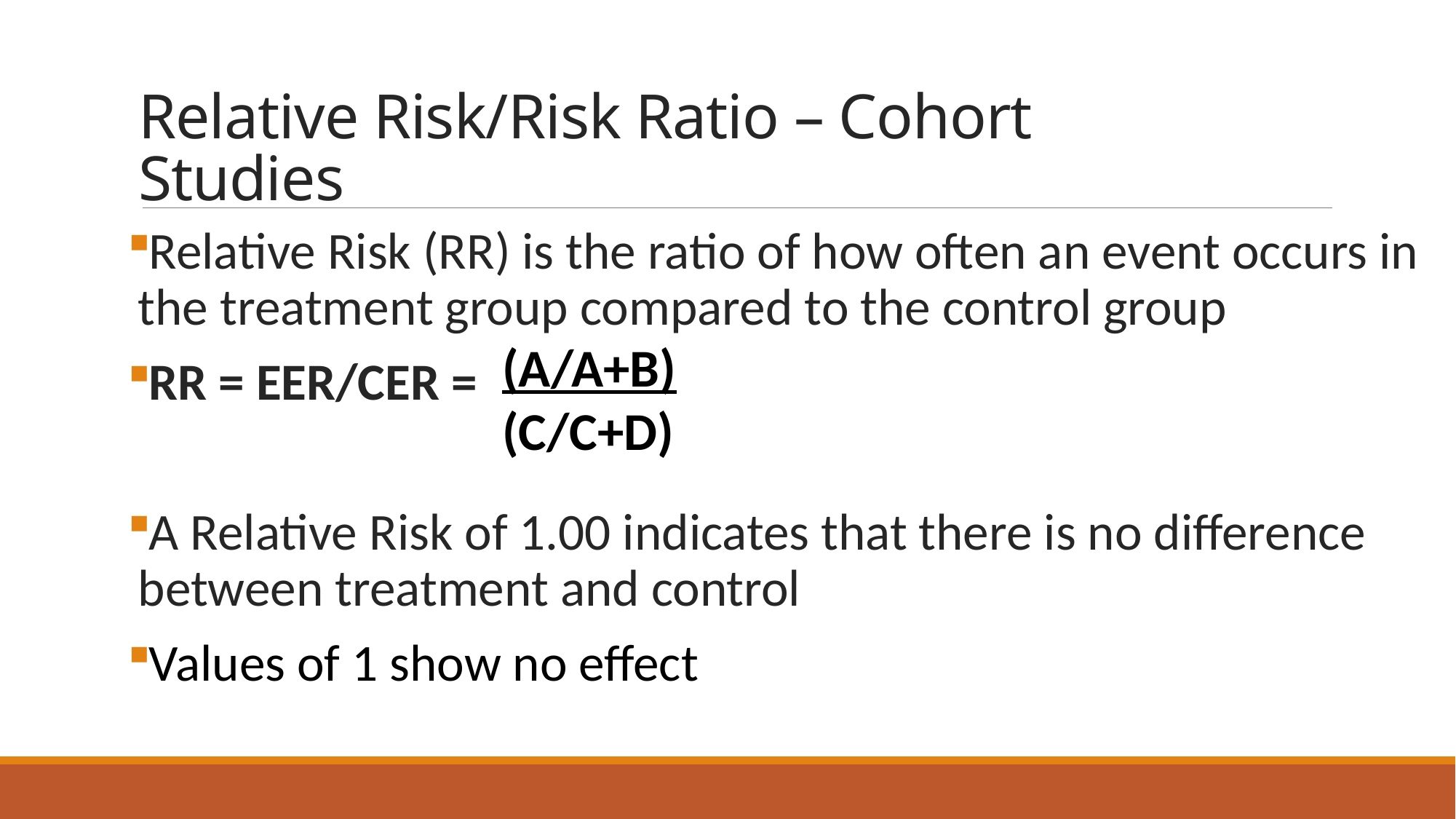

# Relative Risk/Risk Ratio – Cohort Studies
Relative Risk (RR) is the ratio of how often an event occurs in the treatment group compared to the control group
RR = EER/CER =
A Relative Risk of 1.00 indicates that there is no difference between treatment and control
Values of 1 show no effect
(A/A+B)
(C/C+D)

## Slide 6
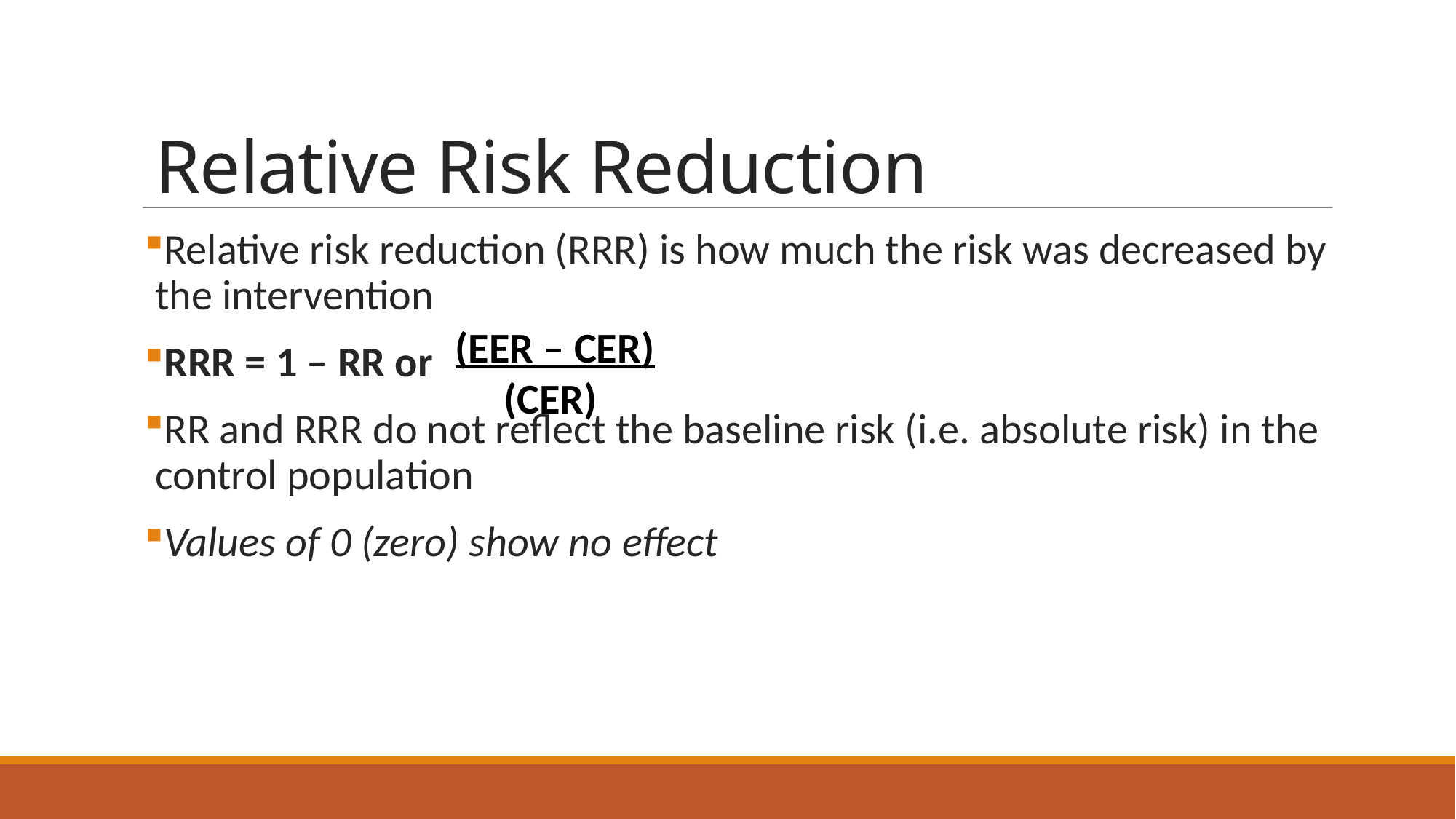

# Relative Risk Reduction
Relative risk reduction (RRR) is how much the risk was decreased by the intervention
RRR = 1 – RR or
RR and RRR do not reflect the baseline risk (i.e. absolute risk) in the control population
Values of 0 (zero) show no effect
(EER – CER)
 (CER)

## Slide 7
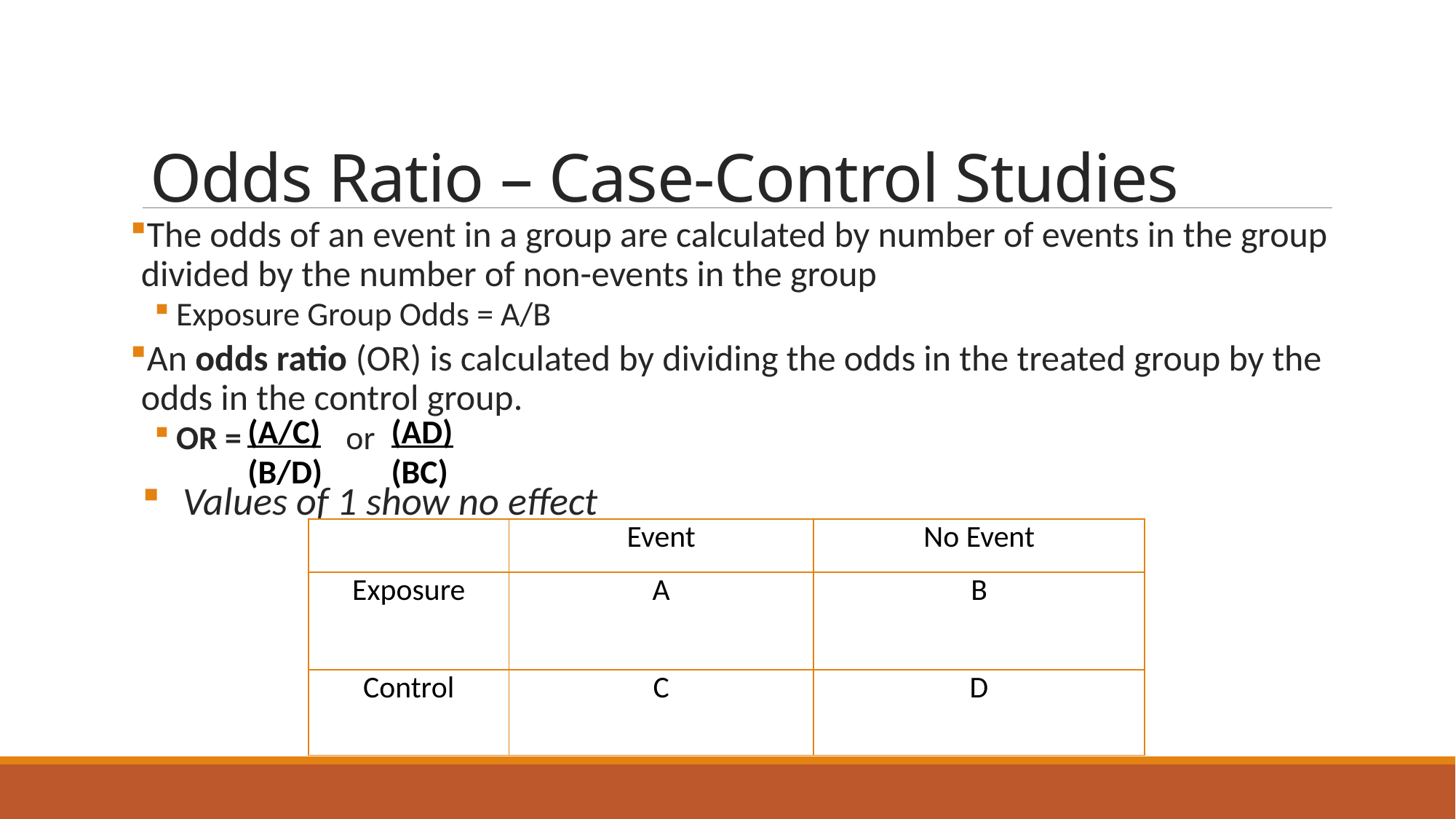

# Odds Ratio – Case-Control Studies
The odds of an event in a group are calculated by number of events in the group divided by the number of non-events in the group
Exposure Group Odds = A/B
An odds ratio (OR) is calculated by dividing the odds in the treated group by the odds in the control group.
OR = or
Values of 1 show no effect
(AD)
(BC)
(A/C)
(B/D)
| | Event | No Event |
| --- | --- | --- |
| Exposure | A | B |
| Control | C | D |

## Slide 8
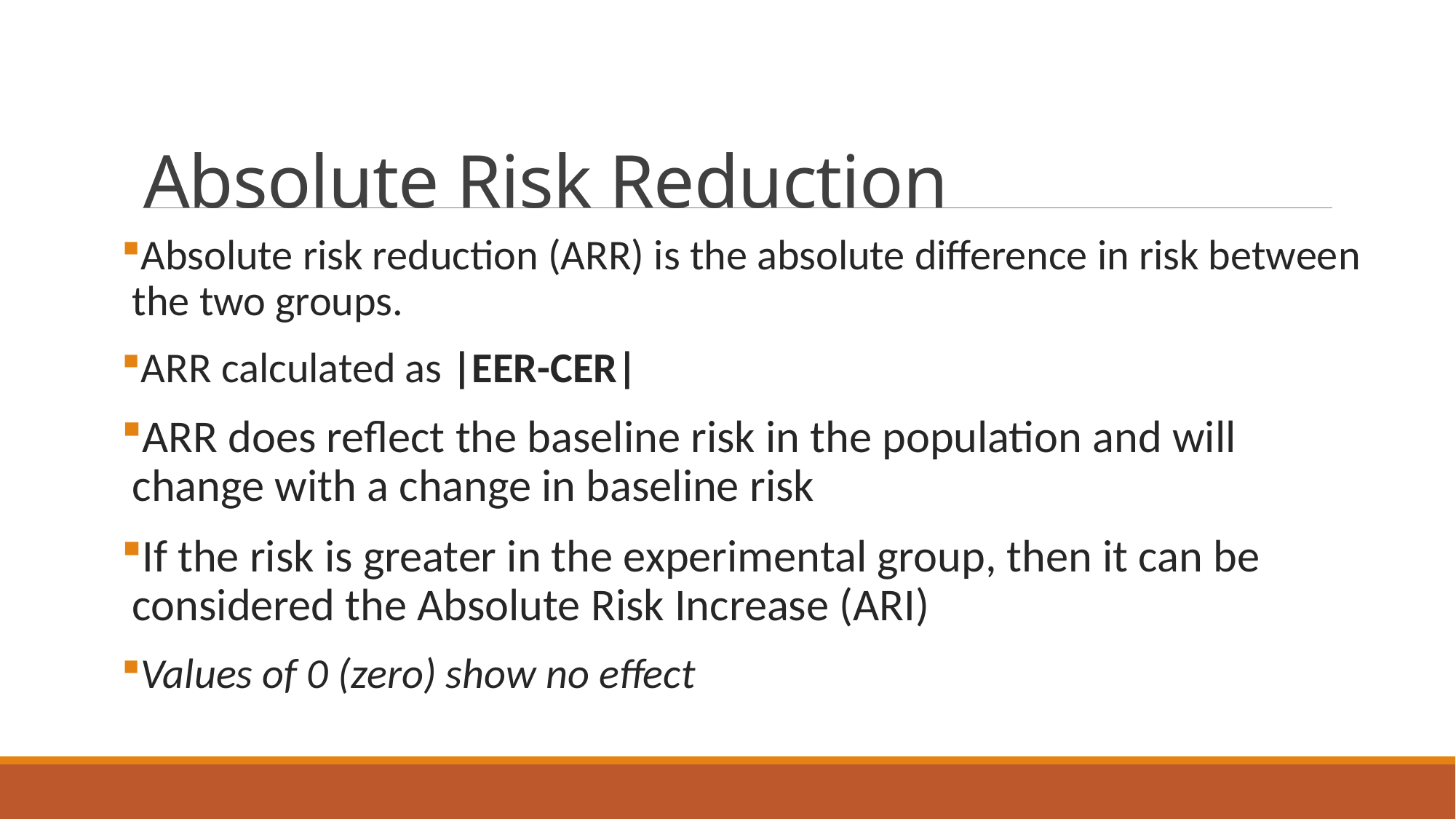

# Absolute Risk Reduction
Absolute risk reduction (ARR) is the absolute difference in risk between the two groups.
ARR calculated as |EER-CER|
ARR does reflect the baseline risk in the population and will change with a change in baseline risk
If the risk is greater in the experimental group, then it can be considered the Absolute Risk Increase (ARI)
Values of 0 (zero) show no effect

## Slide 9
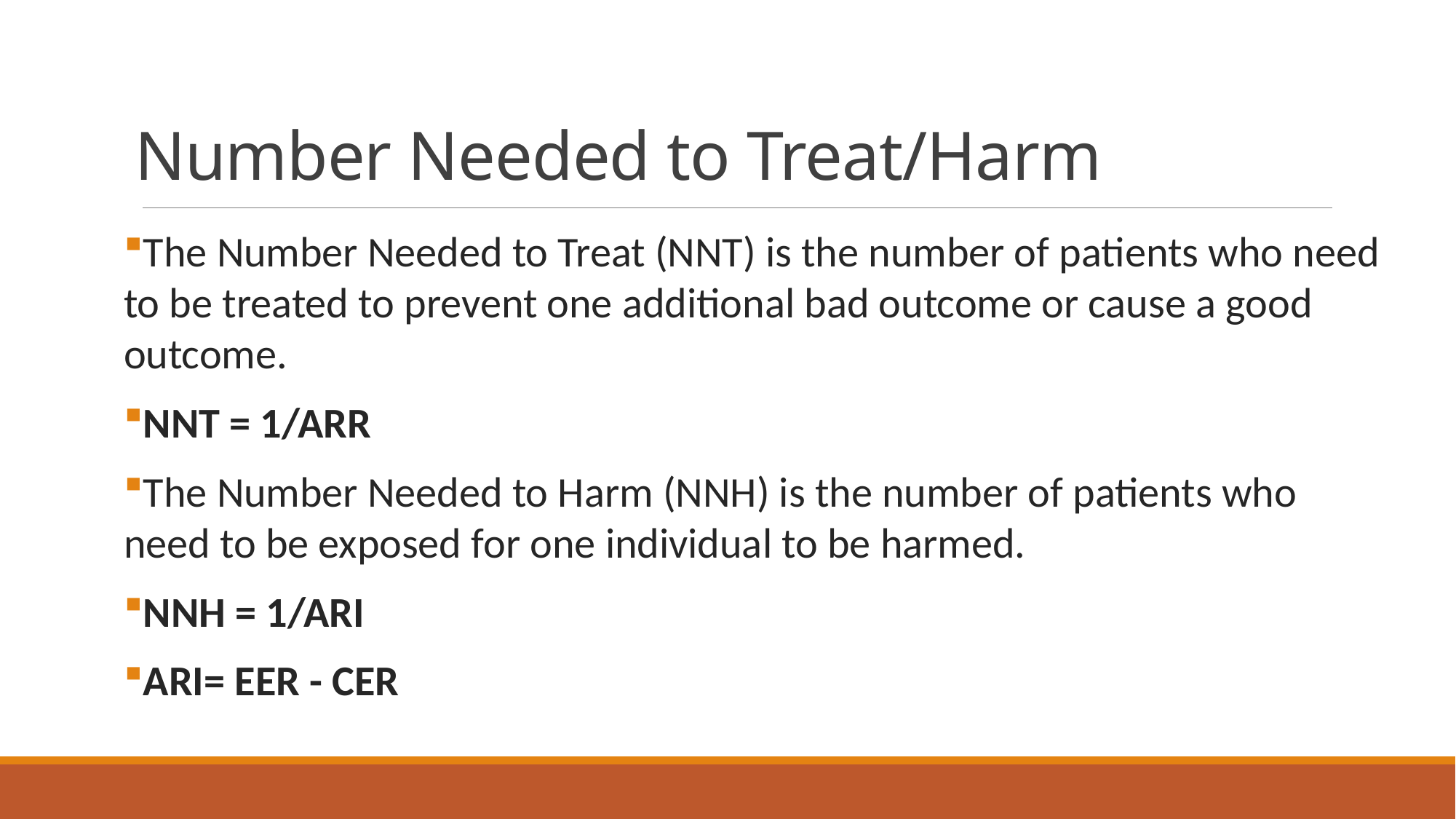

# Number Needed to Treat/Harm
The Number Needed to Treat (NNT) is the number of patients who need to be treated to prevent one additional bad outcome or cause a good outcome.
NNT = 1/ARR
The Number Needed to Harm (NNH) is the number of patients who need to be exposed for one individual to be harmed.
NNH = 1/ARI
ARI= EER - CER

## Slide 10
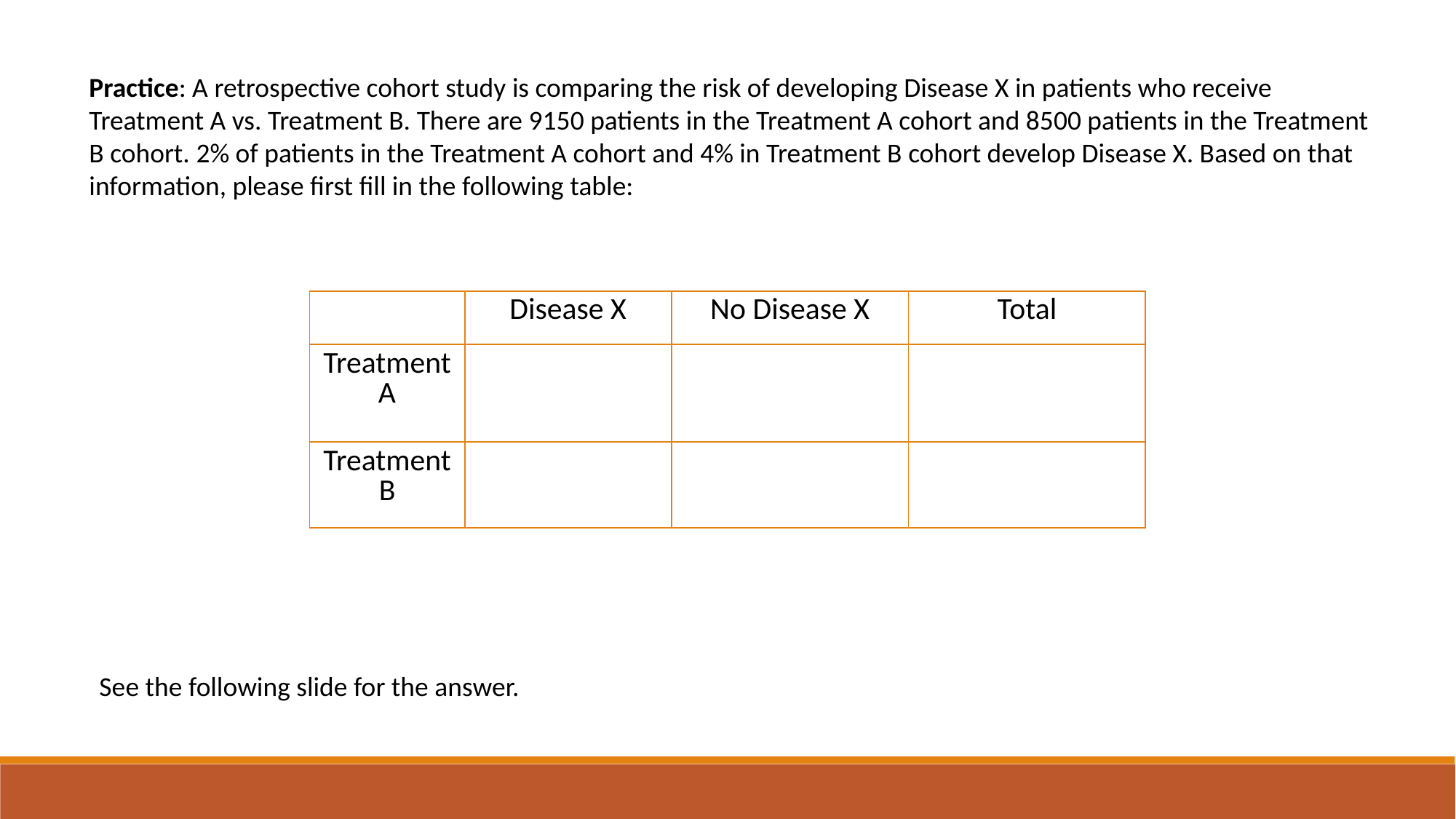

Practice: A retrospective cohort study is comparing the risk of developing Disease X in patients who receive Treatment A vs. Treatment B. There are 9150 patients in the Treatment A cohort and 8500 patients in the Treatment B cohort. 2% of patients in the Treatment A cohort and 4% in Treatment B cohort develop Disease X. Based on that information, please first fill in the following table:
| | Disease X | No Disease X | Total |
| --- | --- | --- | --- |
| Treatment A | | | |
| Treatment B | | | |
See the following slide for the answer.

## Slide 11
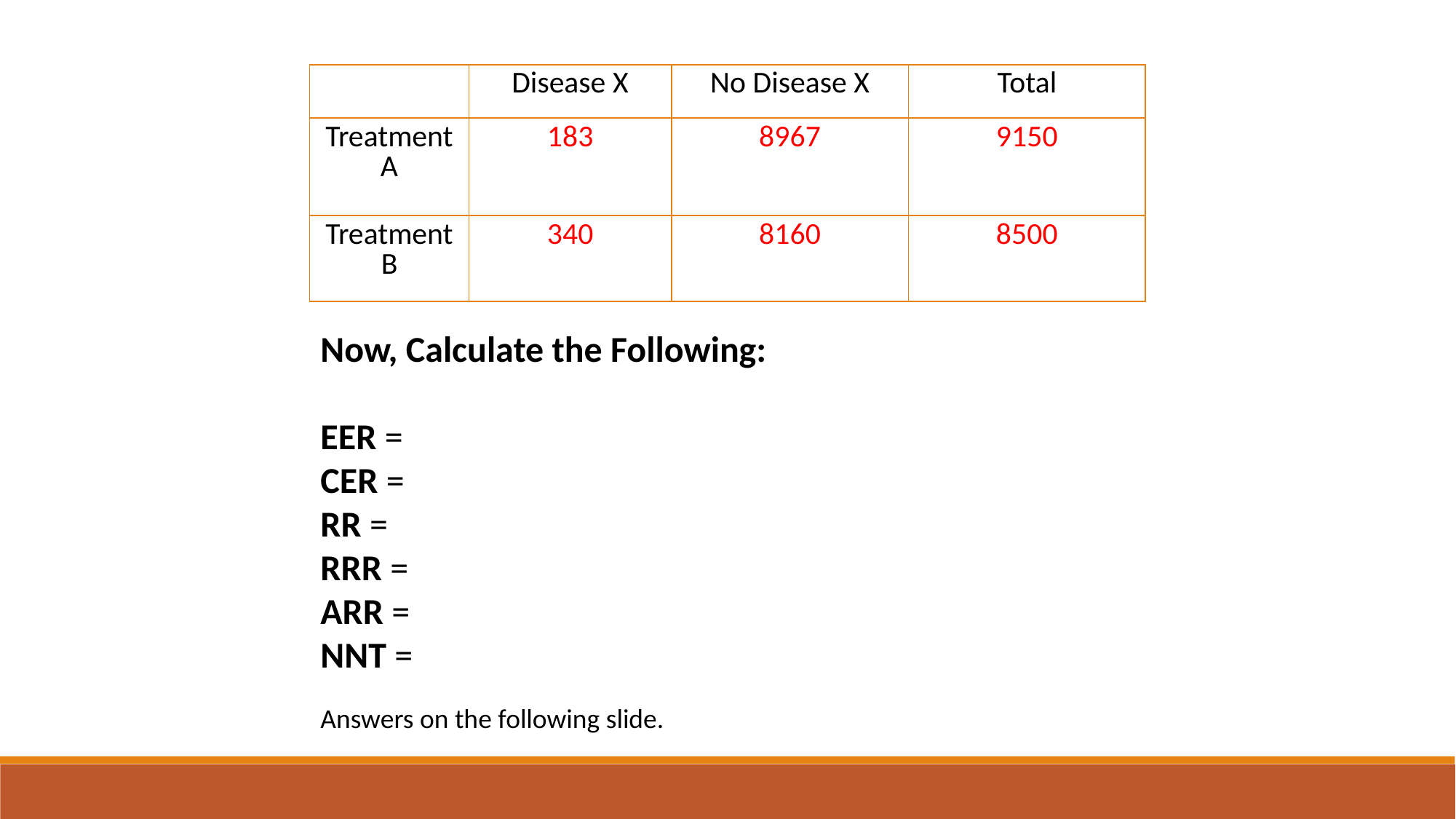

| | Disease X | No Disease X | Total |
| --- | --- | --- | --- |
| Treatment A | 183 | 8967 | 9150 |
| Treatment B | 340 | 8160 | 8500 |
Now, Calculate the Following:
EER =
CER =
RR =
RRR =
ARR =
NNT =
Answers on the following slide.

## Slide 12
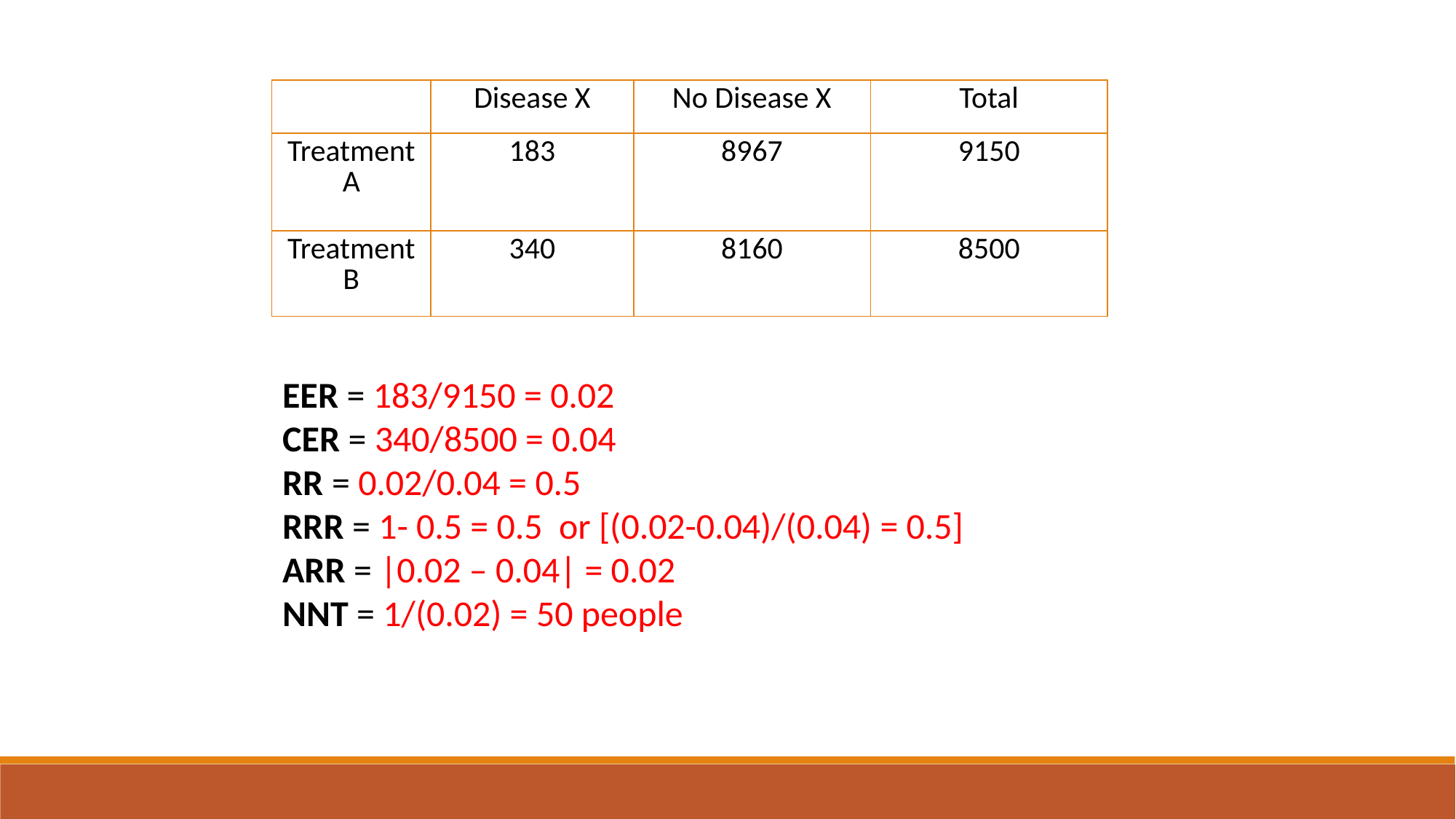

| | Disease X | No Disease X | Total |
| --- | --- | --- | --- |
| Treatment A | 183 | 8967 | 9150 |
| Treatment B | 340 | 8160 | 8500 |
EER = 183/9150 = 0.02
CER = 340/8500 = 0.04
RR = 0.02/0.04 = 0.5
RRR = 1- 0.5 = 0.5 or [(0.02-0.04)/(0.04) = 0.5]
ARR = |0.02 – 0.04| = 0.02
NNT = 1/(0.02) = 50 people

## Slide 13
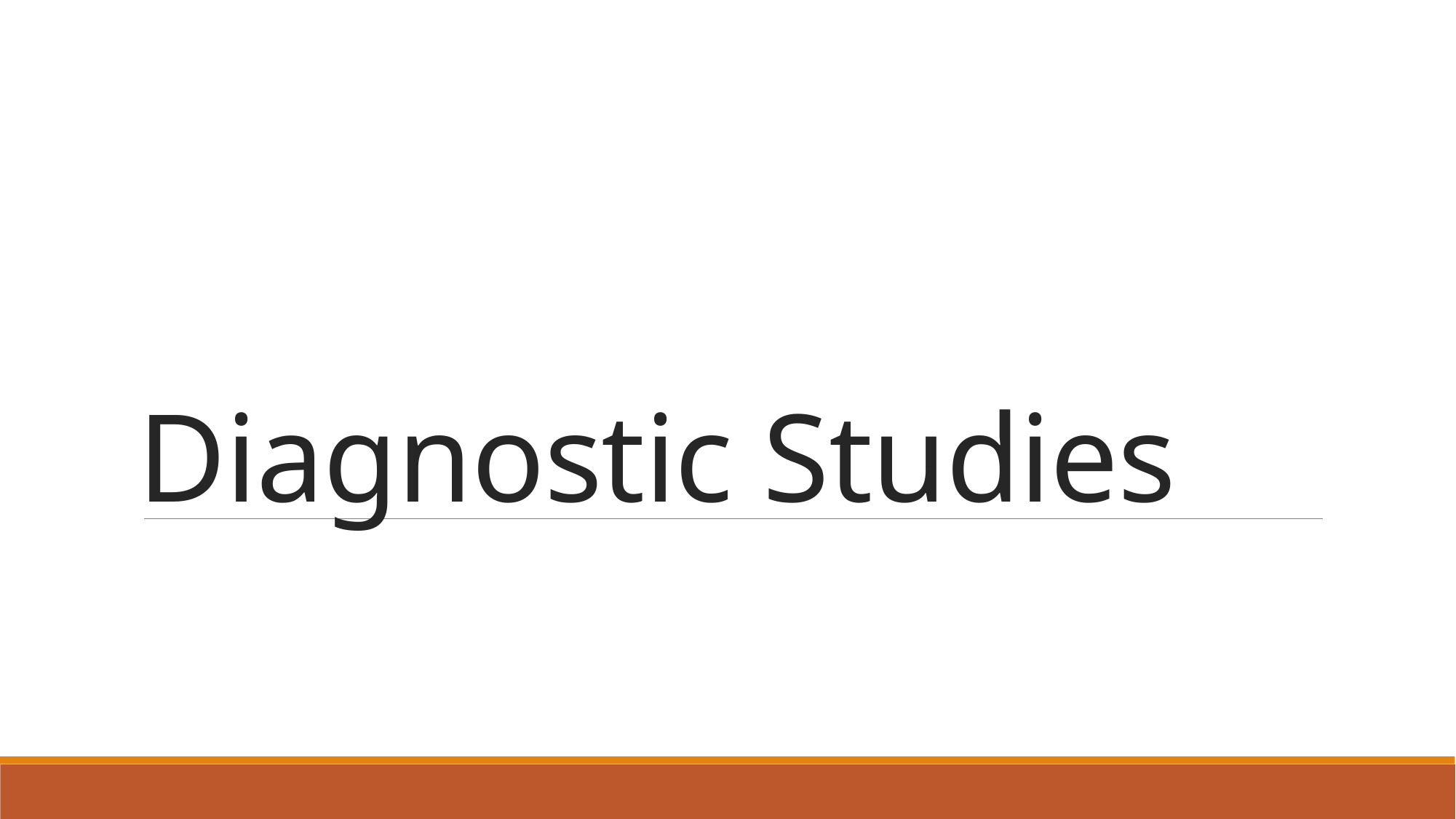

# Diagnostic Studies

## Slide 14
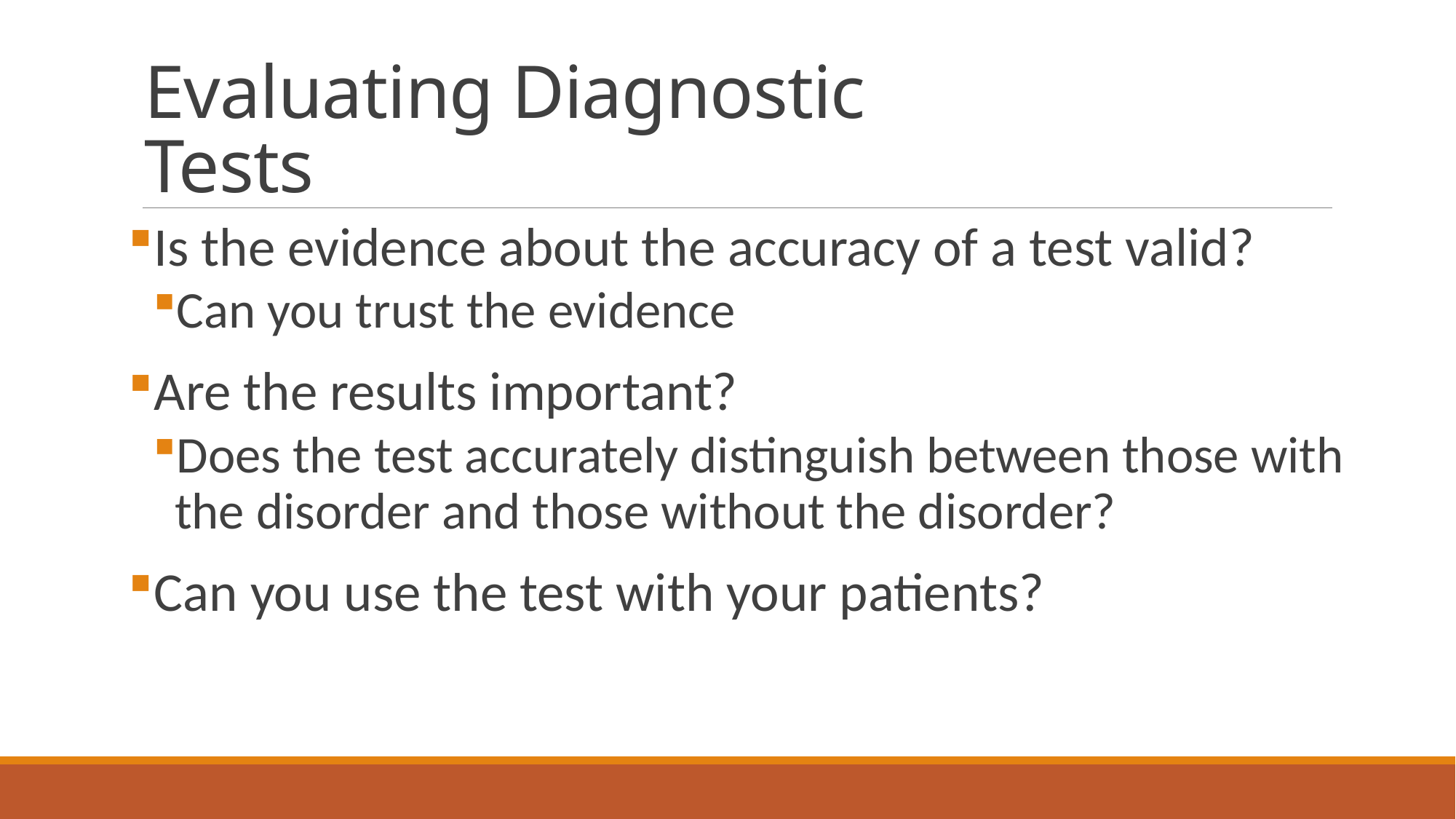

# Evaluating Diagnostic Tests
Is the evidence about the accuracy of a test valid?
Can you trust the evidence?
Are the results important?
Does the test accurately distinguish between those with the disorder and those without the disorder?
Can you use the test with your patients?

## Slide 15
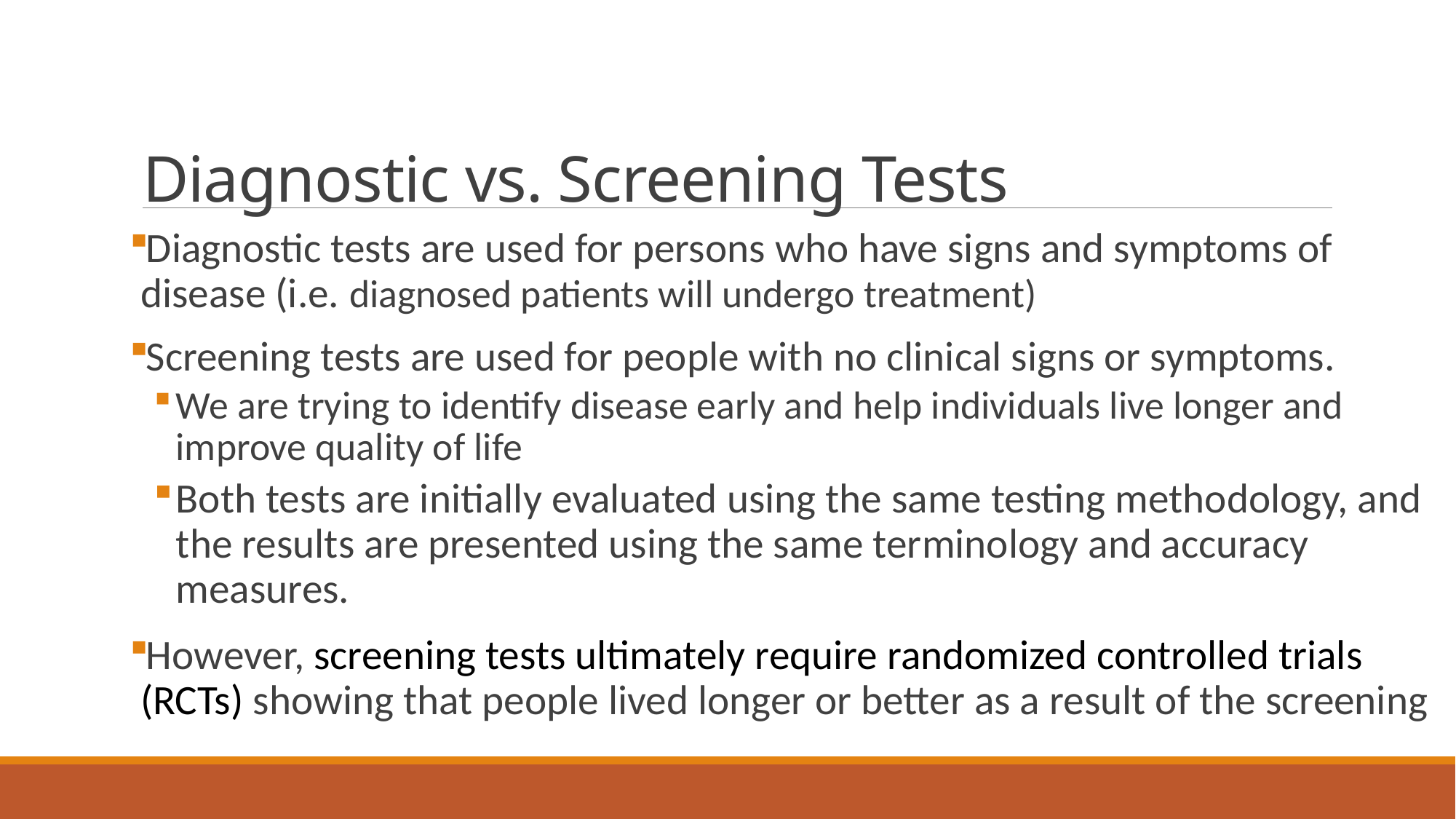

# Diagnostic vs. Screening Tests
Diagnostic tests are used for persons who have signs and symptoms of disease (i.e. diagnosed patients will undergo treatment)
Screening tests are used for people with no clinical signs or symptoms.
We are trying to identify disease early and help individuals live longer and improve quality of life
Both tests are initially evaluated using the same testing methodology, and the results are presented using the same terminology and accuracy measures.
However, screening tests ultimately require randomized controlled trials (RCTs) showing that people lived longer or better as a result of the screening

## Slide 16
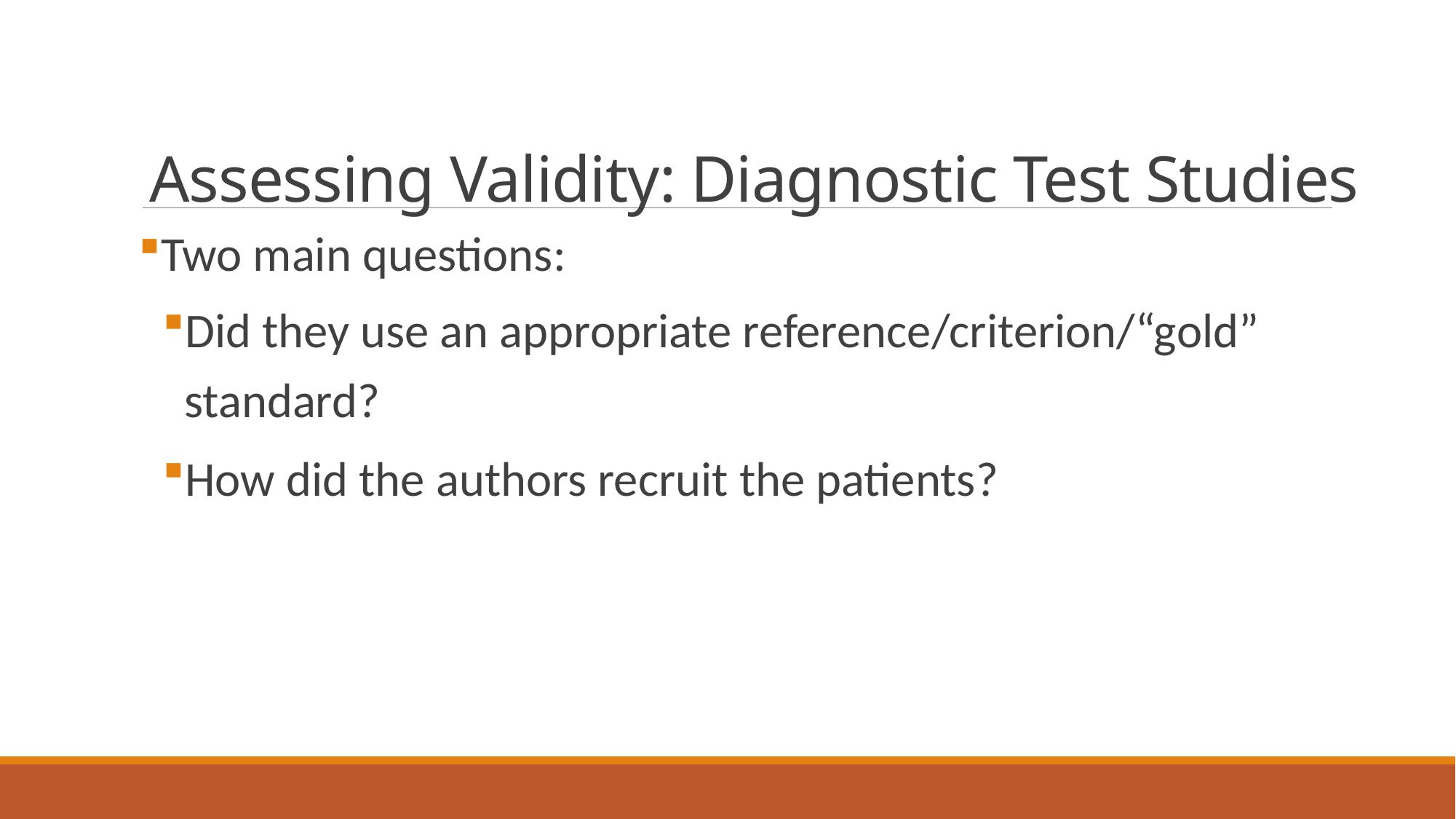

# Assessing Validity: Diagnostic Test Studies
Two main questions:
Did they use an appropriate reference/criterion/“gold” standard?
How did the authors recruit the patients?

## Slide 17
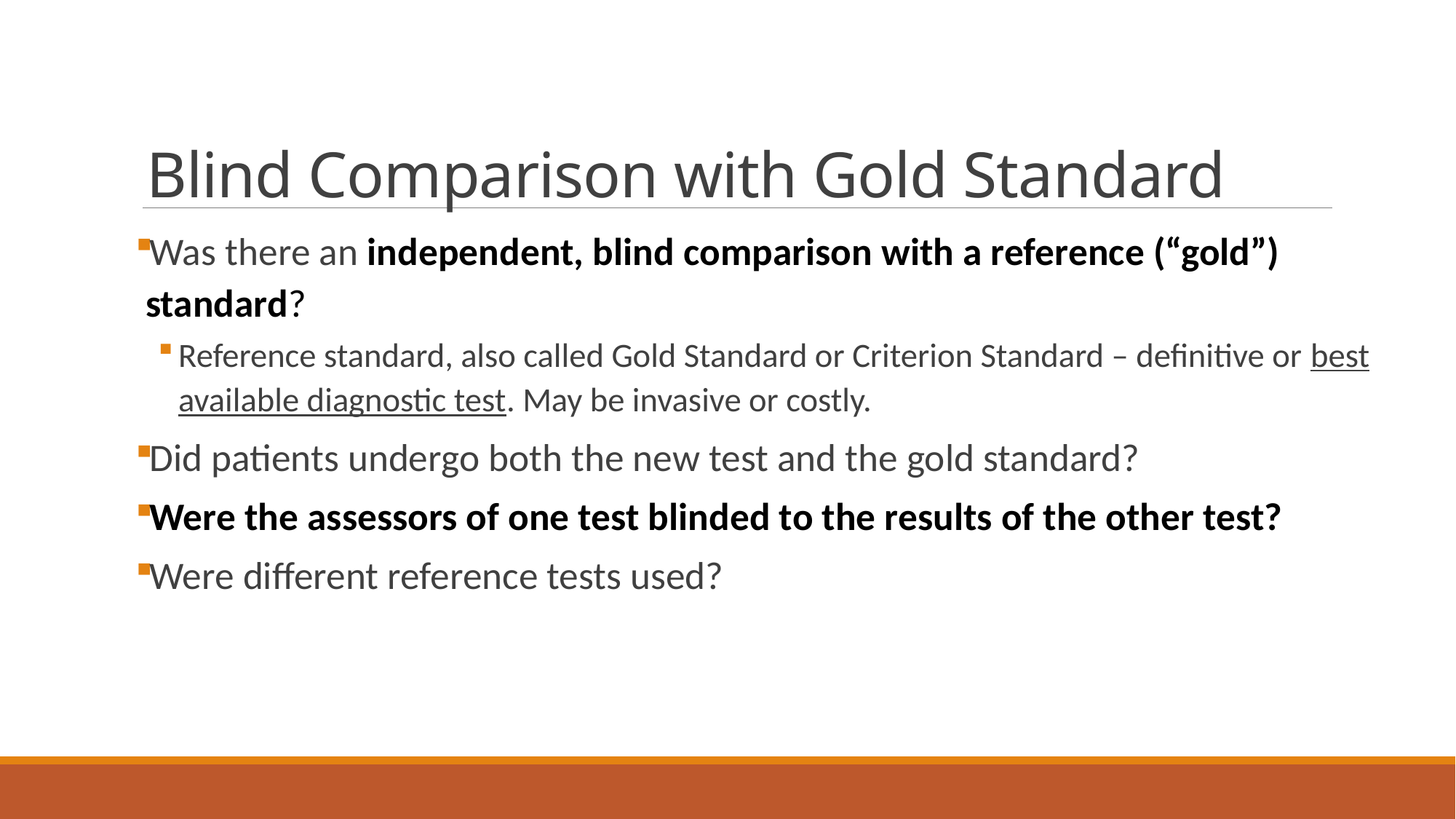

# Blind Comparison with Gold Standard
Was there an independent, blind comparison with a reference (“gold”) standard?
Reference standard, also called Gold Standard or Criterion Standard – definitive or best available diagnostic test. May be invasive or costly.
Did patients undergo both the new test and the gold standard?
Were the assessors of one test blinded to the results of the other test?
Were different reference tests used?

## Slide 18
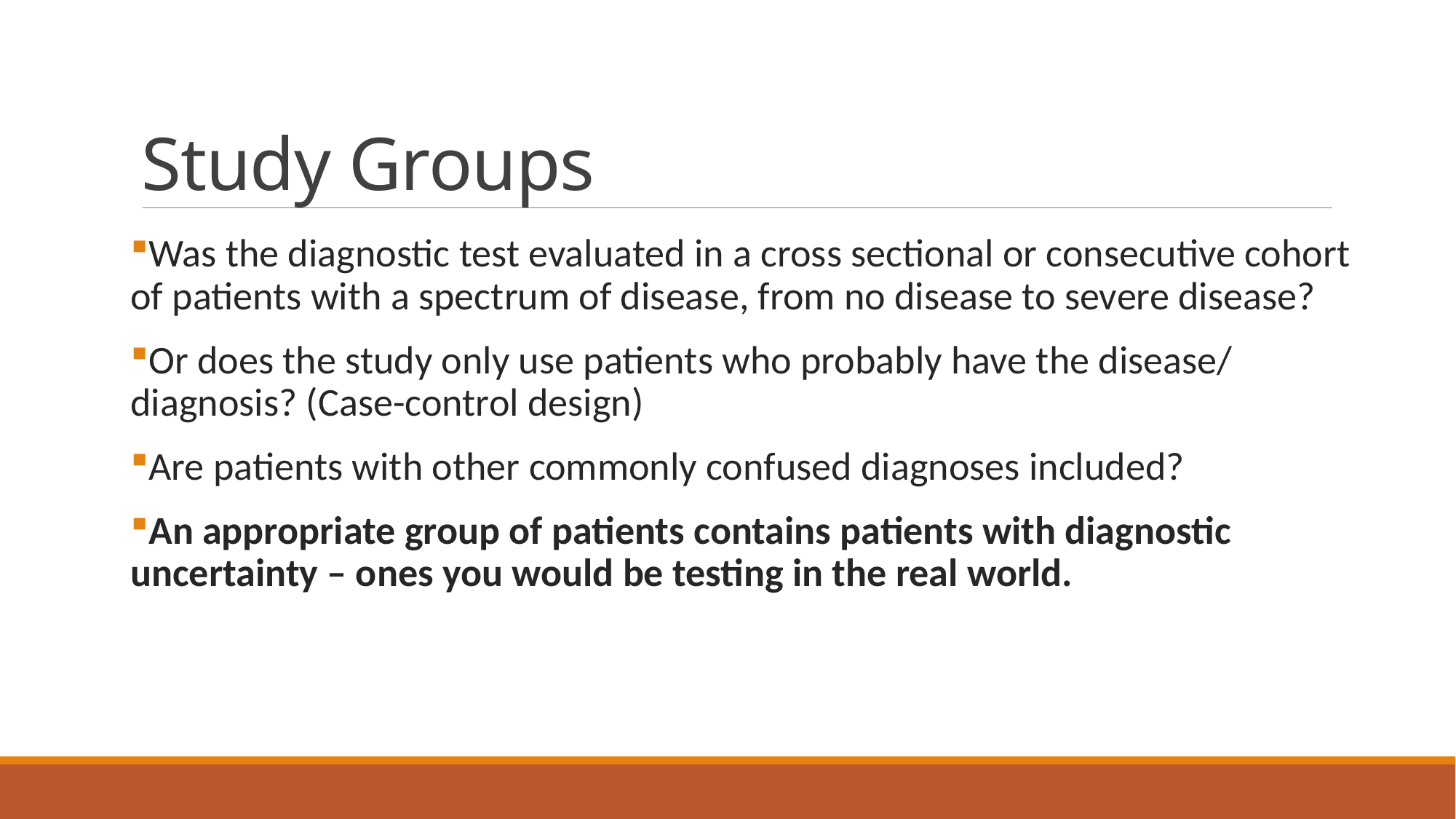

# Study Groups
Was the diagnostic test evaluated in a cross sectional or consecutive cohort of patients with a spectrum of disease, from no disease to severe disease?
Or does the study only use patients who probably have the disease/ diagnosis? (Case-control design)
Are patients with other commonly confused diagnoses included?
An appropriate group of patients contains patients with diagnostic uncertainty – ones you would be testing in the real world.

## Slide 19
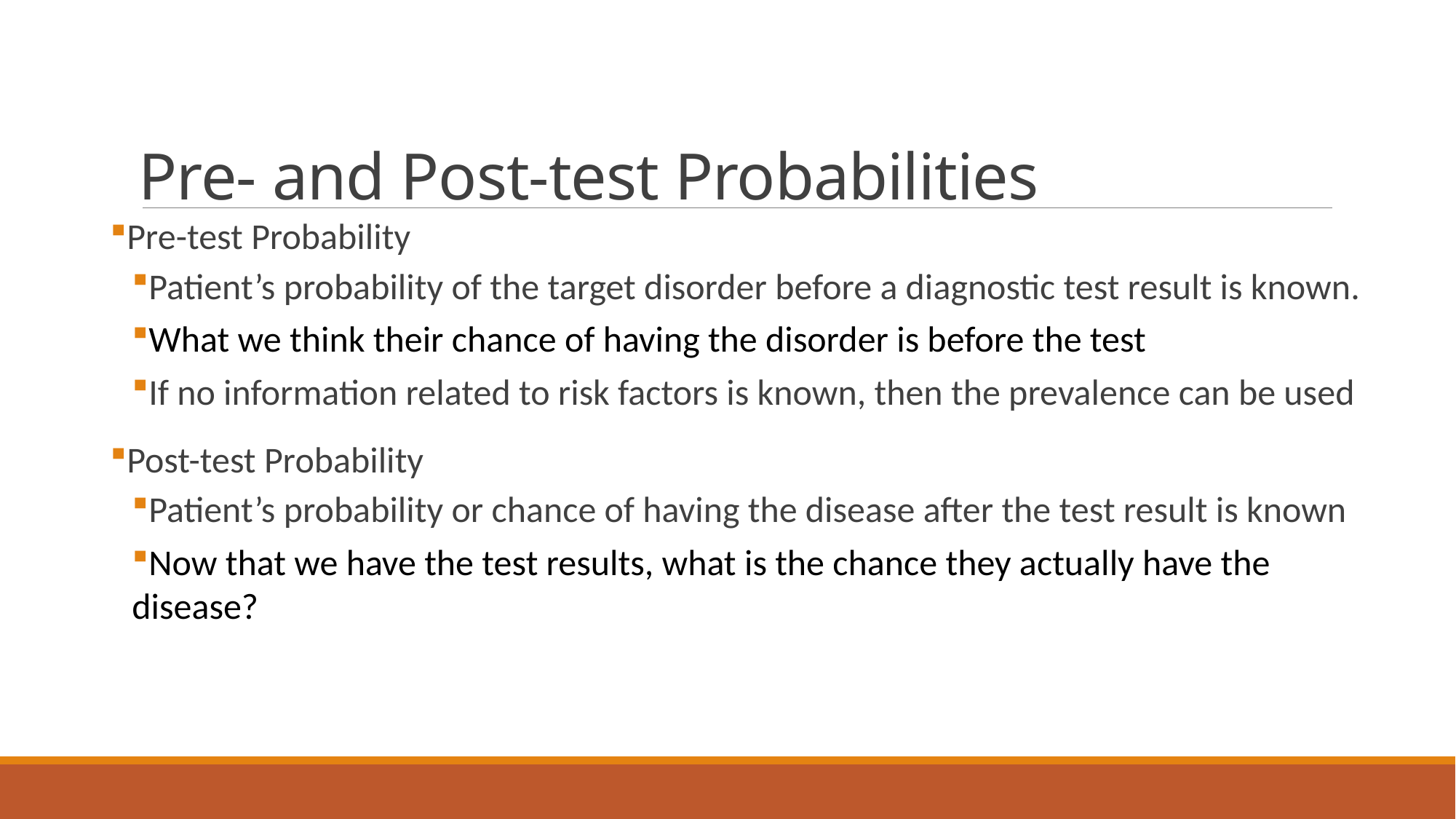

# Pre- and Post-test Probabilities
Pre-test Probability
Patient’s probability of the target disorder before a diagnostic test result is known.
What we think their chance of having the disorder is before the test
If no information related to risk factors is known, then the prevalence can be used
Post-test Probability
Patient’s probability or chance of having the disease after the test result is known
Now that we have the test results, what is the chance they actually have the disease?

## Slide 20
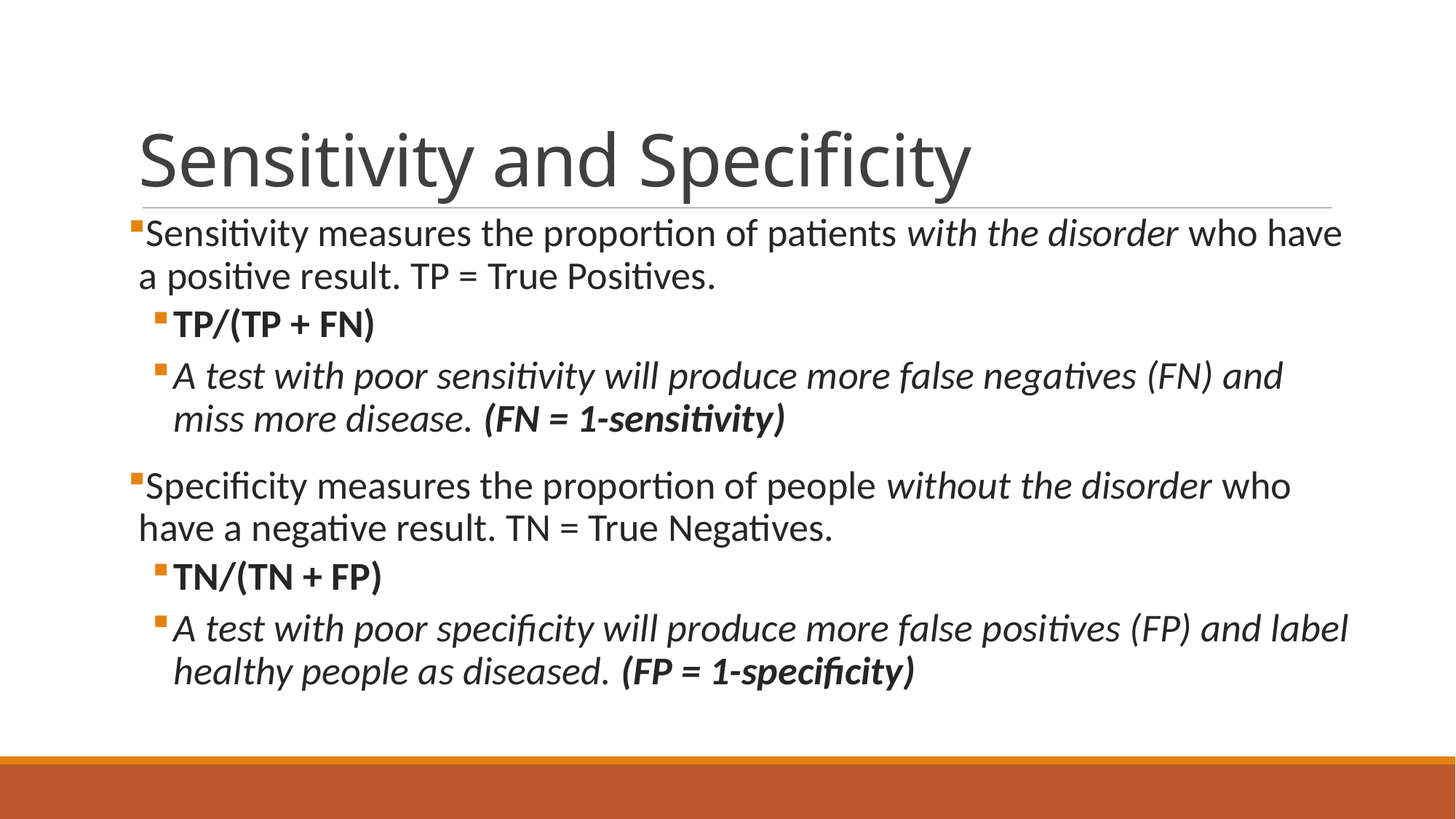

# Sensitivity and Specificity
Sensitivity measures the proportion of patients with the disorder who have a positive result. TP = True Positives.
TP/(TP + FN)
A test with poor sensitivity will produce more false negatives (FN) and miss more disease. (FN = 1-sensitivity)
Specificity measures the proportion of people without the disorder who have a negative result. TN = True Negatives.
TN/(TN + FP)
A test with poor specificity will produce more false positives (FP) and label healthy people as diseased. (FP = 1-specificity)

## Slide 21
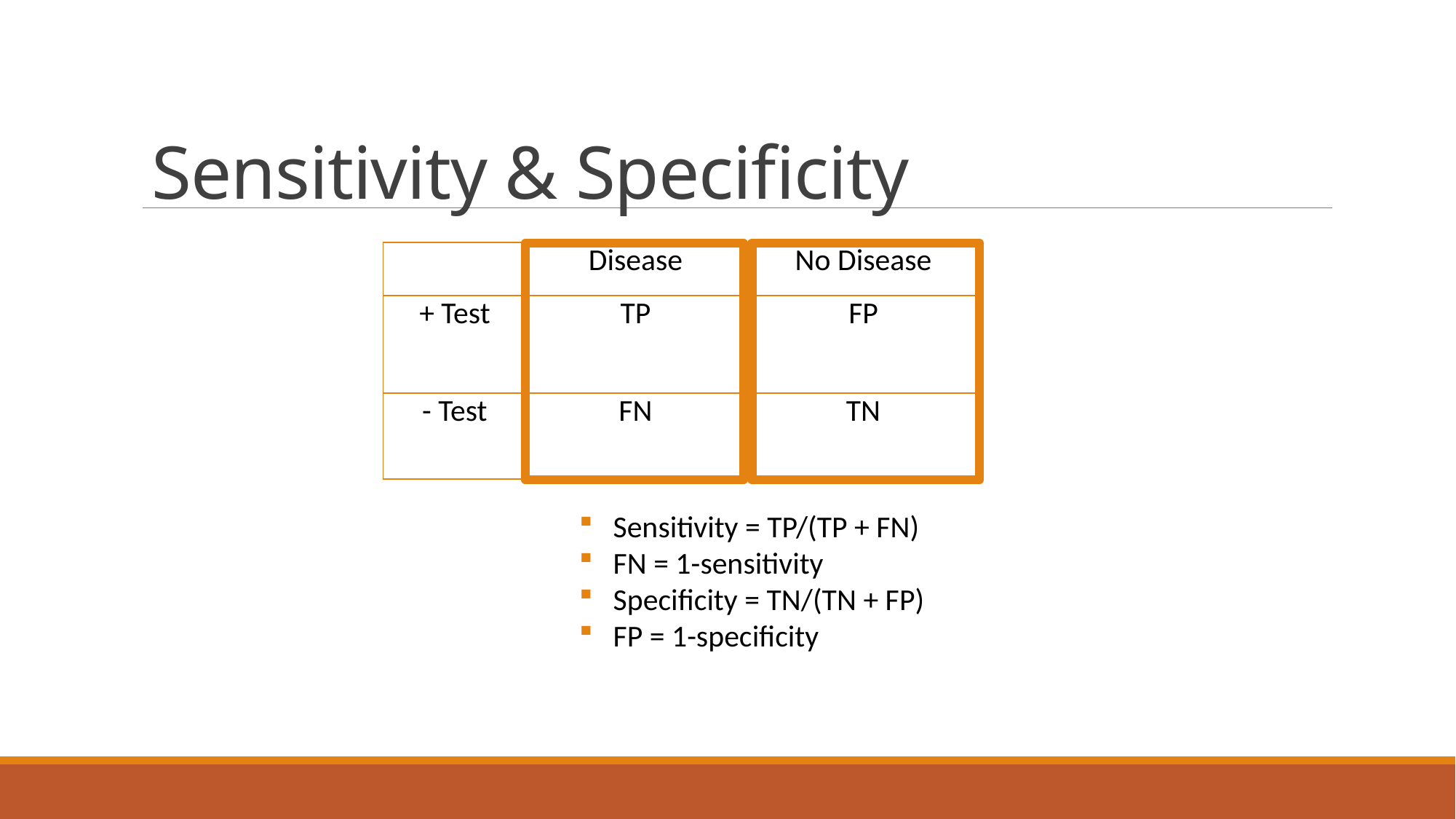

# Sensitivity & Specificity
| | Disease | No Disease |
| --- | --- | --- |
| + Test | TP | FP |
| - Test | FN | TN |
Sensitivity = TP/(TP + FN)
FN = 1-sensitivity
Specificity = TN/(TN + FP)
FP = 1-specificity

## Slide 22
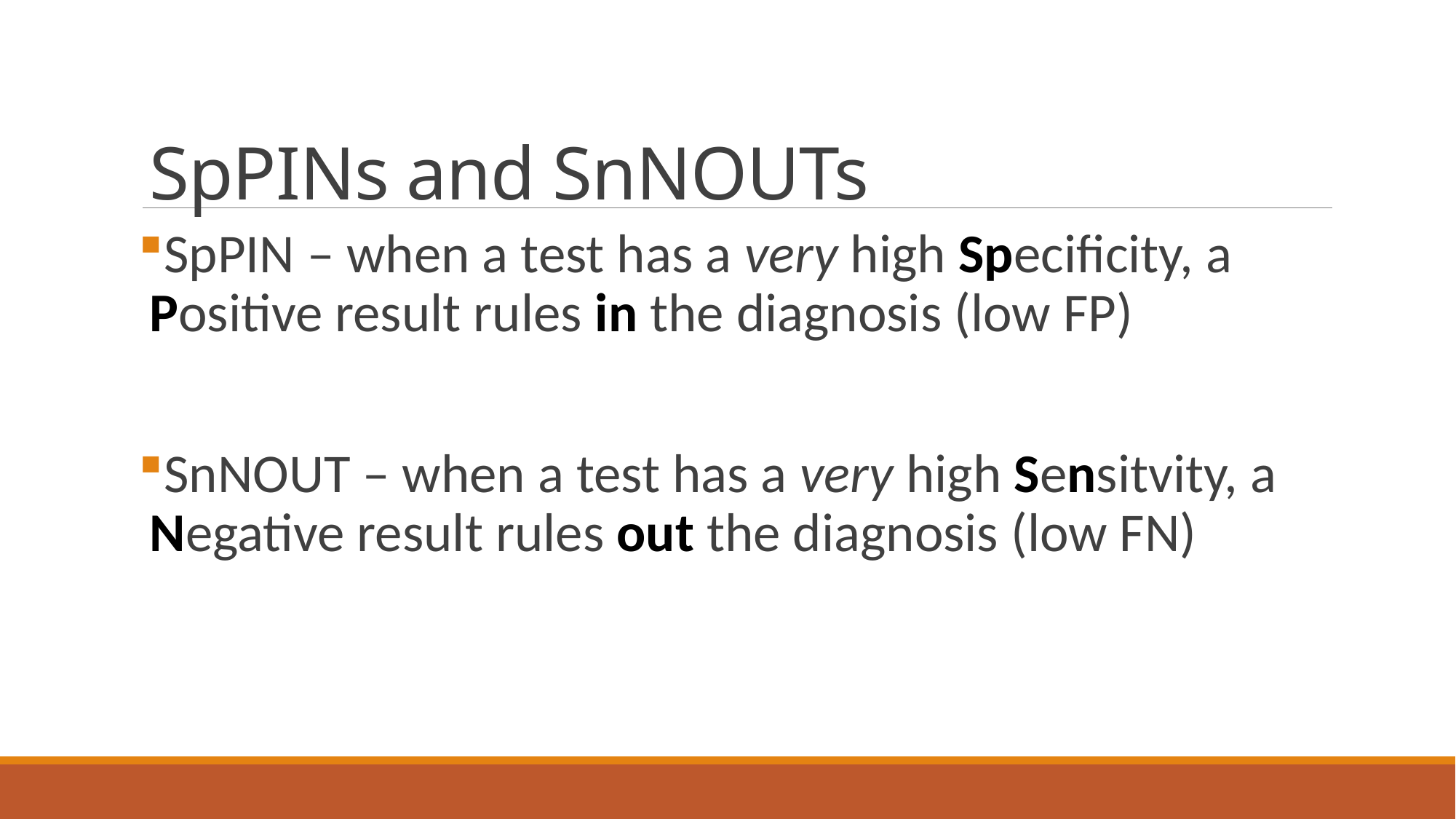

# SpPINs and SnNOUTs
SpPIN – when a test has a very high Specificity, a Positive result rules in the diagnosis (low FP)
SnNOUT – when a test has a very high Sensitvity, a Negative result rules out the diagnosis (low FN)

## Slide 23
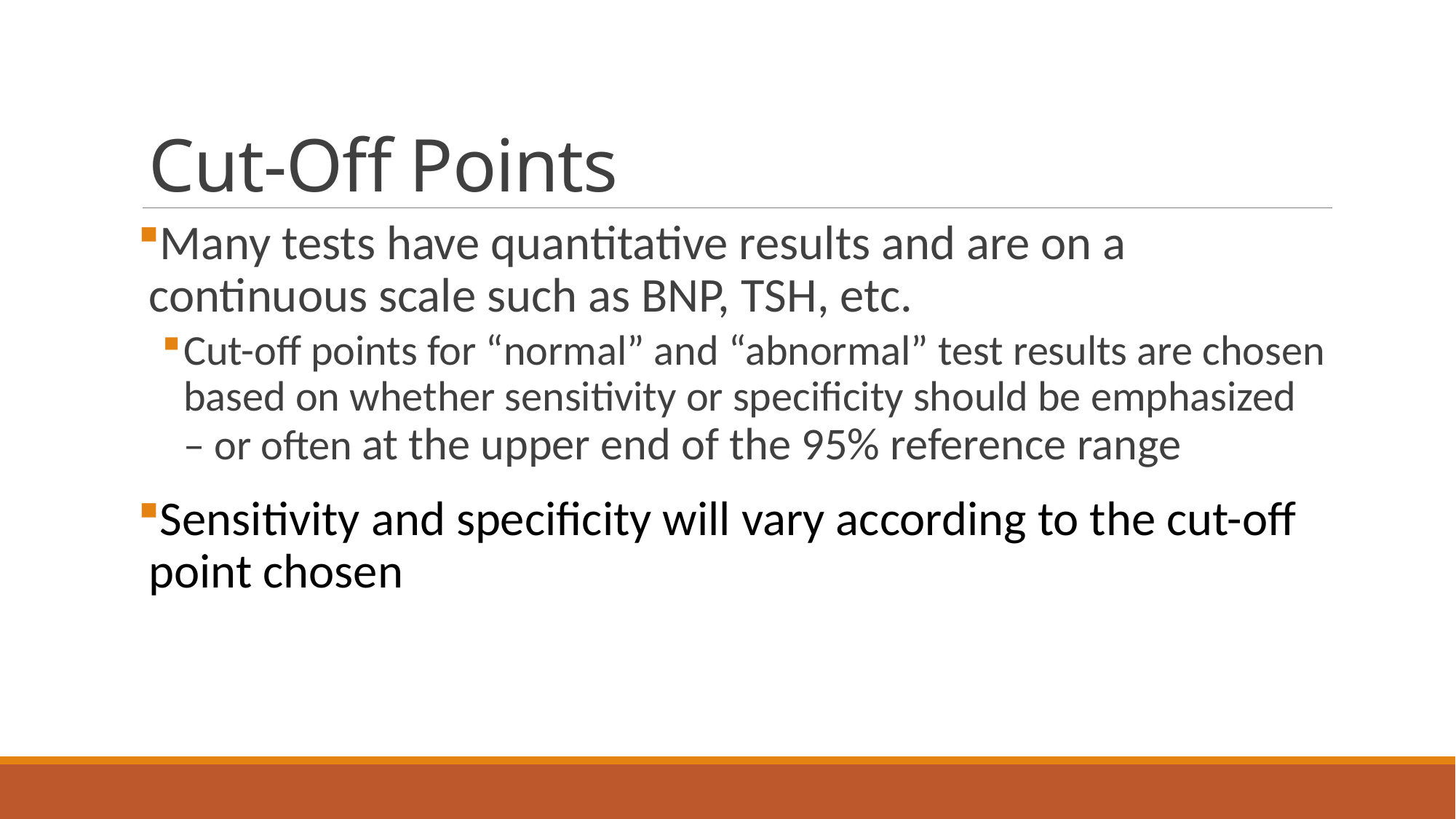

# Cut-Off Points
Many tests have quantitative results and are on a continuous scale such as BNP, TSH, etc.
Cut-off points for “normal” and “abnormal” test results are chosen based on whether sensitivity or specificity should be emphasized – or often at the upper end of the 95% reference range
Sensitivity and specificity will vary according to the cut-off point chosen

## Slide 24
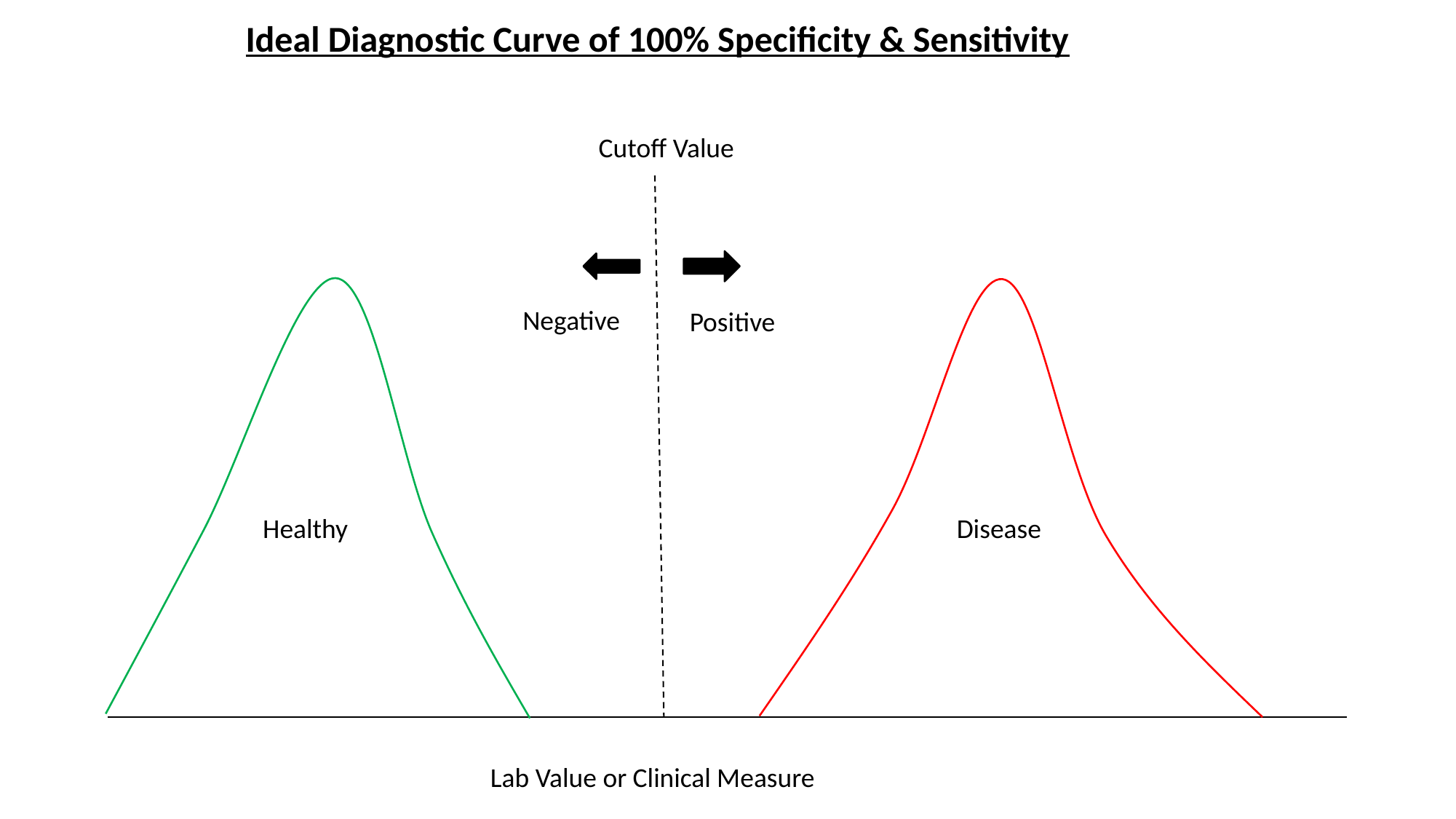

Ideal Diagnostic Curve of 100% Specificity & Sensitivity
Cutoff Value
Negative
Positive
Healthy
Disease
Lab Value or Clinical Measure

## Slide 25
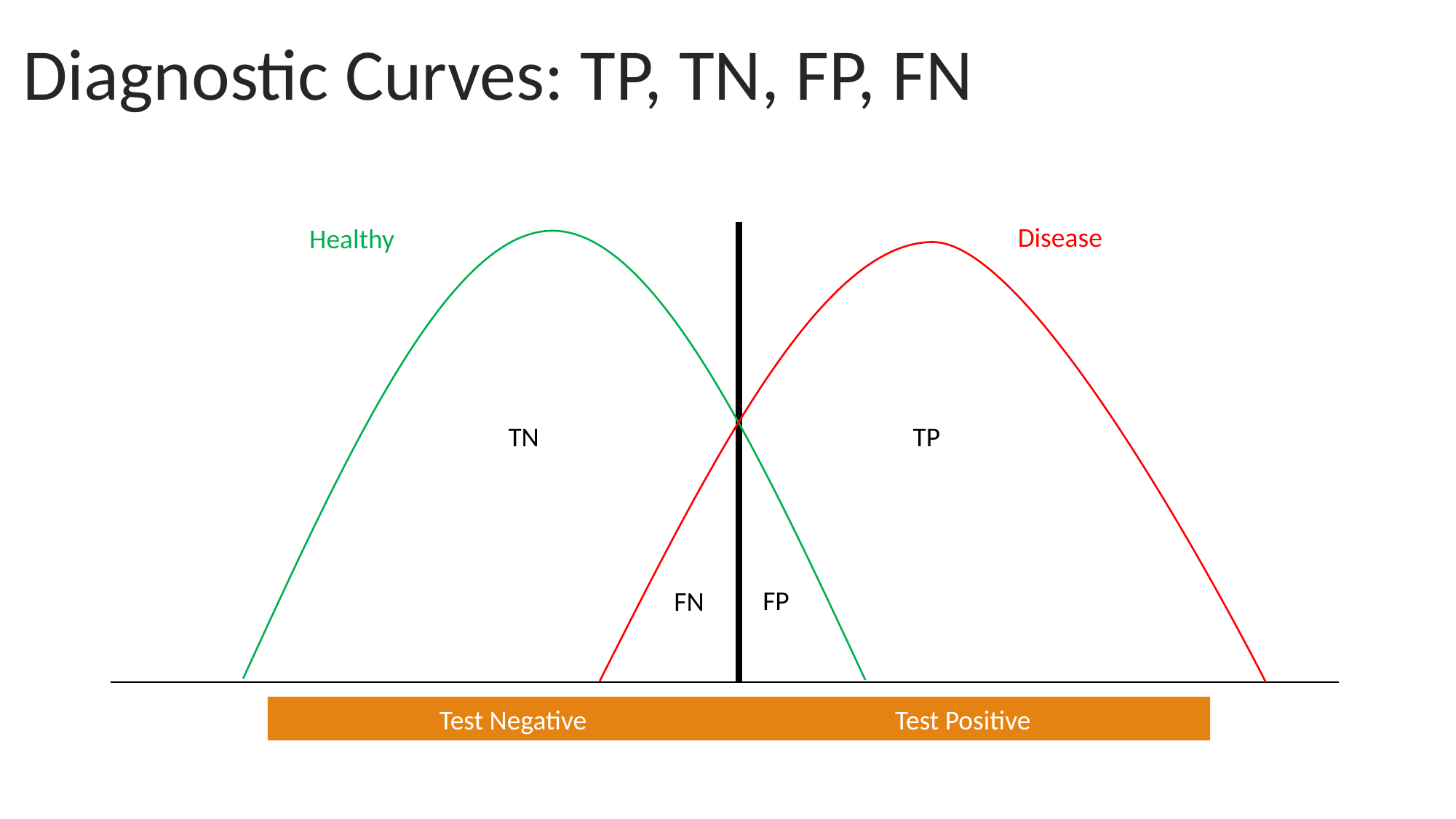

Diagnostic Curves: TP, TN, FP, FN
Disease
Healthy
 Test Negative Test Positive
TN
TP
FP
FN

## Slide 26
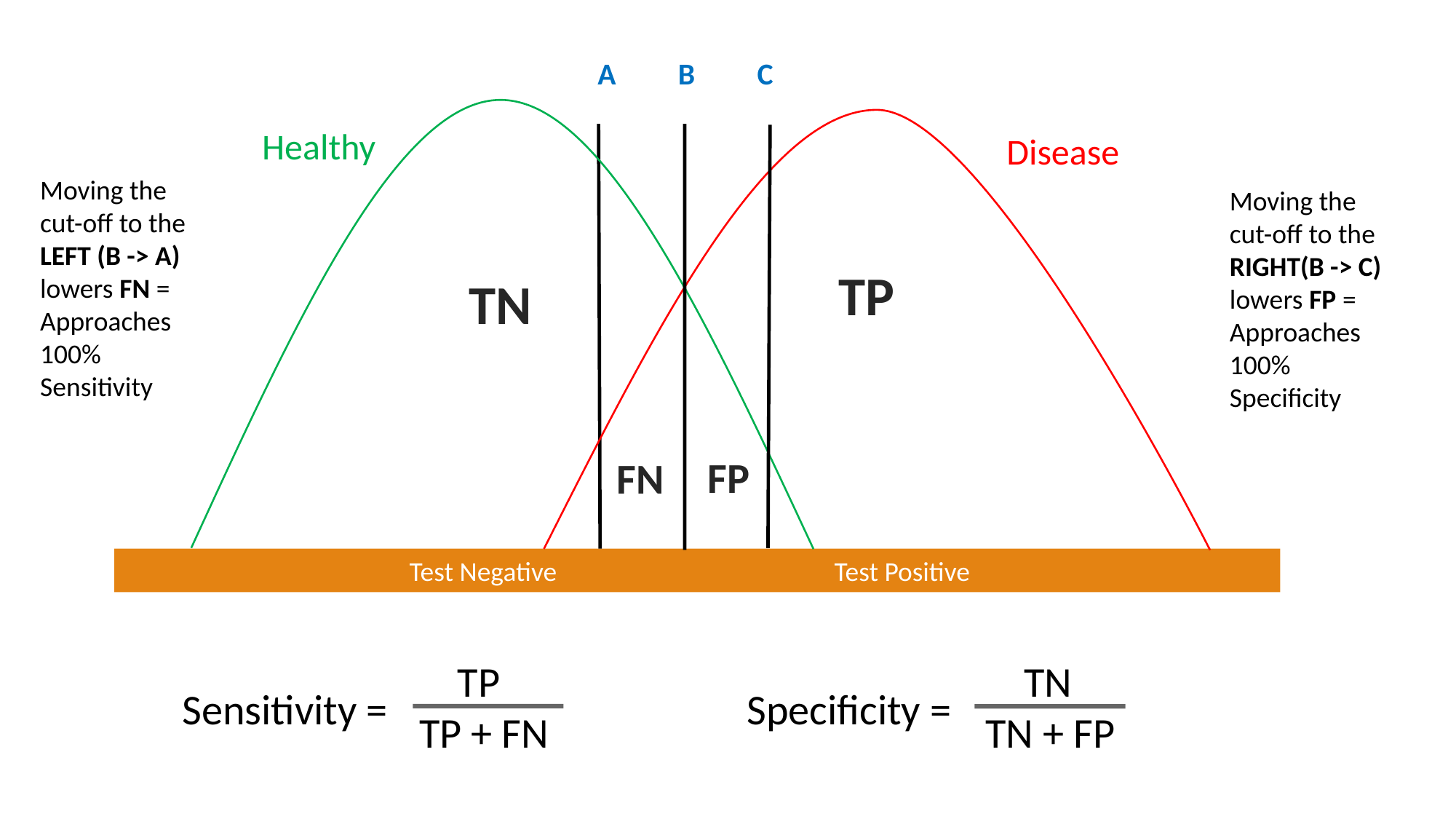

A B C
Healthy
Disease
 Test Negative Test Positive
Moving the cut-off to the LEFT (B -> A) lowers FN = Approaches 100% Sensitivity
Moving the cut-off to the RIGHT(B -> C) lowers FP = Approaches 100% Specificity
TP
TN
FP
FN
 TN
TN + FP
 TP
 TP + FN
Sensitivity =
Specificity =

## Slide 27
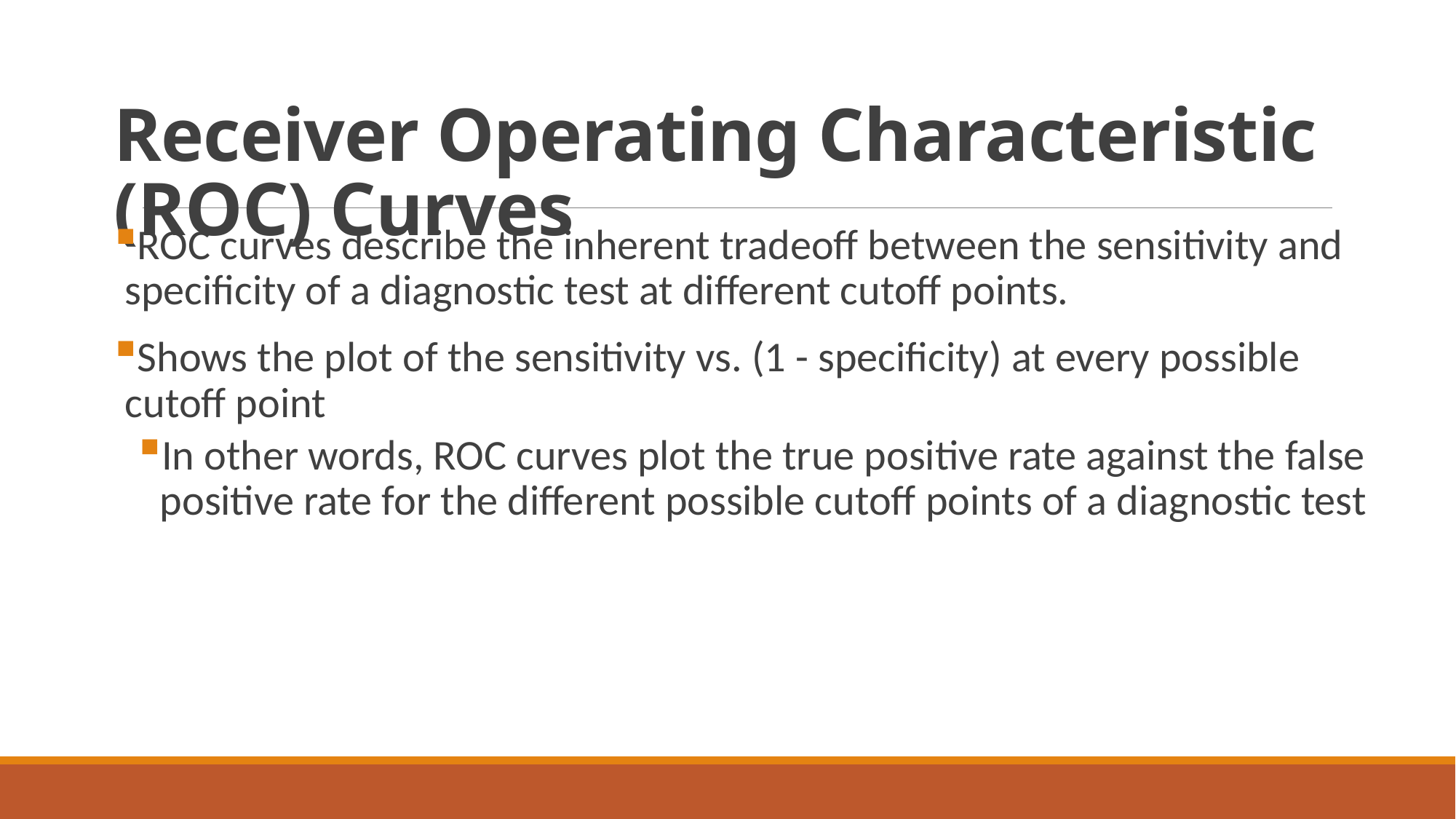

# Receiver Operating Characteristic (ROC) Curves
ROC curves describe the inherent tradeoff between the sensitivity and specificity of a diagnostic test at different cutoff points.
Shows the plot of the sensitivity vs. (1 - specificity) at every possible cutoff point
In other words, ROC curves plot the true positive rate against the false positive rate for the different possible cutoff points of a diagnostic test

## Slide 28
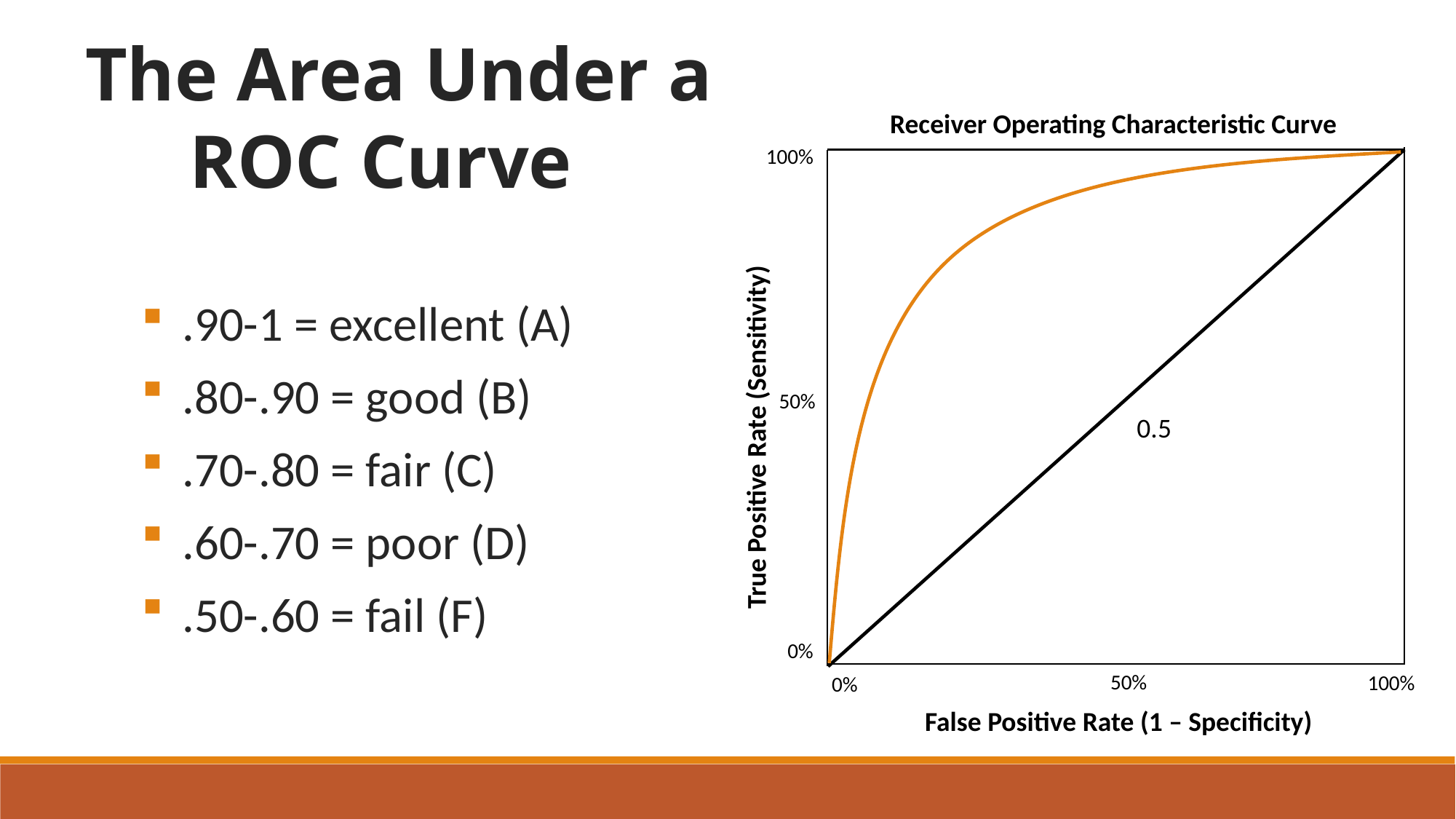

The Area Under a ROC Curve
Receiver Operating Characteristic Curve
100%
.90
.90-1 = excellent (A)
.80-.90 = good (B)
.70-.80 = fair (C)
.60-.70 = poor (D)
.50-.60 = fail (F)
50%
True Positive Rate (Sensitivity)
0.5
0%
50%
100%
0%
False Positive Rate (1 – Specificity)

## Slide 29
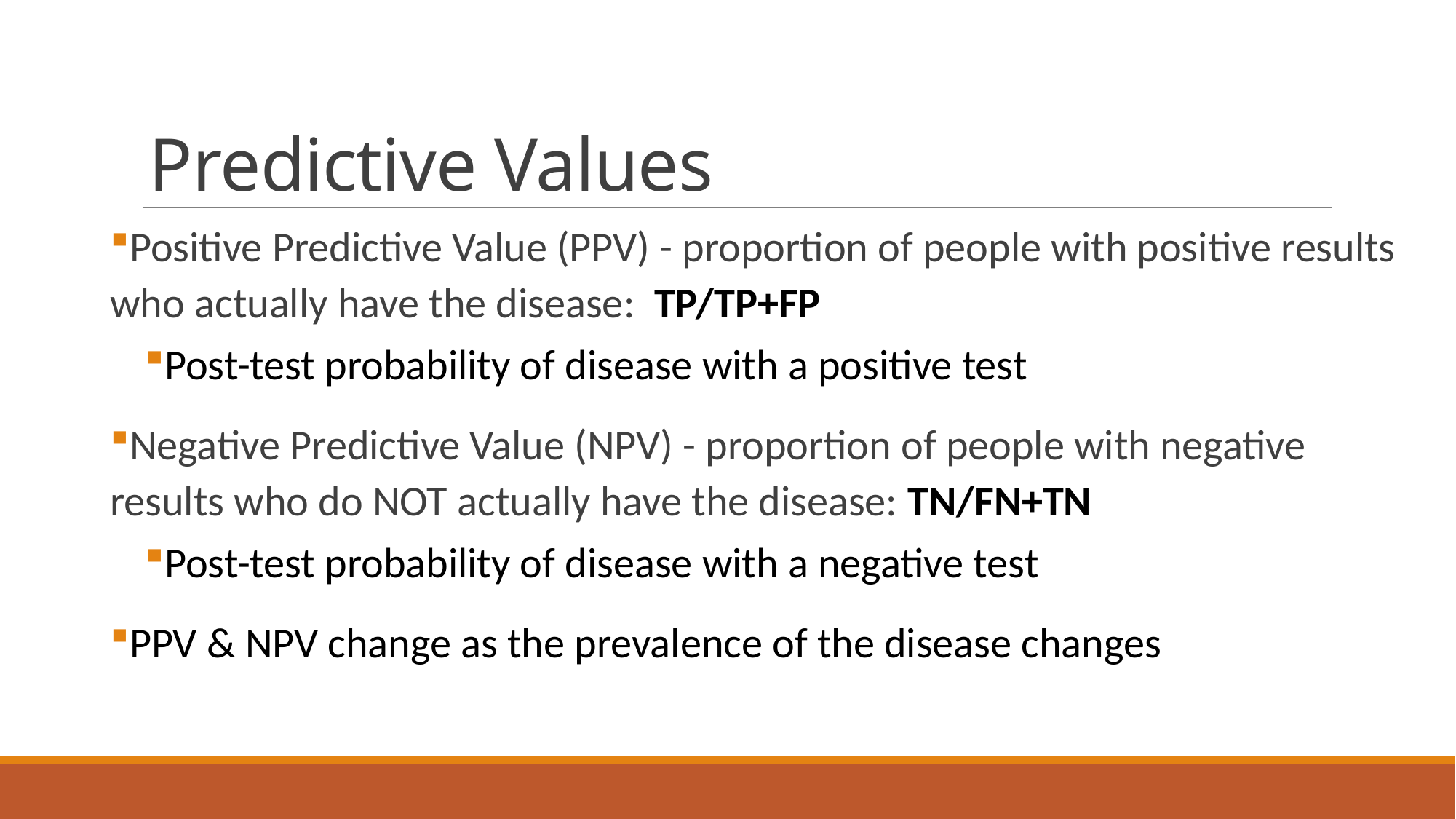

# Predictive Values
Positive Predictive Value (PPV) - proportion of people with positive results who actually have the disease: TP/TP+FP
Post-test probability of disease with a positive test
Negative Predictive Value (NPV) - proportion of people with negative results who do NOT actually have the disease: TN/FN+TN
Post-test probability of disease with a negative test
PPV & NPV change as the prevalence of the disease changes

## Slide 30
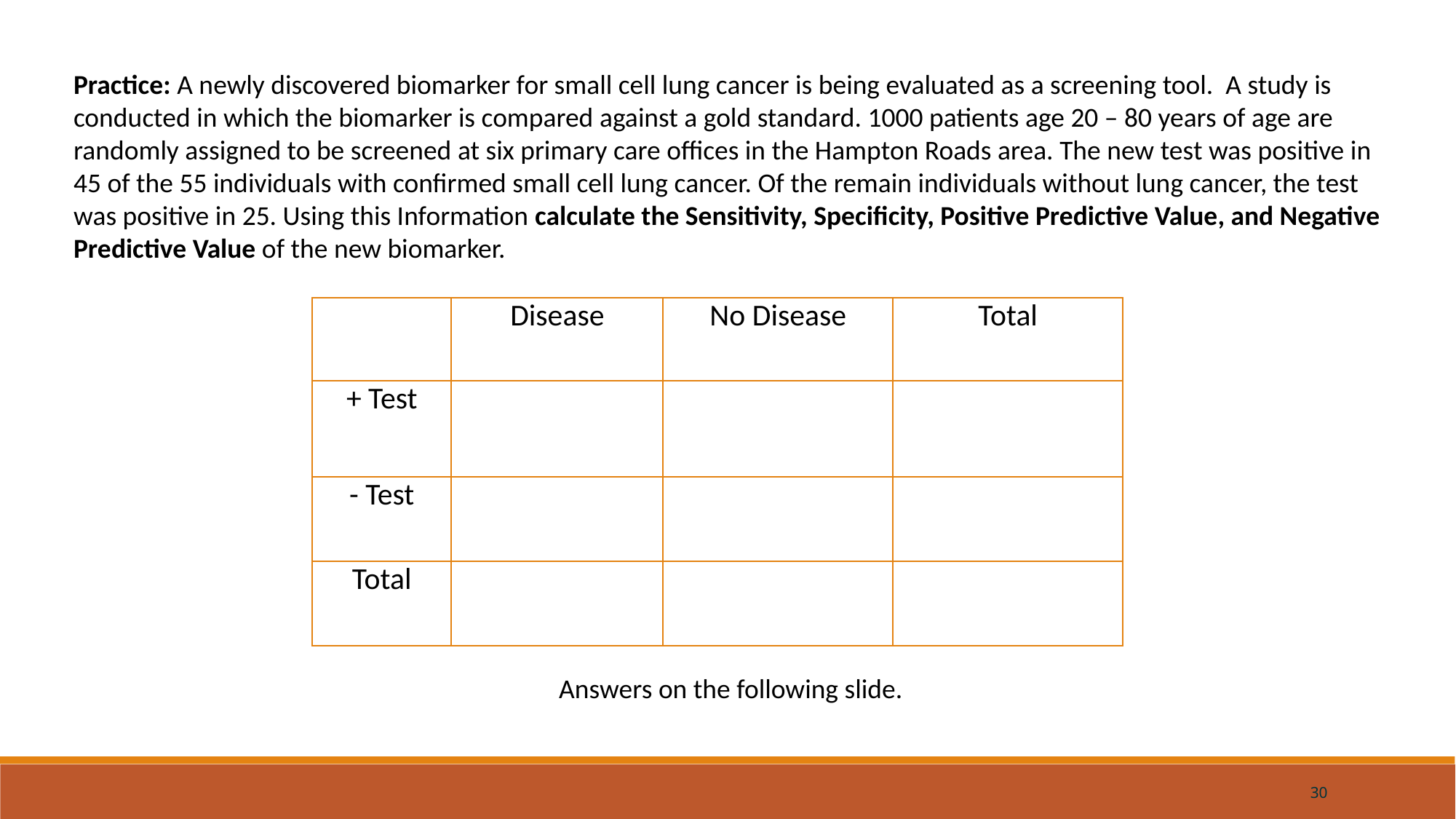

Practice: A newly discovered biomarker for small cell lung cancer is being evaluated as a screening tool. A study is conducted in which the biomarker is compared against a gold standard. 1000 patients age 20 – 80 years of age are randomly assigned to be screened at six primary care offices in the Hampton Roads area. The new test was positive in 45 of the 55 individuals with confirmed small cell lung cancer. Of the remain individuals without lung cancer, the test was positive in 25. Using this Information calculate the Sensitivity, Specificity, Positive Predictive Value, and Negative Predictive Value of the new biomarker.
| | Disease | No Disease | Total |
| --- | --- | --- | --- |
| + Test | | | |
| - Test | | | |
| Total | | | |
Answers on the following slide.
30

## Slide 31
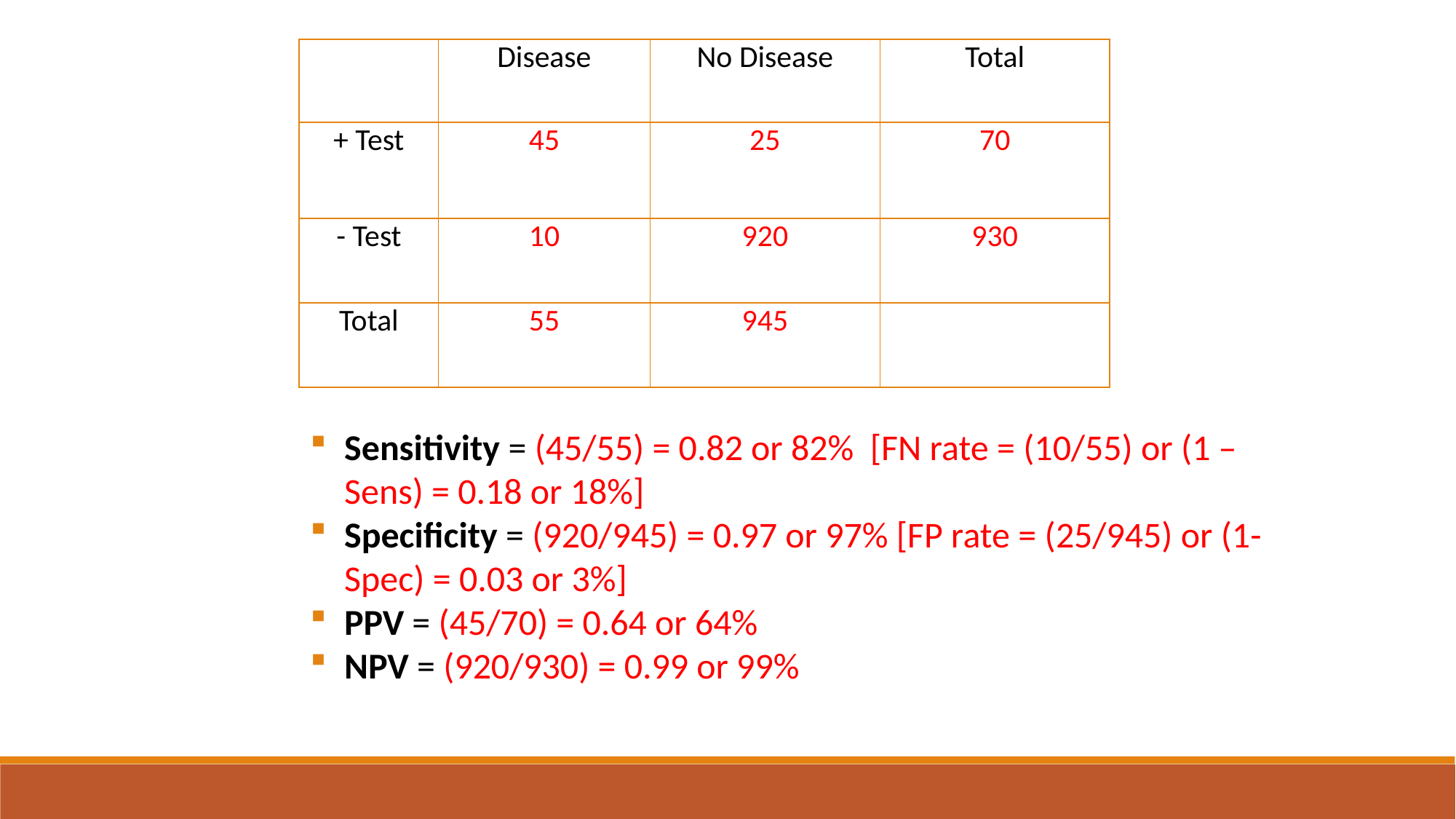

| | Disease | No Disease | Total |
| --- | --- | --- | --- |
| + Test | 45 | 25 | 70 |
| - Test | 10 | 920 | 930 |
| Total | 55 | 945 | |
Sensitivity = (45/55) = 0.82 or 82% [FN rate = (10/55) or (1 – Sens) = 0.18 or 18%]
Specificity = (920/945) = 0.97 or 97% [FP rate = (25/945) or (1-Spec) = 0.03 or 3%]
PPV = (45/70) = 0.64 or 64%
NPV = (920/930) = 0.99 or 99%

## Slide 32
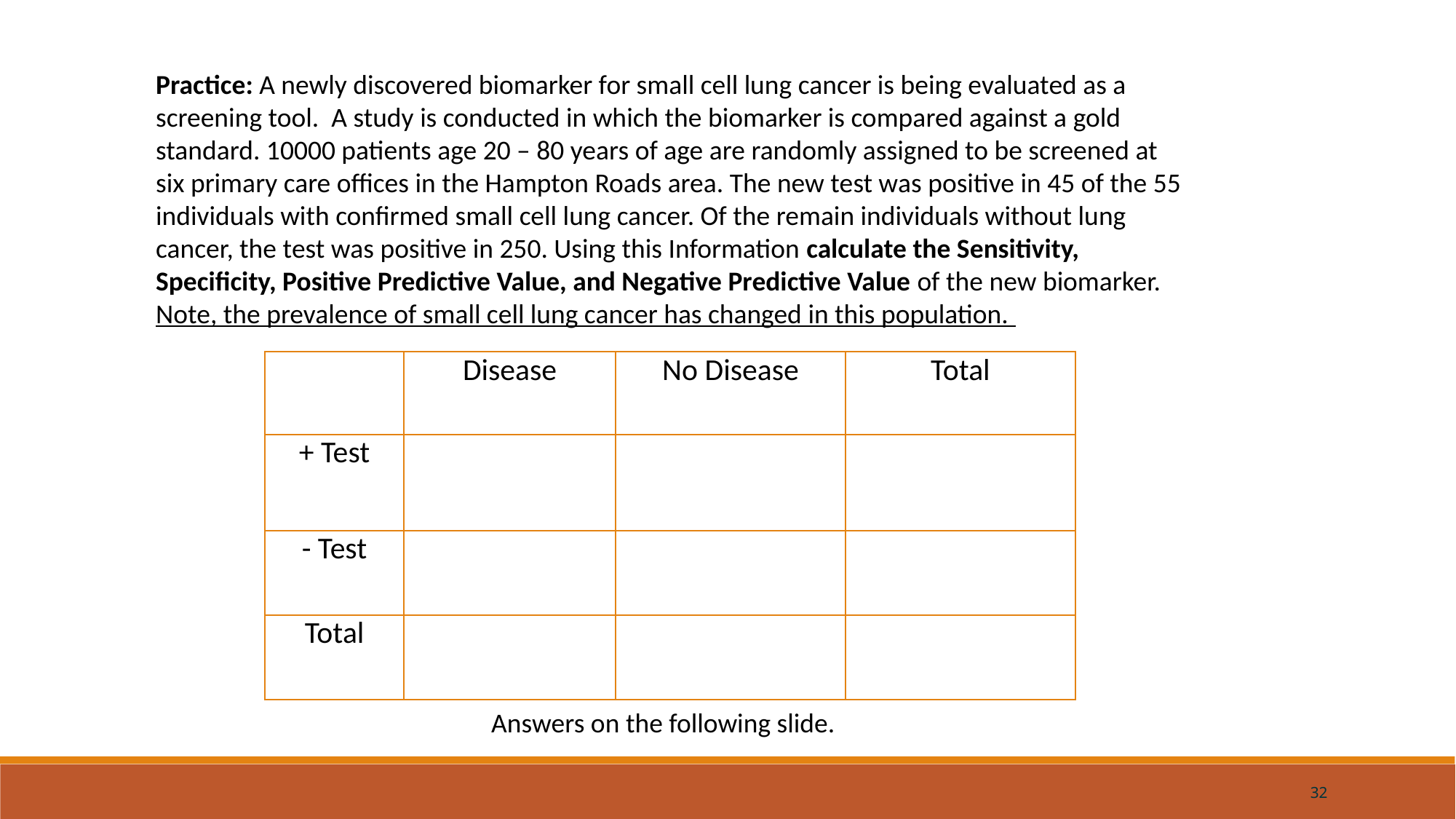

Practice: A newly discovered biomarker for small cell lung cancer is being evaluated as a screening tool. A study is conducted in which the biomarker is compared against a gold standard. 10000 patients age 20 – 80 years of age are randomly assigned to be screened at six primary care offices in the Hampton Roads area. The new test was positive in 45 of the 55 individuals with confirmed small cell lung cancer. Of the remain individuals without lung cancer, the test was positive in 250. Using this Information calculate the Sensitivity, Specificity, Positive Predictive Value, and Negative Predictive Value of the new biomarker. Note, the prevalence of small cell lung cancer has changed in this population.
| | Disease | No Disease | Total |
| --- | --- | --- | --- |
| + Test | | | |
| - Test | | | |
| Total | | | |
Answers on the following slide.
32

## Slide 33
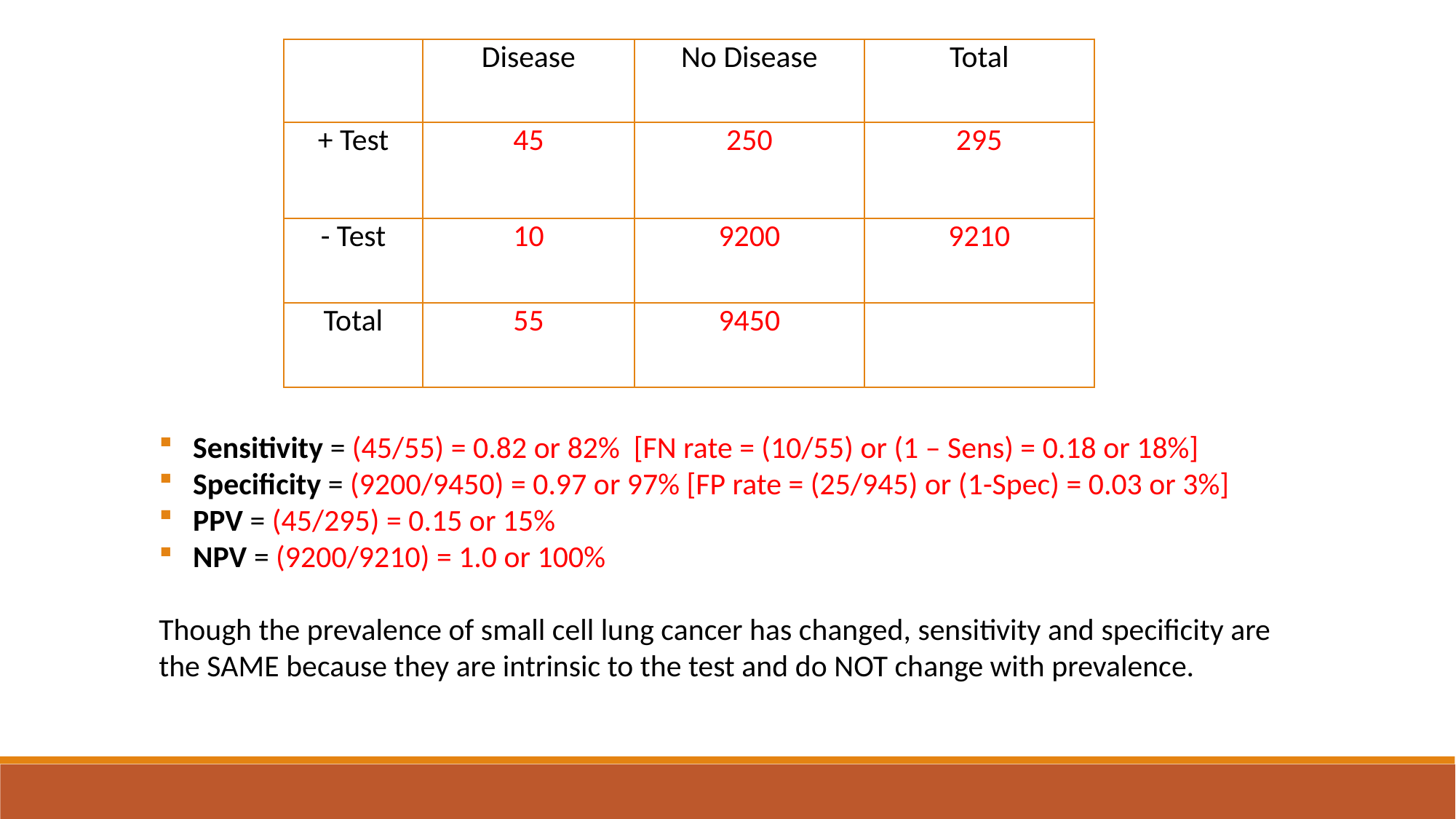

| | Disease | No Disease | Total |
| --- | --- | --- | --- |
| + Test | 45 | 250 | 295 |
| - Test | 10 | 9200 | 9210 |
| Total | 55 | 9450 | |
Sensitivity = (45/55) = 0.82 or 82% [FN rate = (10/55) or (1 – Sens) = 0.18 or 18%]
Specificity = (9200/9450) = 0.97 or 97% [FP rate = (25/945) or (1-Spec) = 0.03 or 3%]
PPV = (45/295) = 0.15 or 15%
NPV = (9200/9210) = 1.0 or 100%
Though the prevalence of small cell lung cancer has changed, sensitivity and specificity are the SAME because they are intrinsic to the test and do NOT change with prevalence.

## Slide 34
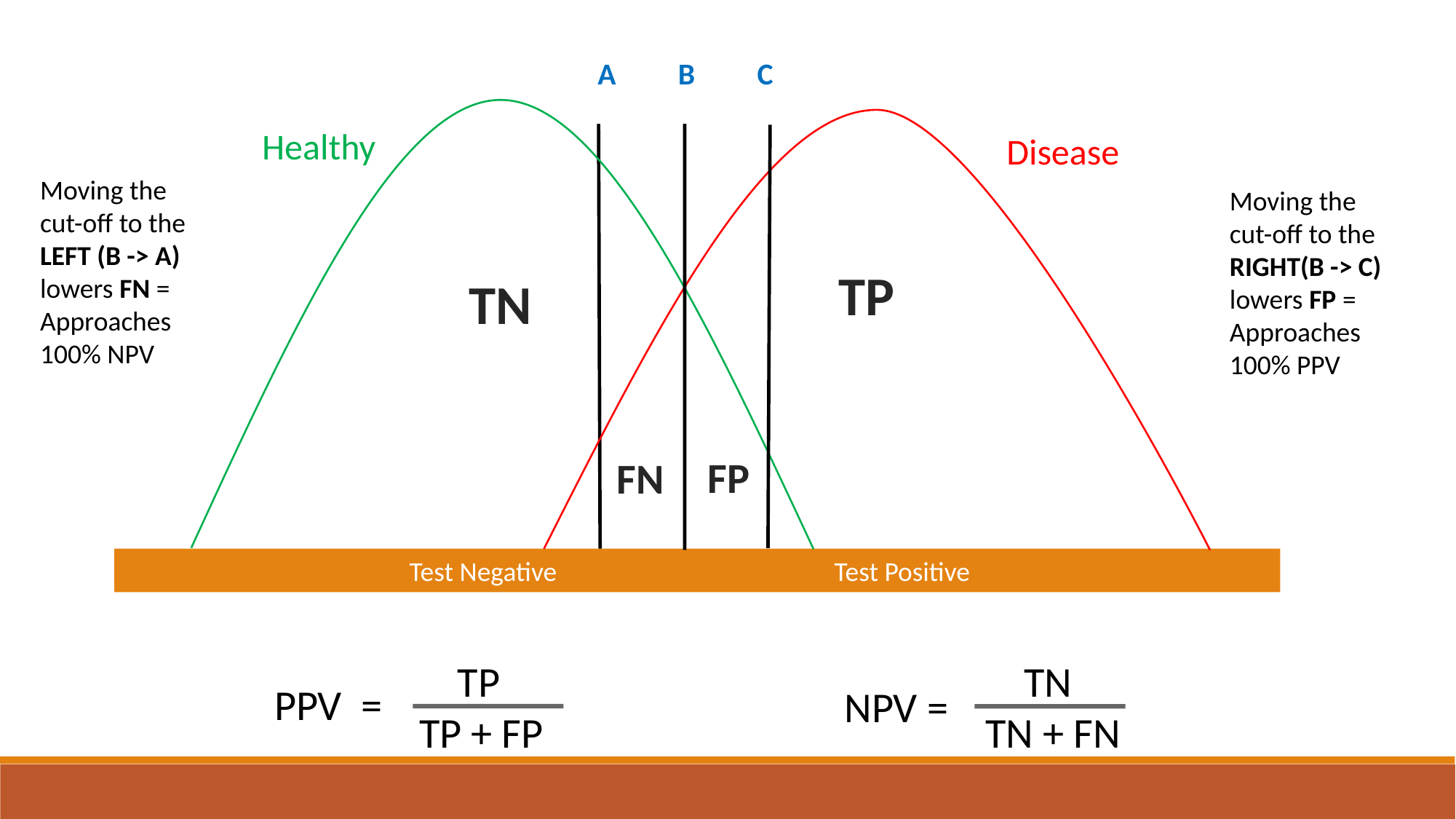

A B C
Healthy
Disease
 Test Negative Test Positive
Moving the cut-off to the LEFT (B -> A) lowers FN = Approaches 100% NPV
Moving the cut-off to the RIGHT(B -> C) lowers FP = Approaches 100% PPV
TP
TN
FP
FN
 TN
TN + FN
 TP
 TP + FP
PPV =
NPV =

## Slide 35
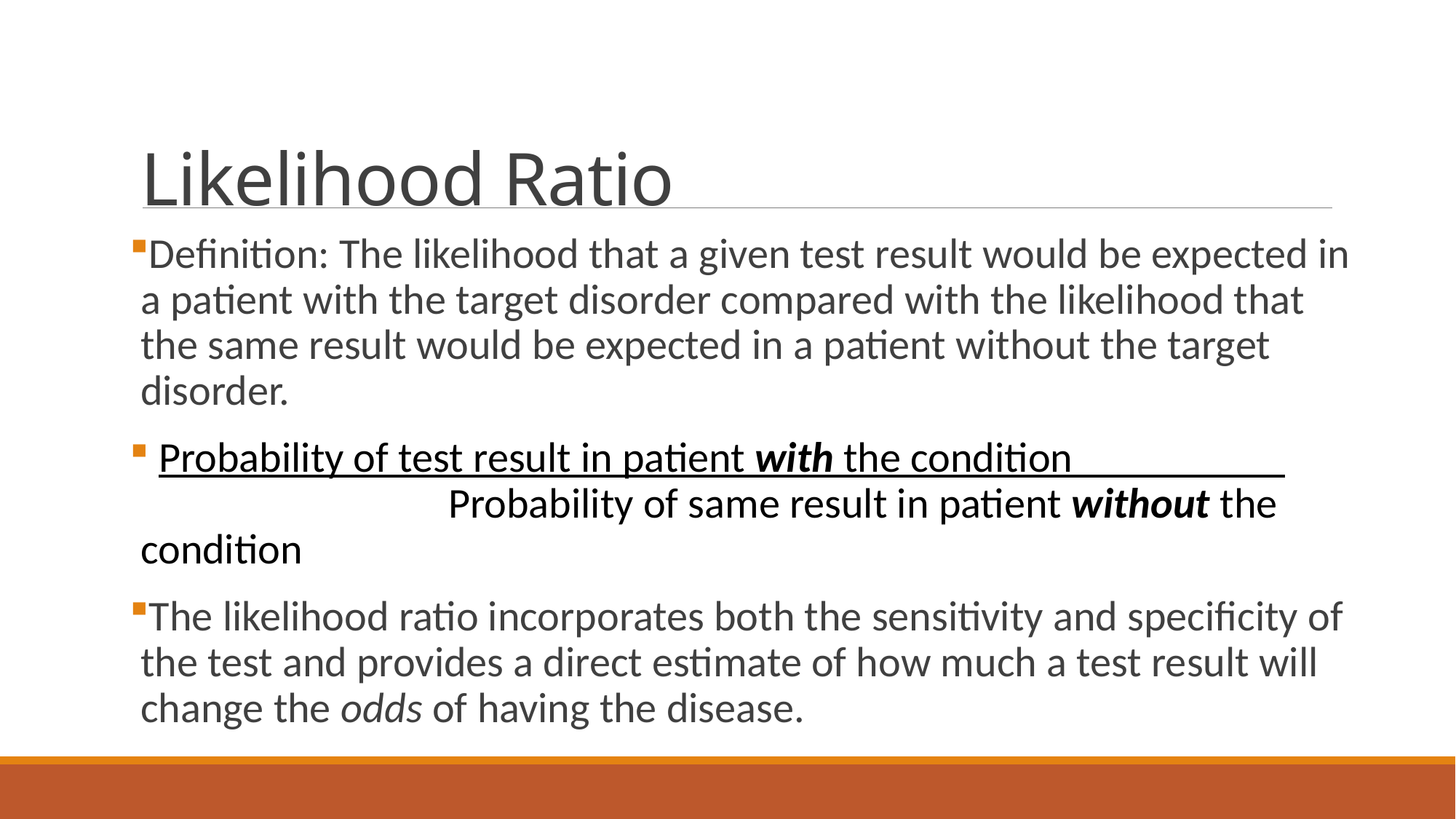

# Likelihood Ratio
Definition: The likelihood that a given test result would be expected in a patient with the target disorder compared with the likelihood that the same result would be expected in a patient without the target disorder.
 Probability of test result in patient with the condition Probability of same result in patient without the condition
The likelihood ratio incorporates both the sensitivity and specificity of the test and provides a direct estimate of how much a test result will change the odds of having the disease.
LR=

## Slide 36
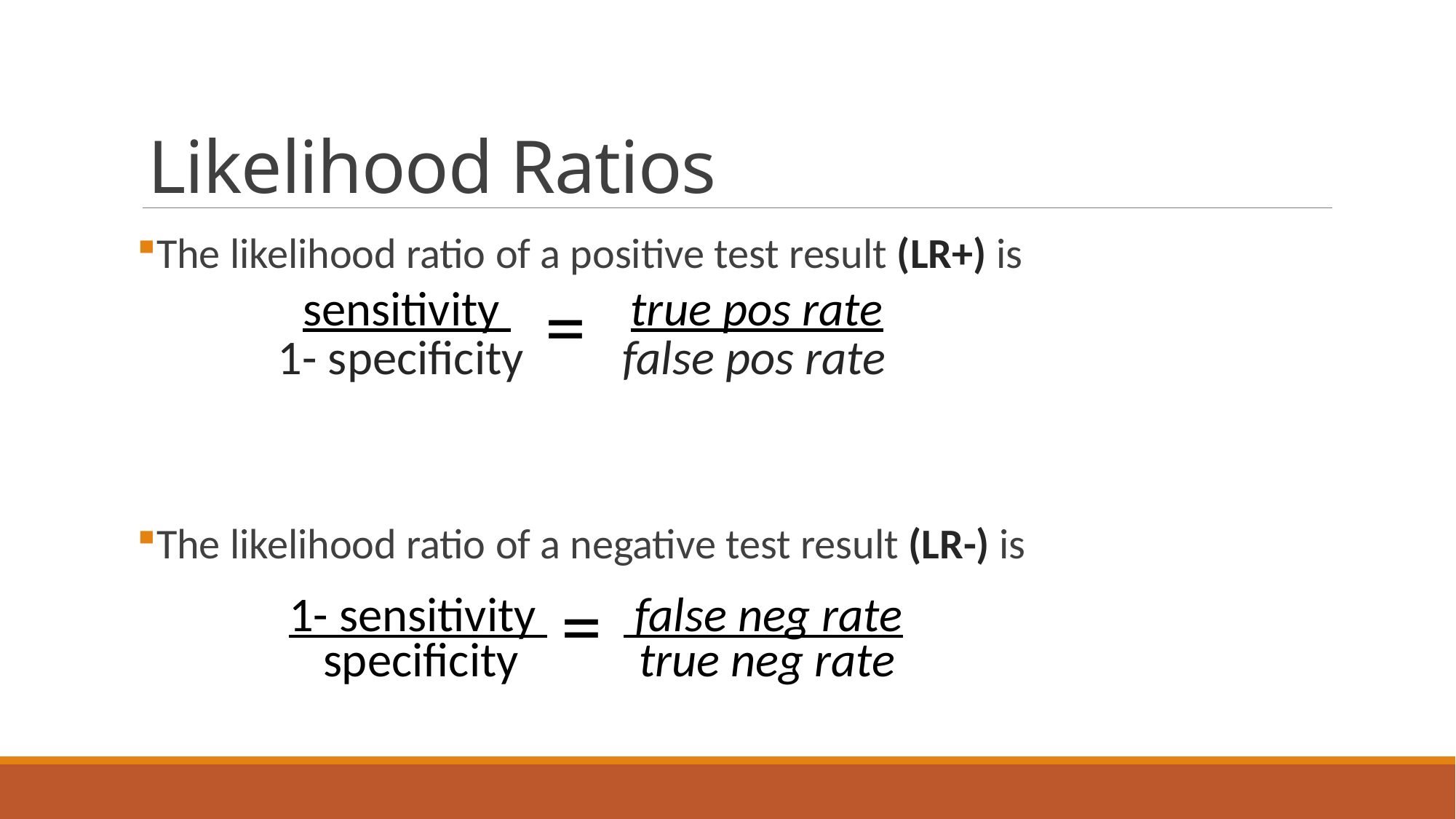

# Likelihood Ratios
The likelihood ratio of a positive test result (LR+) is
 sensitivity true pos rate
The likelihood ratio of a negative test result (LR-) is
 1- sensitivity false neg rate
=
1- specificity false pos rate
=
specificity true neg rate

## Slide 37
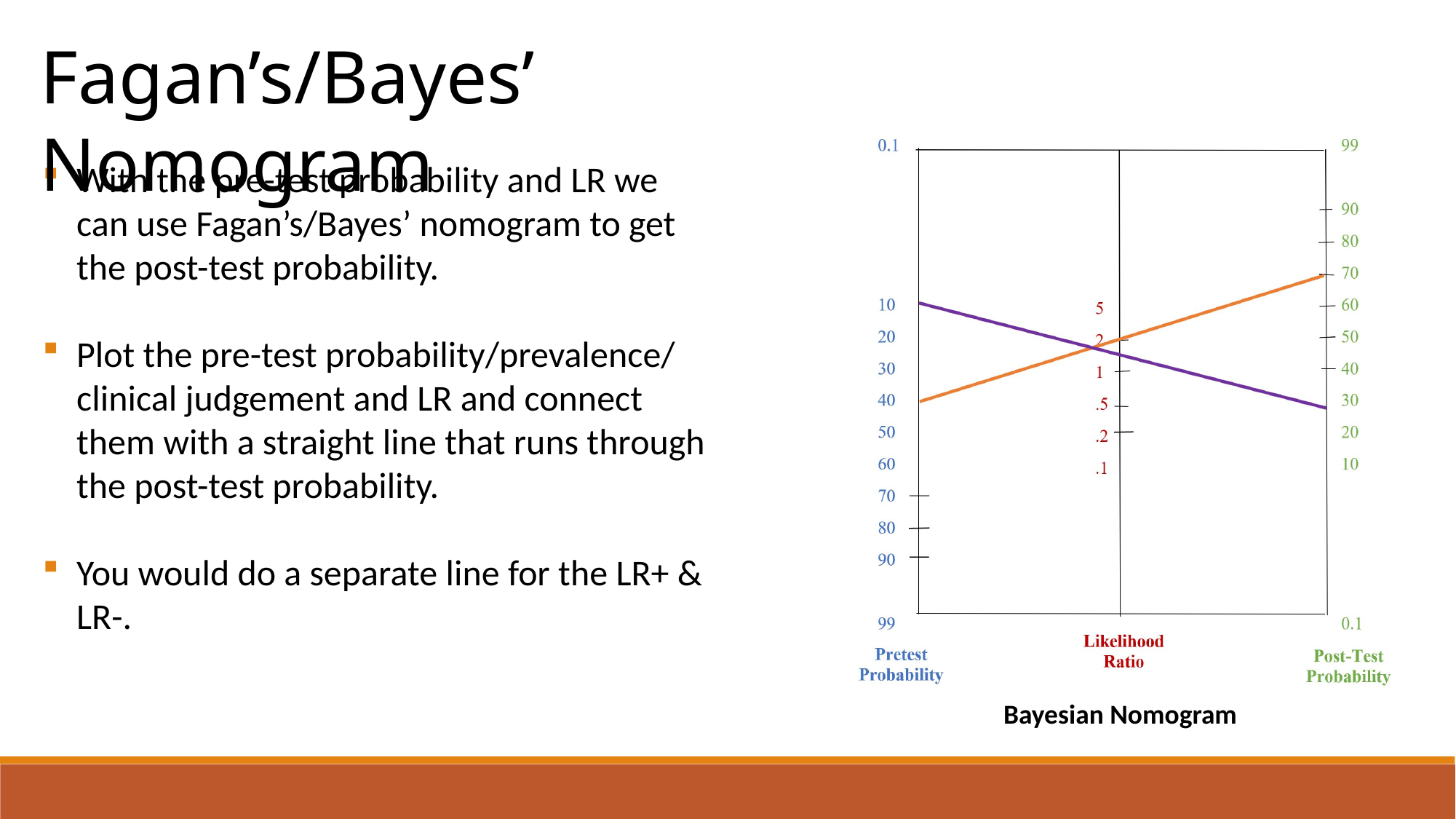

Fagan’s/Bayes’ Nomogram
With the pre-test probability and LR we can use Fagan’s/Bayes’ nomogram to get the post-test probability.
Plot the pre-test probability/prevalence/ clinical judgement and LR and connect them with a straight line that runs through the post-test probability.
You would do a separate line for the LR+ & LR-.
Bayesian Nomogram
