# Supplementary material for: Peer-Developed Modules on Basic Biostatistics and Evidence-Based Medicine Principles for Undergraduate Medical Education
Source: MedEdPORTAL. 2020 Nov 24;16:11026. doi: 10.15766/mep_2374-8265.11026 (PMC7703476; doi:10.15766/mep_2374-8265.11026)
Supplement: Supplementary file 1 — Module 1 Study Design and Bias.pptxModule 1 Problem Set.docxModule 1 Problem Set Answer Key.docxModule 1 Formative Quiz.docxModule 1 Formative Quiz Answer Key.docxModule 2 Interpreting Data from Clinical Trials.pptxModule 2 Problem Set.docxModule 2 Problem Set Answer Key.docxModule 2 Formative Quiz.docxModule 2 Formative Quiz Answer Key.docxModule 3 Diagnostic and Therapy Trial Results.pptxModule 3 Problem Set.docxModule 3 Problem Set Answer Key.docxModule 3 Formative Quiz.docxModule 3 Formative Quiz Answer Key.docxImplementation Guide.docxPostsession Evaluation Survey.docx [file mep_2374-8265.11026-s001.zip › M. Module 3 Problem Set Answer Key.docx]

**Module 3 Problem Set Answer Key**

Instructions: Please review the following answers. For incorrect answers, please refer back to module and/or discuss with peers on why each answer was correct or incorrect.

**Case 1**

The year is 2019 and you’re an M3 on your internal medicine clerkship when…*The zombie apocalypse begins!* You have been holed up in the hospital for at least a month and by now 60% of the general population has been afflicted with the zombie virus. You’ve seen 200 patients who have been bit by a zombie and of those patients, 150 individuals have turned into zombies and 50 have remained symptom free within one week of exposure. Of the 150 patients who became zombies, 120 of them had leukocytosis (WBC >11,000 per mm^3^). Of the 50 patients who did not turn into zombies, 25 of them had leukocytosis.

1. One of your friends was out collecting food when he got surprised by a zombie several hours ago. Physical exam findings reveal a suspicious bite mark on his arm. While waiting for his lab results your attending turns to you and asks, *“what is the pre-test probability of your friend becoming a zombie?”*
   1. 0%
   2. 25%
   3. 45%
   4. 60%
   5. **75%**
   6. 100%

The pre-test probability is an estimate of a positive disease/condition taking into account all known risk factors. In this case, you are given an observed estimate of the number of people who would become zombies following a bite: 150/200 = .75

Notably, we were given prevalence of the disease as a whole (60%) but that is not the best pretest probability because it does not take into account known risk factors.

Your friend’s WBC count comes back at 10,000 per mm^3^, a negative result. He insists he feels fine, but you’ve seen enough zombie movies to know not to go to sleep when someone might turn over night… So you open your textbook to make some calculations…

1. Calculate the sensitivity and specificity of leukocytosis as a predictor for the zombie virus.

*Sensitivity: 120/150 = .8*

*Specificity: 25/50 = .5*

1. Then, assuming your friend’s pre-test probability is equal to the prevalence in your in-hospital observations, what post-test probability do you report to your attending?

*Approximately 55%.* *When your patient matches the test population, you may use the negative predictive value or positive predictive value for the post-test probability. If they do not, the math becomes more complex and it’s easier to use the nomogram as shown below. However, questions will typically write something along the lines of, “the patient matches the sample population” to indicate that NPV and PPV accurately represent the post-test probability. Because our patient had a negative test, we use NPV = TN/FN+TN.*

1. Your attending argues that due to other risk factors, your friend’s pre-test probability should realistically be closer to 90%. You sigh and refer to the Bayesian Nomogram provided below. Calculate a negative likelihood ratio and decide his new post-test probability that is best represented by which of the following lines?


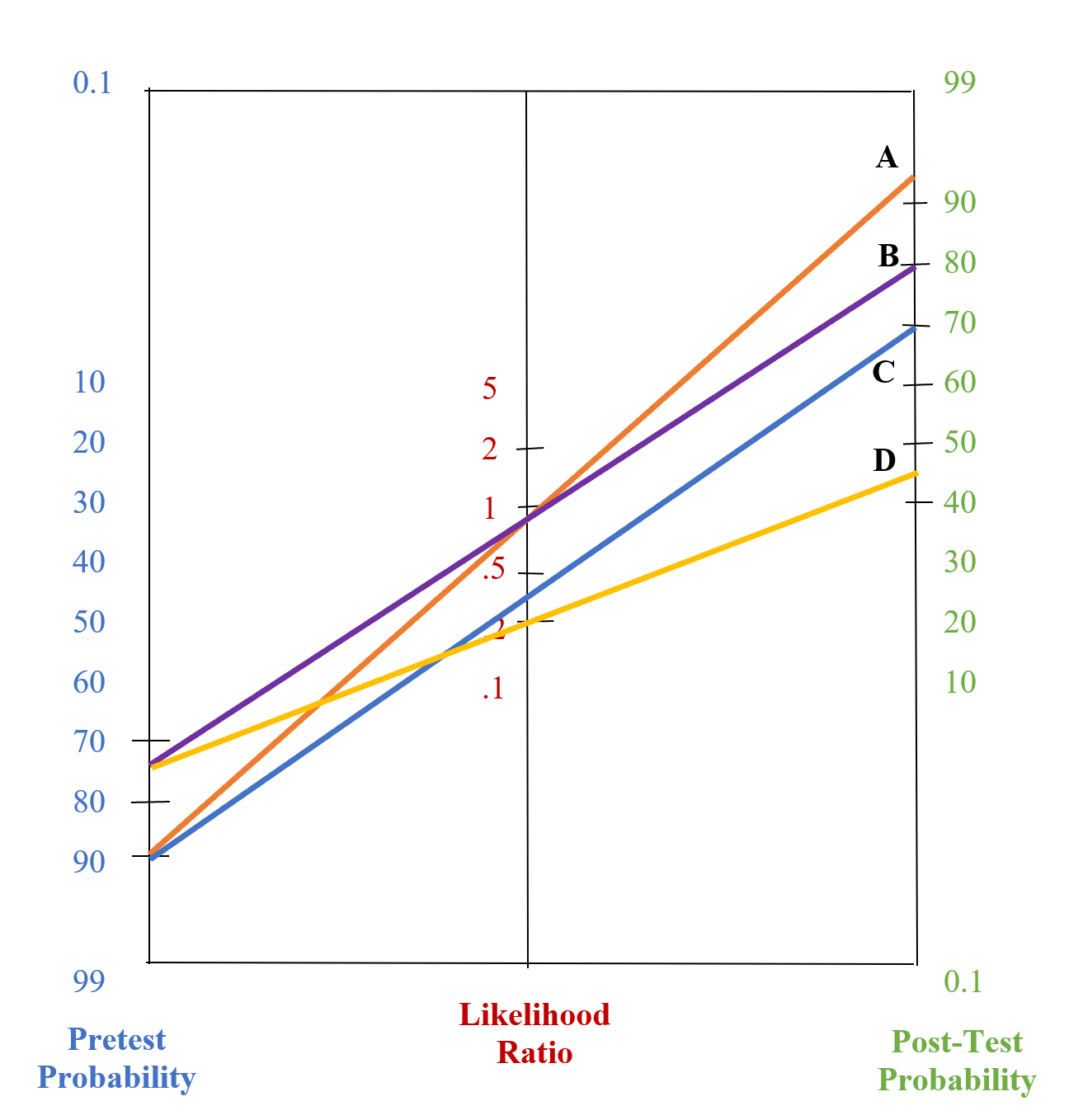


**Bayesian Nomogram**

***C: Pretest 90; LR .4; posttest~70***

*In this case you would use the negative likelihood ratio formula (1 - Sensitivity)/ (Specificity) and trace it on the nomogram. There is a more complicated set of equations you can use to calculate specifically but that is not expected of you now.*

## **Case 2**

Shown below: the distribution of WBC count for patients who became a zombie within a week (Disease) compared to the WBC count for patients who did not (Healthy)


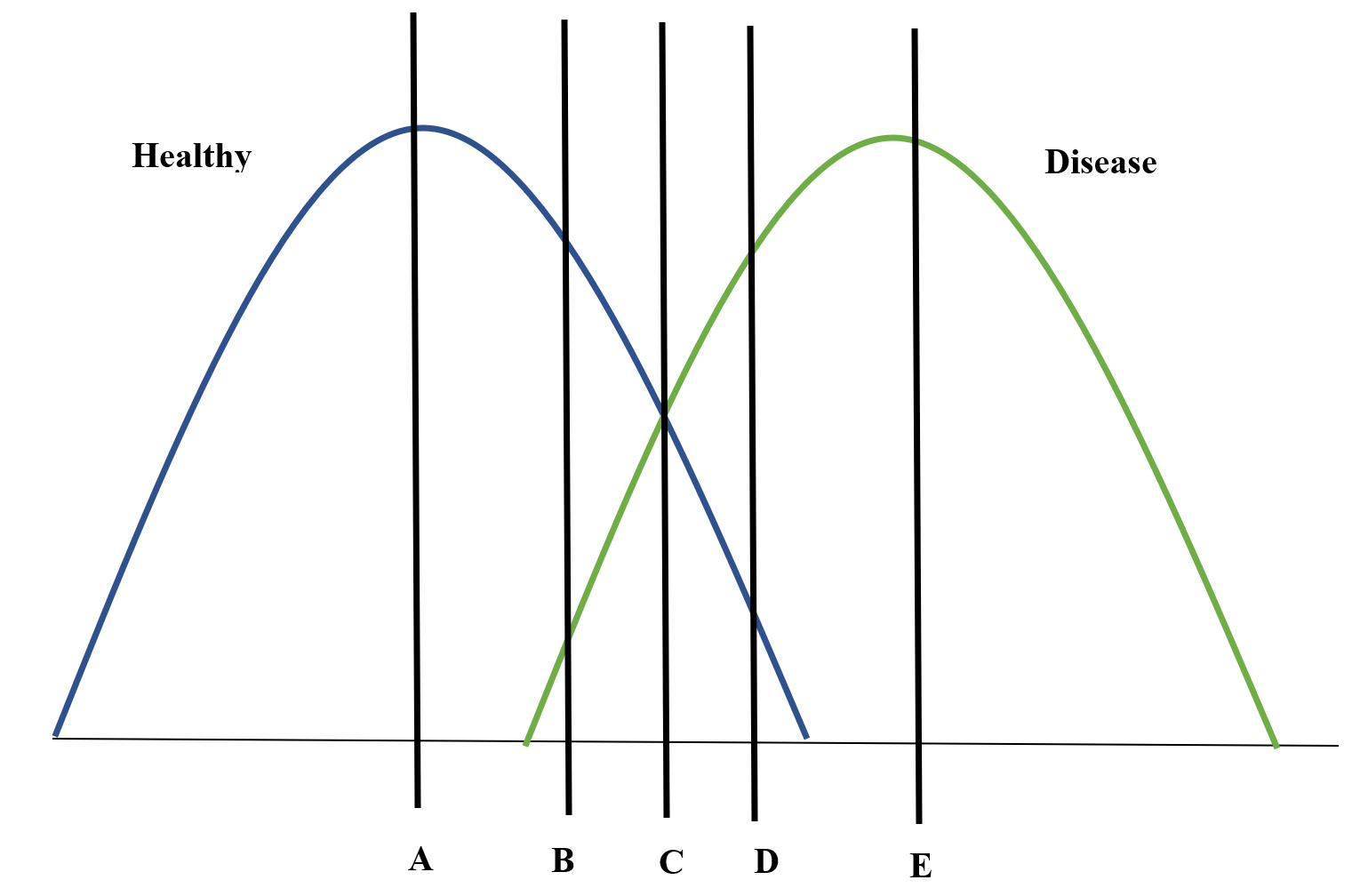


1. You read an angry online comment that claims the cut-off value for WBC count should be changed from D to B. How would each of the following be affected by this change?

Sensitivity: **Up** / down / same

Specificity: Up / **down** / same

Positive Predictive Value: Up / **down** / same

Negative Predictive Value: **Up** / down / same

*In this situation, true positives and false positives occur less frequently and true negatives and false negatives occur more frequently. As a rule of thumb, sensitivity and negative predictive value go up and down together while specificity and positive predictive value do the opposite of those.*

1. Which of the following would be the benefit of taking this comment’s advice?
   1. Increased ability to distinguish true positive from false positive results
   2. **Increased ability to distinguish true negative from false negative results**
   3. Fewer healthy individuals categorized as zombies
   4. Increased power of the study
   5. Reduced type I error
   6. p < .05

*B is another way of saying that the negative predictive value has increased, which is correct. A says the PPV has increased. C and E say false positives are reduced. D and F are irrelevant to changing the cut-off point.*

1. A new blood test becomes available that has a specificity of 85% and a sensitivity of 90%. Using the same pretest probability from question 1, if 1,000 people are tested, how many of these represent false positive results?
   1. **38**
   2. 75
   3. 212
   4. 675
   5. 750

You can use sensitivity, specificity, and the total number of people to back-calculate your way to values like TP, FP, TN, and FN. 75% of the 1,000 people will become zombies because of what we know from our pretest probability. Of those 750 individuals, 90% of them will test positive (TP) and 10% will test negative (FP). 250 individuals will not become zombies and 85% will test negative (TN) leaving 15% of 250 to test positive when actually negative (FP). 250 x .15 = 38

## **Case 3**

You’re still concerned about your friend, so you go online and find data regarding prophylactic administration of empathy in preventing patients from turning into zombies compared to the former standard of showing sympathy alone. You find a study where 200 patients were administered standardized units of empathy and 80 of them became zombies compared to a control group of 200 patients shown sympathy alone where 150 became zombies.

1. Based on the given information, calculate the Relative Risk (RR) and Relative Risk Reduction (RRR) for the use of empathy (experimental) vs. sympathy (control):
   1. RR .4; RRR .75
   2. RR .4; RRR .6
   3. **RR .53; RRR .47**
   4. RR .53; RRR .75
   5. RR .75; RRR .25
   6. RR .47; RRR 1.47

*EER: 80 / (80 + 120) = .4*

*CER: 150 / (150 + 50) = .75*

***RR*** *= EER/ CER = .4 / .75 = .53*

***RRR*** *= 1 – RR = 1 - .53 = .47*

1. Based on the given information, calculate the Absolute Risk Reduction (ARR) and Number Needed to Treat (NNT) for the use of empathy (experimental) vs. sympathy (control):
   1. **ARR .35; NNT 3**
   2. ARR .35; NNT 10
   3. ARR 2.03; NNT 1
   4. ARR 2.03; NNT 3
   5. ARR .10; NNT 10

EER: 80 / (80 + 120) = .4

CER: 150 / (150 + 50) = .75

**ARR** = |EER – CER| = |.4 - .75| = .35

**NNT** = 1 / ARR = 1 / .35 = 3 (round up to a whole number)
