# Supplementary material for: Peer-Developed Modules on Basic Biostatistics and Evidence-Based Medicine Principles for Undergraduate Medical Education
Source: MedEdPORTAL. 2020 Nov 24;16:11026. doi: 10.15766/mep_2374-8265.11026 (PMC7703476; doi:10.15766/mep_2374-8265.11026)
Supplement: Supplementary file 1 — Module 1 Study Design and Bias.pptxModule 1 Problem Set.docxModule 1 Problem Set Answer Key.docxModule 1 Formative Quiz.docxModule 1 Formative Quiz Answer Key.docxModule 2 Interpreting Data from Clinical Trials.pptxModule 2 Problem Set.docxModule 2 Problem Set Answer Key.docxModule 2 Formative Quiz.docxModule 2 Formative Quiz Answer Key.docxModule 3 Diagnostic and Therapy Trial Results.pptxModule 3 Problem Set.docxModule 3 Problem Set Answer Key.docxModule 3 Formative Quiz.docxModule 3 Formative Quiz Answer Key.docxImplementation Guide.docxPostsession Evaluation Survey.docx [file mep_2374-8265.11026-s001.zip › N. Module 3 Formative Quiz.docx]

**Module 3 Formative Quiz**

Instructions: Please complete the following quiz individually, closed-book, and within 10 minutes to assess your knowledge and understanding of the Module 3 material.

1. Which of the following is true as the cutoff is changed from C to A?


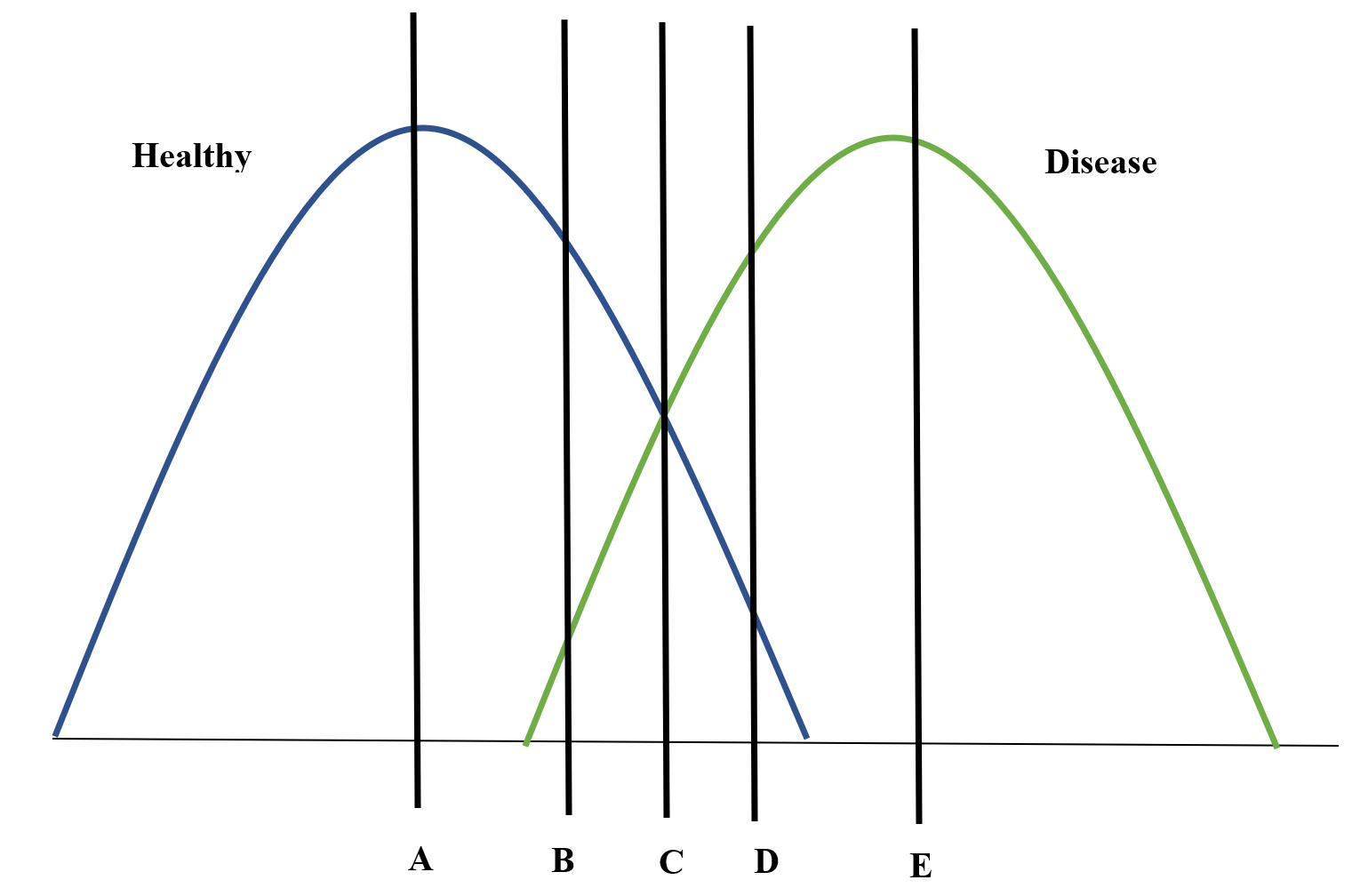


1. Specificity will increase and NPV will increase
2. Sensitivity will increase and PPV will decrease
3. Specificity will decrease and PPV will increase
4. Sensitivity will decrease and NPV will decrease
5. A new biomarker for prostate cancer has been discovered. The marker can be easily collected and measured from a patient’s serum. 1000 men aged 25-85 with an unknown cancer status were tested and the results compared against a gold standard. The sensitivity and specificity were determined to be 95% and 90% respectively. If the prevalence of prostate cancer is in the study population is 10%, what is the probability that a positive test truly indicates disease?
6. 0.85
7. 0.75
8. 0.39
9. 0.51
10. 0.65
11. A novel therapy, drug X, for DM II was developed to increase insulin sensitivity in patients. Drug X was compared to metformin for the reduction of all-cause mortality in 2500 patients over a period of 72 months. The results of the trial illustrated by the table below. Using the information below what is the number needed to treat?

|  | Drug X | Metformin |
| --- | --- | --- |
| Death by any Cause at 72 months | 75 | 125 |
| Alive at 72 months | 1175 | 1125 |

1. 56
2. 32
3. 25
4. 78
5. 16
6. A group of researchers identified 500 individuals with esophageal cancer based on a questionnaire given to patients at a nearby hospital. These patients compared to a group of 500 individuals who reported no history of esophageal cancer. In the questionnaire, both groups were asked if they smoke or have ever smoked. The prevalence of smoking was 33% among the esophageal cancer group and 14% among the group with no history of esophageal cancer. Which of the following would be used to calculate an Odds Ratio?

|  | Esophageal Cancer | No Esophageal Cancer |
| --- | --- | --- |
| Smoker | 165 | 70 |
| Never Smoked | 335 | 430 |

1. (165/335)/(70/430)
2. (165/70)/(335/430)
3. (70/430)/(165/335)
4. (335/430)/(165/70)

5. Two competing organizations that specialize in autoimmune disorders developed criteria for diagnosing Mixed Connective Tissue Disease (MCTD). With a sample size of 350 patients, of which 200 had MCTD, they evaluated which criteria would be better. Based on the table below, which statement below is the most accurate?

| **Criteria** | **Sensitivity** | **Specificity** |
| --- | --- | --- |
| **“Rheumatic Fanatics” Criteria** | 166/200 (83%) | 144/150 (96%) |
| **“AI: AutoImmune” Criteria** | 188/200 (94%) | 126/150 (84%) |

1. The “Rheumatic Fanatics” criteria would be more appropriate for ruling in a diagnosis.
2. The “AI: AutoImmune” criteria is superior to the “Rheumatic Fanatics” criteria when ruling in a diagnosis.
3. The “AI: AutoImmune” criteria would be more appropriate for ruling in a diagnosis.
4. The “Rheumatic Fanatics” criteria is superior to the “AI: AutoImmune” criteria when ruling out a diagnosis.

|  |  |
| --- | --- |
