# Supplementary material for: Peer-Developed Modules on Basic Biostatistics and Evidence-Based Medicine Principles for Undergraduate Medical Education
Source: MedEdPORTAL. 2020 Nov 24;16:11026. doi: 10.15766/mep_2374-8265.11026 (PMC7703476; doi:10.15766/mep_2374-8265.11026)
Supplement: Supplementary file 1 — Module 1 Study Design and Bias.pptxModule 1 Problem Set.docxModule 1 Problem Set Answer Key.docxModule 1 Formative Quiz.docxModule 1 Formative Quiz Answer Key.docxModule 2 Interpreting Data from Clinical Trials.pptxModule 2 Problem Set.docxModule 2 Problem Set Answer Key.docxModule 2 Formative Quiz.docxModule 2 Formative Quiz Answer Key.docxModule 3 Diagnostic and Therapy Trial Results.pptxModule 3 Problem Set.docxModule 3 Problem Set Answer Key.docxModule 3 Formative Quiz.docxModule 3 Formative Quiz Answer Key.docxImplementation Guide.docxPostsession Evaluation Survey.docx [file mep_2374-8265.11026-s001.zip › P. Implementation Guide.docx]

**Implementation Guide**

Instructions: The following guide has been prepared to provide detailed information on how to effectively set up and deliver the prepared learning materials. It is furthermore designed as a starting point in teaching methods, but please note that deliverance of the learning materials may vary based on the individual program due to varying class size, room space available, and student availability, among other factors.

**Recruiting Peer-Instructors**

For programs who desire to solicit peer-instructors to lead the in-class learning activities and problem-solving group sessions, we recommend distributing a survey that gauges student interest and willingness to participate in such a capacity. If the program features a routine Journal Club for medical students or a similar student organization, then those may be appropriate avenues for identifying and recruiting interested students. Ideally, 1 student would be recruited for every 30-40 students in the class (e.g. 4 students for a class size of 150). Based on our experiences conducting this activity, a 1:30-40 ratio has been found to be optimal for supervising 4 to 5 groups, with approximately 6 to 8 students per group. If more students than the number of instructors required express interest, then we recommend evaluating survey responses for student availability, commitment to teaching, and self-motivation, among other factors. Note that a background in evidence-based medicine (EBM) is not required, but an interest is certainly encouraged.

**Training Peer-instructors**

Note that the training process for peer-instructors will vary widely across institutions and programs due to varying curricula, access to faculty experts, and other resources. As such, some of our training methods, such as conducting monthly Journal Clubs, are not necessary, but are encouraged to strengthen knowledge and apply skills in EBM.

At our program, we trained as peer-instructors through a mix of monthly 1-hour Journal Club meetings, routine meetings with faculty experts, and self-directed studying for the United States Medical Licensing Examination (USMLE) Step One. As part of our monthly Journal Club meetings, we solicited practicing physicians to identify an article of interest and to facilitate the meeting. Over 2 academic years, the Journal Club meetings took place from August to April. At each meeting, we encouraged active participation among all members to form appropriate background questions, interpret the data, and evaluate the significance of the study’s conclusions. At our routine meetings with faculty experts, we discussed preparation for future Journal Club meetings, areas of focus that other students needed improvement on, and misconceptions in current understanding of EBM. For self-directed studying, we individually assessed our own gaps in knowledge and utilized various resources to address our weaknesses, aligning with what was tested as part of the USMLE Step One Content Outline.

The timeline for training at our program took approximately 18 months, but this was largely due to our 2-year preclinical curriculum. As we have prepared the materials and acknowledge that many programs now feature a shortened preclinical curriculum, the training process could theoretically take place over the course of 3 to 4 months under the guidance of a dedicated faculty expert in EBM.

**Encouraging Pre-session Preparation**

Encouraging students to review the prepared online modules (Appendices A, F, K) is highly recommended for them to obtain an optimal learning experience from the group problem-solving sessions. To optimize student preparation, we encourage sharing that understanding the information from the online modules will be necessary to complete the in-class learning activity and that a lack of review may lead to students feeling lost. However, we understand that individual student schedules may vary widely and as such, we recommend dedicating the first 10 minutes of each session to a brief review of topics related to each module.

**Creating Small Groups**

For the group problem-solving sessions, we recommend sorting students randomly into groups of 6 to 8 students. In our program with approximately 151 students, this random assortment would lead to 20 groups of students. However, for smaller programs, a corresponding decrease in group size may be appropriate, such as 4 students per group. Students may elect to sort themselves voluntarily, especially if the problem-solving sessions are conducted as a self-directed activity with a group of peers. However, for programs that desire to implement an in-class learning activity component, we strongly recommend random assortment to diversify thought processes and ensure that all students are placed within a group of similar size.

**Facilitating the Group Problem-Solving Sessions**

As students are completing the in-class learning activity, we recommend assigning the peer-instructors to a specific set of student groups. Subsequently, peer-instructors may be available to those groups to clarify any questions, encourage discussion, and guide students towards the correct thought process. Faculty content experts, if available, can also assist with any questions requiring additional clarification. Peer-instructors should also keep track of all students who participate and obtain their contact information in order to send the anonymous survey afterwards for feedback.

**Distributing the Survey**

At the end of the final session, all students who have participated in at least one session should receive a survey (Appendix P) to provide anonymous feedback. The survey should preferably be distributed through email or some other form of online communication to ensure delivery and ease of organizing responses.
