# Supplementary material for: Peer-Developed Modules on Basic Biostatistics and Evidence-Based Medicine Principles for Undergraduate Medical Education
Source: MedEdPORTAL. 2020 Nov 24;16:11026. doi: 10.15766/mep_2374-8265.11026 (PMC7703476; doi:10.15766/mep_2374-8265.11026)
Supplement: Supplementary file 1 — Module 1 Study Design and Bias.pptxModule 1 Problem Set.docxModule 1 Problem Set Answer Key.docxModule 1 Formative Quiz.docxModule 1 Formative Quiz Answer Key.docxModule 2 Interpreting Data from Clinical Trials.pptxModule 2 Problem Set.docxModule 2 Problem Set Answer Key.docxModule 2 Formative Quiz.docxModule 2 Formative Quiz Answer Key.docxModule 3 Diagnostic and Therapy Trial Results.pptxModule 3 Problem Set.docxModule 3 Problem Set Answer Key.docxModule 3 Formative Quiz.docxModule 3 Formative Quiz Answer Key.docxImplementation Guide.docxPostsession Evaluation Survey.docx [file mep_2374-8265.11026-s001.zip › Q. Postsession Evaluation Survey.docx]

**Instructions**: The information you provide in this survey will be collected anonymously. The data obtained through this survey will

**Peer-Led Evidence-Based Medicine (EBM) Evaluation Survey**

not be reported on an individual basis. Data will be used for improving program quality.

Please rate your opinion for the items below following the 5-Point Likert Scale:

**1 = Strongly Disagree; 2 = Disagree; 3 = Neutral; 4 = Agree; 5 = Strongly Agree**

To what extent do you agree or disagree with the following statements:

| 1. Peer-led EBM sessions were more conducive to learning problem-solving than traditional lectures. | **1** | **2** | **3** | **4** | **5** |
| --- | --- | --- | --- | --- | --- |
| 2. The team-based format facilitated learning. | **1** | **2** | **3** | **4** | **5** |
| 3. The material covered was Step 1 relevant. | **1** | **2** | **3** | **4** | **5** |
| 4. The case/content posted online prior to the session prepared me for the in-class problem solving. | **1** | **2** | **3** | **4** | **5** |
| 5. The goals of the sessions were clearly communicated. | **1** | **2** | **3** | **4** | **5** |
| 6. I recommend this model of EBM peer-led teaching to be implemented earlier in the curriculum. | **1** | **2** | **3** | **4** | **5** |
| 7. My level of understanding of EBM has improved after these sessions. | **1** | **2** | **3** | **4** | **5** |
| 8. The peer-led sessions increased my confidence in engaging in EBM topics during rounds in clerkship years. | **1** | **2** | **3** | **4** | **5** |
| 9. I would like to see this peer-led model applied to other disciplines. | **1** | **2** | **3** | **4** | **5** |
| 10. I would recommend this peer-led format to other medical schools. | **1** | **2** | **3** | **4** | **5** |
| 11. The presence of the faculty expert in the room was helpful. | **1** | **2** | **3** | **4** | **5** |
| 12. The peers leading the sessions were well prepared and effective. | **1** | **2** | **3** | **4** | **5** |

13. Free Response: Please share your feedback and/or comments regarding the effectiveness of the peer-led EBM sessions.
