# Supplementary material for: Peer-Developed Modules on Basic Biostatistics and Evidence-Based Medicine Principles for Undergraduate Medical Education
Source: MedEdPORTAL. 2020 Nov 24;16:11026. doi: 10.15766/mep_2374-8265.11026 (PMC7703476; doi:10.15766/mep_2374-8265.11026)
Supplement: Supplementary file 1 — Module 1 Study Design and Bias.pptxModule 1 Problem Set.docxModule 1 Problem Set Answer Key.docxModule 1 Formative Quiz.docxModule 1 Formative Quiz Answer Key.docxModule 2 Interpreting Data from Clinical Trials.pptxModule 2 Problem Set.docxModule 2 Problem Set Answer Key.docxModule 2 Formative Quiz.docxModule 2 Formative Quiz Answer Key.docxModule 3 Diagnostic and Therapy Trial Results.pptxModule 3 Problem Set.docxModule 3 Problem Set Answer Key.docxModule 3 Formative Quiz.docxModule 3 Formative Quiz Answer Key.docxImplementation Guide.docxPostsession Evaluation Survey.docx [file mep_2374-8265.11026-s001.zip › _Educational_Summary_Report_11026.pdf]

MedEdPORTAL® | The AAMC Journal of Teaching and Learning Resources

Original Publication

Open Access

Peer-Developed Modules on Basic Biostatistics and Evidence-Based Medicine Principles for Undergraduate Medical Education

Daniel H. Mai*, Jonathan S. Taylor-Fishwick, William Sherred-Smith, MS, Anthony Pang, MS, Justin Yaworsky, Sean Whitty, MS, Alex Lafever, MS, Cody Mcilvain, MS, Mark Schmitt, MBS, Michelle Rogers-Johnson, PhD, April Pace, MLS, Anca D. Dobrian, PhD

*Corresponding author: danielhmai94@gmail.com

Abstract

Introduction: Evidence-based medicine (EBM) is pivotal in shaping patient care, yet it is challenging to incorporate into undergraduate medical education (UME) due to a lack of dedicated resources within the preclinical curriculum. To address this challenge, we used a peer-led approach to explain difficult concepts through language that students can understand at their shared level of understanding. **Methods:** Four second-year medical students trained in EBM over 18 months by facilitating monthly journal clubs, ultimately leading to their involvement as peer-instructors. With input from a faculty expert, peer-instructors designed integrative PowerPoint modules and interactive problem sets on basic biostatistics and EBM principles. Assessment included formative quizzes with multiple attempts to achieve at least 80% to demonstrate mastery of core learning objectives. Afterwards, students were invited to provide feedback using a 5-point Likert scale survey. **Results:** Of second-year students who participated, all 151 demonstrated 80% competency on each quiz. Eighty-seven (58%) students completed the survey on which, 77% agreed/strongly agreed that their level of understanding of EBM improved after the peer-led sessions, 76% agreed/strongly agreed that the sessions were more conducive to learning compared to traditional lectures, and 94% agreed/strongly agreed that the material covered was relevant to the USMLE Step 1. **Discussion:** This peer-led approach has been rated as effective by learners, improving their ability to critically appraise and apply clinical evidence. To promote integration of EBM into UME, we have prepared modules, problem sets, quizzes, and an outline of the problem-solving sessions for universal adoption.

Keywords

Evidence-Based Medicine, Biostatistics, Peer-Learning, Self-Directed Learning, Flipped Classroom, Problem-Based Learning, Self-Assessment, Self-Regulated Learning, Statistics

Educational Objectives

By the end of this activity, learners will be able to:

1. Distinguish between different study designs and recognize the strengths and limitations associated with each.
2. Appraise the validity of evidence from a trial or clinical study.
3. Perform calculations needed to interpret the results of a trial or study.
4. Assess the statistical and clinical importance of evidence presented from a trial or clinical study.
5. Identify the strengths and limitations of peer-led instruction in the preclinical curriculum.

Citation:
Mai DH, Taylor-Fishwick JS, Sherred-Smith W, et al. Peer-developed modules on basic biostatistics and evidence-based medicine principles for undergraduate medical education. *MedEdPORTAL*. 2020;16:11026. https://doi.org/10.15766/mep_2374-8265.11026

Introduction

Evidence-based medicine (EBM) is a critically important component in the everyday practice of medicine as clinicians appraise and apply available evidence from relevant studies and trials into their diagnostic scripts and treatment regimens. Indeed, the practice of medicine itself has been founded upon evidence and data that support treatment regimens practiced today in hospitals throughout the nation.[1] Robust evidence is critically needed before clinicians may adopt novel strategies for treatment and management of diseases that have previously been resistant to most therapies, ultimately leading to improved patient outcomes.[1,2]

Furthermore, physicians who lack a strong background in EBM may fail to recognize and appreciate the cost-saving differences

Copyright © 2020 Mai et al. This is an open-access publication distributed under the terms of the Creative Commons Attribution license.

of alternative treatments, thereby leading to suboptimal care at higher costs. This relationship has been established in prior studies evaluating the management of patients with multisystemic diseases such as hypertension, diabetes and end-stage renal disease.[3] Consequently, the cost-saving nature of a refined EBM skillset is amplified beyond an individual instance of care.[4,5] Therefore, it is of utmost importance to integrate and reinforce EBM principles to develop a strong foundation for all physicians, starting with undergraduate medical education (UME), and continuing throughout clerkship training and residency.

Despite the importance of EBM instruction, many UME programs currently face barriers to expand their preclinical curricula and incorporate topics such as EBM due to lack of dedicated time, lack of financial resources to hire and train faculty, or difficulty generating and maintaining student interest.[6,7] Several programs have attempted innovative strategies to integrate more EBM instruction into their UME curricula; however, their approaches required substantial resources that limit such adoption.[8-10] For example, though effective, it is difficult and impractical to develop a standalone scientific literature course to expose students to the critical appraisal and application of clinical evidence.[11] Such a course would require formally trained faculty members to dedicate a significant amount of time and preparation.[16] Similarly, programs throughout the nation vary widely in the strength and size of their research departments, which leads to a wide variation of opportunities available for students to conduct research and to apply EBM principles.[11-14] Therefore, affordable and innovative solutions to address the gap in EBM instruction are critically needed.

In addition, most of current understanding of EBM principles in medical students stems from their preparation for the USMLE Step 1, which has been perceived as the main hurdle between obtaining a residency position at a program and specialty of their choice.[15,16] Though the USMLE Step 1 content outline incorporates several EBM principles as subtopics under a variety of basic biostatistical topics, there is no strong evidence to support that high examination scores correlate with clinical decision-making capabilities, including the application of EBM.[17,18] Furthermore, numerous medical students have reported that they feel rather unprepared to apply EBM principles towards their own practice of medicine.[19-21] Consequently, preparation for the USMLE Step 1 alone may not serve as sufficient preparation as students transition into the clerkship years and that additional instruction specific to EBM may benefit these learners.

To address these barriers in the UME curriculum, we proposed an innovative peer-led approach that involved problem-based EBM instruction. Problem-based learning has been well documented as an effective strategy in reinforcing concepts,[22-25] and peer-led instruction has been demonstrated as a successful means of disseminating information. Conceptually, peer-led sessions have important cognitive and social advantages over faculty-led sessions for complex topics including biostatistics and EBM.[26] Students have cognitive congruence because they share the same body of knowledge or knowledge framework. Students also share the same social roles, experiences, and motivation to learn through social congruence.[27] As such, peer-instructors are able to explain difficult concepts through this shared language and knowledge base.[28] Medical students are also more attuned to the perceived learning needs of their peers because of their shared experiences, allowing them to be more supportive.[29,30]

Studies in medical and health professions education showed that peer-teaching sessions are at least as effective as and possibly more effective than faculty-led sessions. A systematic review with a meta-analysis of 10 peer teaching studies found that the test scores of students assigned to peer instructors were significantly higher than those assigned to faculty instructors.[31] Peer-led instruction is also often cited as a solution to faculty shortages prevalent in health care education, and in part alleviates the need for resources, such as hiring a dedicated faculty member for such instruction.[28] Lastly, peer-led instruction empowers students to become instructors, strengthening their knowledge base and positioning them as leaders.[27,31,32]

Our peer-led approach stood out as a highly unique innovation in EBM instruction. There were few reports of peer-led instruction involving students within the same class for USMLE- or EBM-related topics in the literature. A thorough search of published literature found only two published research initiatives looking at the effects of peer-led sessions on USMLE Step 1 board scores, and those studies used peers from successive classes, not same-class peers.[33,34] In addition, systematic reviews of peer-led instruction identified epidemiology and biostatistics concepts as ideal for peer-led instruction, but there were very few initiatives in the published literature related to these concepts.[31]

From the available literature, current alternatives to EBM instruction lacked viable and resource-free solutions for universal adoption, lacked collaborative approaches to break down difficult concepts, focused too strongly on individualized topics rather than building a basic foundation in EBM, or lacked reinforcement of material through quizzing and assessments.[24,35-39] To address each of these gaps from the literature, our innovative approach

Copyright © 2020 Mai et al. This is an open-access publication distributed under the terms of the Creative Commons Attribution license.

involved medical students developing interactive and self-directed modules with corresponding assessments for students to educate themselves and their peers as well. Additionally, the self-paced design of the learning activities allowed for longitudinal learning at their own convenience. Furthermore, to align with student interest in their preparation for the USMLE Step 1, we have incorporated the same concepts in basic biostatistics as delineated in the content outline, but we have further expanded on the subtopics involving EBM principles as our primary focus.[17] These modules have been designed for independent study and for discussion in a team-based setting with the goal of successfully applying EBM principles through critically appraising and applying clinical evidence.

Methods

Peer-Teachers Training and Qualification

As peer instructors, we were a self-selected group of four students who willingly expressed a desire to lead the EBM organization at Eastern Virginia Medical School (EVMS). To allow students the opportunity to teach their peers, any second-year medical student who held a leadership position in the EBM organization was offered an opportunity to participate as a peer instructor. Over 2 academic years, we were trained and mentored for approximately 18 months in order to develop a strong background in basic biostatistics and EBM by a faculty expert who helped facilitate monthly 1-hour journal club meetings focused on EBM topics from August to April. To prepare us to become leaders within the EBM organization, the faculty expert delivered a series of informal sessions, which included an introduction to EBM and sessions in the critical appraisal of an intervention trial, a diagnostic test study, and a systematic review. As part of our additional training, we dedicated approximately 1 hour each week to identify scientific articles for presentation at our EBM organization meetings, self-study topics identified in the USMLE Step 1 Content Outline,[17] or meet with the faculty expert to review preparation techniques on how to effectively teach. During the spring semester of our second year, we each dedicated 1 additional hour per week to prepare the educational materials. The faculty expert reviewed all of the prepared materials prior to online posting and in-class presentation.

Note, however, that an 18-month training period was not required for implementation of this activity. We recognized that many programs have moved to a shortened preclinical curriculum. As such, preparation time for peer instructors could be shortened down 3 to 4 months under the guidance of a faculty expert knowledgeable in EBM, especially as we have alleviated the need to prepare the learning materials. However, the timeframe for preparation may depend on additional factors such as intrinsic motivation of the students and faculty availability to provide guidance on teaching methods.

Development

Our main goal was to teach preclinical medical students to critically appraise and apply clinical evidence from scientific literature and clinical trials in order to prepare them for both the USMLE Step 1 and for their upcoming clerkships. We focused on covering most topics on the USMLE Step 1 Course Content section on “Biostatistics, Epidemiology/Population Health, & Interpretation of the Medical Literature.”[17]

The full list of topics covered included the following:

- Types of study design
- Study biases
- Risk quantification
- Statistical hypotheses
- Statistical testing
- Validity assessment
- Evaluation of diagnostic tests

All peer instructors contributed to the development of all learning materials by reviewing in-class lectures and third-party resources, including First Aid for the USMLE Step 1 and Boards and Beyond.[40,41] We designed the questions and problem sets to reflect important concepts of basic biostatistics and EBM that we have previously encountered while preparing for the USMLE Step 1. Though we did not sample the active learning materials beforehand with a focus group of students or collect any evidence related to their efficacy, we required that each fellow peer instructor and faculty expert complete each activity to ensure quality and fairness in difficulty, timing, and clarity.

Overview

Our peer-led intervention consisted of three flipped classroom problem-solving sessions. Prior to each group problem-solving session, we instructed learners to review a PowerPoint presentation that was posted online, containing information relevant to each session (Appendices A, F, or K). While there is no time limit, each presentation took approximately 30 minutes to review. We did not require any prerequisite knowledge from students prior to reviewing the PowerPoints.

Each in-person session included 1 hour of live interactive small-group, problem-based learning. We arranged tables in a room to accommodate six to eight students at each table, with a whiteboard or scratch paper available at every table.

Copyright © 2020 Mai et al. This is an open-access publication distributed under the terms of the Creative Commons Attribution license.

Alternatively, students had the option to self-study at home and communicate online with their peers.

At the beginning of each problem-solving session, we dedicated 10 minutes to review difficult concepts from the presession modules in order to exchange ideas and identify knowledge gaps as a large group. Then students worked through the respective problem set (Appendices B, G, or L) with their small groups for 35 minutes. The questions were a combination of multiple-choice and open-ended formats, which students answered utilizing an open-textbook approach. Each group designated one student as the group facilitator to promote discussion and to record the responses on behalf of the group. With each problem, we encouraged students to discuss their thought process and rationale for why each question answer was correct or incorrect, and to use whiteboards or scratch paper to openly demonstrate the groups thought process behind each question. For difficult problems, we encouraged students to review the corresponding concept in a textbook or online while also encouraging peers to educate others through their own understanding and language.

After going through the problem set, we instructed students to spend 15 minutes reviewing the respective answer key (Appendices C, H, or M) and the corresponding explanations. For incorrect answers, we encouraged students to discuss their thought process with peers and correct any misconceptions.

After the session, we instructed students to complete the respective five or six-question formative quizzes (Appendices D, I, or N) individually at home, and on their own time. Students were told to complete them as a closed-textbook quiz with a 10-minute time limit. This activity in total took approximately 40 minutes (25 minutes to take the quiz and 15 minutes to review the answers). We required students to achieve at least 80% to demonstrate mastery of the objectives; students could attempt the quiz multiple times to meet the requirement. If a score of at least 80% was not achieved through the first attempt, we encouraged students to review the corresponding modules and preparation materials prior to attempting the quiz again. We utilized 80% as a threshold to demonstrate mastery because it aligned with the EVMS preclinical curriculum policy for all formative quizzes, but we furthermore believed that 80% was a sufficient percentage of items correct on a five- or six-item quiz to demonstrate basic understanding. We provided an answer key and explanatory notes for each of the quizzes (Appendices E, J, or O) and encouraged students to further discuss their thought process with peers and correct any misconceptions.

For more information related to the implementation of our learning activity, we developed an implementation guide (Appendix P) that details topics such as how to effectively recruit peer-instructors.

Student Learning Assessment

Following the last peer-led session we invited students to fill out an optional 12-item survey (Appendix Q) asked students to evaluate topics such as thoughts on the effectiveness of the sessions and self-improvement on an EBM-related topic on a 5-point Likert scale (1 = *strongly disagree*, 5 = *strongly agree*). We also included an additional item with an open-ended free response question for students to share their thoughts related to the effectiveness of the peer-led EBM sessions. The evaluation survey was constructed by the Office of Assessment at EVMS. As peer instructors, we excluded ourselves from filling out the survey. Note that the survey was not necessary for successful implementation of peer-led EBM instruction but allowed for assessment and feedback that may indicate areas for improvement.

Results

Each of the three EBM problem-solving sessions had an attendance of 151 second-year medical students who were divided randomly into small groups of six to eight students, with a total of 20 groups per session. Following each session, all 151 students completed the independent formative quiz and achieved a score of at least 80% correct, demonstrating mastery in the basic biostatistics and EBM principles tested.

A total of 87 students (58% response rate) completed the postsession evaluation survey. The summary of *agree* and *strongly agree* responses are summarized in the Table. Over 70% of the students agreed or strongly agreed on eight out of the 12 survey questions. More specifically, 77% agreed/strongly agreed that their level of understanding of EBM improved after the peer-led sessions, 76% agreed/strongly agreed that the sessions were more conducive to learning compared to traditional lectures, and 94% agreed/strongly agreed that the material covered was relevant to the USMLE Step 1.

Discussion

EBM training is essential for medical students in order for them to develop statistical reasoning, critical thinking, and critical appraisal skills. Given the importance of EBM skills to patient outcomes and practice improvement, it is important to identify educational initiatives, such as peer learning, that are effective and also valued by students. To address current barriers to

Copyright © 2020 Mai et al. This is an open-access publication distributed under the terms of the Creative Commons Attribution license.

Table. Student Responses of Agree/Strongly Agree to Survey Evaluating Peer-Led EBM Problem-Solving Sessions ($N = 87$)

Survey Statement	Agree/Strongly Agree (%)
Peer-led EBM sessions were more conducive to learning problem solving than traditional lectures.	76
The team-based format facilitated learning.	70
The material covered was Step 1 relevant.	94
The case/content posted online prior to the session prepared me for the in-class problem solving.	74
The goals of the sessions were clearly communicated.	87
I recommend this model of EBM peer-led teaching to be implemented earlier in the curriculum.	59
My level of understanding of EBM has improved after these sessions.	77
The peer-led sessions increased my confidence in engaging in EBM topics during rounds in clerkship years.	64
I would like to see this peer-led model applied to other disciplines.	35
I would recommend this peer-led format to other medical schools.	62
The presence of the faculty expert in the room was helpful.	71
The peers leading the sessions were well prepared and effective.	86

Abbreviation: EBM, evidence-based medicine

instruction in basic biostatistics and EBM in the UME curriculum, we have instituted a peer-led approach to develop a variety of learning materials with self-directed online PowerPoint modules, interactive group sessions with problem sets to reinforce learning, and formal assessments with formative quizzes to evaluate understanding.

Overall, the results demonstrated that students who participated in peer-led problem-solving sessions generally held a positive and favorable outlook on the experience, such that the majority of respondents (77%) had self-reported improvement in basic understanding and application of biostatistics and EBM principles. The survey results suggested student satisfaction for the majority of the respondents, as more than 80% reported the sessions to be helpful and relevant to their education, including preparation for the USMLE Step 1. From these positive results, we look favorably towards preparing medical students for USMLE Step 1 and clerkship training.

On a personal account, we found great satisfaction in educating our peers and strengthening our leadership and teaching skills. From preparing online modules to facilitating the in-class small-group problem-solving sessions, we strongly believe that our peer-led approach optimized learning by disseminating information through shared language and by allowing students to exchange ideas and thought processes, similar to the clinical decision-making process of practicing physicians.

Of note, the survey item of, “I would like to see this peer-led model applied to other disciplines,” had a 36% response rate for *agree* or *strongly agree*, which was anticipated. A literature review found that students were very positive towards peer-led sessions in biostatistics and EBM, but not necessarily towards other medical subjects.[42] However, we acknowledge that this response rate stood out as a stark contrast to the response rates of other survey items. As such, we believe that there is room for improvement by reaching out to students and gathering their thoughts on whether a peer-led model could be applied to other disciplines outside of basic biostatistics and EBM.

As demonstrated by the remaining survey responses with a greater than 50% response rate of *agree* or *strongly agree*, we concluded that a student-driven and peer-led approach benefitted our peers and may be adopted by other medical schools. Such an approach does not require any financial resources, integrates common goals (e.g., preparation for the USMLE Step 1 and clerkship training) into practice sessions, and provided a basic foundation to critically appraise and apply clinical evidence. Furthermore, we believe that our approach to EBM instruction may be most appropriate during the second year of medical school as students prepare for the clerkship years.

Based on the survey results, we believe that we have successfully met our objectives by improving our peers’ perception on knowledge and skills in basic biostatistics and EBM principles through peer-led instruction.

Despite these strengths, we also recognized that there may be limitations to our approach. Though our survey data demonstrated promising results, we also expected some degree of selection bias to the evaluation survey. For example, our peers may serve as our acquaintances and subsequently report more favorable outcomes with their self-improvement and satisfaction from the problem-solving sessions. A stronger and more objective evaluation would include USMLE Step 1 performance scores on basic biostatistics and EBM principles and in-class examinations. We further acknowledge that a robust infrastructure must exist to maintain the sustainability of this approach with ongoing support from module instructors and training and motivation of the medical students as peer-teachers. Similarly, successful implementation of the peer-led problem-solving sessions required individual motivation to review the online modules beforehand, active participation in the group discussions, and completion of the formative quizzes with subsequent review of answers and explanations.

Future plans include collecting quantitative data based on results from competency quizzes and examination items that test EBM concepts. Also, we will collect qualitative data from student focus

Copyright © 2020 Mai et al. This is an open-access publication distributed under the terms of the Creative Commons Attribution license.

groups to better understand attitudes of students towards peer-teaching of EBM that will inform us on needed refinements to improve learning outcomes and the overall learning experience. We are hopeful that, using the instructional materials and approaches disseminated in this report, other medical schools will consider adopting our peer-led model for instruction on basic biostatistics and EBM principles. While our second-year medical students participated in the sessions as part of an in-class learning activity, we designed all learning materials with the purpose of universal adoption and review for all preclinical students, regardless of months completed after matriculation into medical school. Furthermore, the learning modules did not require an in-class active learning component in large groups. They could have been applied in an individual learning context with independent completion and self-review of the problem sets and quizzes based on provided answer keys and explanatory notes. In closing, medical students through independent or small group self-directed learning may benefit from utilizing our modules and resources to improve outcomes on the USMLE Step 1 and to prepare themselves in applying EBM concepts during clerkships and towards their own practice of medicine.

Appendices

A. Module 1 Study Design and Bias.pptx
B. Module 1 Problem Set.docx
C. Module 1 Problem Set Answer Key.docx
D. Module 1 Formative Quiz.docx
E. Module 1 Formative Quiz Answer Key.docx
F. Module 2 Interpreting Data from Clinical Trials.pptx
G. Module 2 Problem Set.docx
H. Module 2 Problem Set Answer Key.docx
I. Module 2 Formative Quiz.docx
J. Module 2 Formative Quiz Answer Key.docx
K. Module 3 Diagnostic and Therapy Trial Results.pptx
L. Module 3 Problem Set.docx
M. Module 3 Problem Set Answer Key.docx
N. Module 3 Formative Quiz.docx
O. Module 3 Formative Quiz Answer Key.docx
P. Implementation Guide.docx
Q. Postsession Evaluation Survey.docx

All appendices are peer reviewed as integral parts of the Original Publication.

Daniel H. Mai: Medical Student, School of Medicine, Eastern Virginia Medical School

Jonathan S. Taylor-Fishwick: Medical Student, School of Medicine, Eastern Virginia Medical School

William Sherred-Smith, MS: Medical Student, School of Medicine, Eastern Virginia Medical School

Anthony Pang, MS: Medical Student, School of Medicine, Eastern Virginia Medical School

Justin Yaworsky: Medical Student, School of Medicine, Eastern Virginia Medical School

Sean Whitty, MS: Medical Student, School of Medicine, Eastern Virginia Medical School

Alex Lafever, MS: Medical Student, School of Medicine, Eastern Virginia Medical School

Cody Mcilvain, MS: Medical Student, School of Medicine, Eastern Virginia Medical School

Mark Schmitt, MBS: Medical Student, School of Medicine, Eastern Virginia Medical School

Michelle Rogers-Johnson, PhD: Director of Assessment, Eastern Virginia Medical School

April Pace, MLS: Librarian, Eastern Virginia Medical School

Anca D. Dobrian, PhD: Professor, Department of Physiological Sciences, Eastern Virginia Medical School

Disclosures
None to report.

Funding/Support
None to report.

Ethical Approval
Reported as not applicable.

References

1. Sackett DL. Evidence-based medicine. *Semin Perinatol*. 1997;21(1):3-5. https://doi.org/10.1016/S0146-0005(97)80013-4
2. Chung KC, Ram AN. Evidence-based medicine: the fourth revolution in American medicine? *Plast Reconstr Surg*. 2009; 123(1):389-398. https://doi.org/10.1097/PRS.0b013e3181934742
3. Lewis SJ, Orland BI. The importance and impact of evidence-based medicine. *J Manag Care Pharm*. 2004;10(5 Suppl A):3-5. https://doi.org/10.18553/jmcp.2004.10.S5-A.S3
4. Gahalaut P, Chauhan S, Mishra N, Rastogi M, Thakur R. Drug advertisements in two dermatology journals: a critical comparison of IJDVL and JAAD. *Indian J Dermatol Venereol Leprol*. 2014;80(2):115-121. https://doi.org/10.4103/0378-6323.129381

Copyright © 2020 Mai et al. This is an open-access publication distributed under the terms of the Creative Commons Attribution license.

5. Becker A, Dörter F, Eckhardt K, et al. The association between a journal's source of revenue and the drug recommendations made in the articles it publishes. *CMAJ*. 2011;183(5):544-548. https://doi.org/10.1503/cmaj.100951
6. Pagoto SL, Spring B, Coups EJ, Mulvaney S, Couto MF, Ozakinci G. Barriers and facilitators of evidence-based practice perceived by behavioral science health professionals. *J Clin Psychol*. 2007;63(7):695-705. https://doi.org/10.1002/jclp.20376
7. Hatala R, Guyatt G. Evaluating the teaching of evidence-based medicine. *JAMA*. 2002;288(9):1110-1112. https://doi.org/10.1001/jama.288.9.1110
8. Srinivasan M, Weiner M, Breitfeld PP, Brahmi F, Dickerson KL, Weiner G. Early introduction of an evidence-based medicine course to preclinical medical students. *J Gen Int Med*. 2002; 17(1):58-65. https://doi.org/10.1046/j.1525-1497.2002.10121.x
9. Keim SM, Howse D, Bracke P, Mendoza K. Promoting evidence-based medicine in preclinical medical students via a federated literature search tool. *Med Teach*. 2008;30(9-10): 880-884. https://doi.org/10.1080/01421590802258912
10. Premanath KF. Introduction of evidence-based medicine in undergraduate medical curriculum for development of professional competencies in medical students. *Curr Opin Anaesthesiol*. 2012;25(6):719-723.
11. Marusic A, Marusic M. Teaching students how to read and write science: a mandatory course on scientific research and communication in medicine. *Acad Med*. 2003;78(12):1235-1239. https://doi.org/10.1097/00001888-200312000-00007
12. Liamputtong P, ed. *Research Methods in Health: Foundations for Evidence-Based Practice*. Oxford University Press; 2010.
13. Mostafa SR, Khashab SK, Fouaad AS, Abdel Baky MA, Waly AM. Engaging undergraduate medical students in health research: students' perceptions and attitudes, and evaluation of a training workshop on research methodology. *J Egypt Public Health Assoc*. 2006;81(1-2):99-118.
14. Laskowitz DT, Drucker RP, Parsonnet J, Cross PC, Gesundheit N. Engaging students in dedicated research and scholarship during medical school: the long-term experiences at Duke and Stanford. *Acad Med*. 2010;85(3):419-428. https://doi.org/10.1097/ACM.0b013e3181ccc77a
15. Chen DR, Priest KC, Batten JN, Fragoso LE, Reinfeld BI, Laitman BM. Student perspectives on the "Step 1 Climate" in preclinical medical education. *Acad Med*. 2019;94(3):302-304. https://doi.org/10.1097/ACM.0000000000002565
16. Beck Dallaghan GL, Byerley JS, Howard N, Bennett WC, Gilliland KO. Medical school resourcing of USMLE Step 1 Preparation: questioning the validity of Step 1. *Med Sci Educ*. 2019;29(4): 1141-1145. https://doi.org/10.1007/s40670-019-00822-1
17. United States Medical Licensing Examination. USMLE Content Outline. Accessed November 15, 2019. https://www.usmle.org/pdfs/usmlecontentoutline.pdf
18. McGaghie WC, Cohen ER, Wayne DB. Are United States Medical Licensing Exam Step 1 and 2 scores valid measures for postgraduate medical residency selection decisions? *Acad Med*. 2011;86(1):48-52. https://doi.org/10.1097/ACM.0b013e3181ffacdb
19. Maggio LA. Educating physicians in evidence based medicine: current practices and curricular strategies. *Perspect Med Educ*. 2016;5(6):358-361. https://doi.org/10.1007/s40037-016-0301-5
20. Ilic D, Forbes K. Undergraduate medical student perceptions and use of evidence based medicine: a qualitative study. *BMC Med Educ*. 2010;10:58. https://doi.org/10.1186/1472-6920-10-58
21. Eyal L, Cohen R. Preparation for clinical practice: a survey of medical students' and graduates' perceptions of the effectiveness of their medical school curriculum. *Med Teach*. 2006;28(6):162-170. https://doi.org/10.1080/01421590600776578
22. Hosny S, Ghaly MS. Teaching evidence-based medicine using a problem-oriented approach. *Med Teach*. 2014;36(Suppl 1):S62-S68. https://doi.org/10.3109/0142159X.2014.886007
23. O'Neil J, Croniger C. Critical appraisal worksheets for integration into an existing small-group problem-based learning curriculum. *MedEdPORTAL*. 2018;14:10682. https://doi.org/10.15766/mep_2374-8265.10682
24. Pasarica M, Harris DM, Simms-Cendan J, Gorman AL. Collaborative learning activity utilizing evidence-based medicine to improve medical student learning of the lifestyle management of obesity. *MedEdPORTAL*. 2016;12:10426. https://doi.org/10.15766/mep_2374-8265.10426
25. Swanberg SM, Mi M, Engwall K. An integrated, case-based approach to teaching medical students how to locate the best available evidence for clinical care. *MedEdPORTAL*. 2017; 13:10531. https://doi.org/10.15766/mep_2374-8265.10531
26. Yu TC, Wilson NC, Singh PP, Lemanu DP, Hawken SJ, Hill AG. Medical students-as-teachers: a systematic review of peer-assisted teaching during medical school. *Adv Med Educ Pract*. 2011;2:157-172. https://doi.org/10.2147/AMEP.S14383
27. Ten Cate O, Durning S. Peer teaching in medical education: twelve reasons to move from theory to practice. *Med Teach*. 2007;29(6):591-599. https://doi.org/10.1080/01421590701606799
28. Burgess A, Dornan T, Clarke AJ, Menezes A, Mellis C. Peer tutoring in a medical school: perceptions of tutors and tutees. *BMC Med Educ*. 2016;16(1):85. https://doi.org/10.1186/s12909-016-0589-1
29. Hermann-Werner A, Gramer R, Erschens R, et al. Peer-assisted learning (PAL) in undergraduate medical education: an overview. *Z Evid Fortbild Qual Gesundhwes*. 2017;121:74-81. https://doi.org/10.1016/j.zefq.2017.01.001
30. Loda T, Erschens R, Loenneker H, et al. Cognitive and social congruence in peer-assisted learning—a scoping review. *PLoS*

Copyright © 2020 Mai et al. This is an open-access publication distributed under the terms of the Creative Commons Attribution license.

One. 2019;14(9):e0222224. https://doi.org/10.1371/journal.pone.0222224

31. Rees E, Sinha Y, Chitnis A, Archer J, Fotheringham V, Renwick S. Peer-teaching of evidence-based medicine. *Clin Teach*. 2014;11(4):259-263. https://doi.org/10.1111/tct.12144
32. Widyahening IS, Findyartini A, Ranakusuma RW, Dewiasty E, Harimurti K. Evaluation of the role of near-peer teaching in critical appraisal skills learning: a randomized crossover trial. *Int J Med Educ*. 2019;10:9-15. https://doi.org/10.5116/ijme.5c39.b55b
33. Alcamo AM, Davids AR, Way DP, Lynn J, Vandre DD. The impact of a peer-designed and -led USMLE Step 1 review course: improvement in preparation and scores. *Acad Med*. 2010; 85(10):S45-48. https://doi.org/10.1097/ACM.0b013e3181ed1cb9
34. Schwartz LF, Lineberry M, Park YS, Kamin CS, Hyderi AA. Development and evaluation of a student-initiated test preparation program for the USMLE Step 1 Examination. *Teach Learn Med*. 2018;30(2):193-201. https://doi.org/10.1080/10401334.2017.1386106
35. Nelson D, Bedinghaus JM. Team-based learning of EBM: case-control and cohort studies. *MedEdPORTAL*. 2013;9:9447. https://doi.org/10.15766/mep_2374-8265.9447
36. Weaver C. An approach to teaching medical students evidence-based medicine and basic research concepts. *MedEdPORTAL*. 2006;2:415. https://doi.org/10.15766/mep_2374-8265.415
37. Murrow L, Crites G, Goggans D, Gaines J, Stowe J, Richardson WS. Using evidence to inform risk or harm decisions. *MedEdPORTAL*. 2014;10:9990. https://doi.org/10.15766/mep_2374-8265.9990
38. Felix T, Richard D, Faber F, Zimmermann J, Adams N. Coffee talks: an innovative approach to teaching clinical reasoning and information mastery to medical students. *MedEdPORTAL*. 2015;11:10004. https://doi.org/10.15766/mep_2374-8265.10004
39. Boykan R, Chitkara M, Kenefick C, Messina C. An integrated practical evidence-based medicine curriculum: two small-group sessions to teach PICO question formation and searching strategies. *MedEdPORTAL*. 2013;9:9446. https://doi.org/10.15766/mep_2374-8265.9446
40. Le T, Bhushan V, Sochat M. First Aid for the USMLE Step 1 2019. 29th ed. McGraw-Hill Education; 2019.
41. Ryan J. Boards and beyond. Accessed November 15, 2019. https://www.boardsbeyond.com
42. Friel O, Kell D, Higgins M. The evidence base for peer-assisted learning in undergraduate medical education: a scoping study. *MedEdPublish*. 2018;1. https://doi.org/10.15694/mep.2018.0000044.1

Received: February 28, 2020
Accepted: July 21, 2020
Published: November 24, 2020

Copyright © 2020 Mai et al. This is an open-access publication distributed under the terms of the Creative Commons Attribution license.
